# Correction: Projecting contact matrices in 177 geographical regions: An update and comparison with empirical data for the COVID-19 era

**DOI:** 10.1371/journal.pcbi.1012454

**Published:** 2024-09-18

**Authors:** Kiesha Prem, Kevin van Zandvoort, Petra Klepac, Rosalind M. Eggo, Nicholas G. Davies, Centre for the Mathematical Modelling of Infectious Diseases COVID-19 Working Group, Alex R. Cook, Mark Jit

## Notice of Republication

After this article [1] was published, it came to light that there are a number of descriptive and coding errors in the article. To address this, the authors corrected the errors in their code and calculations and updated the text of their manuscript and Supporting Information. The changes do not have any substantial effect on the description of the quantitative results described in the article, or to the overall qualitative conclusions of the example model of physical distancing during the COVID-19 pandemic.

The article was republished on September 4, 2024, to replace the original article with the updated, corrected version, to provide an updated link to the repository with the corrected version of the code, and to replace the original versions of Figs 2, 3, 4, and 5 with the updated versions generated using the corrected code. S1 Text in [1] was also replaced with the updated, corrected version. Please download this article again to view the correct version. The originally published, uncorrected article and the republished, corrected article are provided here for reference.

The errors corrected are as follows:

- When constructing the household age matrix (HAM) for countries without household age structure data, the authors had previously divided the HAM of a POLYMOD or DHS country by “P_α_^c^” instead of “P_a_^c^” (i.e. with subscript alpha α instead of a). This was incorrect because they should be adjusting the household age structure by the age distribution of the household members instead of the individual. They have corrected the typographical mistake in the Supporting Information (S1 Text) and have updated the codes calculating HAM for countries without household age structure data.
- The authors defined $\lambda_{a_{i},\alpha }^{H}$ to allow for visitors to contribute to the total number of contacts through the parameter δ^H^. However, during the extrapolation of contacts at home, they had previously only accounted for household members, hence leaving out δ^H^ which represented visitors to the home from outside the household. They have now included δ^H^ in the calculations and text in S1 Text, previously missed in the extrapolation step. However, for work and school settings, they cannot distinguish between visitors and work/schoolmates, and as a result, they had to drop the δ^W^ and δ^S^ parameters from the estimation of contact patterns at the workplace and school.
- The calculations in the code have been corrected (to reflect the equations in the methods) when adjusting the matrices by the age-specific population ratios in the extrapolation step for contacts at other locations. Previously the authors multiplied by the vector containing the age-specific population ratio to the rows of λ _a,α_^O^ matrix instead of the columns; this is now corrected.
- In the extrapolation step to derive the frequency-dependent age- and location-specific contact matrices at the workplace and school, the authors have revised the calculation in the code and text in S1 Text. Previously, in the extrapolation step (in S1 Text), contact matrices at the workplace and school were adjusted by age-specific population ratios in addition to the age-specific working and schooling populations. However, when deriving the age-specific contact matrices at the workplace and school using a frequency-dependent approach, the authors realized that this step is unnecessary. This term has been removed from the text in the Supporting Information. However, as not all working individuals went to work on the day of the survey, the contact matrices at the workplace have been adjusted by the proportion of individuals who went to work on the day of the survey. The equations in section A.5. of S1 Text have been changed to reflect this.
- The authors have corrected the errors in their code described above and have shared their corrected code here: https://github.com/kieshaprem/synthetic-contact-matrices.
- A note has been added to the Abstract, Methods, and Supporting Information to highlight that the matrices only cover populations up to age 80 years.
- The correction of the code used to generate the figures has led to minor changes to the detailed quantitative results shown in Figs 2, 3, 4, and 5 and in the corrected versions of Fig 2 and Fig 3 the updated synthetic matrices have slightly better correlations with the empirical matrices than the previous synthetic matrices.

The article’s main conclusions are not affected by the correction of the descriptive and coding errors. A summary of the changes to the results following the errata can be found in S3 File provided with this notice.

A member of *PLOS Computational Biology*’s Editorial Board reviewed the updates and advised that all issues were satisfactorily addressed in the republished version of the article.

## Supporting information

S1 File Originally published, uncorrected article. (PDF)

S2 File Republished, corrected article. (PDF)

S3 File Summary of changes to the results following the errata. (DOCX)
