# Supplementary material for: Projecting contact matrices in 177 geographical regions: An update and comparison with empirical data for the COVID-19 era
Source: PLoS Comput Biol. 2021 Jul 26;17(7):e1009098. doi: 10.1371/journal.pcbi.1009098 (PMC8354454; doi:10.1371/journal.pcbi.1009098)
Supplement: S1 Text — (DOCX) [file pcbi.1009098.s001.docx]

Projecting contact matrices in 177 geographical regions: an update and comparison with empirical data for the COVID-19 era

Supplementary Material

Kiesha Prem, Kevin van Zandvoort, Petra Klepac, Rosalind M Eggo, Nicholas G Davies,

Centre for the Mathematical Modelling of Infectious Diseases COVID-19 Working Group,

Alex R Cook, Mark Jit[[1]](#footnote-1)

Contents

[List of Tables 1](#_Toc144640206)

[List of Figures 1](#_Toc144640207)

[A. Materials and methods 3](#_Toc144640208)

[A.1. Population age structure 3](#_Toc144640209)

[A.2. Household age structure 3](#_Toc144640210)

[A.2.1. Household data 3](#_Toc144640211)

[A.2.2. Household age matrix (HAM) 3](#_Toc144640212)

[A.2.3. Household age matrix (HAM) validation for POLYMOD and DHS countries 5](#_Toc144640213)

[A.3. Working population 5](#_Toc144640214)

[A.4. School-going population 5](#_Toc144640215)

[A.5. Age- and location-specific contacts 5](#_Toc144640216)

[A.6. Age- and location-specific contacts by rural and urban areas 9](#_Toc144640217)

[A.7. Age-stratified compartmental model of the physical distancing interventions for COVID-19 9](#_Toc144640218)

[A.8. Hierarchical model of POLYMOD contact data 11](#_Toc144640219)

[References 14](#_Toc144640220)

# List of Tables

| A | Description of Demographic Household Surveys from 43 countries..................................................4 |
| --- | --- |
| B | Geographical regions included in the study.......................................................................................7 |
| C | Parameters of the age-stratified SEIR model...................................................................................10 |

# List of Figures

| A | Age-stratified stochastic SEIR model.................................................................................................9 |
| --- | --- |
| B | Set of up to four adjacent age groups..............................................................................................12 |
| C | Smoothness between successive age groups on the prior distribution............................................13 |

# Materials and methods

## Population age structure

The United Nations Population Division provides the population age composition in five-year age intervals $P_{a}$ (i.e., number of individuals in age group $a$) for the 177 geographical regions in this study [1]. They also present the estimation of rural and urban population age compositions for the 177 geographical regions [2].

## Household age structure

### Household data

We extracted the household data from POLYMOD contact study [3]—for the eight POLYMOD countries: Belgium, Germany, Finland, the United Kingdom, Italy, Luxembourg, the Netherlands, and Poland. The Demographic Household Surveys (DHS) provides nationally-representative household surveys of the whole population for several low- and middle-income countries [4]. **Table A** lists the 43 DHS countries included in the study. The largest survey conducted in India in 2015 included ~3 million individuals from ~600,000 households. The DHS also provides data for rural and urban areas, allowing us to derive rural-urban household age matrices.

### Household age matrix (HAM)

To estimate the age-specific contacts at home for county $c$, we need to first construct the household age matrix (HAM) for country $c$. The HAM of country $c$, $\mathbf{h}_{a,\alpha}^{c}$, represents the mean number of household members of age $\alpha$ of an individual aged $a$. Although this can be easily derived for the eight POLYMOD and 43 DHS countries, it is not straightforward for other countries or geographical regions without available household structure data. In Prem et al. [5], we provide detailed steps to project a country’s household age structure.

1. **Population ratio matrices:** For the 51 POLYMOD and DHS countries, we first derive their population ratio matrices $\boldsymbol{\Pi}_{a,\alpha}^{c}$ by dividing the elements of HAM ($\mathbf{h}_{a,\alpha}^{c}$) by the proportion of the population of country $c$ aged $\alpha$, $P_{\alpha}^{c}$. The population ratio matrix of a country measures the propensity of having a household member aged $\alpha$ for an individual aged $a$ after adjusting for the population age structure. Because we know the population age composition for the 177 geographical regions, we can project their HAM by extrapolating the relationship between $P_{\alpha}^{c}$ and $\mathbf{h}_{a,\alpha}^{c}$ as estimated from the POLYMOD and DHS countries, and applying that to the country’s population age profile. We could simply use the mean population ratio matrix of the POLYMOD and DHS countries to derive the HAM of country $c$, $\mathbf{h}_{a,\alpha}^{c}$, as follows $P_{\alpha}^{c}\overline{\boldsymbol{\Pi}_{a,\alpha}^{c}}$.
2. **HAM weights:** However, as household age structures vary across countries in different stages of development and with different demographics, we use 14 country characteristics from the World Bank and United Nations Educational, Scientific and Cultural Organization Institute for Statistics (UIS) databases [6] to quantify the similarity of these 126 geographical regions (with no available household data). The 14 country characteristics include gross domestic product per capita, total fertility rate and adolescent fertility rate, population density, population growth rate, under-five mortality rate, the life expectancy of males and females, mortality rates of males, risk of maternal death, mortality from road traffic injury, the incidence of tuberculosis, as proxies for overall health, internet penetration rate, and secondary school education attainment levels in the country. We then calculate the pairwise Euclidean distances of each of the 14 standardized variables of these 126 geographical regions with the 51 POLYMOD and DHS countries. We generated 10 000 bootstrap samples of the pairwise distances between countries (between indicators) and selected the combination that maximized the correlation between $P_{\alpha}^{c}$ and $\sum_{\alpha} P_{\alpha}^{c}\mathbf{h}_{a,\alpha}^{c}$. For each of the 126 geographical regions, the reciprocal of the bootstrapped pairwise distances provide the weights which we then use to derive the weighted mean of the population ratio matrices of the 51 POLYMOD and DHS countries.
3. **HAM projection:** Using the derived weighted mean of the population ratio matrices, we project the HAM $\mathbf{h}_{a,\alpha}^{c}$ for geographical region $c$.

**Table A.** Description of Demographic Household Surveys from 43 countries.

| **Country** | **Country code** | **Survey year** | **Survey Type** | **Number of households** | **Number of individuals** |
| --- | --- | --- | --- | --- | --- |
| Afghanistan | AFG | 2015 | Standard DHS | 24395 | 203708 |
| Angola | AGO | 2015 | Standard DHS | 16109 | 74902 |
| Bangladesh | BGD | 2014 | Standard DHS | 17300 | 81624 |
| Benin | BEN | 2017 | Standard DHS | 14156 | 74673 |
| Bolivia (Plurinational State of) | BOL | 2008 | Standard DHS | 19564 | 77757 |
| Cambodia | KHM | 2014 | Standard DHS | 15825 | 74122 |
| Cameroon | CMR | 2011 | Standard DHS | 14214 | 72622 |
| Chad | TCD | 2014 | Standard DHS | 17233 | 99620 |
| Colombia | COL | 2015 | Standard DHS | 44614 | 162459 |
| Congo | COG | 2011 | Standard DHS | 11632 | 51449 |
| Democratic Republic of the Congo | COD | 2013 | Standard DHS | 18171 | 95949 |
| Dominican Republic | DOM | 2013 | Standard DHS | 11464 | 41267 |
| Ethiopia | ETH | 2016 | Standard DHS | 16650 | 75224 |
| Ghana | GHA | 2017 | Standard DHS | 11835 | 43945 |
| Guatemala | GTM | 2014 | Standard DHS | 21383 | 102510 |
| Guinea | GIN | 2018 | Standard DHS | 7912 | 49543 |
| Guyana | GUY | 2009 | Standard DHS | 5632 | 22845 |
| Haiti | HTI | 2016 | Standard DHS | 13405 | 59547 |
| Honduras | HND | 2011 | Standard DHS | 21362 | 100555 |
| India | IND | 2015 | Standard DHS | 601509 | 2869043 |
| Indonesia | IDN | 2017 | Standard DHS | 47963 | 197723 |
| Kenya | KEN | 2014 | Standard DHS | 36430 | 153840 |
| Kyrgyzstan | KGZ | 2012 | Standard DHS | 8040 | 35805 |
| Lesotho | LSO | 2014 | Standard DHS | 9402 | 40197 |
| Liberia | LBR | 2013 | Standard DHS | 9333 | 48219 |
| Malawi | MWI | 2015 | Standard DHS | 26361 | 120492 |
| Maldives | MDV | 2016 | Standard DHS | 6050 | 32656 |
| Mali | MLI | 2018 | Standard DHS | 9510 | 54571 |
| Nepal | NPL | 2016 | Standard DHS | 11040 | 49064 |
| Niger | NER | 2012 | Standard DHS | 10750 | 64011 |
| Nigeria | NGA | 2018 | Standard DHS | 40427 | 188010 |
| Pakistan | PAK | 2017 | Standard DHS | 14540 | 100869 |
| Peru | PER | 2012 | Continuous DHS | 27218 | 103211 |
| Philippines | PHL | 2017 | Standard DHS | 27496 | 120273 |
| Senegal | SEN | 2017 | Continuous DHS | 8380 | 78950 |
| Sierra Leone | SLE | 2013 | Standard DHS | 12629 | 75299 |
| South Africa | ZAF | 2016 | Standard DHS | 11083 | 38850 |
| Timor-Leste | TLS | 2016 | Standard DHS | 11502 | 61496 |
| Togo | TGO | 2013 | Standard DHS | 9549 | 46577 |
| Uganda | UGA | 2016 | Standard DHS | 19588 | 91167 |
| Viet Nam | VNM | 2005 | Standard AIS | 6337 | 26833 |
| Zambia | ZMB | 2018 | Standard DHS | 12831 | 65454 |
| Zimbabwe | ZWE | 2015 | Standard DHS | 10534 | 43706 |

### Household age matrix (HAM) validation for POLYMOD and DHS countries

In section A.2.2, we describe the methods used to project the HAM of a country.

We perform internal validation using leave-one-out validation to verify that the HAM describing household structure could be reverse-engineered for the POLYMOD and DHS countries for which empirical household age matrices were available. The steps involve projecting that country’s household age structure as if it were unknown (i.e., following steps 1–3 described in section A.2.2) and then comparing against the empirical household data to assess the method’s performance.

## Working population

We allow the number of age-specific contacts made at the workplace to depend on the age structure of the workforce. To derive the working population matrices for each geographical location, we used the 2019 labour force participation rate by sex and 5-year age groups $w_{a}$ for the 177 geographical regions from the International Labour Organization (ILO) [7].

After estimating the working population distribution of ages $w_{a}^{c}$, we project the age-specific contact patterns in the workplace for non-POLYMOD countries.

## School-going population

Similarly, for age-specific contact patterns at school, we construct the age-specific school-going population, including teachers, before projecting the contact patterns in schools. Information on enrolment rates, age ranges of students, pupil-to-teacher ratios by education levels (i.e., pre-primary, primary, secondary, and tertiary) were obtained from UIS [6] and the distribution of teachers by age from Organisation for Economic Co-operation and Development (OECD) [8].

To project the school population, we perform the following steps:

1. **Number of students estimation:** We first estimate the proportion of students in each age interval using the country-specific enrolment rates and the starting and ending ages of students by education level. Together with the population age structure $P_{a}^{c}$, we then estimate the number of students $s_{a}^{c}$ aged $a$ in country $c$. We also estimate the number of students in each education level $l$, $s_{l}^{c}$, for the education levels pre-primary, primary, secondary, and tertiary.
2. **Number of teachers estimation:** After estimating the number of students for each education level in country $c$, $s_{l}^{c}$, we use the country-specific pupil-to-teacher ratio to estimate the number of teachers for each education level, $l$, $t_{l}^{c}$. Together with labour force participation rates by aged and the distribution of teachers by ages, we then estimate the number of teachers $t_{a}^{c}$ aged $a$ in country $c$.
3. **School-going population construction:** We project the school population distribution of ages, as follows:

$$\mathbf{S}_{a}^{c}=\frac{s_{a}^{c}+t_{a}^{c}}{P_{a}^{c}}.$$

4. The elements of $\mathbf{S}_{a}^{c}$ present the proportion of the population aged $a$ in schools in country $c$.

## Age- and location-specific contacts

In Prem et al. (2017) [5], we employed a Bayesian hierarchical modelling framework to estimate modelling to estimate the proclivity of age- and location-specific contact patterns in the POLYMOD countries. The framework estimates both individual-level and population-level parameters and addresses the multi-level structure of the data, accounting for repeat measurements of contacts made in different settings by the same individual in the data. The parameter $\lambda_{a,\alpha}^{L}$ is the key estimand in the model, and it quantifies typical contact rates between individuals of age groups $a$ and $\alpha$ at location $L$. With the projected populations at home, work and school available for the 177 geographical regions (listed in **Table B**), we can now deduce the possible frequency-dependent age- and location-specific contact matrices:

- **Age-specific contacts at home:** Given the projected HAM $\mathbf{h}_{a,\alpha}^{c}$ for country $c$, we can now deduce the possible age-specific contact patterns at home for country c to be:

$$\mu_{a,\alpha}^{H,c}=\lambda_{a,\alpha}^{H}\times\mathbf{(h}_{a,\alpha}^{c}+\delta_{H}).$$

- **Age-specific contacts in the workplace:** With the age-specific working populations for the 177 geographical regions $w_{a}^{c}$ and the proportion of the survey respondent going to work $p_{\text{survey}}^{W}$, we can infer the age-specific contact in the workplace for country $c$ by the following expression:

$$\mu_{a,\alpha}^{W,c}=\lambda_{a,\alpha}^{W}\times(\frac{w_{a}^{c}}{w_{a}^{POLYMOD}} \times p_{\text{survey}}^{W}).$$

- **Age-specific contact in school:** With the projected school-going population available for the 177 geographical regions $\mathbf{S}_{a}^{c}$ and the proportion of the survey respondent in school-going ages going to school $p_{\text{survey}}^{S}$, we can now deduce the possible age-specific contact patterns in school for country c, as follows:

$$\mu_{a,\alpha}^{S,c}=\lambda_{a,\alpha}^{S}\times\left( \frac{\mathbf{S}_{a}^{c}}{\mathbf{S}_{a}^{POLYMOD}}\times p_{\text{survey}}^{S} \right).$$

- **Age-specific contact at other locations:** We estimate the possible age-specific contacts at other locations (i.e., non-home, work, or school) for country c, as follows:

$$\mu_{a,\alpha}^{O,c}=\lambda_{a,\alpha}^{O}\times\frac{P_{\alpha}^{c}}{P_{\alpha}^{POLYMOD}}.$$

**Table B.** Geographical regions included in the study.

| **Regions** | **County code** | **Regions** | **County code** |
| --- | --- | --- | --- |
| Afghanistan | AFG | Ecuador | ECU |
| Albania | ALB | Egypt | EGY |
| Algeria | DZA | El Salvador | SLV |
| Angola | AGO | Equatorial Guinea | GNQ |
| Argentina | ARG | Eritrea | ERI |
| Armenia | ARM | Estonia | EST |
| Austria | AUT | Eswatini | SWZ |
| Azerbaijan | AZE | Ethiopia | ETH |
| Bahamas | BHS | Fiji | FJI |
| Bahrain | BHR | Finland | FIN |
| Bangladesh | BGD | France | FRA |
| Barbados | BRB | Gabon | GAB |
| Belarus | BLR | Gambia | GMB |
| Belgium | BEL | Georgia | GEO |
| Belize | BLZ | Germany | DEU |
| Benin | BEN | Ghana | GHA |
| Bhutan | BTN | Greece | GRC |
| Bolivia (Plurinational State of) | BOL | Guatemala | GTM |
| Bosnia and Herzegovina | BIH | Guinea | GIN |
| Botswana | BWA | Guinea-Bissau | GNB |
| Brazil | BRA | Guyana | GUY |
| Brunei Darussalam | BRN | Honduras | HND |
| Bulgaria | BGR | Hungary | HUN |
| Burkina Faso | BFA | Iceland | ISL |
| Burundi | BDI | India | IND |
| Cabo Verde | CPV | Indonesia | IDN |
| Cambodia | KHM | Iran (Islamic Republic of) | IRN |
| Cameroon | CMR | Iraq | IRQ |
| Canada | CAN | Ireland | IRL |
| Central African Republic | CAF | Israel | ISR |
| Chad | TCD | Italy | ITA |
| Chile | CHL | Jamaica | JAM |
| China | CHN | Jordan | JOR |
| China, Hong Kong SAR | HKG | Kazakhstan | KAZ |
| China, Macao SAR | MAC | Kenya | KEN |
| Colombia | COL | Kuwait | KWT |
| Comoros | COM | Kyrgyzstan | KGZ |
| Congo | COG | Lao People's Democratic Republic | LAO |
| Costa Rica | CRI | Latvia | LVA |
| C√¥te d'Ivoire | CIV | Lesotho | LSO |
| Croatia | HRV | Liberia | LBR |
| Cuba | CUB | Libya | LBY |
| Cyprus | CYP | Lithuania | LTU |
| Czechia | CZE | Luxembourg | LUX |
| Dem. People's Republic of Korea | PRK | Madagascar | MDG |
| Democratic Republic of the Congo | COD | Malawi | MWI |
| Denmark | DNK | Malaysia | MYS |
| Djibouti | DJI | Maldives | MDV |
| Dominican Republic | DOM | Mali | MLI |

| **Regions** | **County code** | **Regions** | **County code** |
| --- | --- | --- | --- |
| Malta | MLT | Serbia | SRB |
| Mauritania | MRT | Sierra Leone | SLE |
| Mauritius | MUS | Singapore | SGP |
| Mexico | MEX | Slovakia | SVK |
| Mongolia | MNG | Slovenia | SVN |
| Montenegro | MNE | Solomon Islands | SLB |
| Morocco | MAR | South Africa | ZAF |
| Mozambique | MOZ | South Sudan | SSD |
| Myanmar | MMR | Spain | ESP |
| Namibia | NAM | Sri Lanka | LKA |
| Nepal | NPL | State of Palestine | PSE |
| Netherlands | NLD | Sudan | SDN |
| New Zealand | NZL | Suriname | SUR |
| Nicaragua | NIC | Sweden | SWE |
| Niger | NER | Switzerland | CHE |
| Nigeria | NGA | Syrian Arab Republic | SYR |
| North Macedonia | MKD | Tajikistan | TJK |
| Norway | NOR | Thailand | THA |
| Oman | OMN | Timor-Leste | TLS |
| Pakistan | PAK | Togo | TGO |
| Panama | PAN | Tonga | TON |
| Papua New Guinea | PNG | Trinidad and Tobago | TTO |
| Paraguay | PRY | Tunisia | TUN |
| Peru | PER | Turkey | TUR |
| Philippines | PHL | Turkmenistan | TKM |
| Poland | POL | Uganda | UGA |
| Portugal | PRT | Ukraine | UKR |
| Puerto Rico | PRI | United Arab Emirates | ARE |
| Qatar | QAT | United Kingdom | GBR |
| Republic of Korea | KOR | United Republic of Tanzania | TZA |
| Republic of Moldova | MDA | United States of America | USA |
| Romania | ROU | Uruguay | URY |
| Russian Federation | RUS | Uzbekistan | UZB |
| Rwanda | RWA | Vanuatu | VUT |
| Saint Lucia | LCA | Venezuela (Bolivarian Republic of) | VEN |
| Saint Vincent and the Grenadines | VCT | Viet Nam | VNM |
| Samoa | WSM | Yemen | YEM |
| Sao Tome and Principe | STP | Zambia | ZMB |
| Saudi Arabia | SAU | Zimbabwe | ZWE |
| Senegal | SEN |  |  |

## Age- and location-specific contacts by rural and urban areas

The population age structure at the various locations—in the household, workplaces, schools, general community—varies by rural and urban subregions of country. Hence, we would expect the contact patterns within a country to also vary by rural and urban subregions. To account for these differences, we also stratified the age- and location-specific contact matrices by rural-urban areas.

The United Nations Population Division provides the estimation of rural and urban population age compositions for the 177 geographical regions [2]. The nationally-representative DHS household surveys provide data for rural and urban areas, allowing us to derive rural-urban HAM and project rural-urban HAM for the countries with no available household data (following steps 1–3 described in section A.2.2). ILO provides the age-specific labour force participation rates by rural and urban regions [9], allowing us to determine the working population age structure, $w_{a}^{c}$, for country $c$ by rural-urban subregions. Using the differences in rural and urban schools’ pupil-to-teacher from the OECD [10], we construct rural and urban school-going population age structures, $\mathbf{S}_{a}^{c}$.

After projecting the populations at home, work and school for rural and urban subregions, we can then deduce the possible age- and location-specific contact matrices following the steps detailed in section A.5.

## Age-stratified compartmental model of the physical distancing interventions for COVID-19

We adapt a discrete-time Susceptible-Exposed-Infectious-Recovered (SEIR) compartmental model [11,12] presented in **Figure A**. The model population is closed, and it ignores all demographic changes in the population (i.e., births, deaths, and ageing). The model stratifies the population into (i) disease states: susceptible, exposed (infected but not yet infectious), infectious (i.e., preclinical, clinical, or subclinical), and recovered (or died/removed) states; and (ii) 5-year age groups until age 70 years and a single category aged $\geq$ 75 years, resulting in 16 age bands.

We implement the model stochastically and allow it to select random values of parameters from the set of uncertainty distributions presented in **Table C**, adapted from [11,12].

| 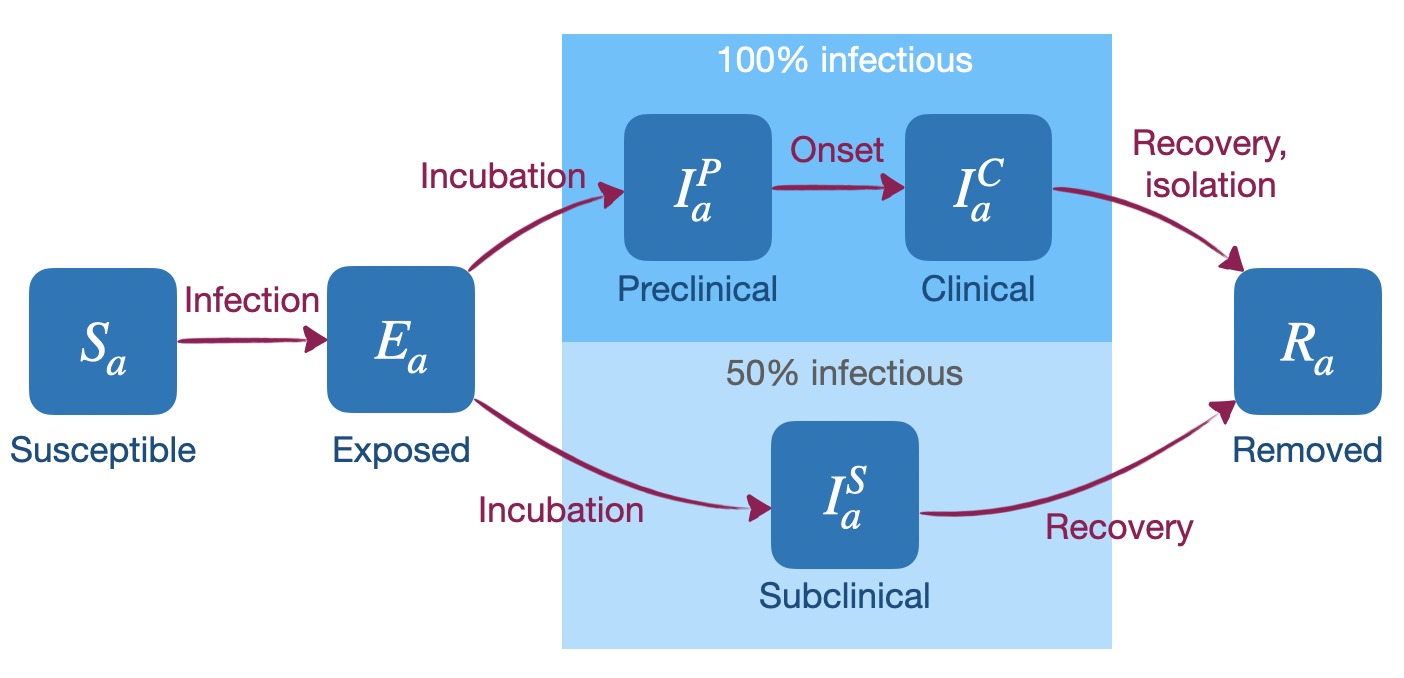 |
| --- |
| **Figure A.** Age-stratified stochastic SEIR model. Adapted from Davies et al. (2020) [11]. |

**Table C.** Parameters of the age-stratified SEIR model.

|  | **Description** | **Values** | **References** |
| --- | --- | --- | --- |
| $\Delta t$ | Time step for simulation | 0.25 days or 6 hours |  |
| $d_{E}$ | Latent period in days | $d_{E} \sim\text{Gamma}\left( 4, 4 \right)$ | [14–16] |
|  | Duration of infectiousness in days |  |  |
| $d_{P}$ | (i) during the preclinical phase | $d_{P} \sim\text{Gamma}\left( 4, 4 \right)$ | [17] |
| $d_{C}$ | (ii) during the clinical phase | $d_{C} \sim\text{Gamma}\left( 4, 4 \right)$ | [14–16] |
| $d_{S}$ | (iii) during the subclinical phase | $d_{S} \sim\text{Gamma}\left( 4, 4 \right)$ | Assumed to be the same as  the total duration of  infectiousness for clinical  cases (i.e., $d_{P}+ d_{C}$) |
| $y_{a}$ | Probability of clinical symptoms on infection for individuals aged $a$ | Age-dependent, as estimated in [18%] | [18] |
| $f$ | Relative infectiousness of subclinical cases | 50% | Assumed |
| $u$ | Probability of transmission per contact with an infectious individual | Refer to text | Derived |
| $N_{a}$ | Number of individuals aged $a$ | Demographic data | [1] |
| $c_{a,\alpha}$ | Number of contacts per day with individuals aged $\alpha$ by an individual age $a$ | Empirical and synthetic contact matrices |  |
| $R_{0}$ | Basic reproduction number | $R_{0} \sim\text{Normal}\left( 2.6, {0.5}^{2} \right)$ | [13] |
|  | Proportion of hospitalised cases requiring critical care | 30% | [20] |
|  | **Duration of disease in days** |  |  |
|  | (i) severe, non-critical disease |  | NHS Digit |
|  | (ii) severe, critical disease | $\sim\text{Gamma}\left( 10, 10 \right)$ | [20] |
|  | **Delay from symptom onset** |  |  |
|  | (i) to becoming a severe case (days) | $\sim\text{Gamma}\left( 7, 7 \right)$ | [19,20] |
|  | (ii) to death (days) | $\sim\text{Gamma}\left( 22, 22 \right)$ | [19,21] |

As described in [11,12], susceptible individuals might acquire the infection when they come in contact with an infectious person and enter the exposed disease state before they become infectious and later either recover or die. The time step of the model is 6 hours. We assume age-specific mixing patterns of individuals alter their likelihood of being exposed to the virus given a certain number of infectious people in the population. The force of infection defined as the rate at which susceptible individuals become exposed t, and is given by for any age group $a$ and time increment $t$ as

$$\lambda_{a,t}=u\times\sum_{\alpha} c_{a,\alpha}\times\frac{I_{\alpha}^{P}+I_{\alpha}^{C}+f\times I_{\alpha}^{S}}{N_{\alpha}}$$

where $u$ is the probability of transmission per contact with an infectious person, $c_{a,\alpha}$ denotes the number of contacts per day with individuals aged $\alpha$ by an individual in age group $a$ per time increment (drawn from the contact matrix), and $\frac{\left( I_{\alpha}^{P}+I_{\alpha}^{C}+fI_{\alpha}^{S} \right)}{N_{\alpha}}$ is the probability that any age $\alpha$ individual contacted is infectious, with $f$ denoting the relative infectiousness of subclinical cases, compared to clinical cases. The basic reproduction number $R_{0}$ is defined as the average number of secondary infections generated by an infectious individual in a fully susceptible population, we calculated it as the absolute value of the dominant eigenvalue of the next generation matrix, which was derived by linearising the system at epidemic equilibrium [13]. For any stochastic run, $u$ is derived from the ratio of this eigenvalue and the $R_{0}$ value selected for that run. We considered six contact matrices when modelling the interventions to the COVID-19 pandemic: the empirically-constructed contact matrices at the study-year and adjusted for the 2020 population, the 2017 synthetic matrices, and the updated synthetic matrices at the national, rural, or urban settings. As the synthetic matrices only go up until age 80, we implemented these in the model by assuming that the modelled contacts made by and with those aged 75–79 years old were representative for all in the 75+ years-old in the model.

The duration (in days) an individual spends in states $E$, $I^{P}$, $I^{C}$, or $I^{S}$ is drawn from distributions $d_{E}$, $d_{P}$, $d_{C}$ and $d_{S}$, respectively (**Table C**) [14–17]. After the latency period, infected individuals are divided into clinical (symptomatic) and sub-clinical (asymptomatic) states with probability $y_{a}$ and $1-y_{a}$, respectively [18]. Clinical individuals will first experience a preclinical phase where they are still infectious (denoted as $I^{P}$ in **Figure A**), followed by a clinical and infectious phase (denoted as $I^{C}$ in **Figure A**). We assume that subclinical individuals, represented as $I^{S}$ in **Figure A**, are half as infectious as clinical cases. Clinical and subclinical individuals have the same duration of infectiousness, and we assume that their clinical severity does not affect their infectiousness. These individuals will later recover or die, and we assume that all individuals who have recovered (or have left the infectious phase) are immune until the end of the simulations. We account for delays in the transitions to different clinical disease states (such as symptom onset to severe case, critical or not; and from onset of severe symptoms to recovery or death) [19–21]. These delays are drawn from distributions reported in the literature; however, they do not change the force of infection or transmissibility.

## Hierarchical model of POLYMOD contact data

To address the multi-level structure of the data, with repeat measurements of contacts made in different settings by the same individual, we employed Bayesian hierarchical modelling to estimate the proclivity of age-specific and location specific contact patterns in each of the POLYMOD countries, as this provided a flexible framework to estimate both individual-level and population-level parameters.

The number of contacts made by individual $i$ at a particular location $L$ with someone in age group $\alpha$, $X_{i,\alpha}^{L}$, is modelled to be Poisson with mean $\mu_{i,\alpha}^{L}$,

$$X_{i,\alpha}^{L}\sim\mathrm{Po}(\mu_{i,\alpha}^{L})$$

where

- the ages of individual $i$, $a_{i}$, and his contact, $\alpha$, are categorised into 5-year age intervals, $\{1,2,\ldots,16\}$; and
- $L$ indicates the location of the contact namely home (L=H), work (W), school (S) and other (O).

The Poisson mean parameter has the general form $\mu_{i,\alpha}^{L}=\sigma_{i}\lambda_{a_{i},\alpha}^{L}$ and varies across locations. In this model, $\sigma_{i}$ is a random effect belonging to individual $i$ which characterises differences in social activity levels across locations and allows for greater than Poisson variability in the number of contacts.

The mean was $\mu_{i,\alpha}^{H}=\sigma_{i}\lambda_{a_{i},\alpha}^{H}(\nu_{i,\alpha}+\delta_{H})$ for home contacts, $\mu_{i,\alpha}^{W}=\sigma_{i}\lambda_{a_{i},\alpha}^{W}(w_{i})$ for work contacts, $\mu_{i,\alpha}^{S}=\sigma_{i}\lambda_{a_{i},\alpha}^{S}(s_{i})$ for school contacts and $\mu_{i,\alpha}^{O}=\sigma_{i}\lambda_{a_{i},\alpha}^{O}$ for contacts at all other locations. The number of cohabitants of $i$ of age $\alpha$, $\nu_{i,\alpha}$, represents household age structure, while $w_{i}$ and $s_{i}$ indicate if $i$ went to work or school on the day of the survey. Contact with visitors at home is allowed via background contact parameter, $\delta_{H}$. The parameter $\lambda_{a_{i},\alpha}^{L}$ quantifies typical contact rates between individuals of age groups $a_{i}$ and $\alpha$ at location $L$ and is the key estimand in the model. The parameter $\lambda_{a_{i},\alpha}^{W}$ represents the contact rates for people who go to work reporting work contacts with other people (regardless of whether the other people are going to work or not). Similarly, $\lambda_{a_{i},\alpha}^{S}$ represents the contact rates for people who go to school reporting school contacts with other people.

**Prior distribution of parameters:** The $\lambda_{a_{i},\alpha}^{L}$ parameter was given a hierarchical prior to impose smoothness between successive age groups, i.e.:

$$\log\lambda_{a_{i},\alpha}^{L}=\sum_{\mathcal{A},A:\mathcal{A},A\in\mathcal{N}_{a_{i},\alpha}} \frac{\epsilon_{\mathcal{A},A}^{L}}{|\mathcal{N}_{a_{i},\alpha}|}$$

where $\epsilon_{\mathcal{A},A}^{L}$ is a hyperparameter of $\lambda_{a_{i},\alpha}^{L}$ and $\mathcal{N}_{a_{i},\alpha}$ is the set of (up to) four adjacent age groups together with $(a_{i},\alpha)$ itself (presented in **Figure B**). The adjacent age groups (light blue regions) together with $(a_{i},\alpha)$ (dark blue regions i.e., A, B, and C) are elements of $\mathcal{N}_{a_{i},\alpha}$.

| 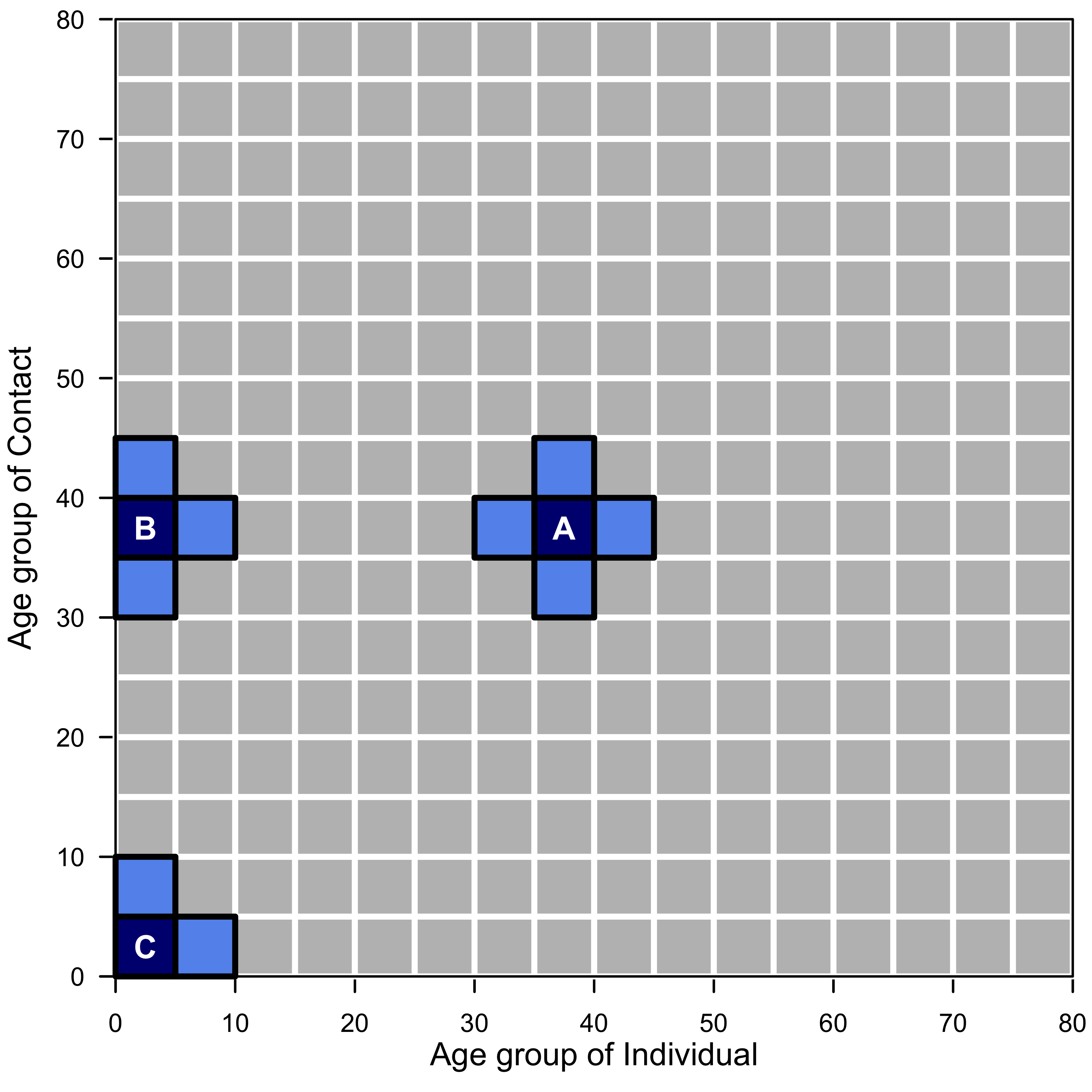 |
| --- |
| **Figure B.** Set of up to four adjacent age groups. |

Contact surveys conducted in several countries [3] indicate strong assortativity of social contacts with age. Hence, to allow the number of contacts to be comparable for individuals of similar ages, the hierarchical prior was designed to impose smoothness between successive age groups (right panel of **Figure C**). This smoothness on the prior distribution of the parameter $\lambda_{a_{i},\alpha}^{L}$ to allow the number of contacts to be comparable for individuals of similar ages (**Figure C**). The hyperparameter $\epsilon_{\mathcal{A},A}^{L}$ has a non-informative prior distribution of $\mathrm{Normal}\sim(0,{100}^{2})$ as represented on the left of **Figure C**.

| 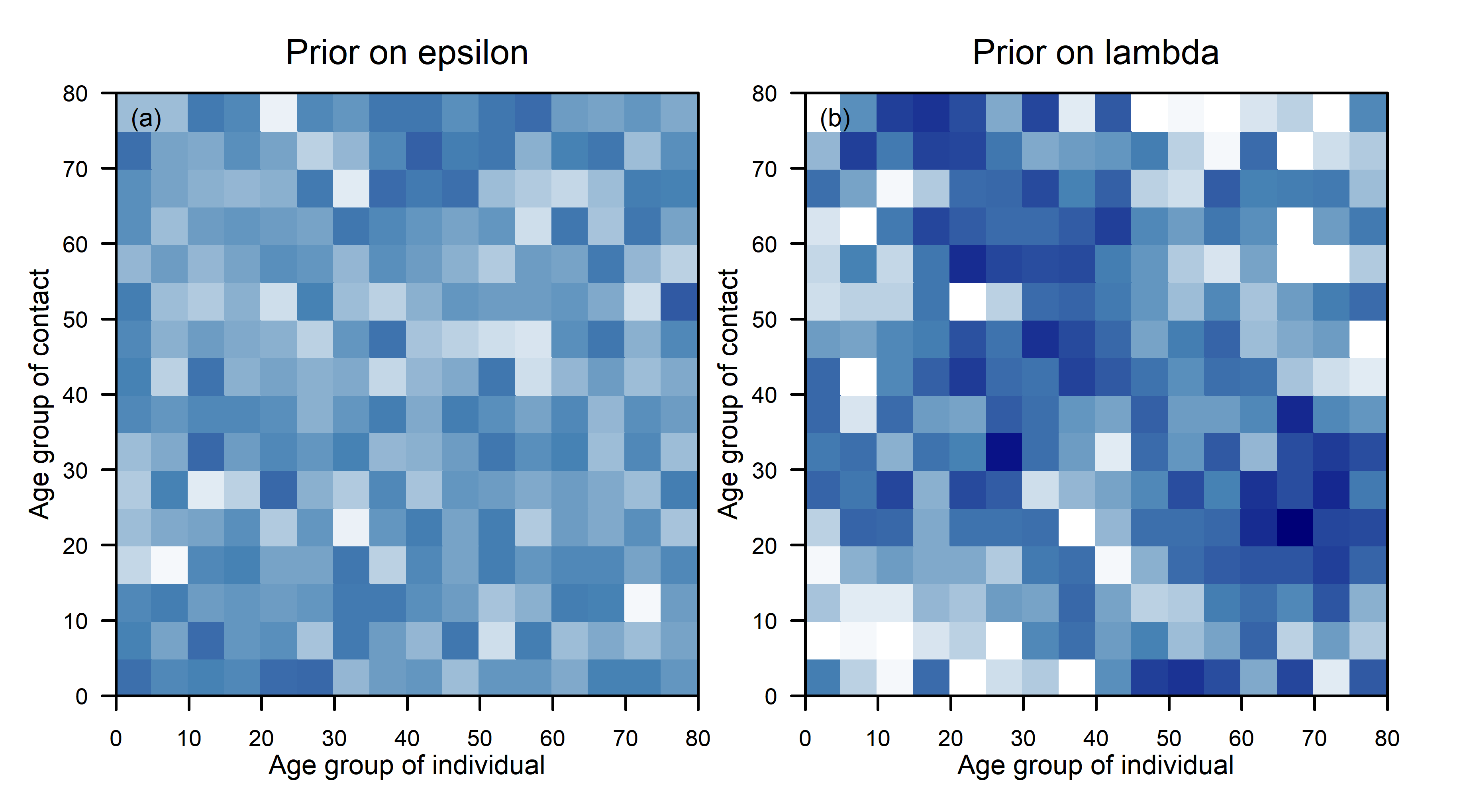 |
| --- |
| **Figure C.** Smoothness between successive age groups on the prior distribution. |

Throughout, we used non-informative prior distributions for all parameters and hyperparameters in the model, presented in Prem et al. (2017) [5], unless otherwise noted.

# References

1. United Nations Department of Economic and Social Affairs Population Division. World Population Prospects. 2019. Available: <https://population.un.org/wpp/>

2. United Nations Department of Economic and Social Affairs Population Division. Urban and Rural Population by Age and Sex, 1980-2015. Available: <https://www.un.org/en/development/desa/population/publications/dataset/urban/urbanAndRuralPopulationByAgeAndSex.asp>

3. Mossong J, Hens N, Jit M, Beutels P, Auranen K, Mikolajczyk R, et al. Social Contacts and Mixing Patterns Relevant to the Spread of Infectious Diseases. Riley S, editor. PLoS Medicine. 2008;5: e74. doi:[10.1371/journal.pmed.0050074](https://doi.org/10.1371/journal.pmed.0050074)

4. Demographic and Health Surveys. The DHS Program - Demographic and Health Survey (DHS). Available: <https://dhsprogram.com/What-We-Do/Survey-Types/DHS.cfm>

5. Prem K, Cook AR, Jit M. Projecting social contact matrices in 152 countries using contact surveys and demographic data. PLoS Computational Biology. 2017;13: e1005697. doi:[10.1371/journal.pcbi.1005697](https://doi.org/10.1371/journal.pcbi.1005697)

6. UNESCO Institute for Statistics. UIS Statistics. Available: <http://data.uis.unesco.org/>

7. International Labour Organization. Labour force by sex and age.

8. OECD. Teachers by age (indicator). 2020. doi:[10.1787/93af1f9d-en](https://doi.org/10.1787/93af1f9d-en)

9. International Labour Organization. Labour force participation rate by sex, age and rural / urban areas.

10. OECD. Differences in rural and urban schools’; student-teacher ratio and class size, 2015. OECD Publishing; 2018.

11. Davies NG, Kucharski AJ, Eggo RM, Gimma A, Edmunds WJ, Jombart T, et al. Effects of non-pharmaceutical interventions on COVID-19 cases, deaths, and demand for hospital services in the UK: a modelling study. The Lancet Public Health. 2020;0. doi:[10.1016/s2468-2667(20)30133-x](https://doi.org/10.1016/s2468-2667(20)30133-x)

12. van Zandvoort K, Jarvis CI, Pearson C, Davies NG, CMMID COVID-19 working Group, Russell TW, et al. Response strategies for COVID-19 epidemics in African settings: a mathematical modelling study. BMC Medicine. 2020;18: 324 doi:[10.1186/s12916-020-01789-2](https://doi.org/10.1186/s12916-020-01789-2)

13. Jarvis CI, Van Zandvoort K, Gimma A, Prem K, Klepac P, Rubin GJ, et al. Quantifying the impact of physical distance measures on the transmission of COVID-19 in the UK. BMC Medicine. 2020;18: 124. doi:[10.1186/s12916-020-01597-8](https://doi.org/10.1186/s12916-020-01597-8)

14. Li Q, Guan X, Wu P, Wang X, Zhou L, Tong Y, et al. Early Transmission Dynamics in Wuhan, China, of Novel Coronavirus–Infected Pneumonia. New England Journal of Medicine. 2020. doi:[10.1056/nejmoa2001316](https://doi.org/10.1056/nejmoa2001316)

15. Bi Q, Wu Y, Mei S, Ye C, Zou X, Zhang Z, et al. Epidemiology and Transmission of COVID-19 in Shenzhen China: Analysis of 391 cases and 1,286 of their close contacts. medRxiv. 2020; 2020.03.03.20028423. doi:[10.1101/2020.03.03.20028423](https://doi.org/10.1101/2020.03.03.20028423)

16. Nishiura H, Linton NM, Akhmetzhanov AR. Serial interval of novel coronavirus (COVID-19) infections. International Journal of Infectious Diseases. 2020;93: 284–286. doi:[10.1016/j.ijid.2020.02.060](https://doi.org/10.1016/j.ijid.2020.02.060)

17. Liu Y, Funk S, Flasche S. The contribution of pre-symptomatic infection to the transmission dynamics of COVID-2019. Wellcome Open Research. 2020;5: 58. doi:[10.12688/wellcomeopenres.15788.1](https://doi.org/10.12688/wellcomeopenres.15788.1)

18. Davies NG, Klepac P, Liu Y, Prem K, Jit M, Eggo RM. Age-dependent effects in the transmission and control of COVID-19 epidemics. Nature Medicine. 2020; 1–7. doi:[10.1038/s41591-020-0962-9](https://doi.org/10.1038/s41591-020-0962-9)

19. Linton NM, Kobayashi T, Yang Y, Hayashi K, Akhmetzhanov AR, Jung S-m, et al. Incubation Period and Other Epidemiological Characteristics of 2019 Novel Coronavirus Infections with Right Truncation: A Statistical Analysis of Publicly Available Case Data. Journal of Clinical Medicine. 2020;9: 538. doi:[10.3390/jcm9020538](https://doi.org/10.3390/jcm9020538)

20. Cao B, Wang Y, Wen D, Liu W, Wang J, Fan G, et al. A Trial of Lopinavir–Ritonavir in Adults Hospitalized with Severe Covid-19. New England Journal of Medicine. 2020;382: 1787–1799. doi:[10.1056/NEJMoa2001282](https://doi.org/10.1056/NEJMoa2001282)

21. The Novel Coronavirus Pneumonia Emergency Response Epidemiology Team. The epidemiological characteristics of an outbreak of 2019 novel coronavirus disease (COVID-19)—China. 2020. China CDC Wkly. 2020;2: 113–122.

1. Correspondence to [mark.jit@lshtm.ac.uk](mailto:mark.jit@lshtm.ac.uk). [↑](#footnote-ref-1)
