# Supplementary material for: Projecting contact matrices in 177 geographical regions: An update and comparison with empirical data for the COVID-19 era
Source: PLoS Comput Biol. 2021 Jul 26;17(7):e1009098. doi: 10.1371/journal.pcbi.1009098 (PMC8354454; doi:10.1371/journal.pcbi.1009098)
Supplement: S2 Text — (DOCX) [file pcbi.1009098.s002.docx]

Projecting contact matrices in 177 geographical regions: an update and comparison with empirical data for the COVID-19 era

Supplementary Material

Kiesha Prem, Kevin van Zandvoort, Petra Klepac, Rosalind M Eggo, Nicholas G Davies,

Centre for the Mathematical Modelling of Infectious Diseases COVID-19 Working Group,

Alex R Cook, Mark Jit^[[1]](#footnote-1)^

Contents

[B. Results 1](#_Toc144640467)

[B.1. Household age matrix (HAM) validation for POLYMOD and DHS countries 1](#_Toc144640468)

[B.2. Household age matrix (HAM) urban and rural comparison 19](#_Toc144640469)

# Results

## Household age matrix (HAM) validation for POLYMOD and DHS countries

The empirical household age structures (i.e., from the data) and the modelled household age structures (i.e., the leave-one-out validation, assuming no available household data) for each POLYMOD and DHS countries are represented in the first and second panels respectively of the figures in this section. In the third panel, a scatter plot of the entries in the observed (x-axis) and modelled (y-axis) HAM. Using the methods described in section A.2.2, we reconstructed the empirical household age structures for the POLYMOD and DHS countries with high fidelity (median correlation between the observed and modelled HAM 0.96, with an interquartile range 0.94–0.97).


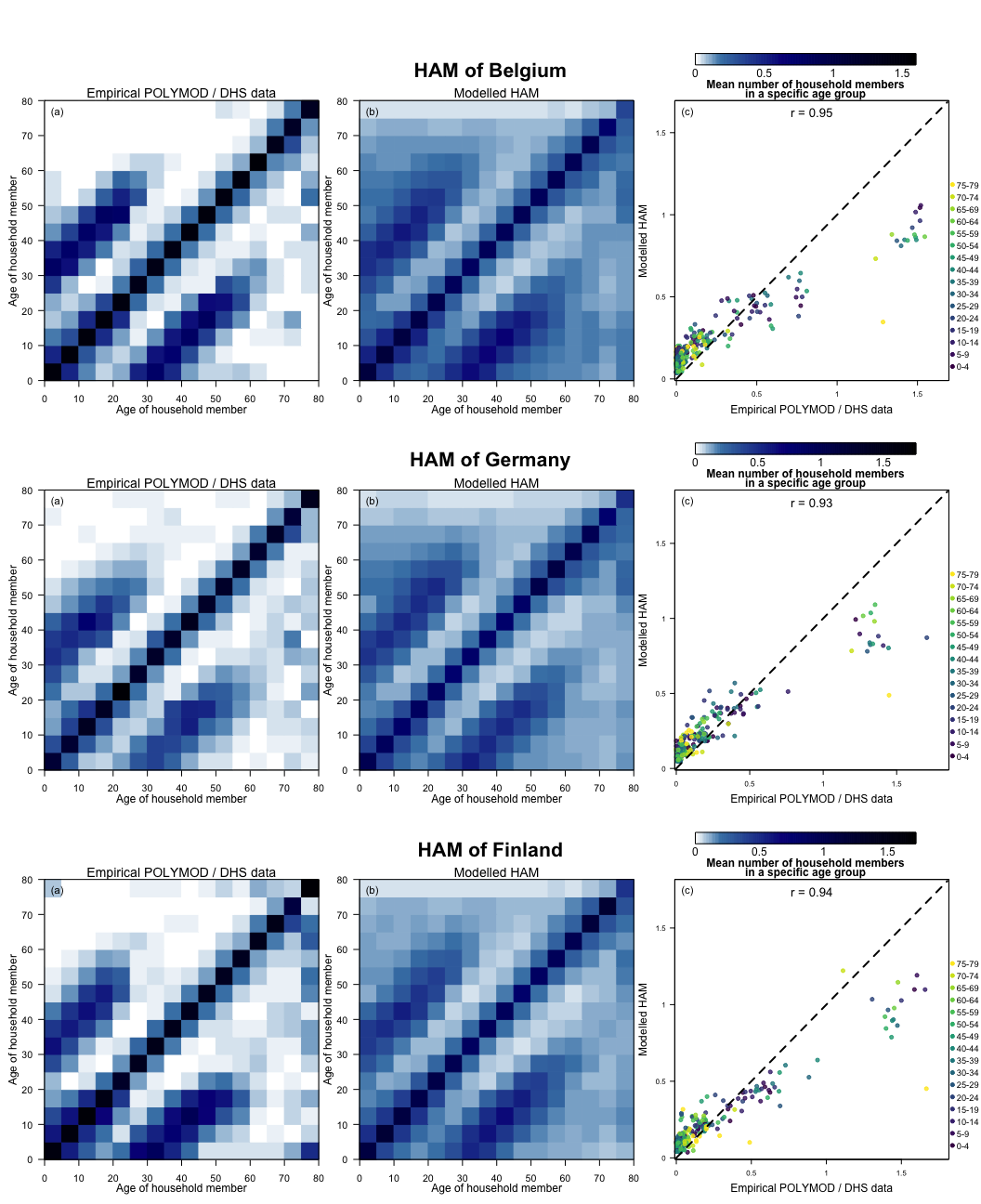

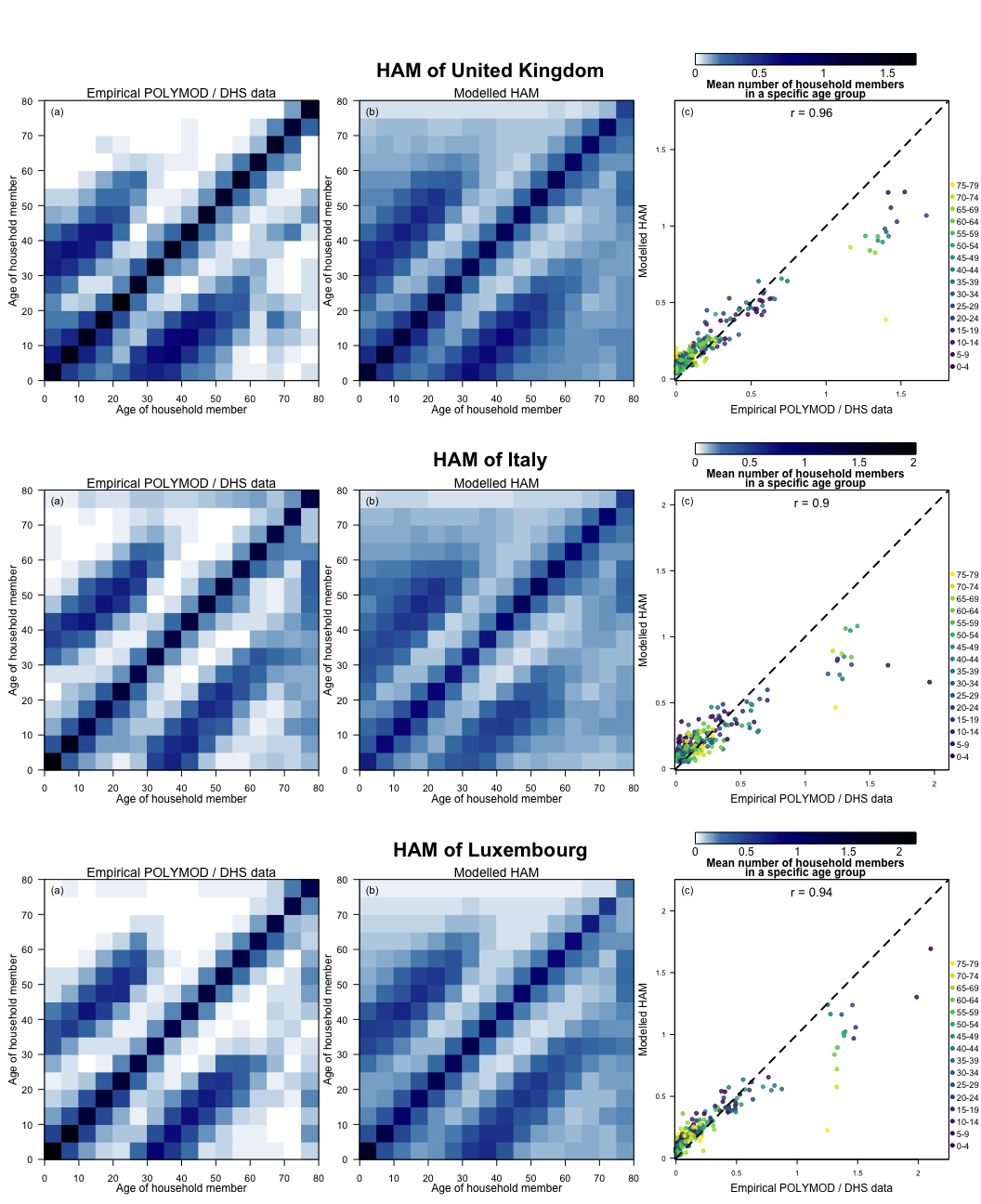

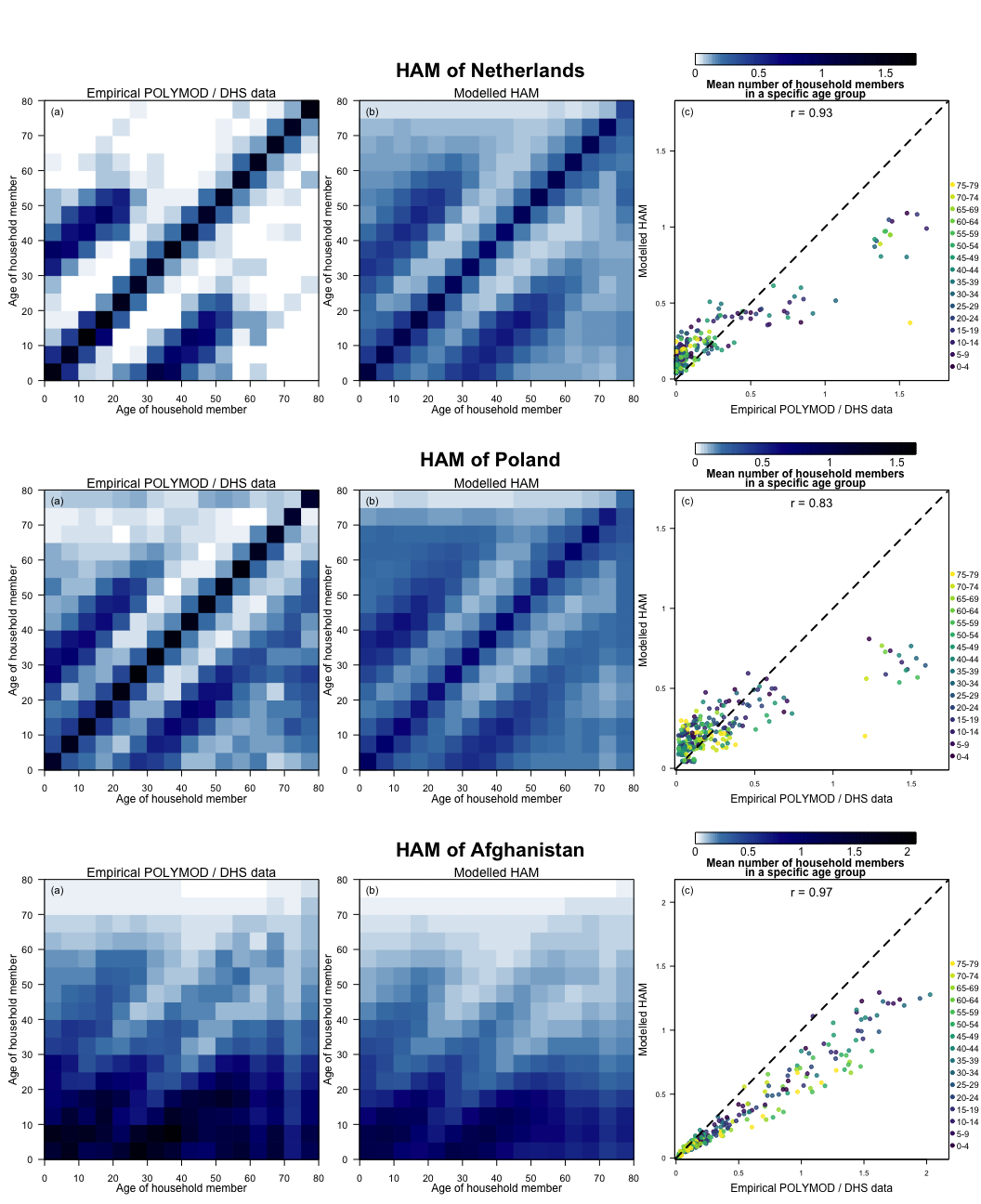

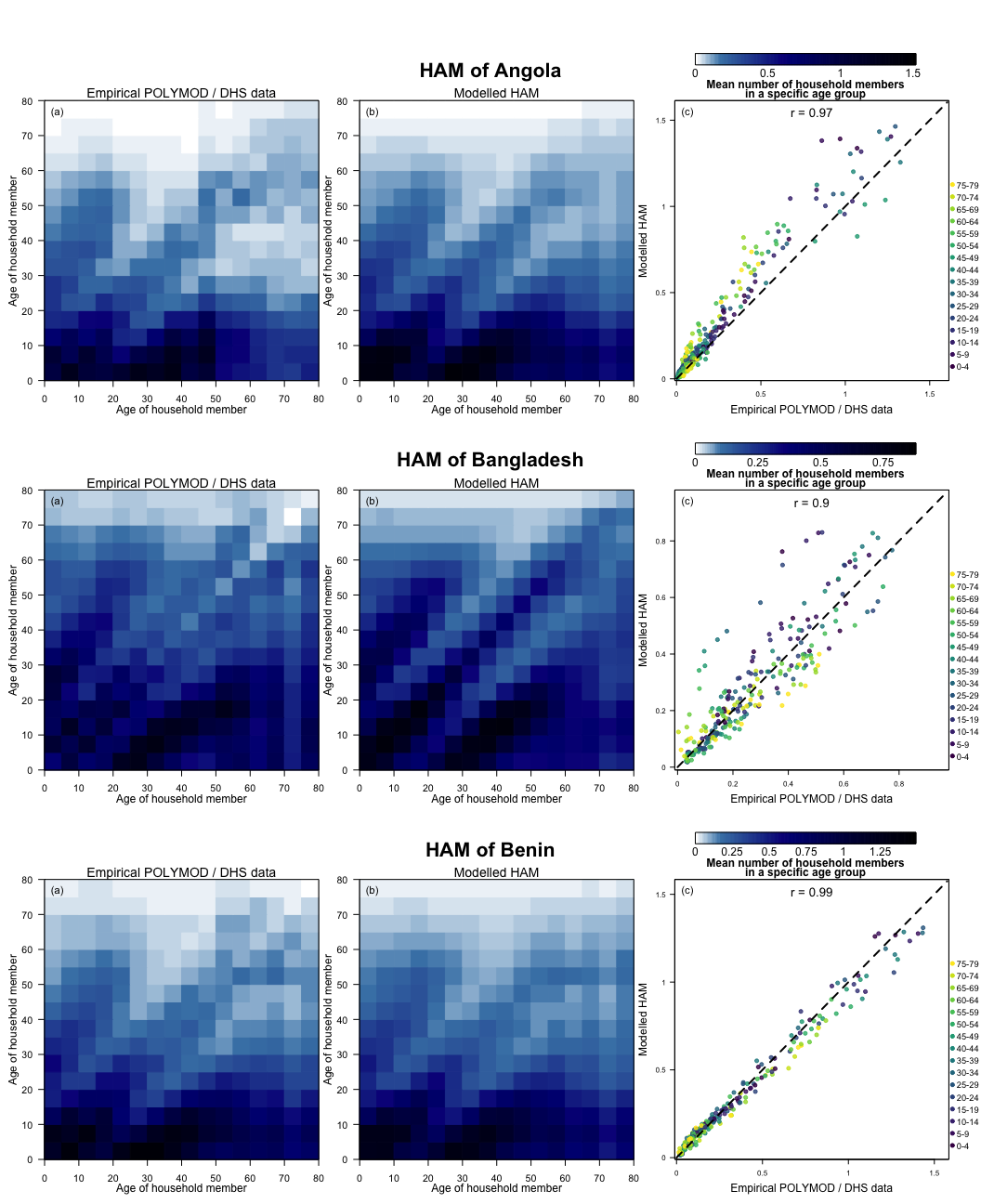

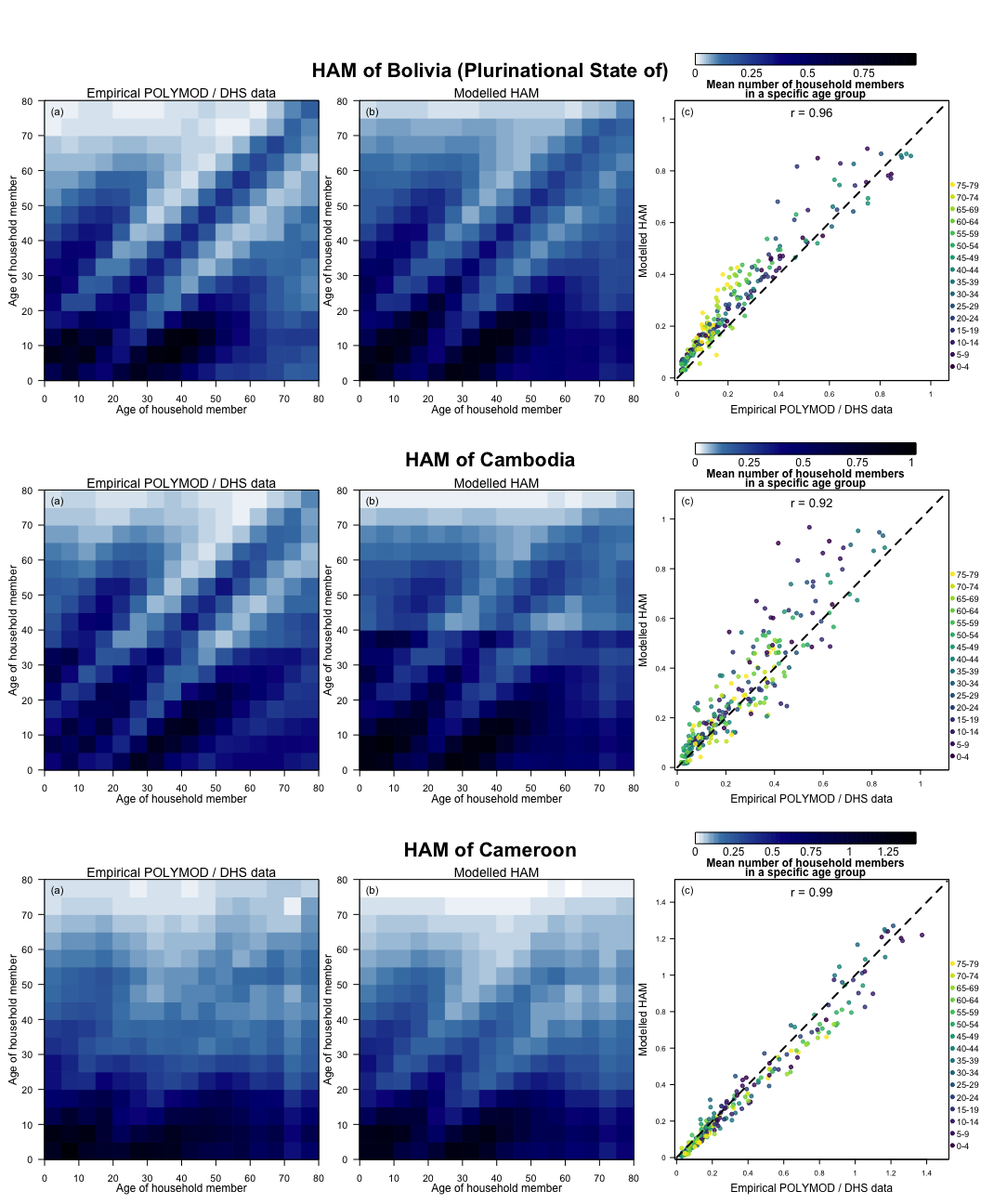

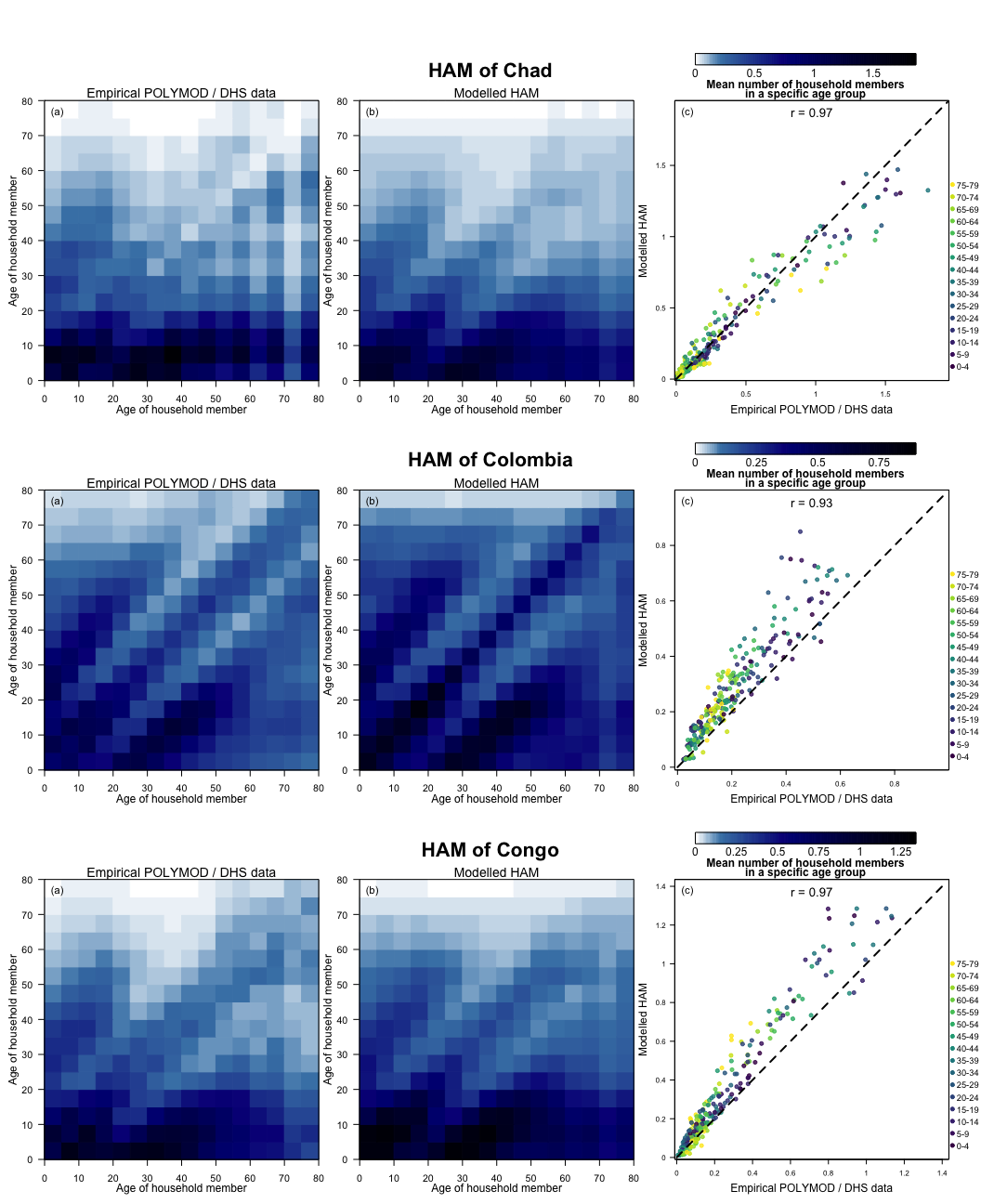

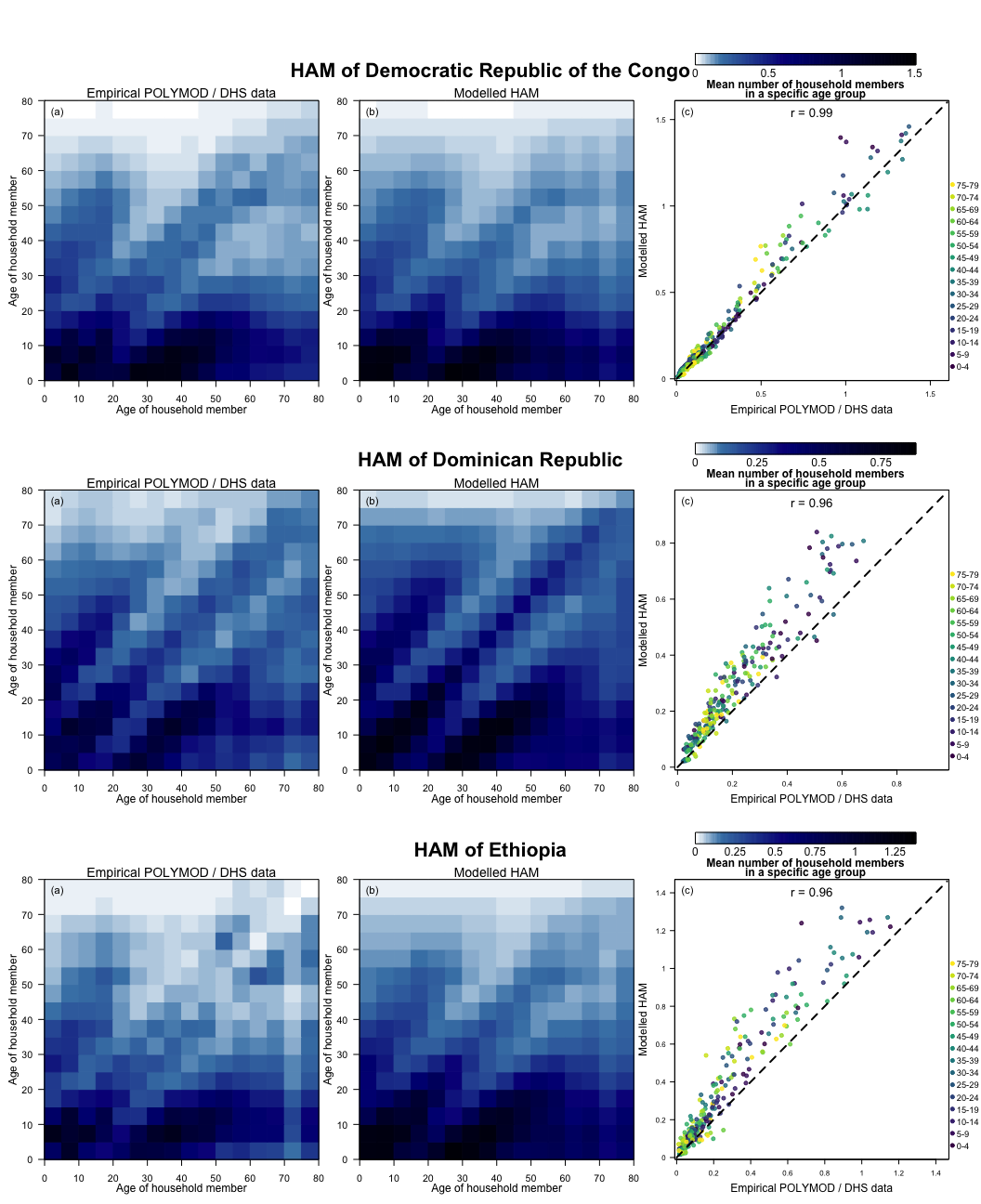

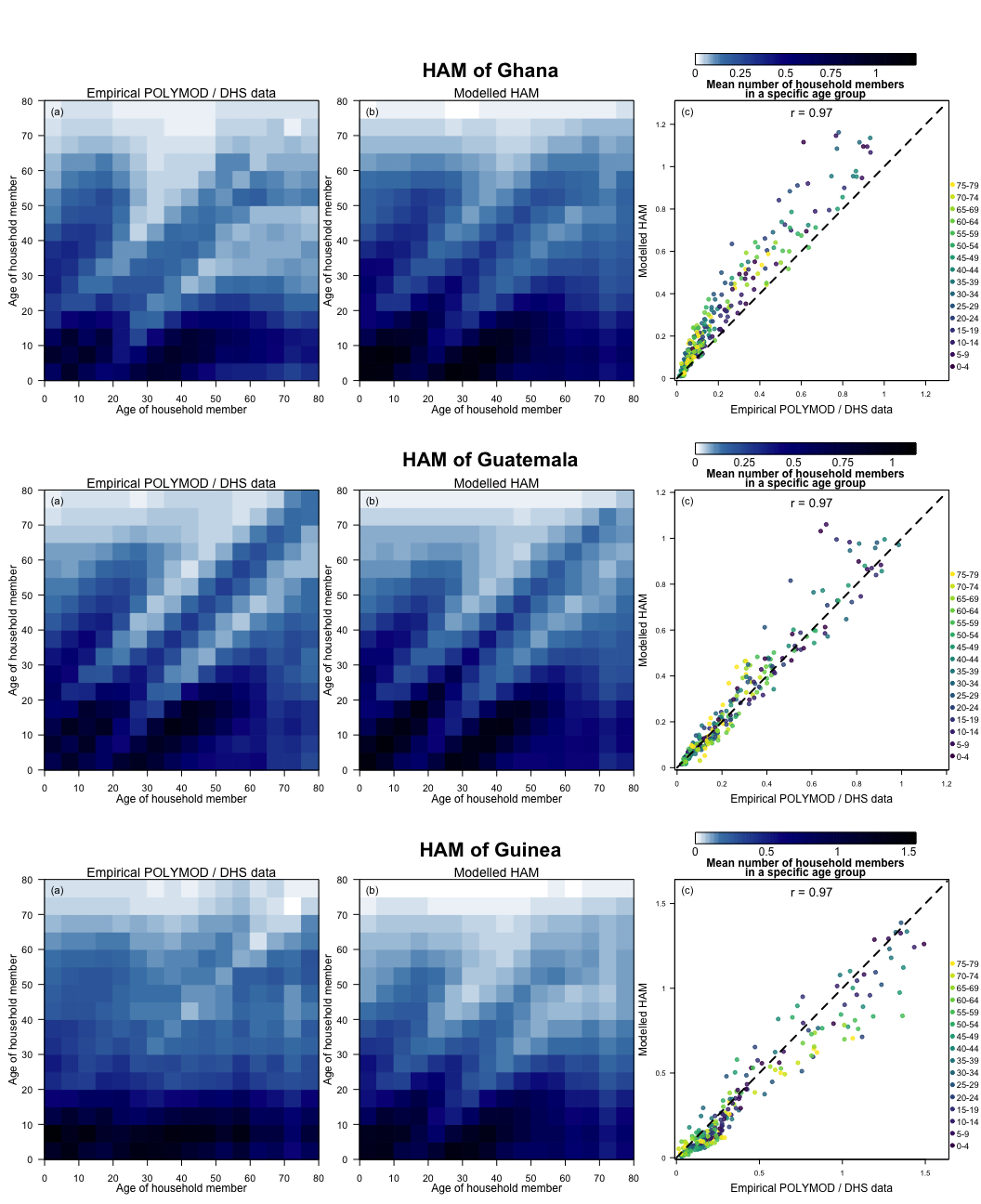

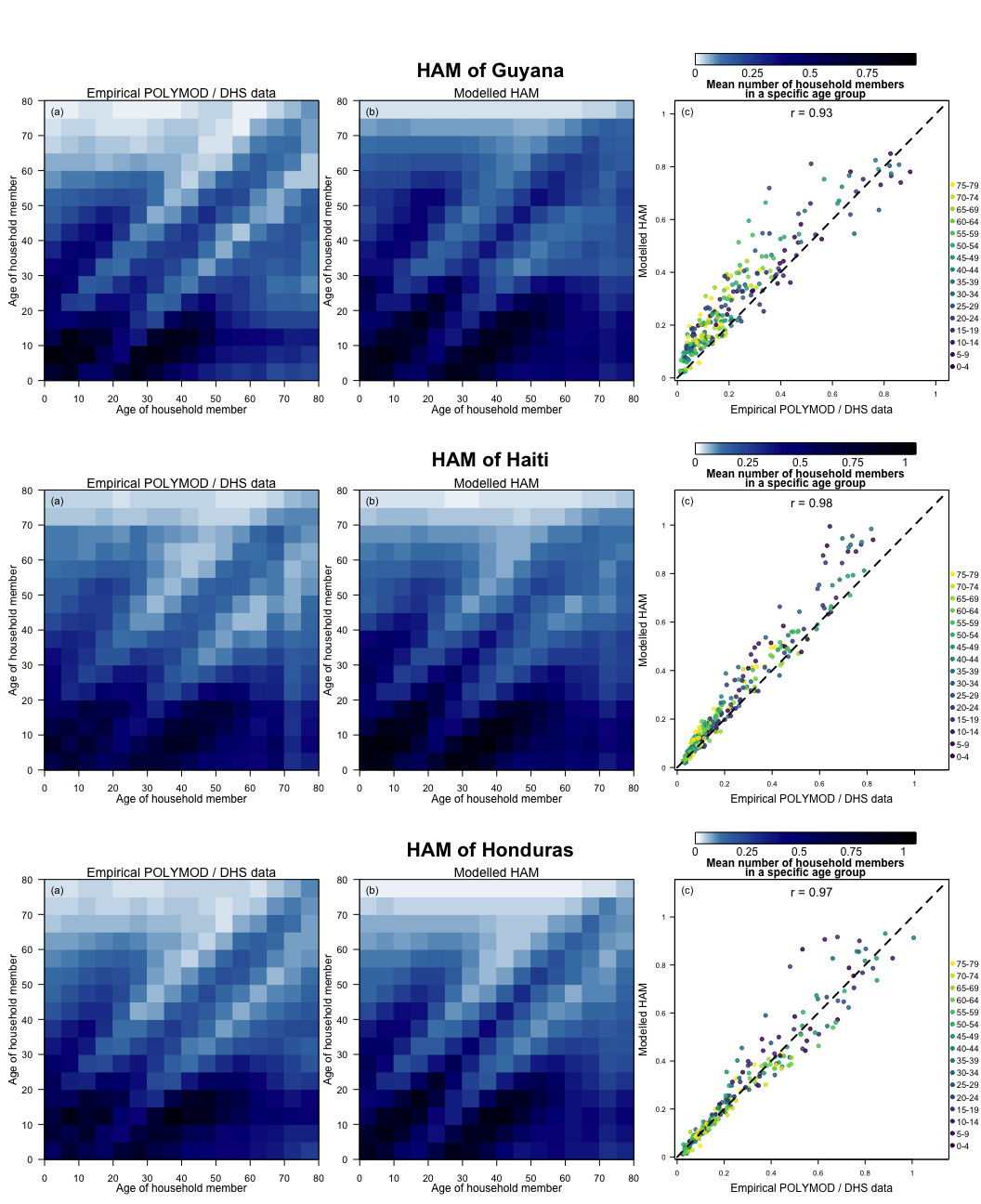

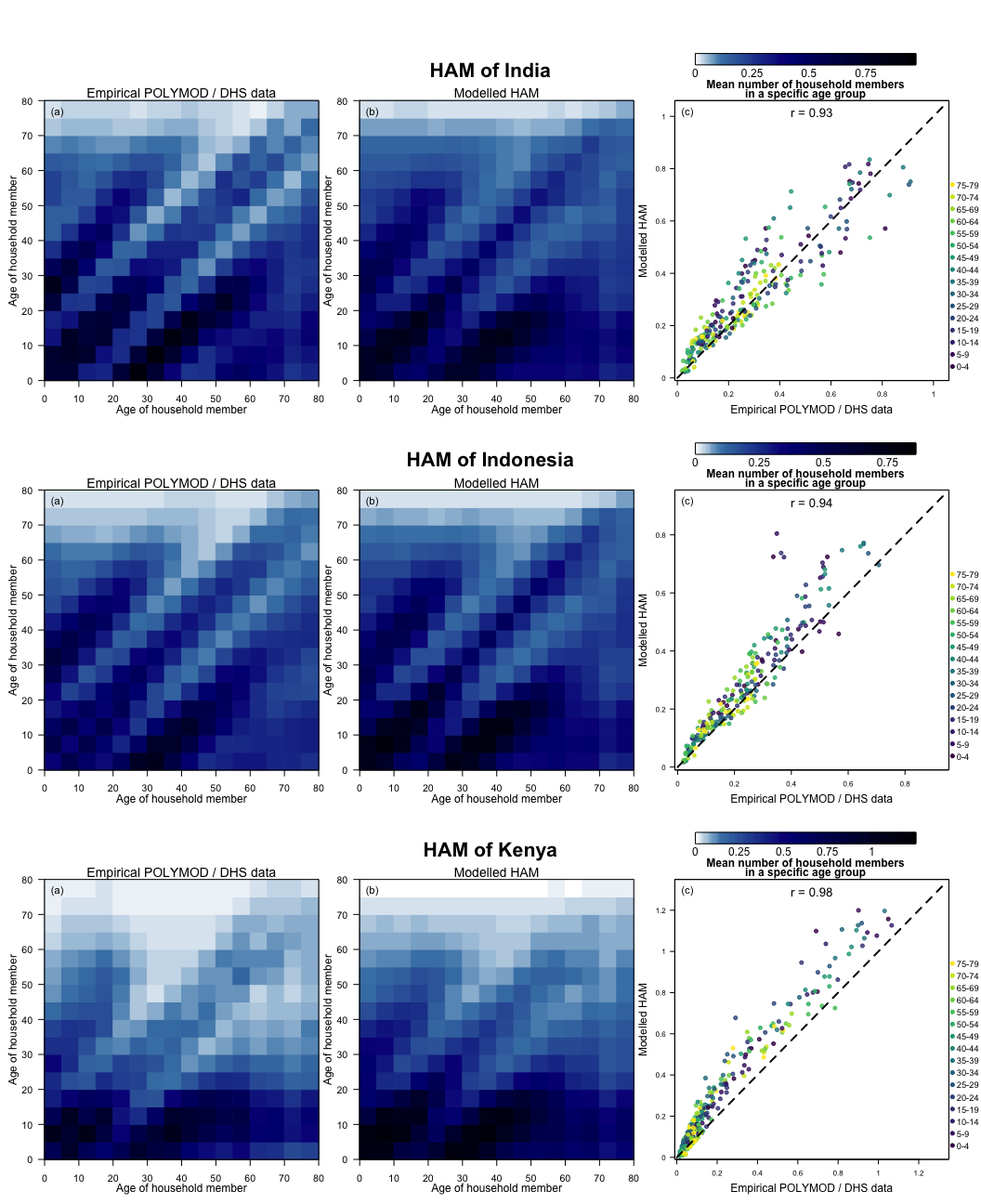

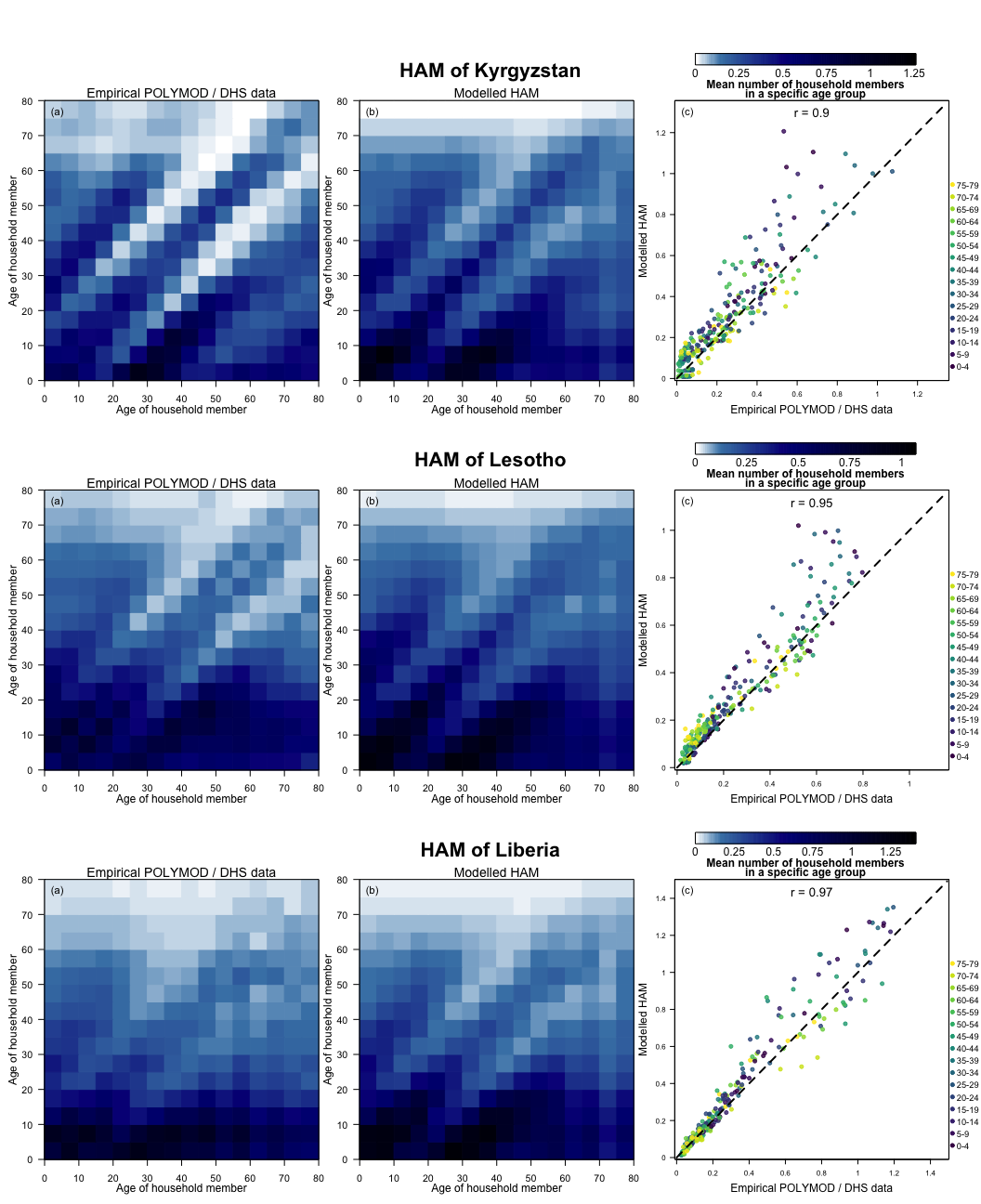

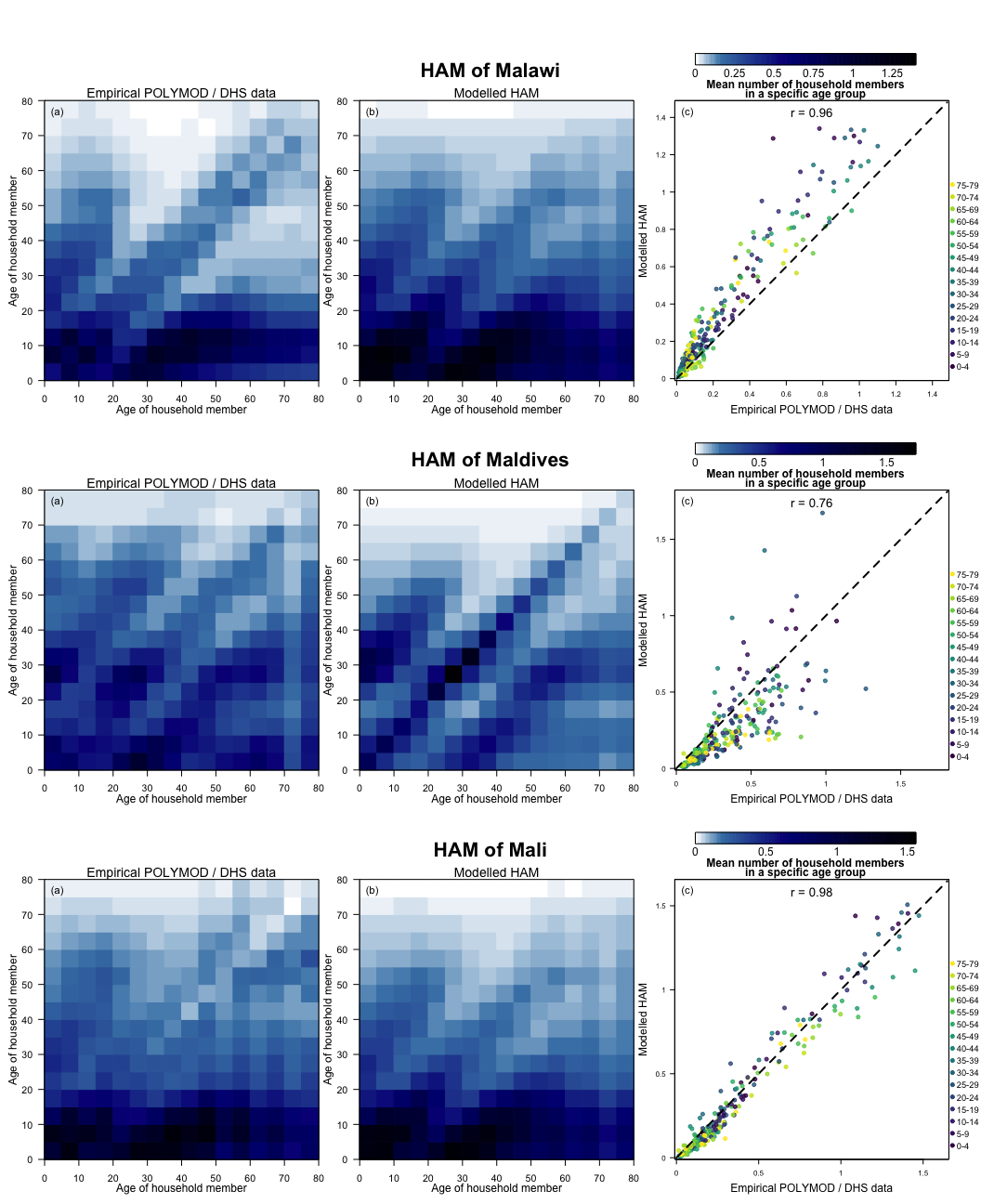

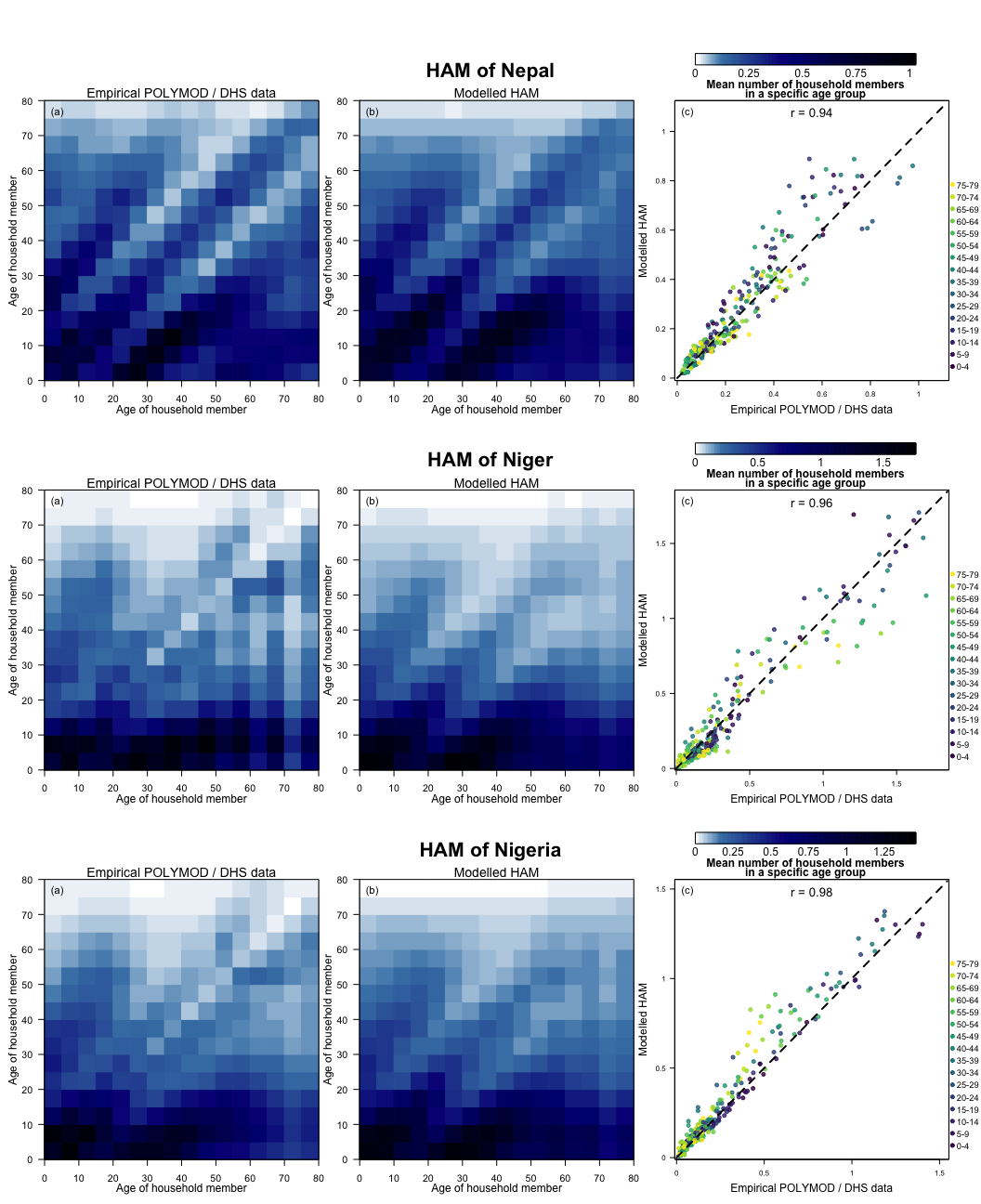

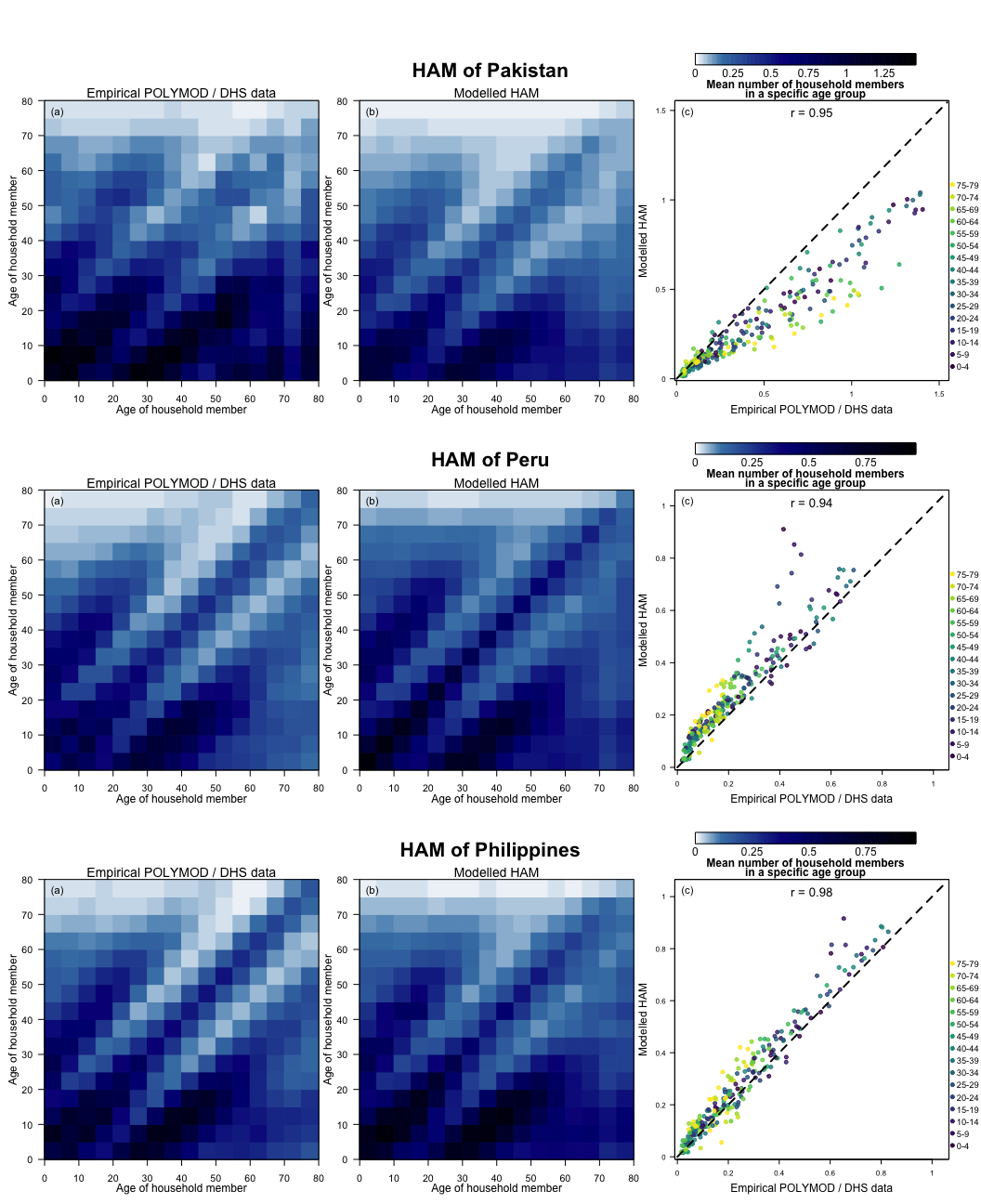

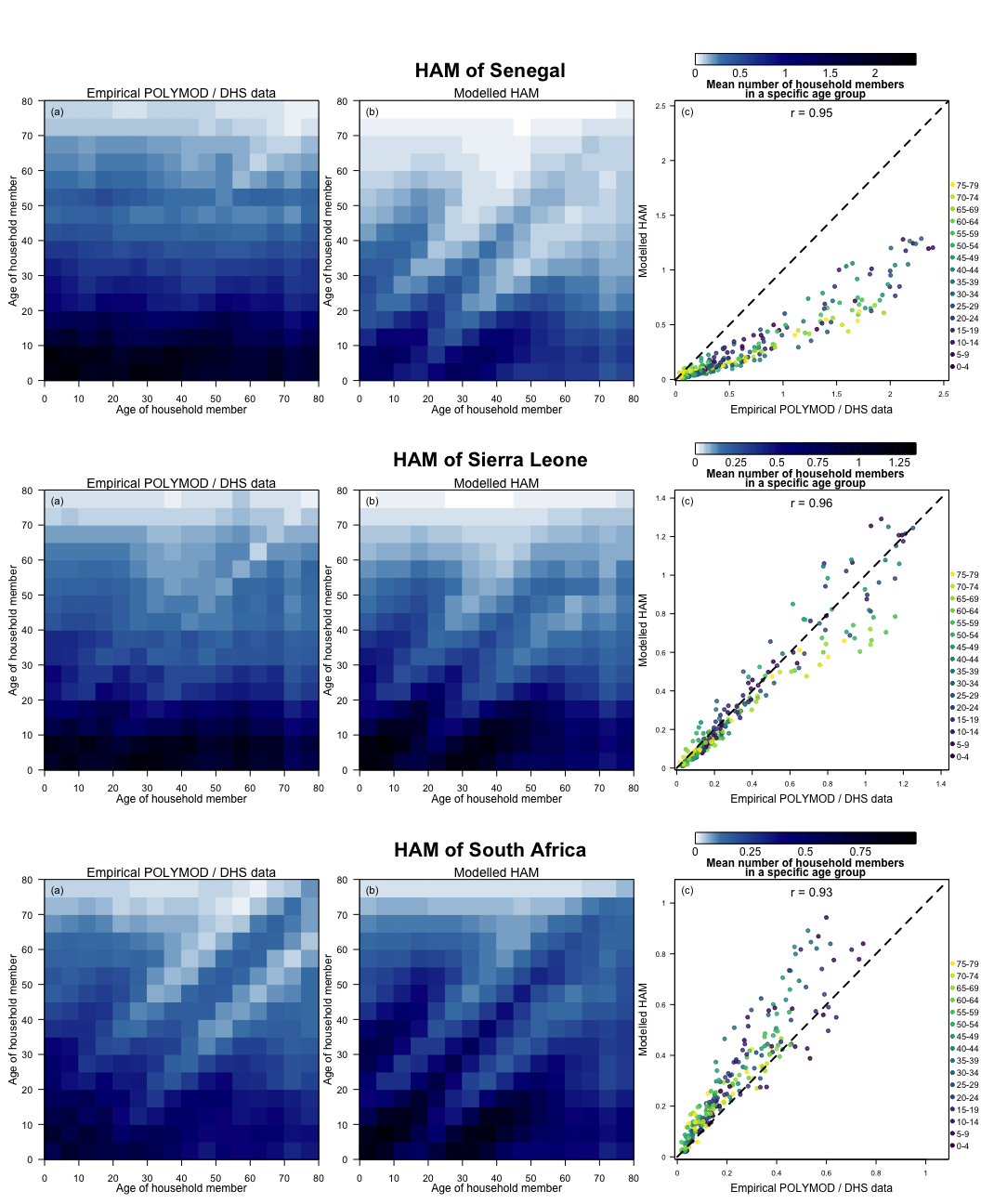

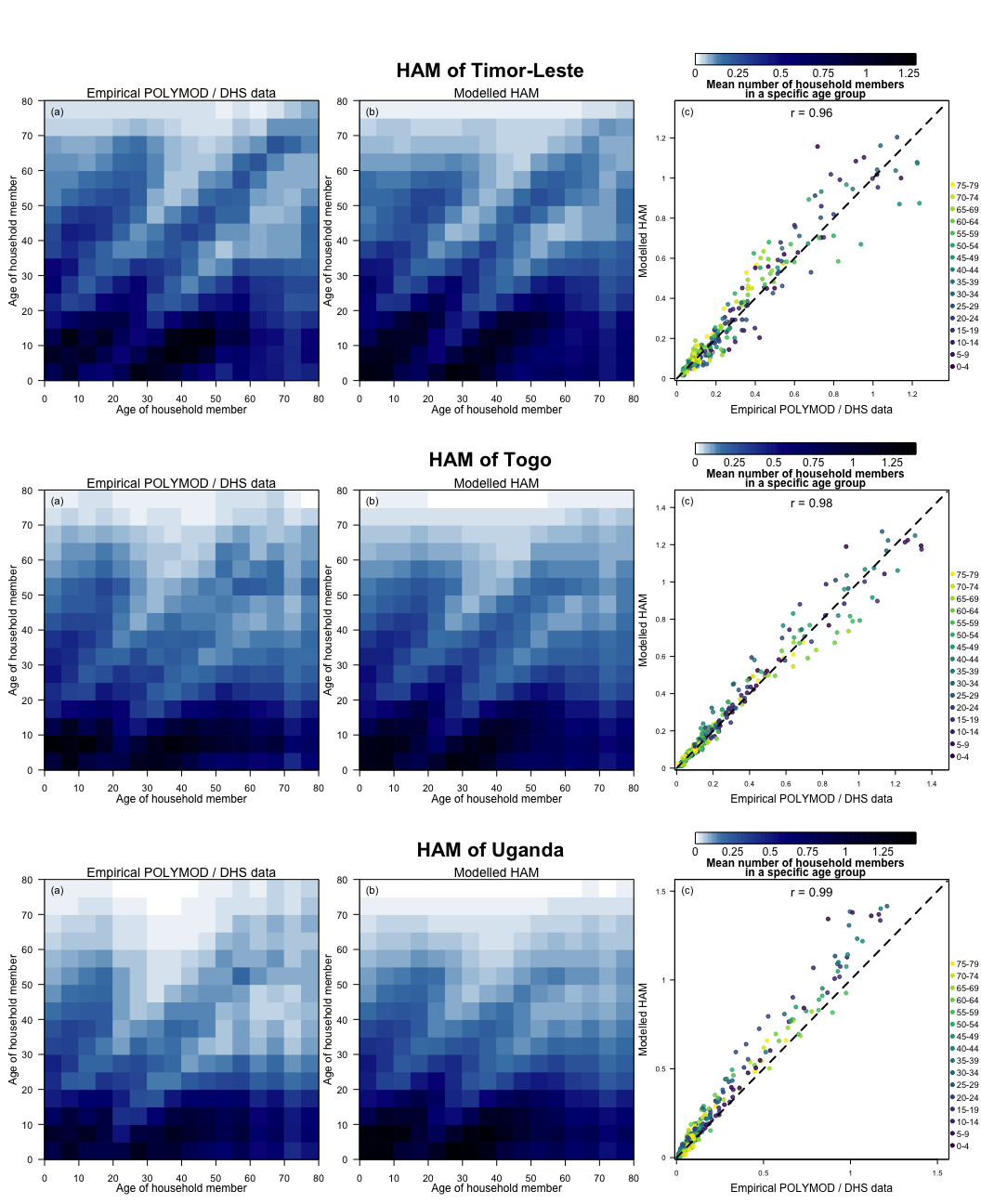


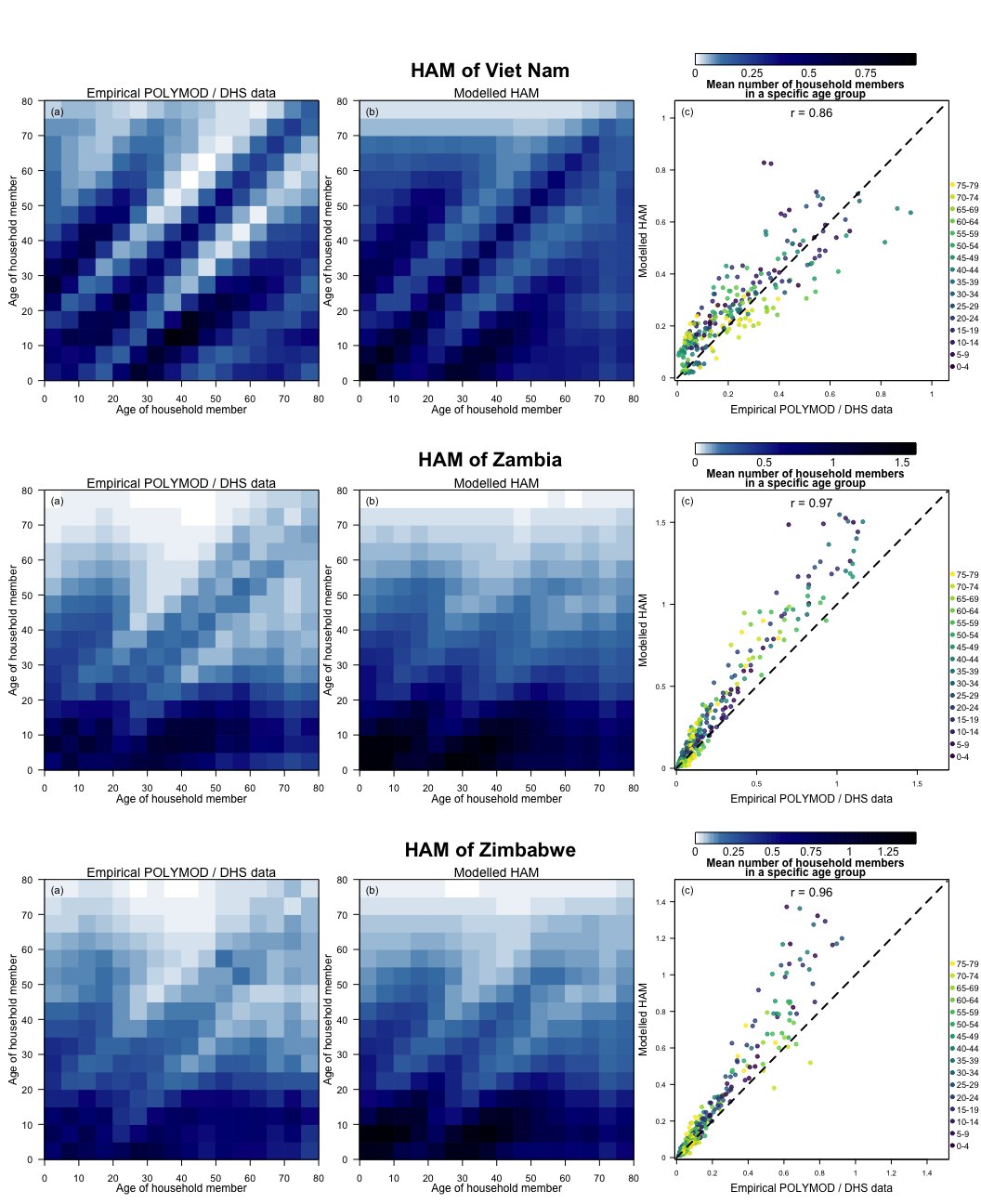


## Household age matrix (HAM) urban and rural comparison

Using rural-urban stratified population and household data, we present the population age compositions and household age matrices for rural and urban areas by the rural and urban subregions for countries with recent DHS household surveys.

| 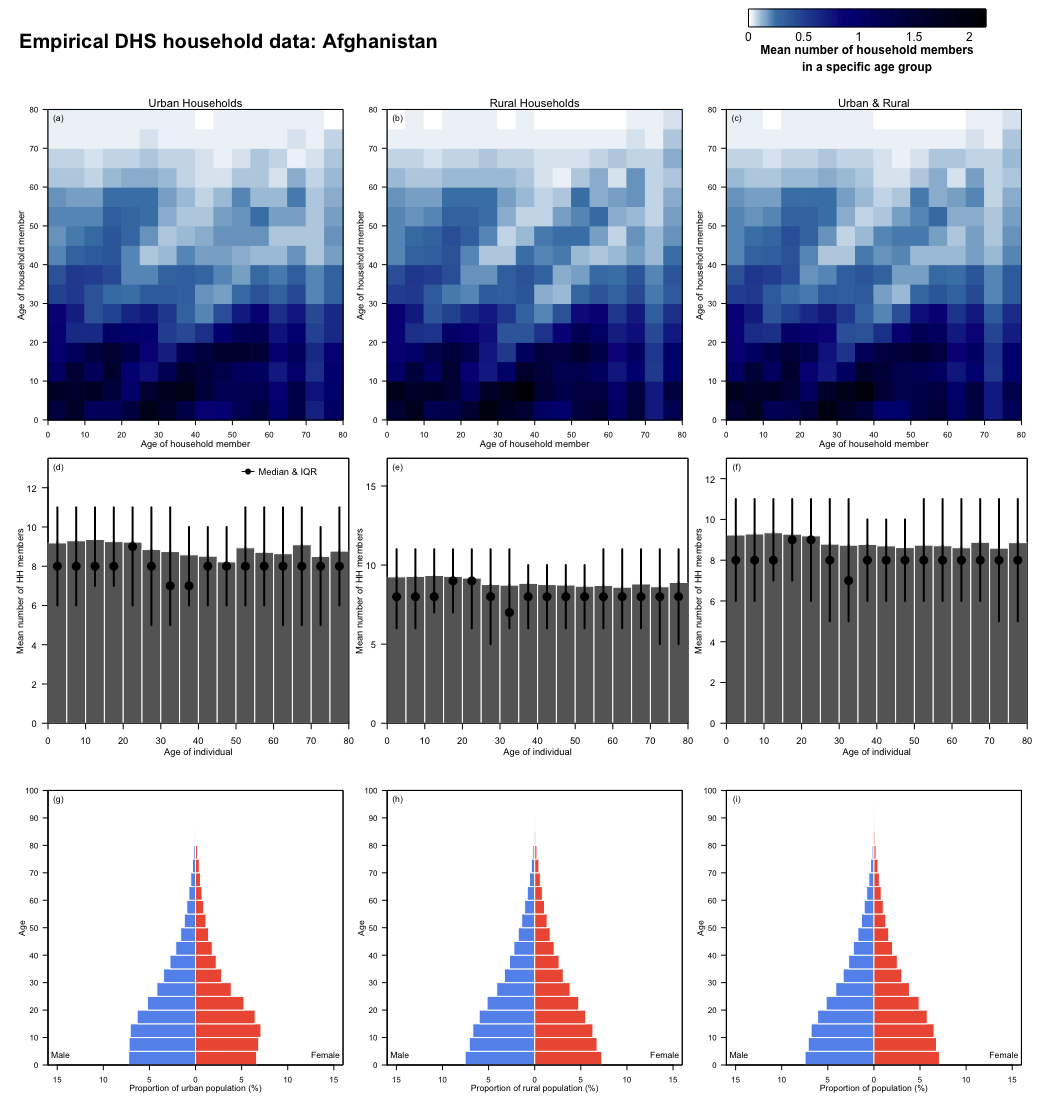 |
| --- |


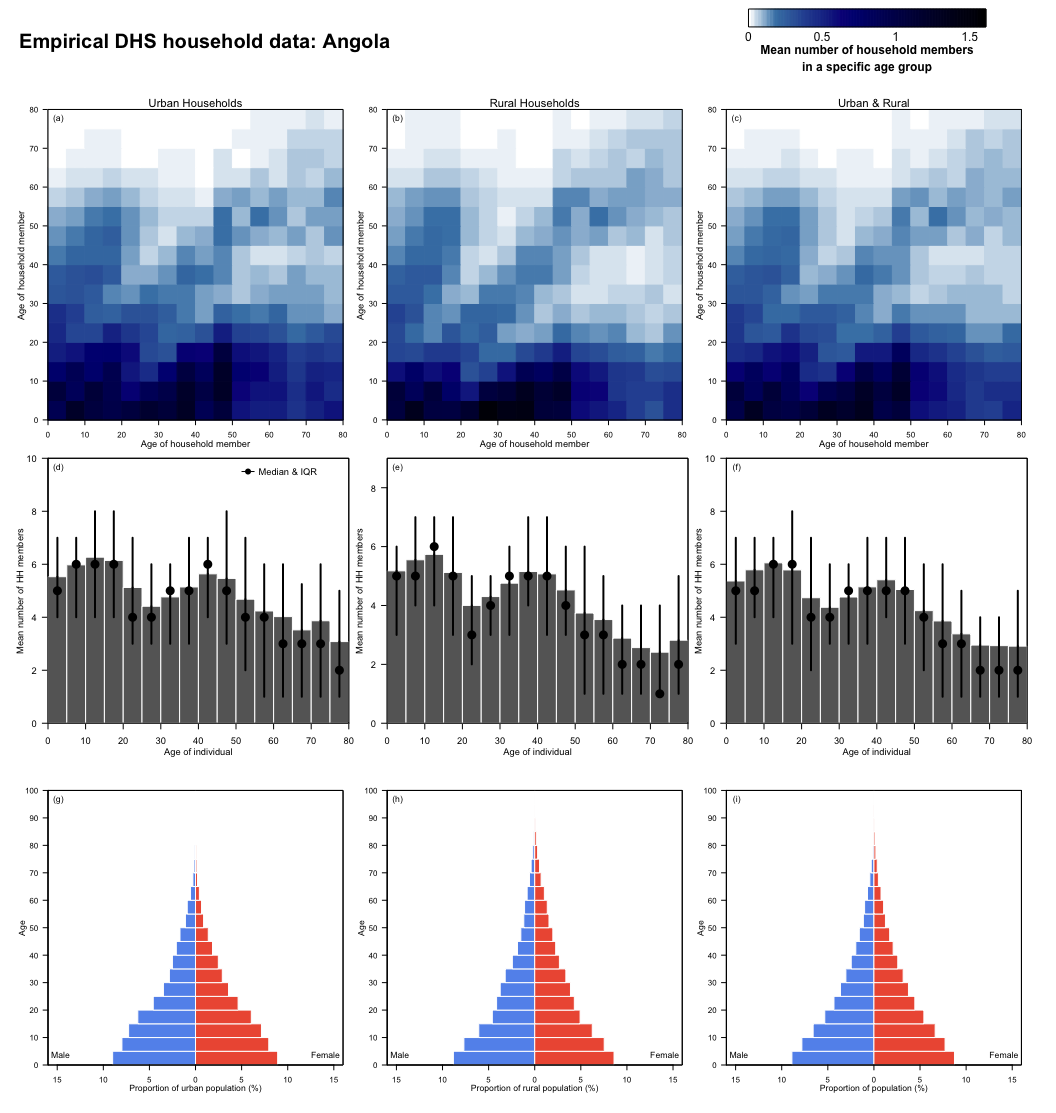

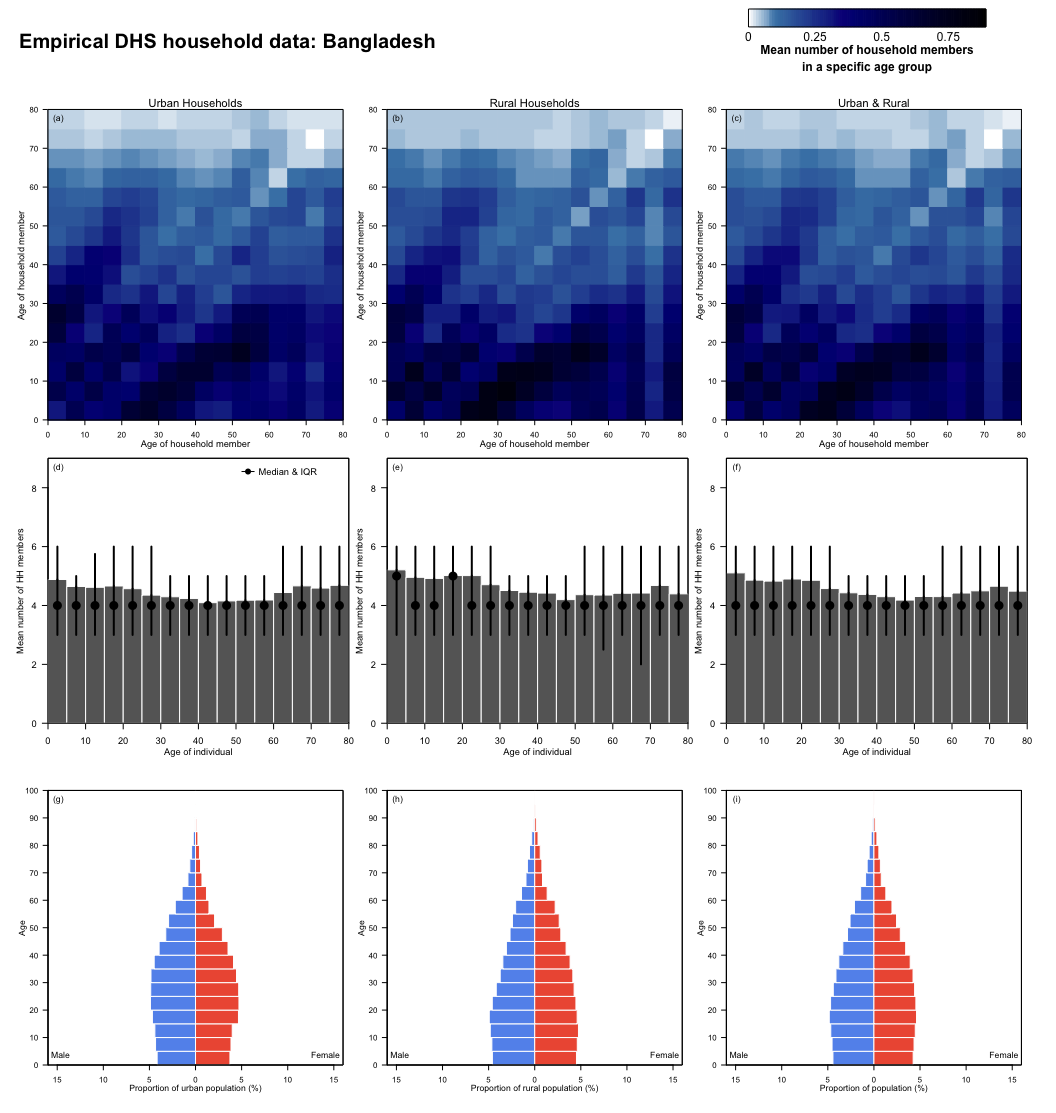

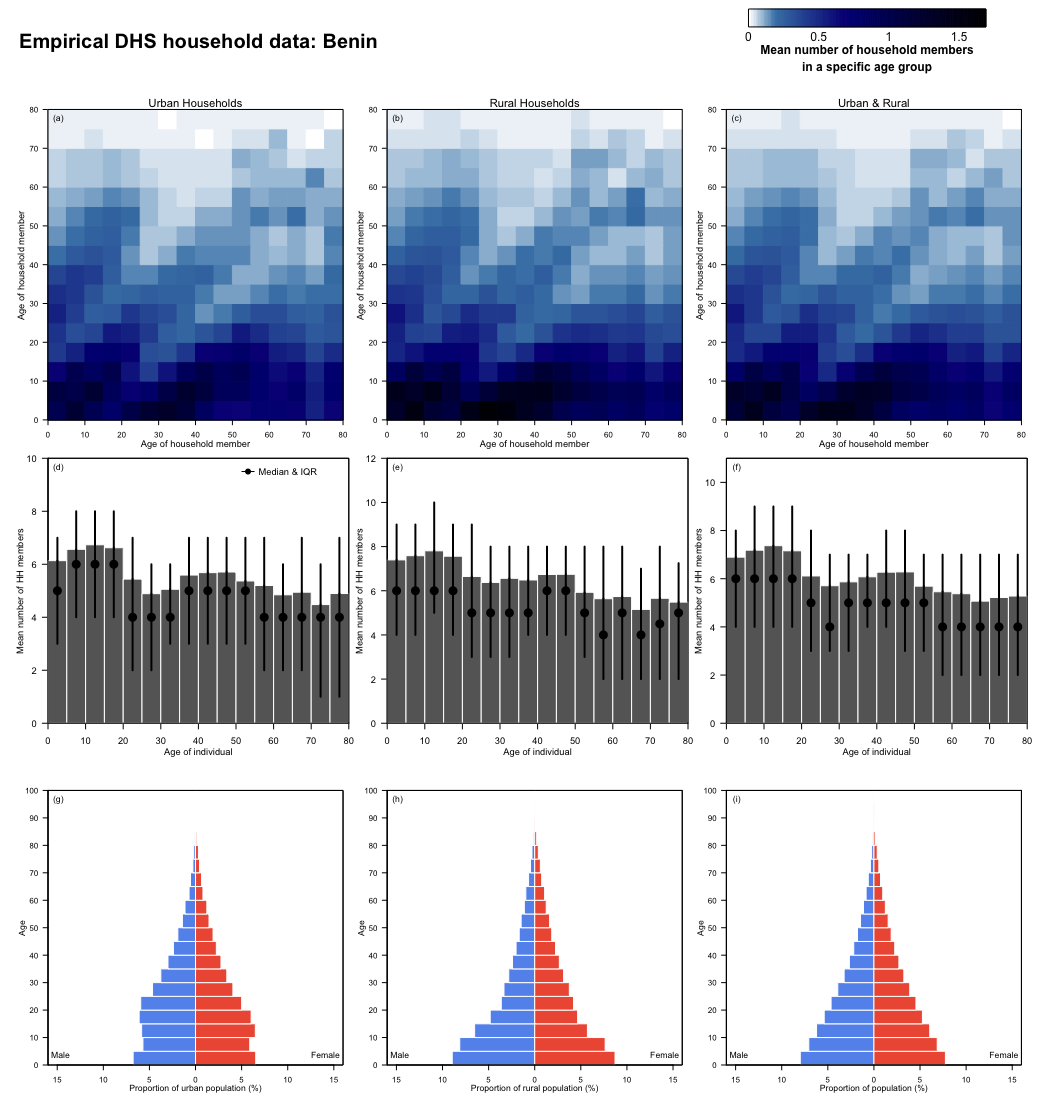

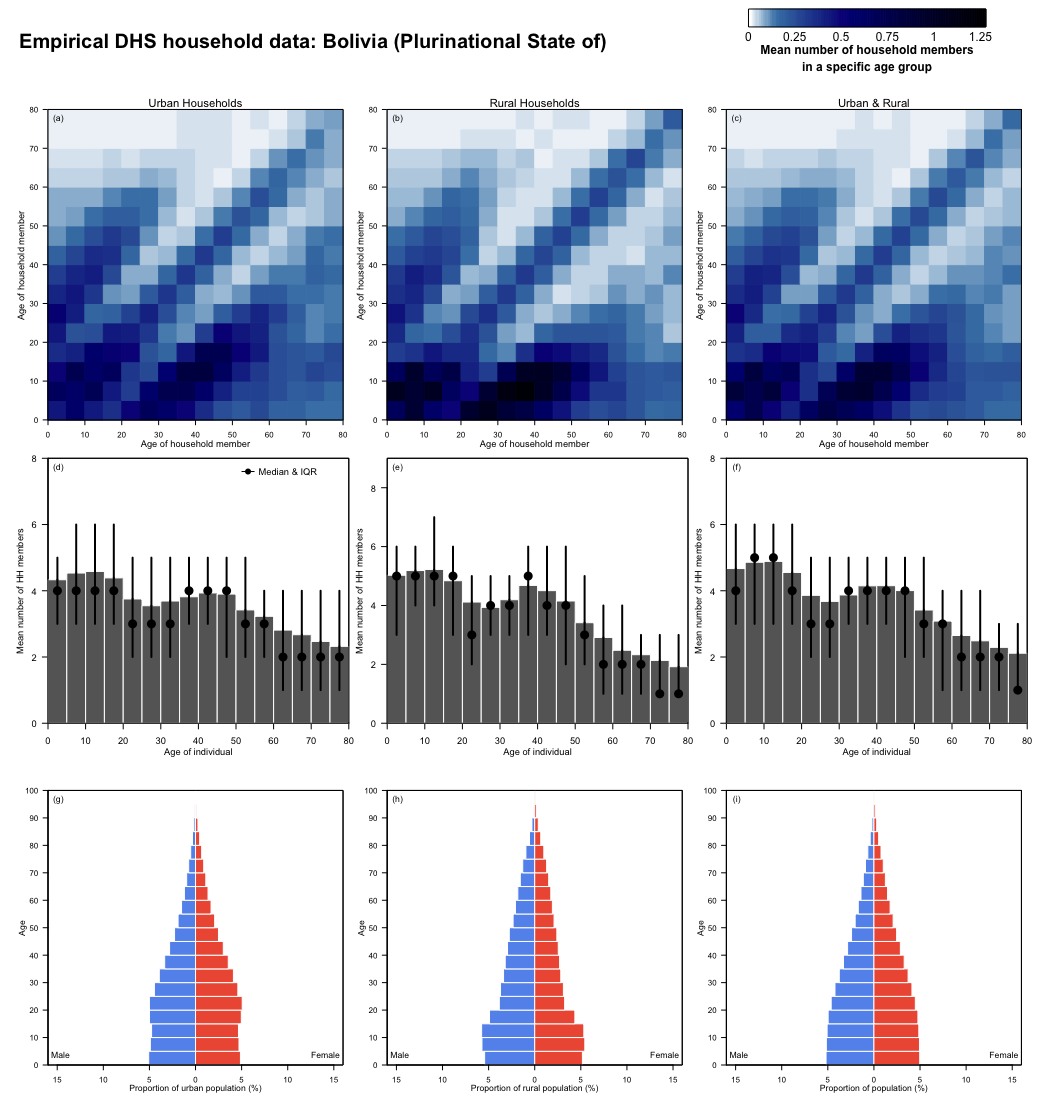

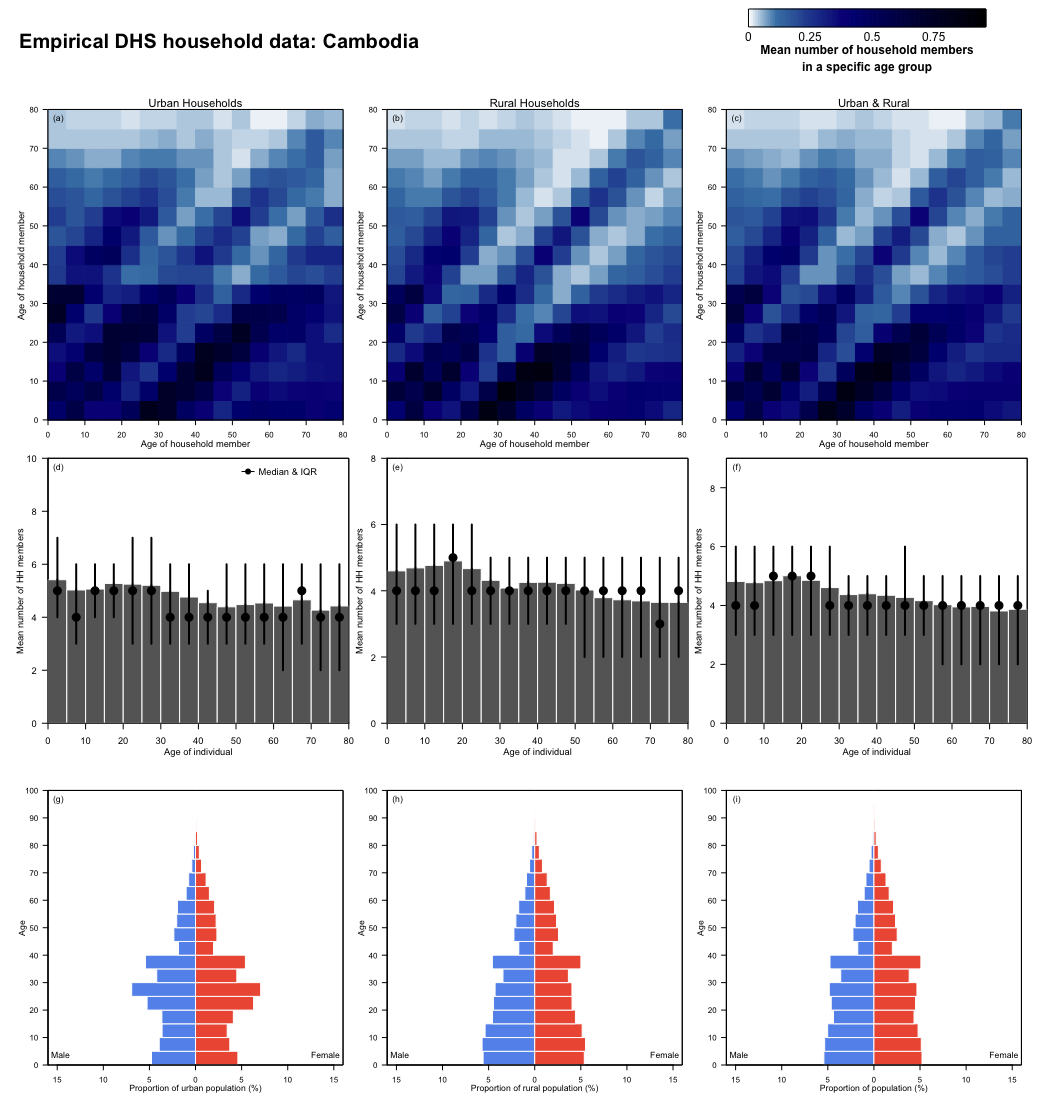

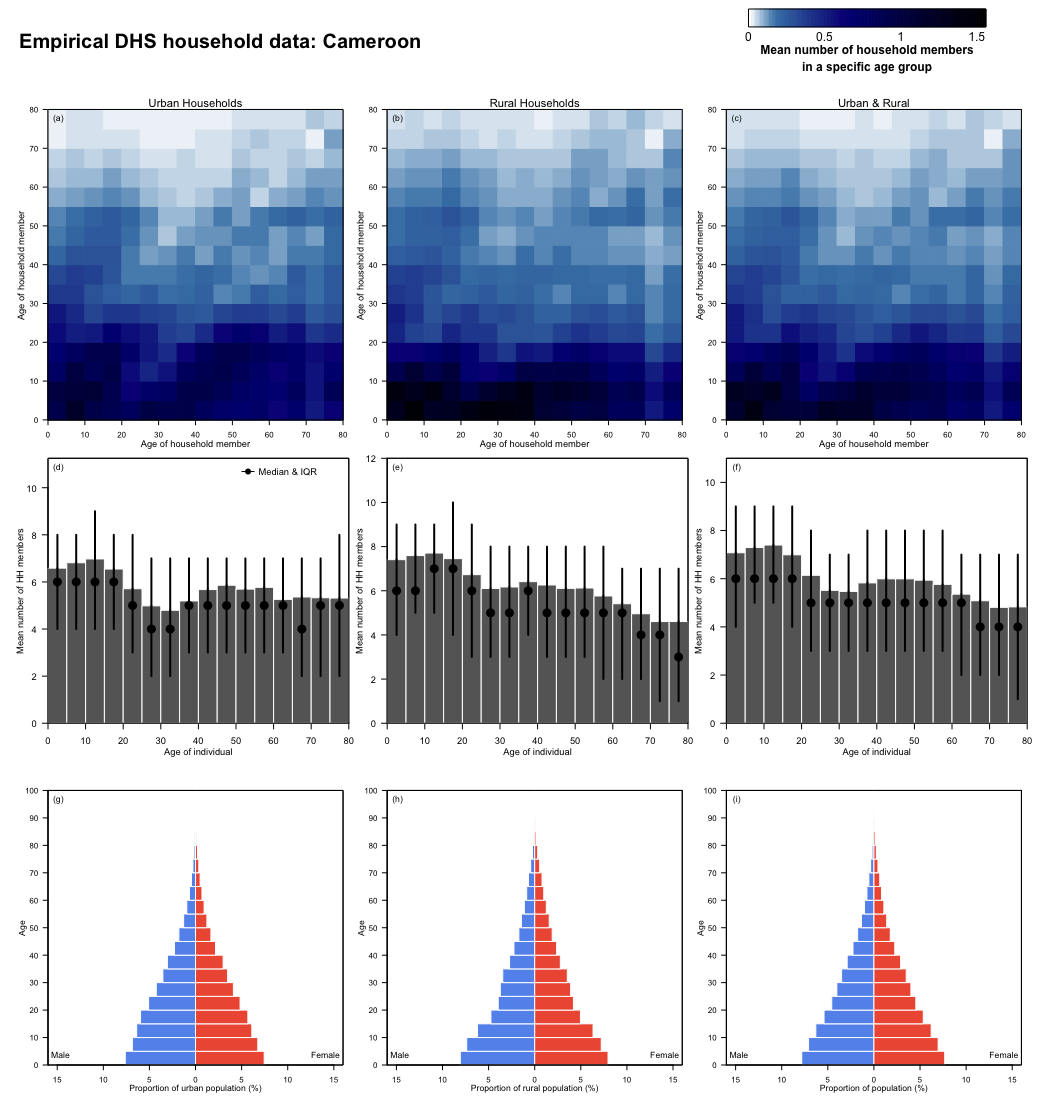

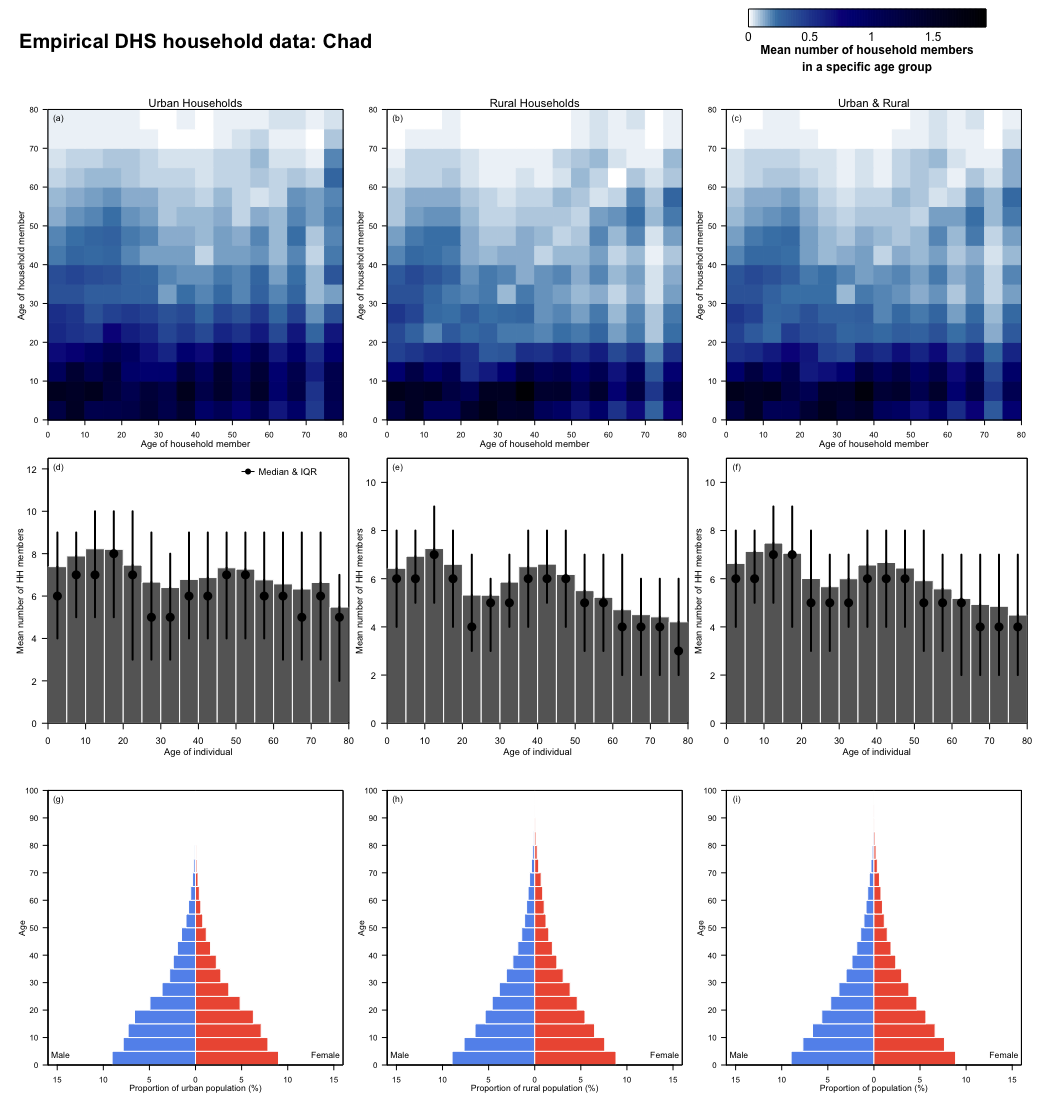

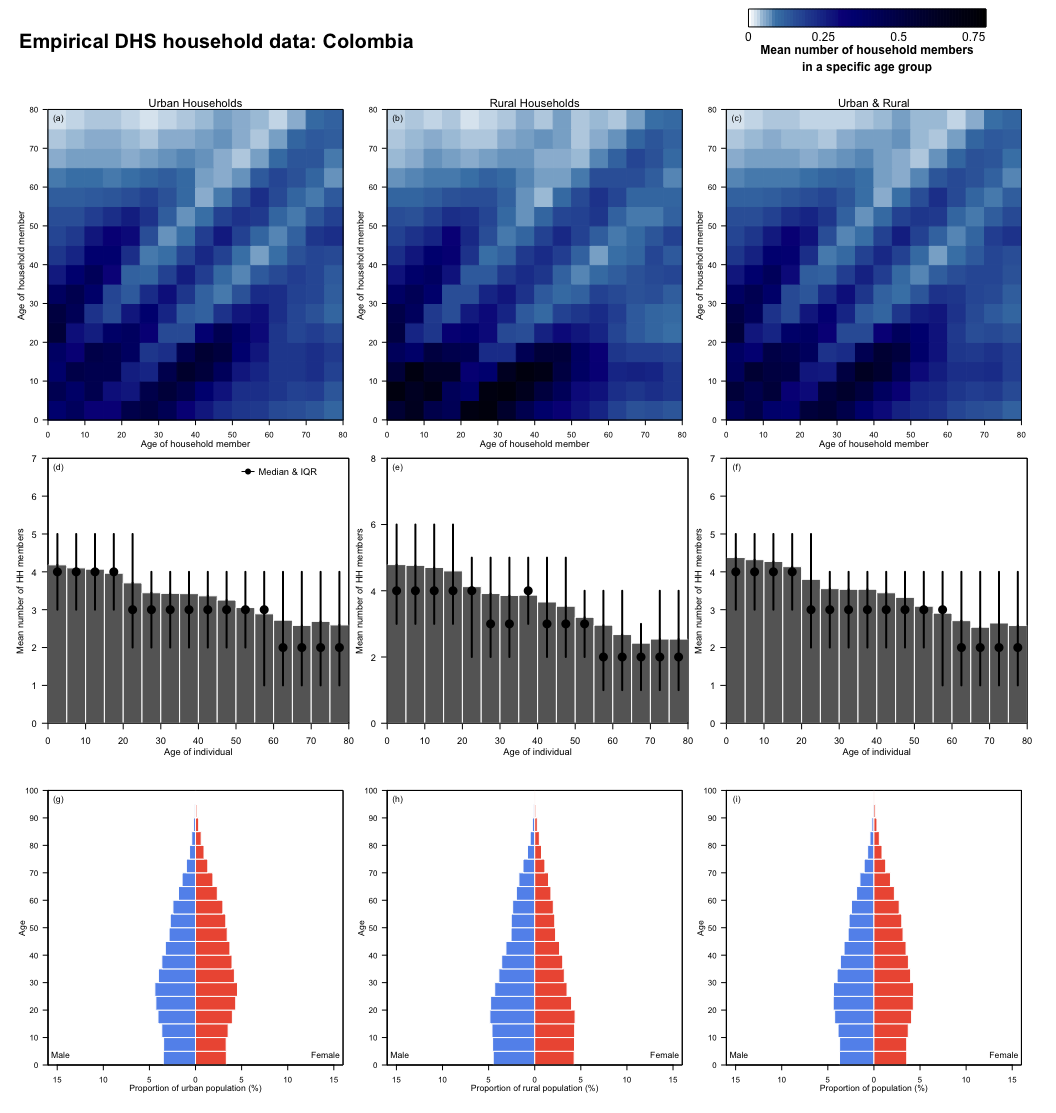

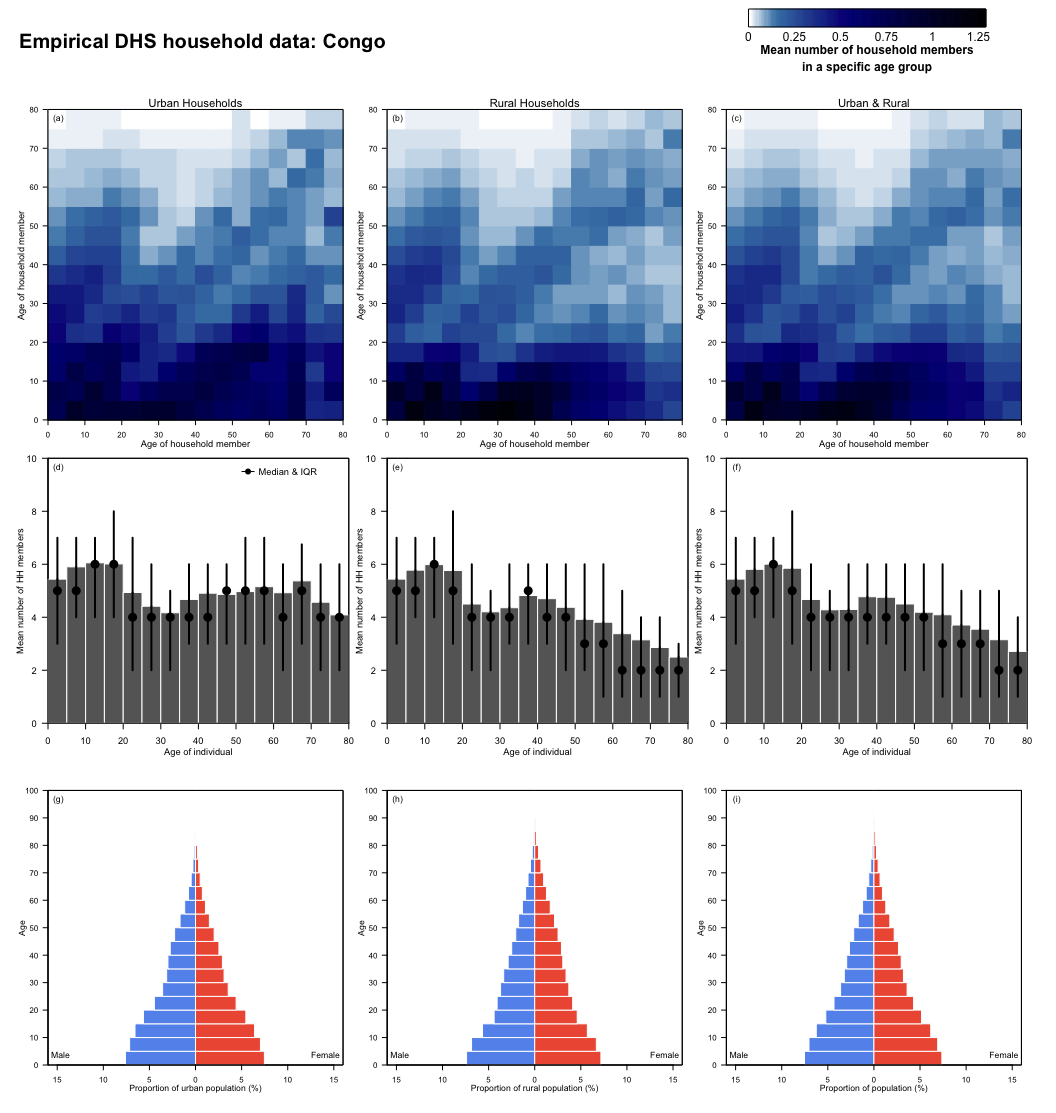

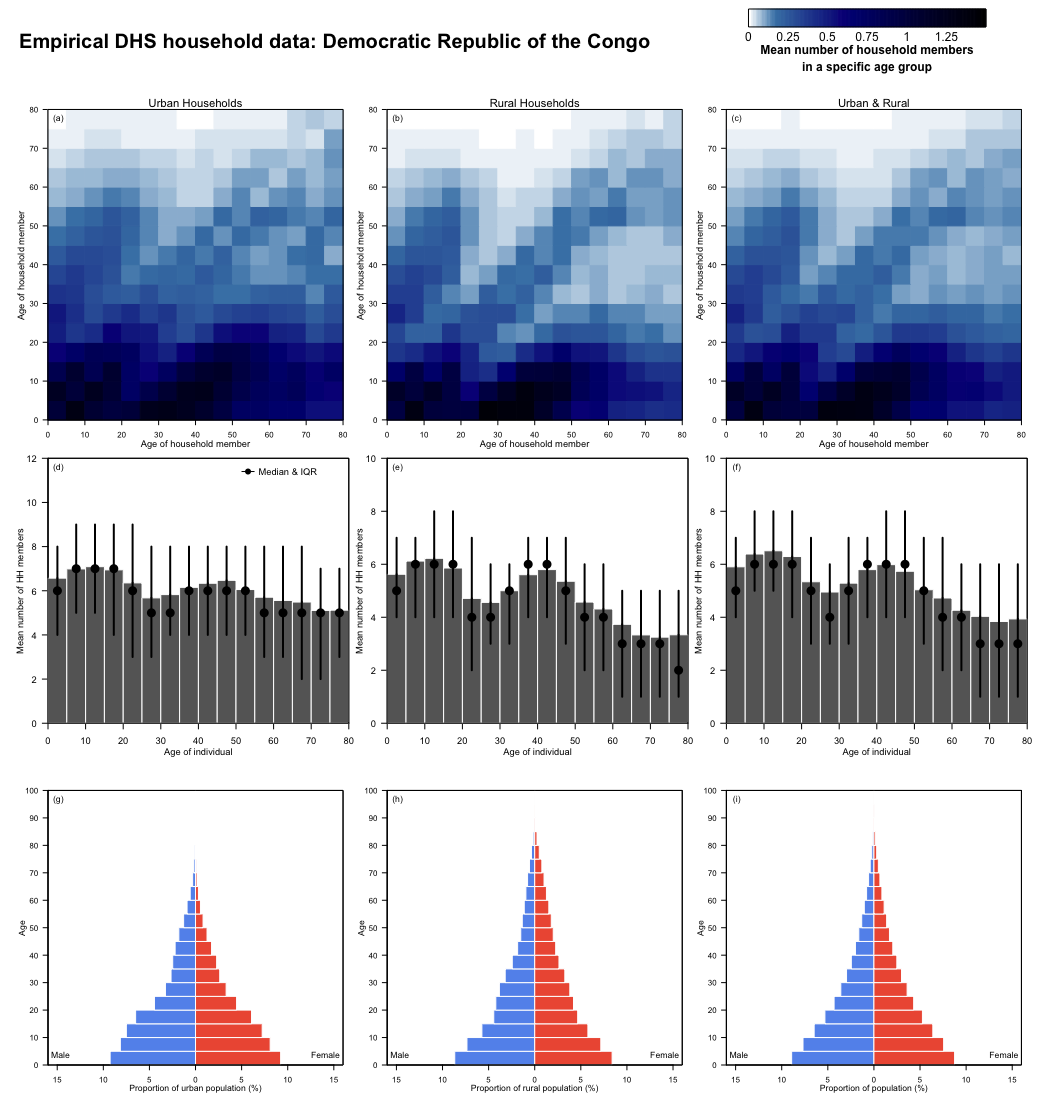

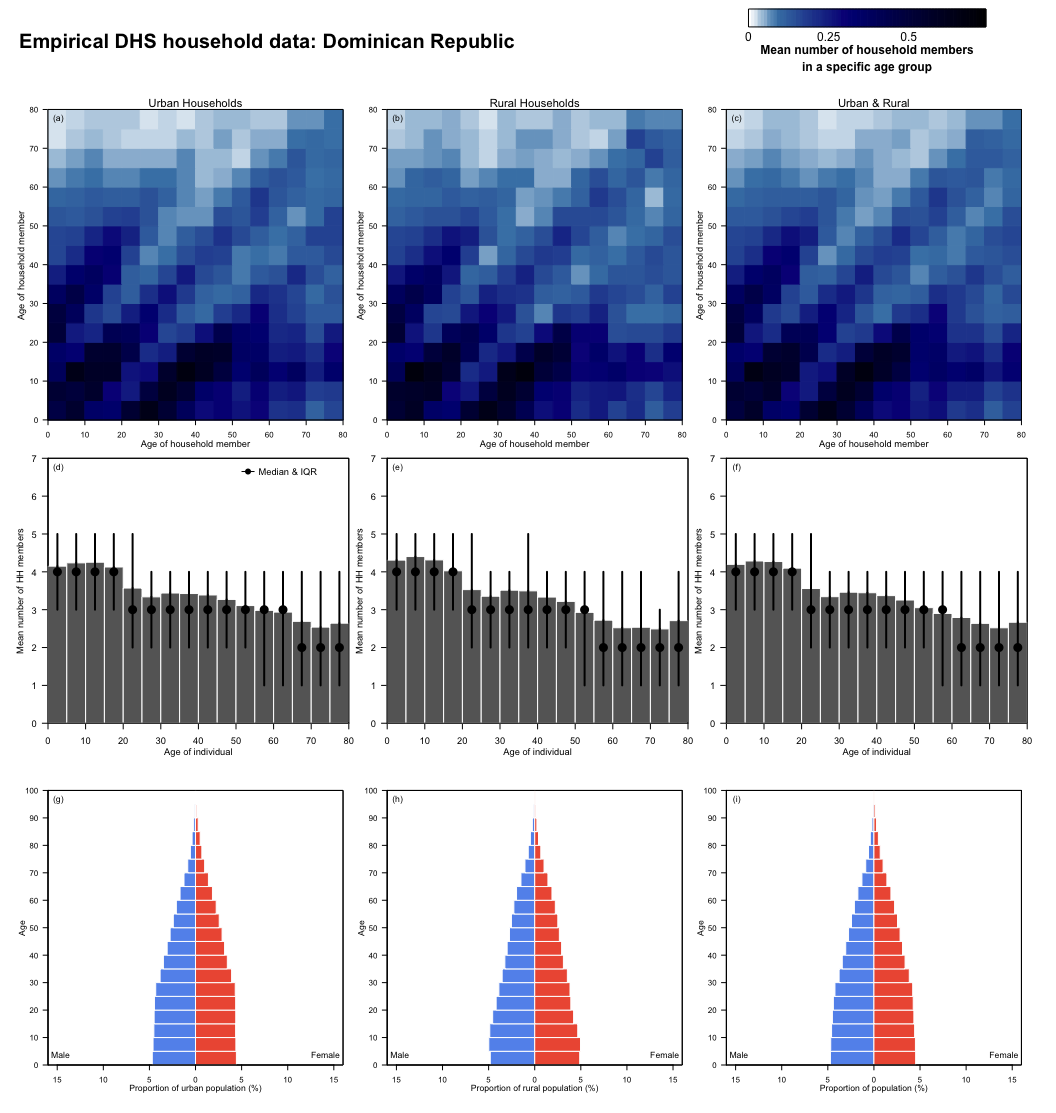

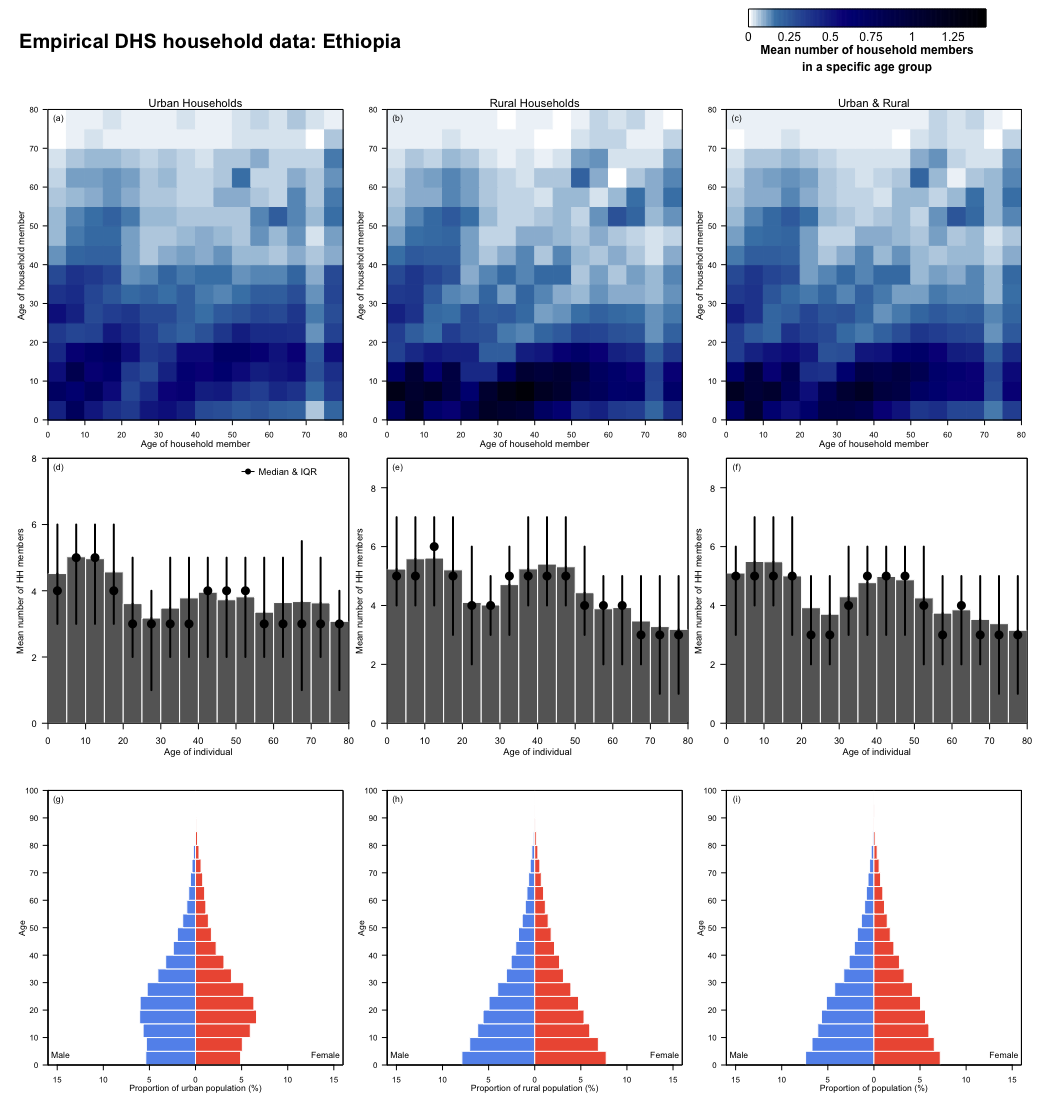

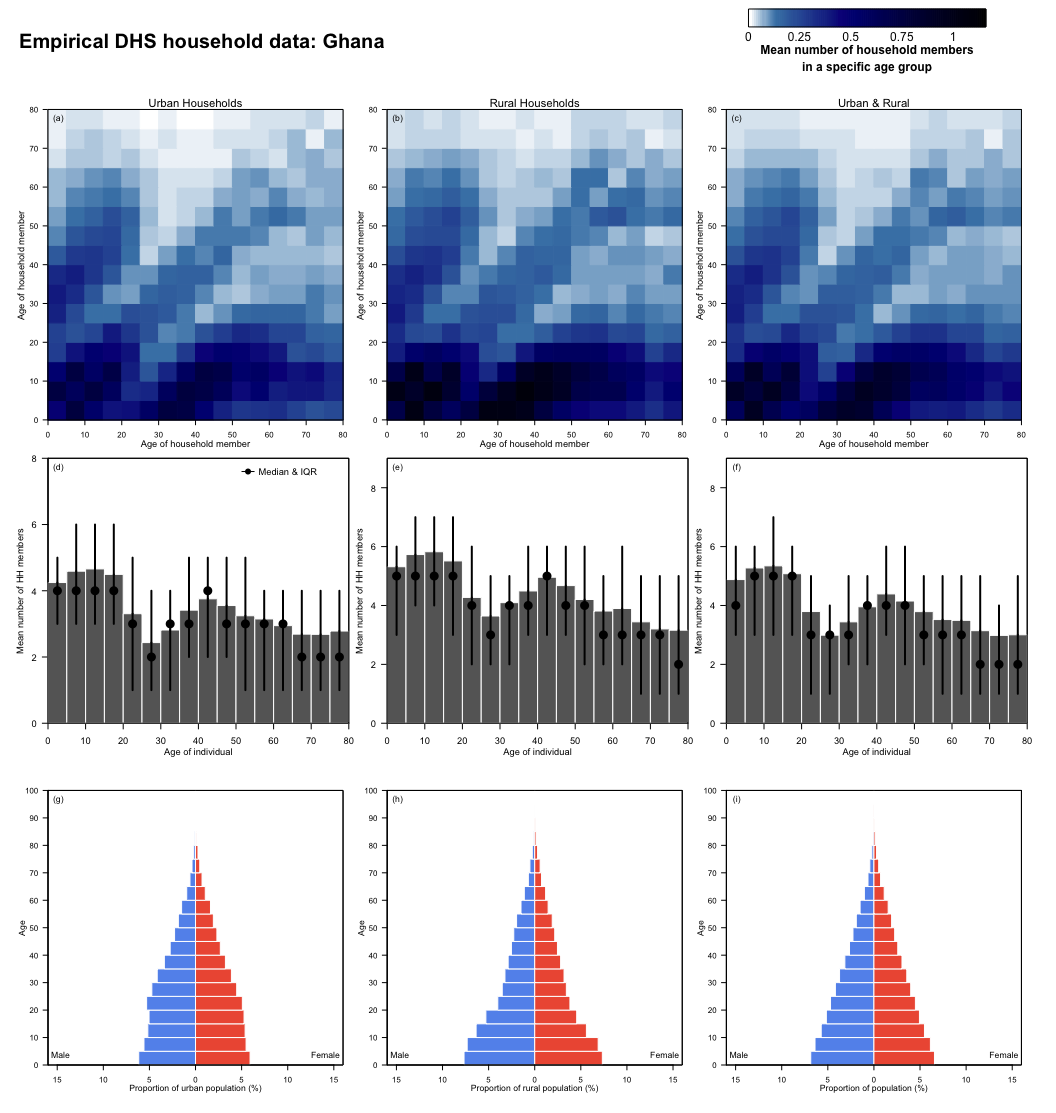

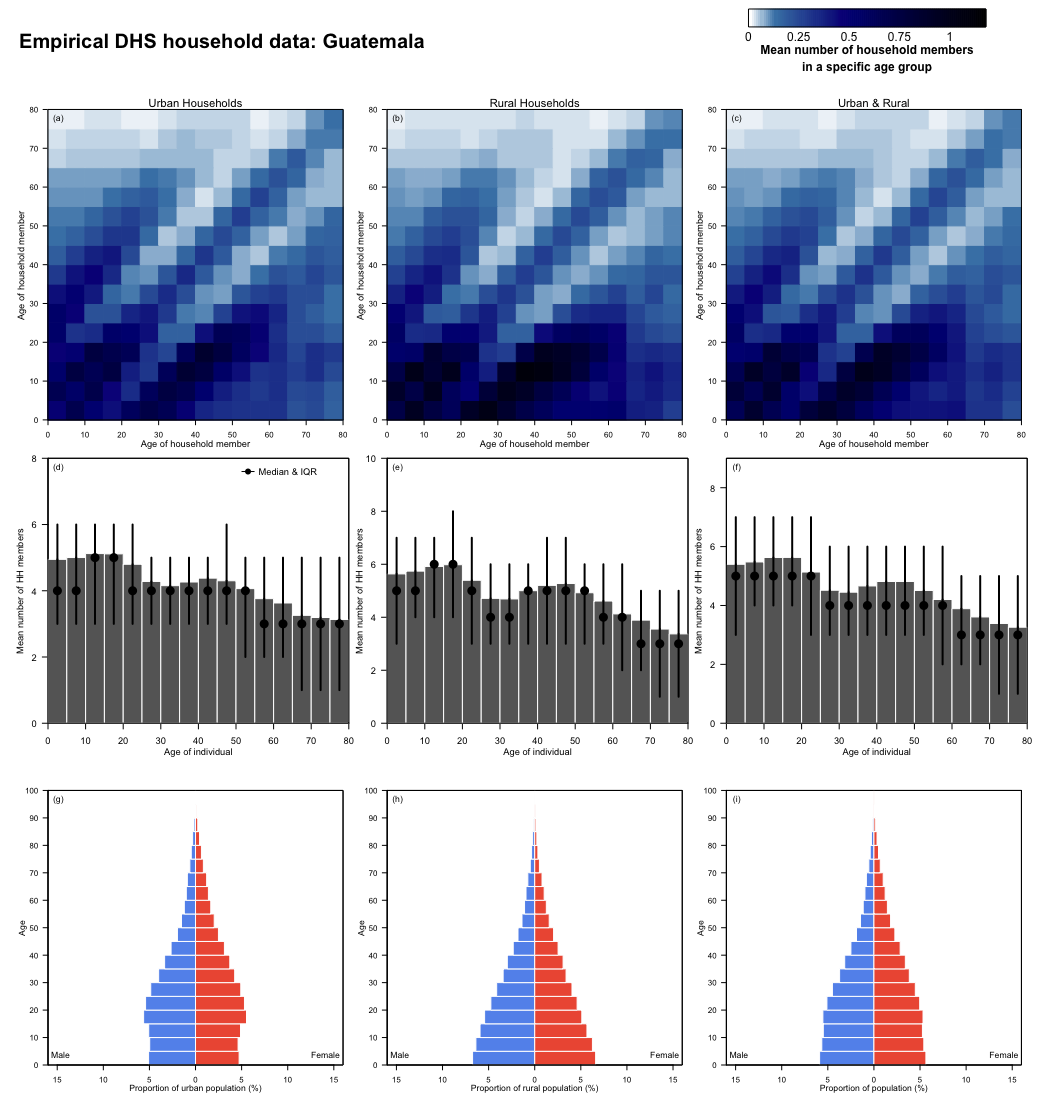

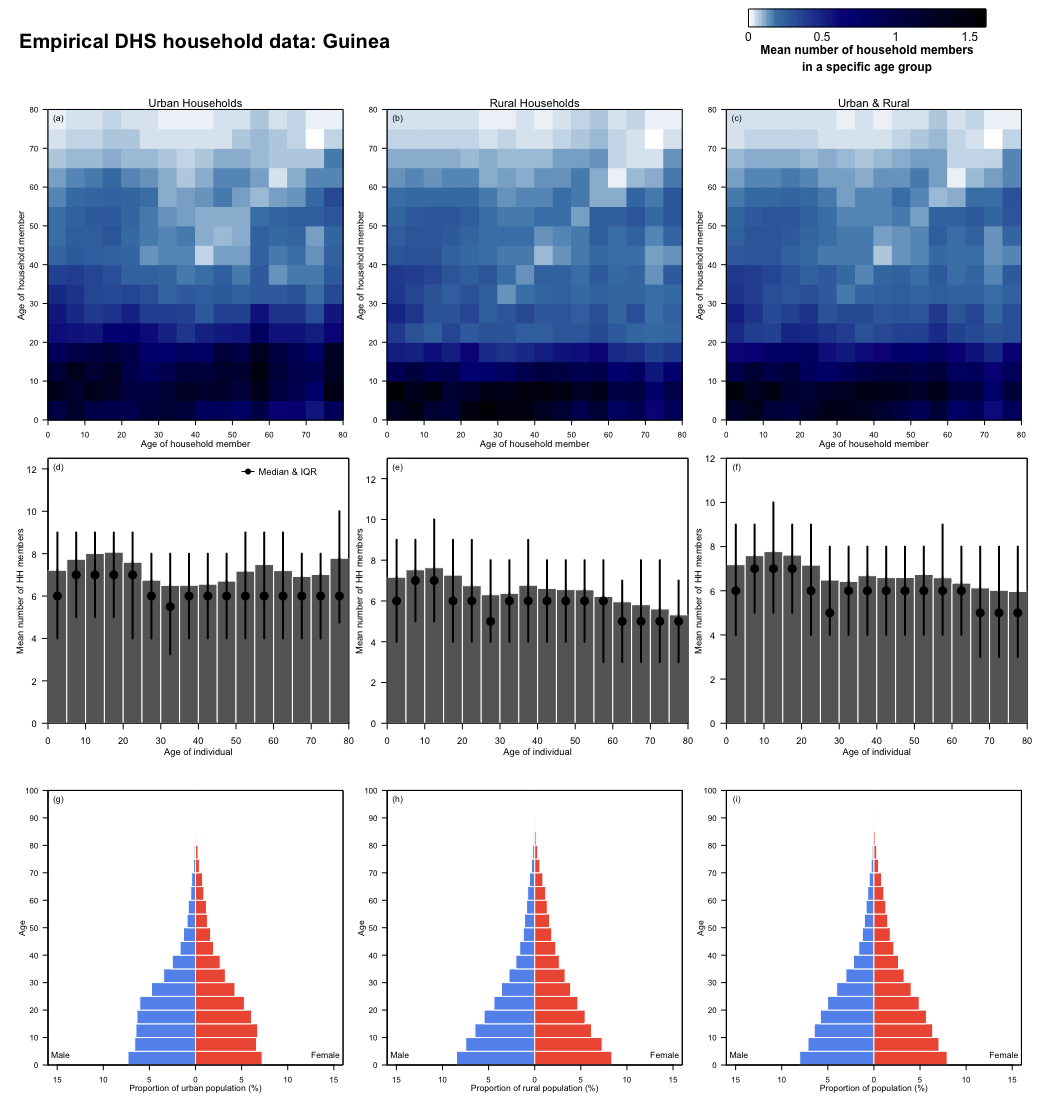

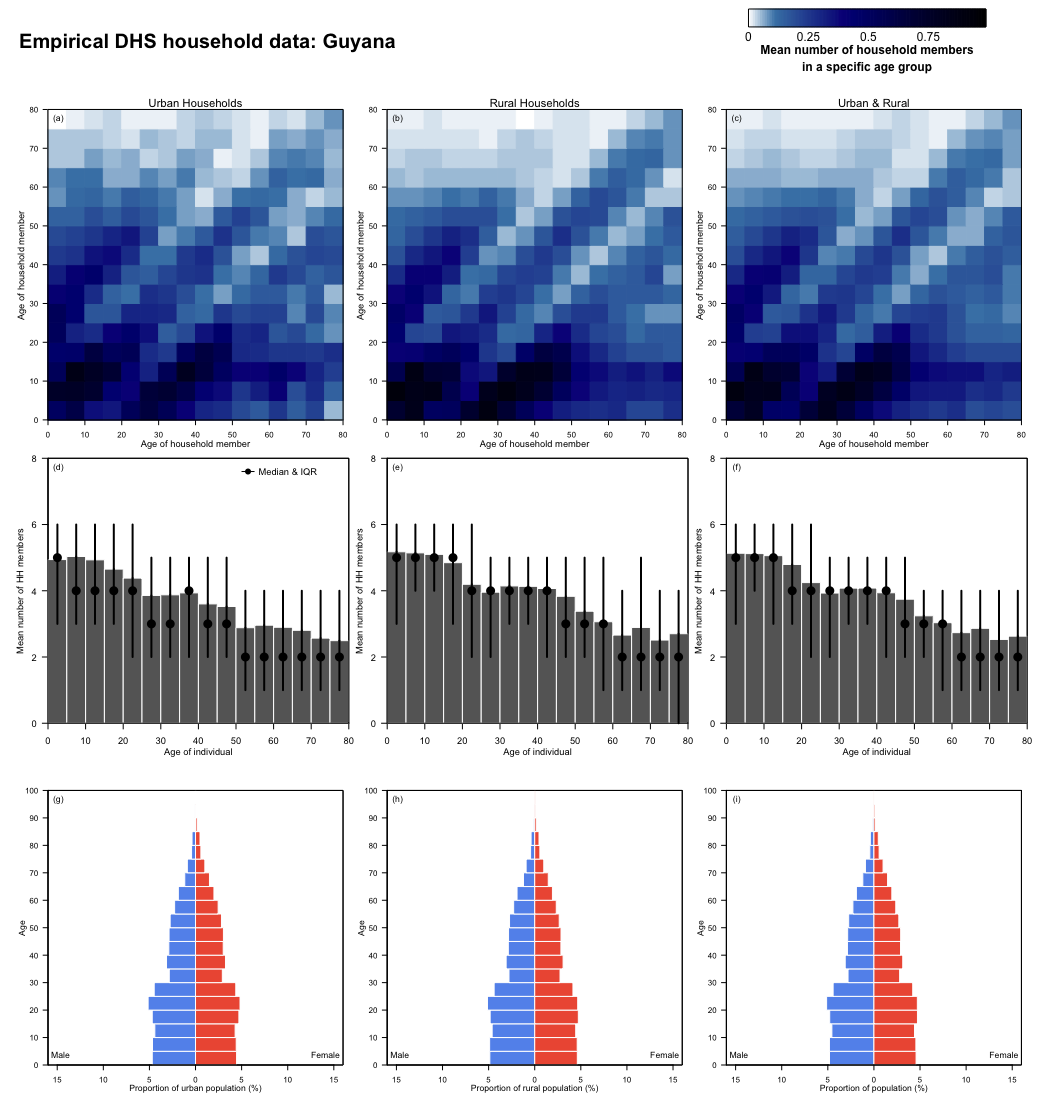

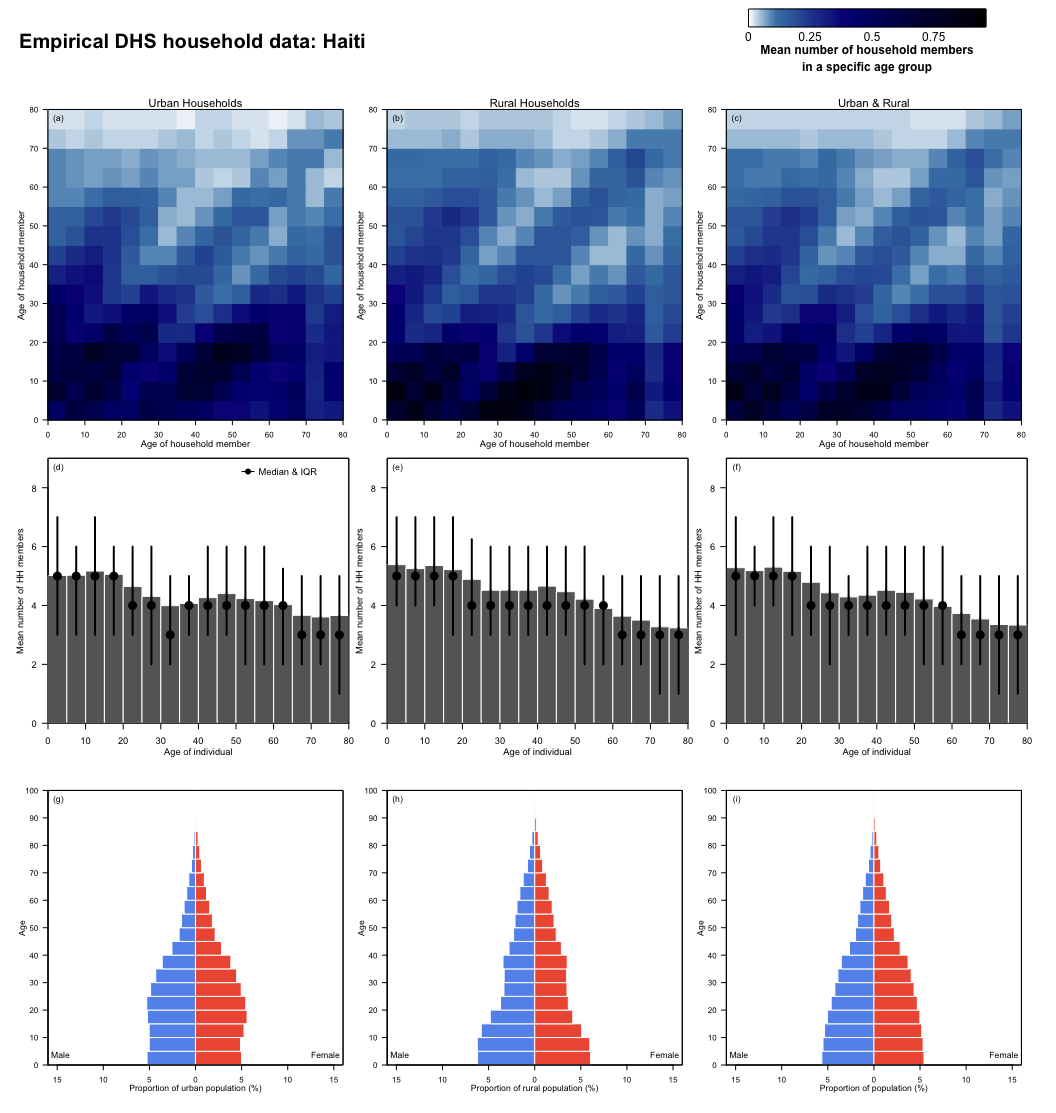

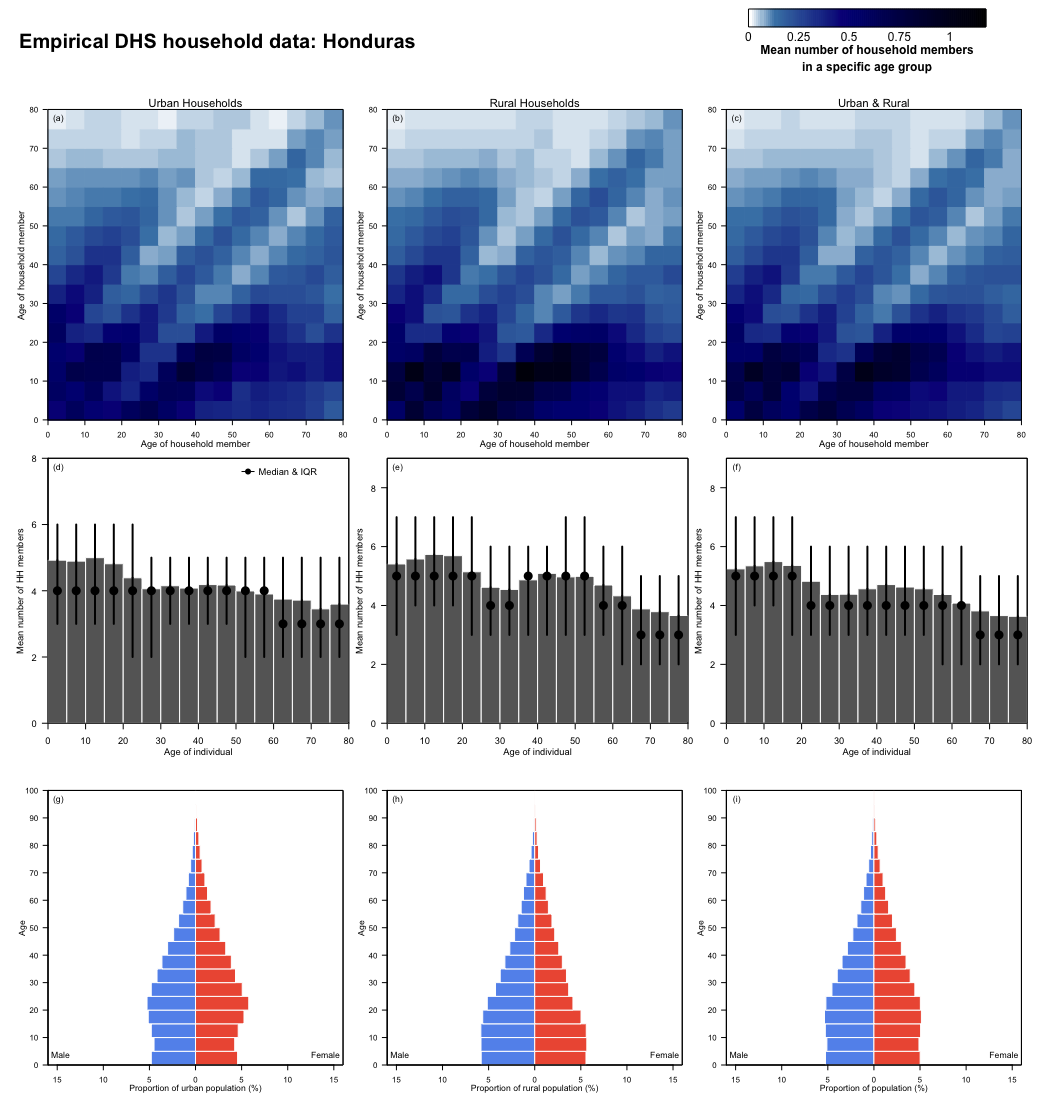

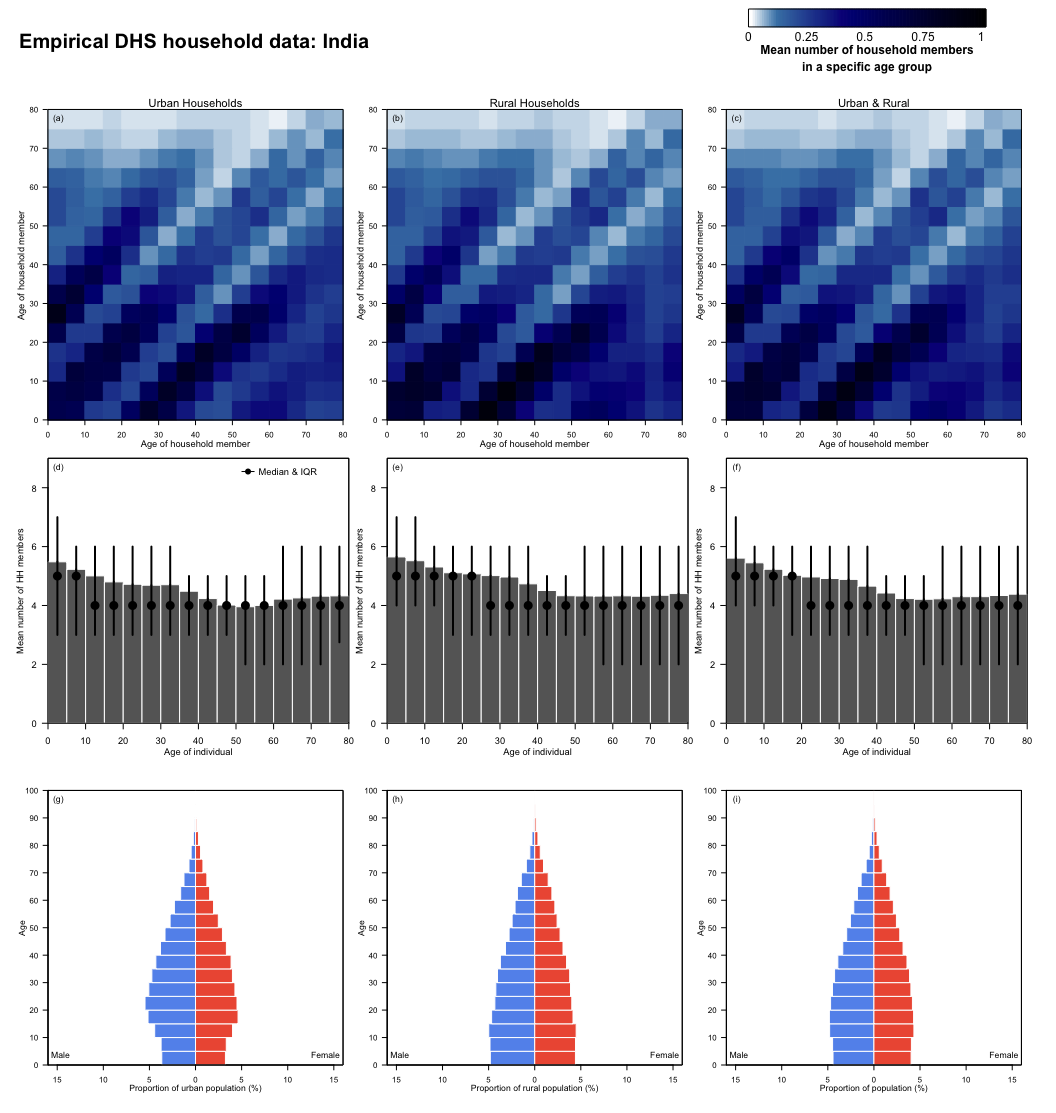

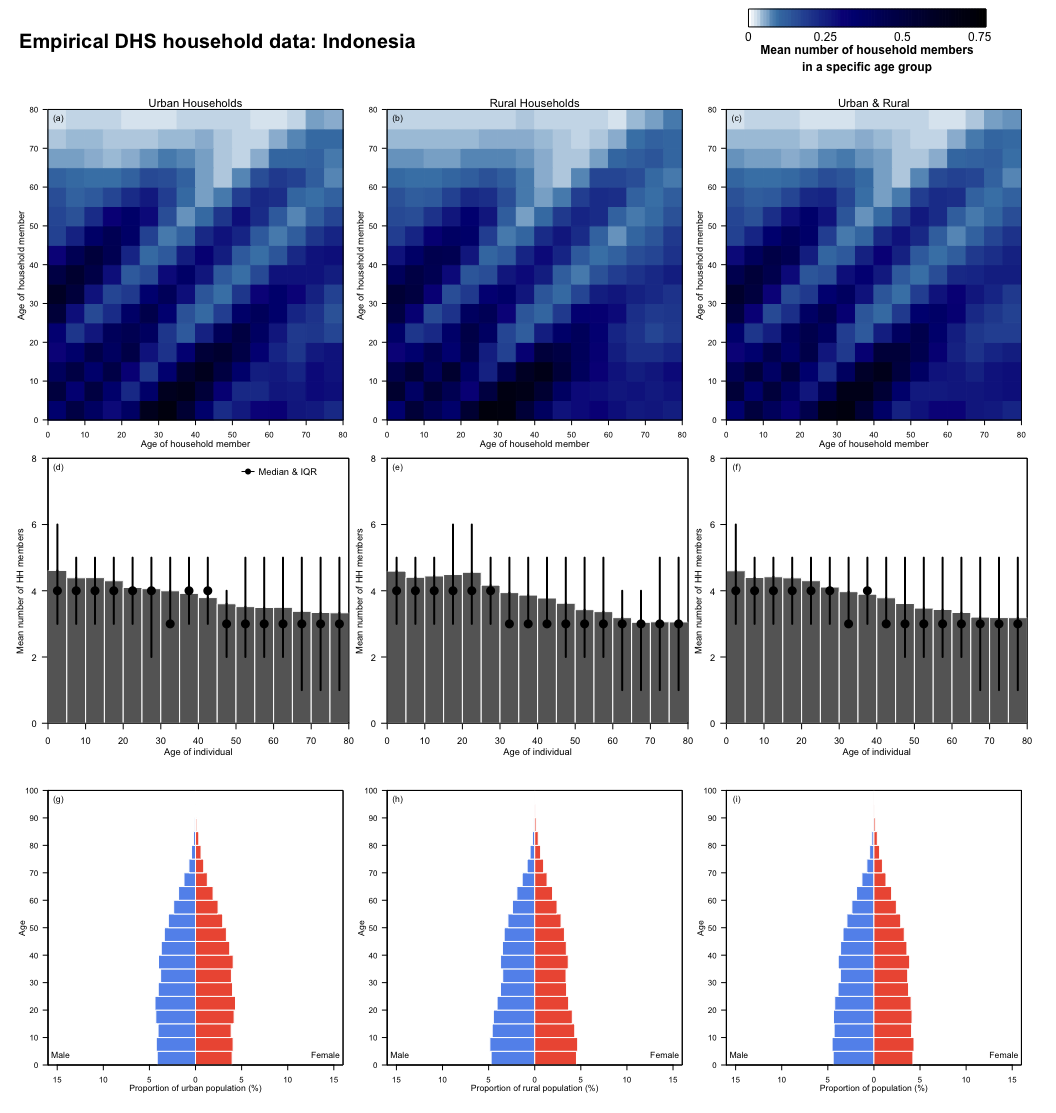

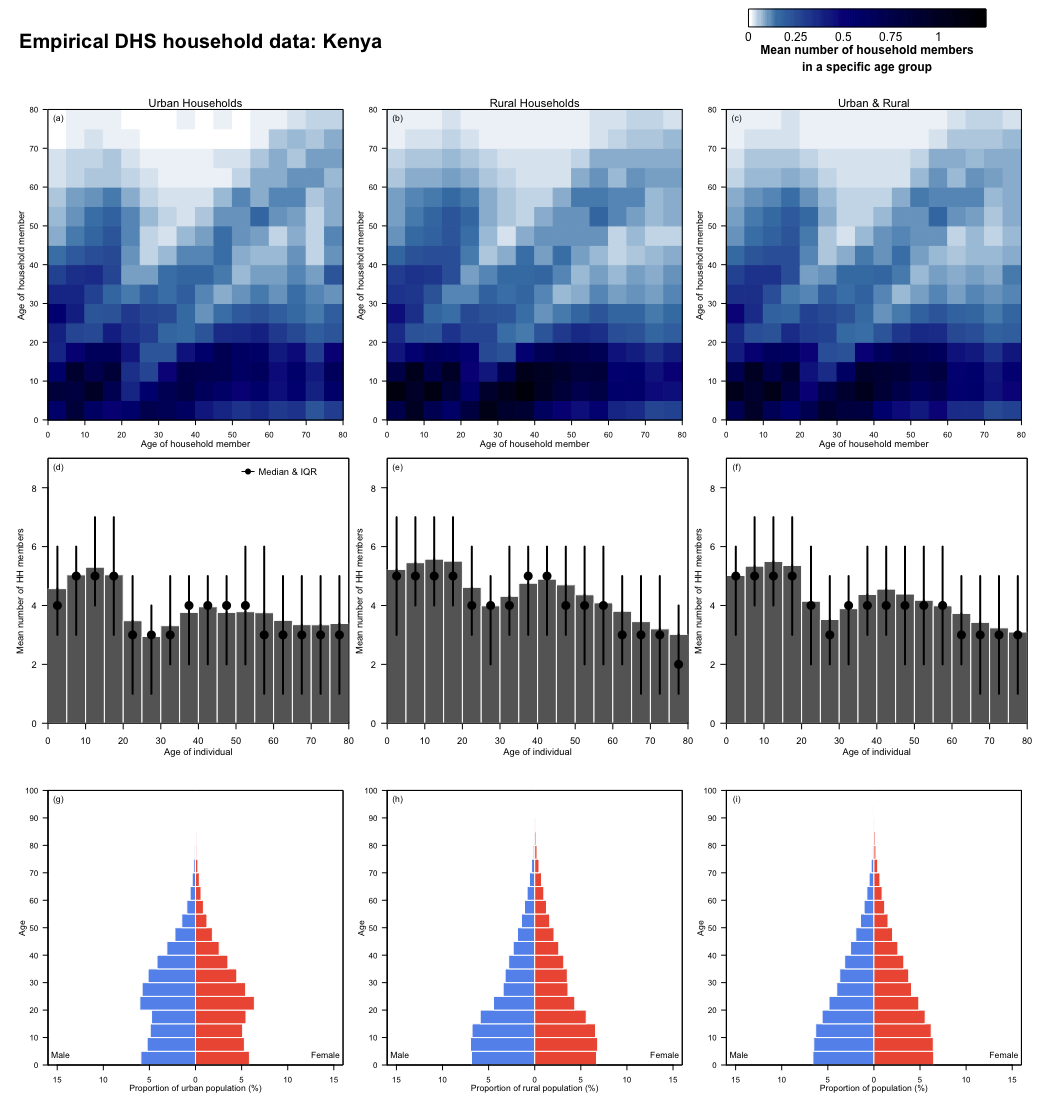

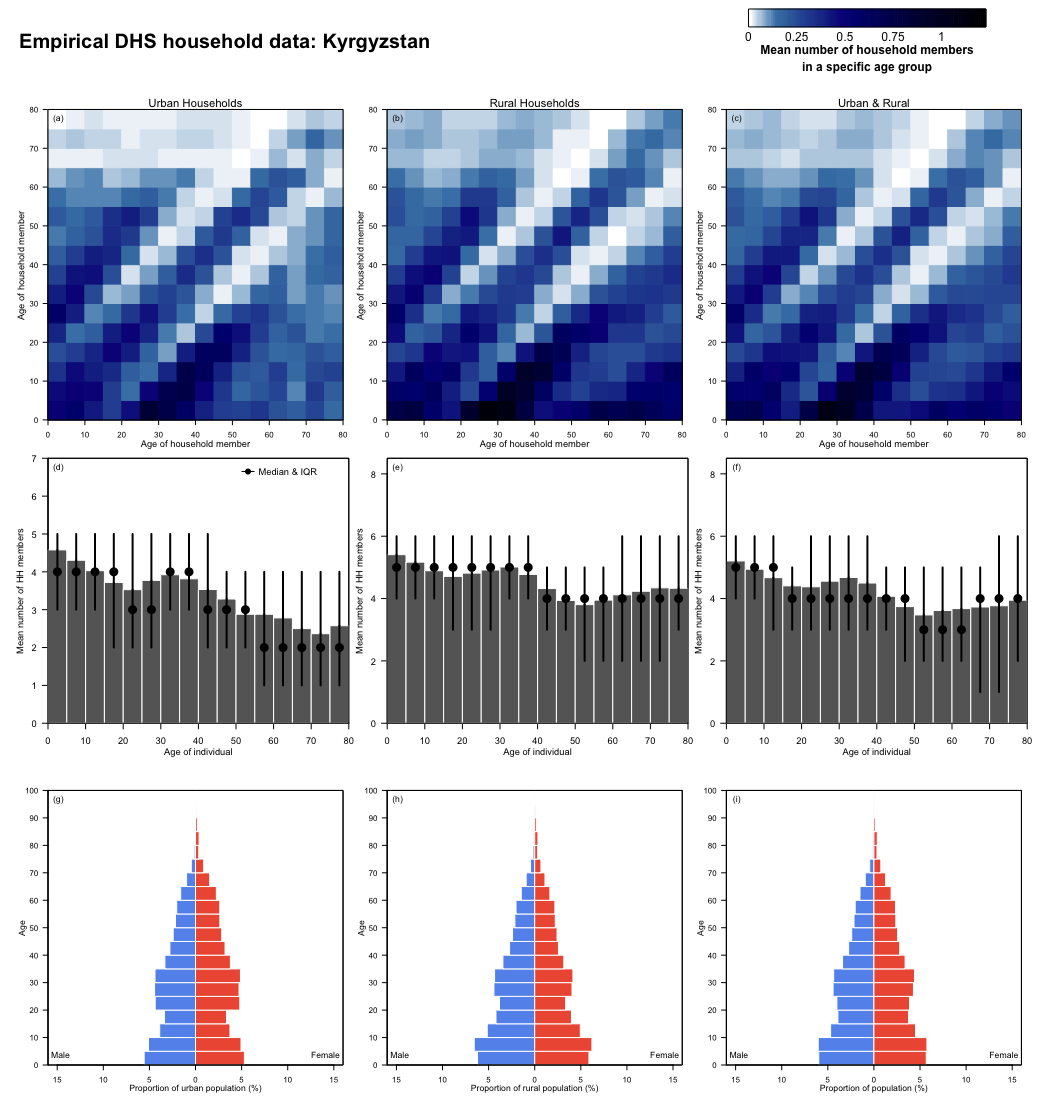

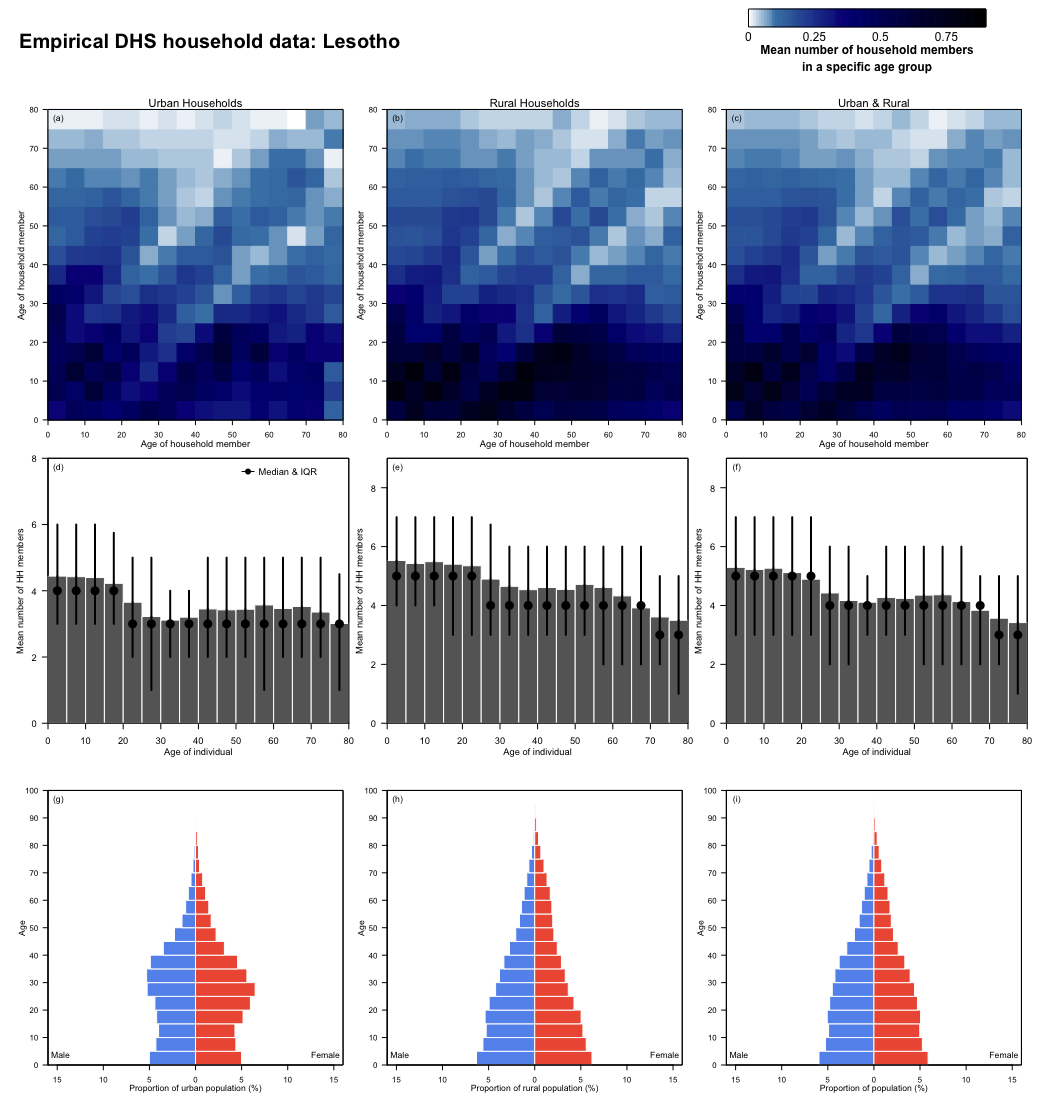

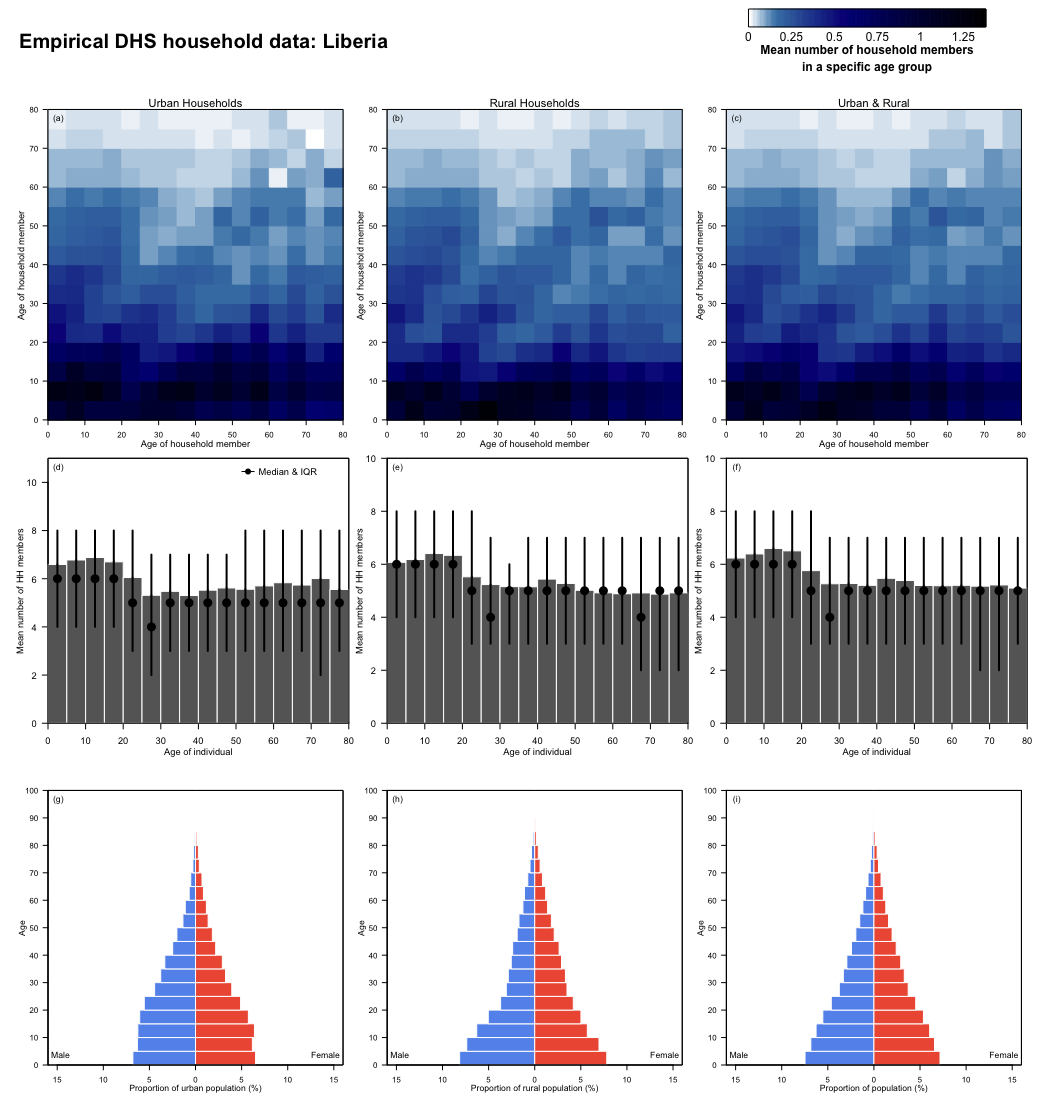

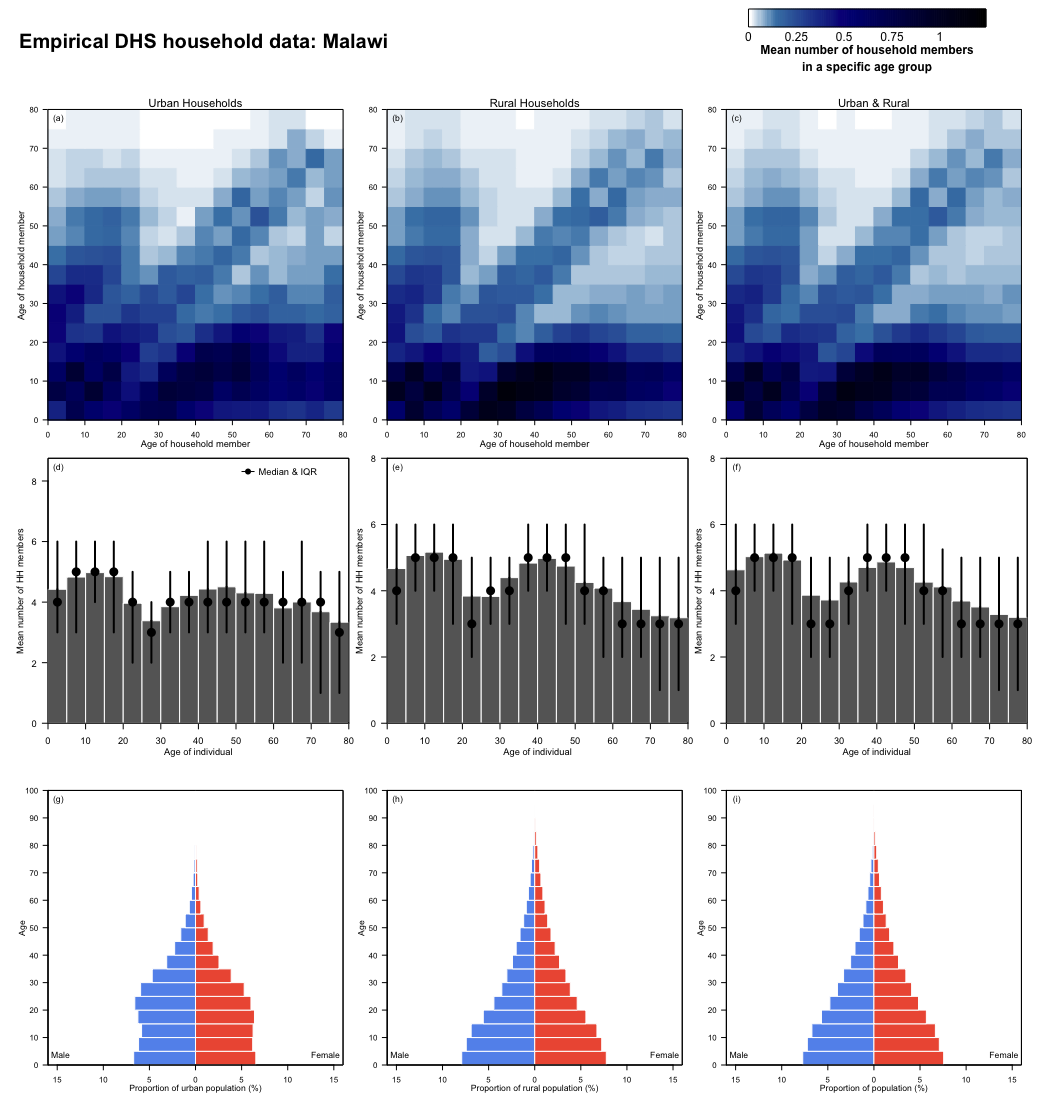

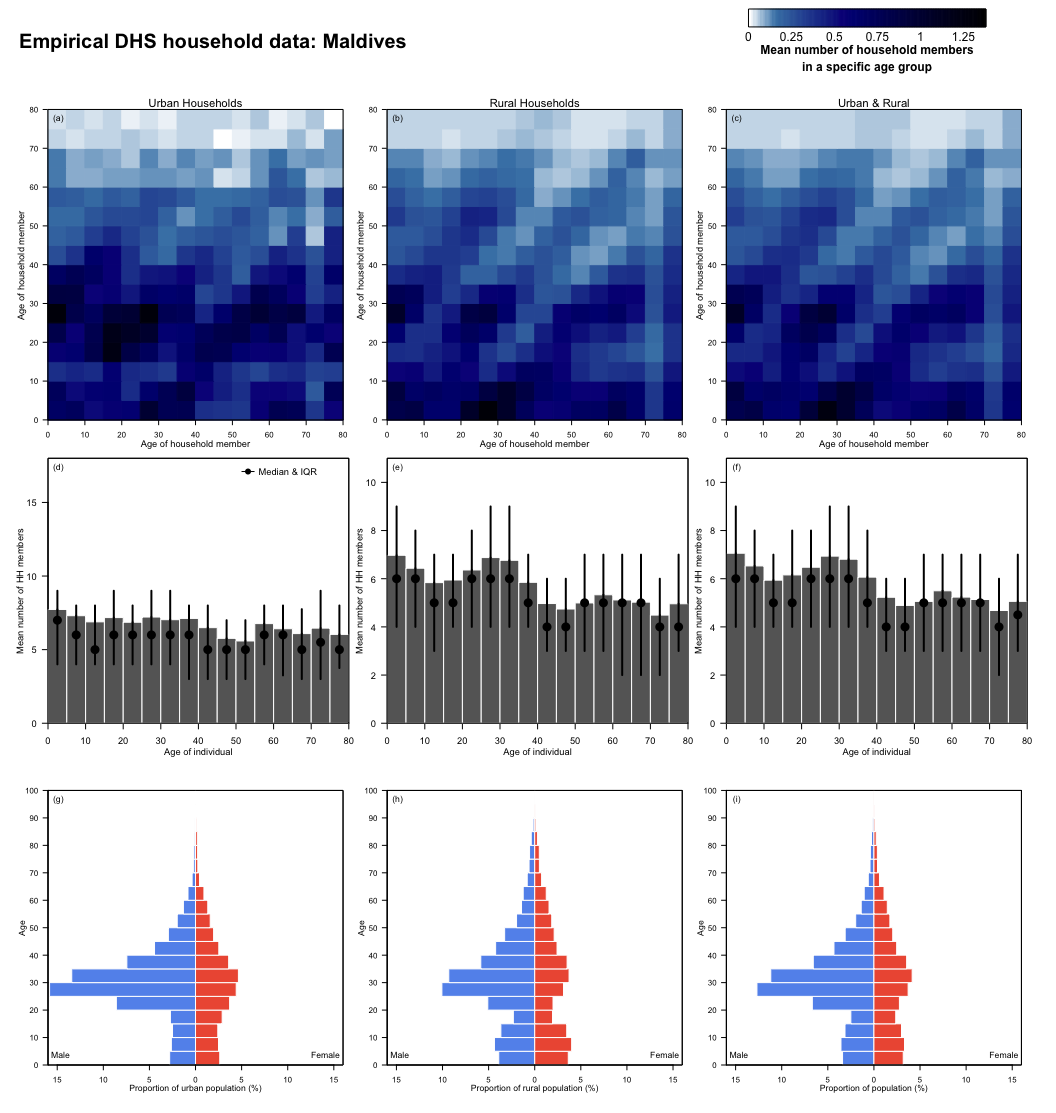

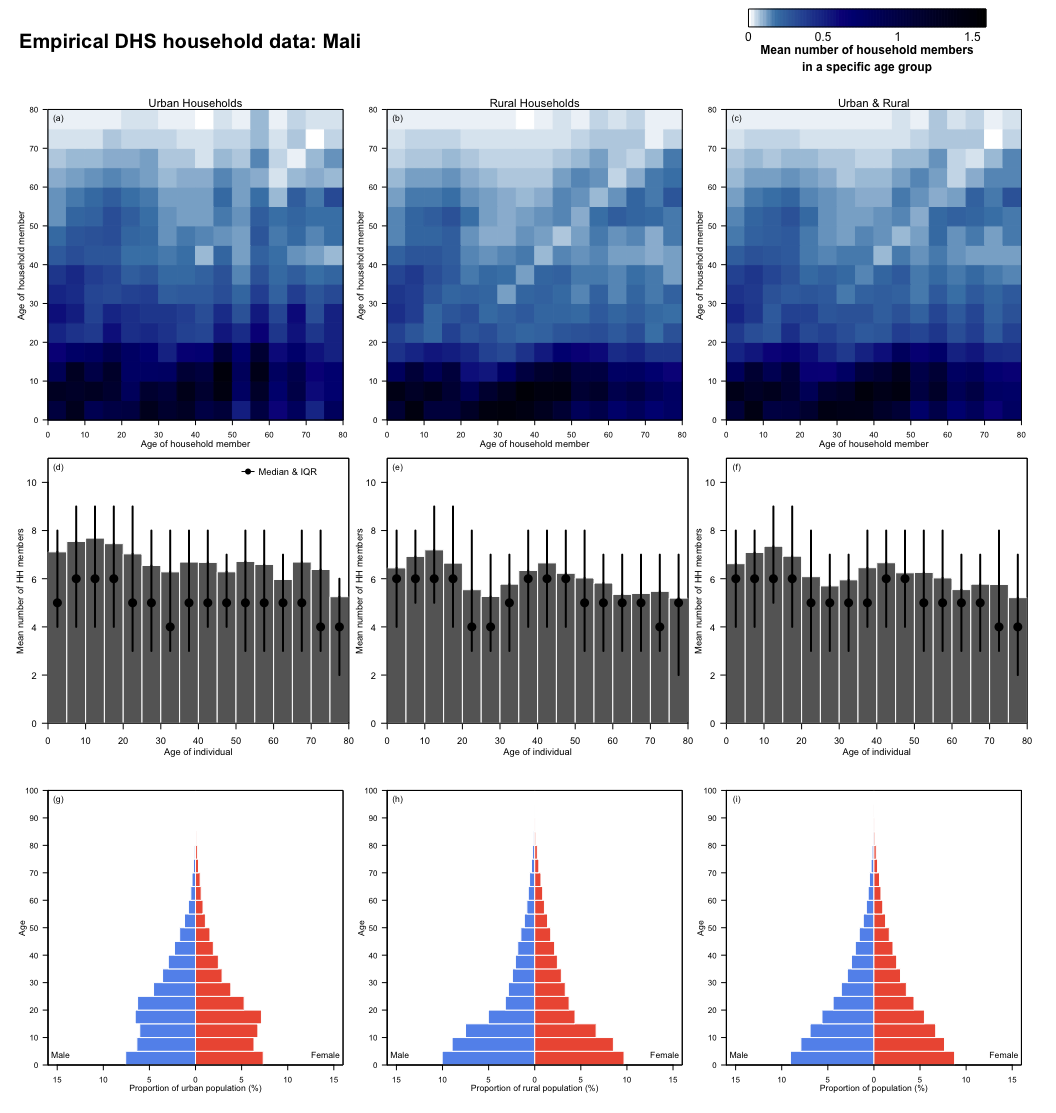

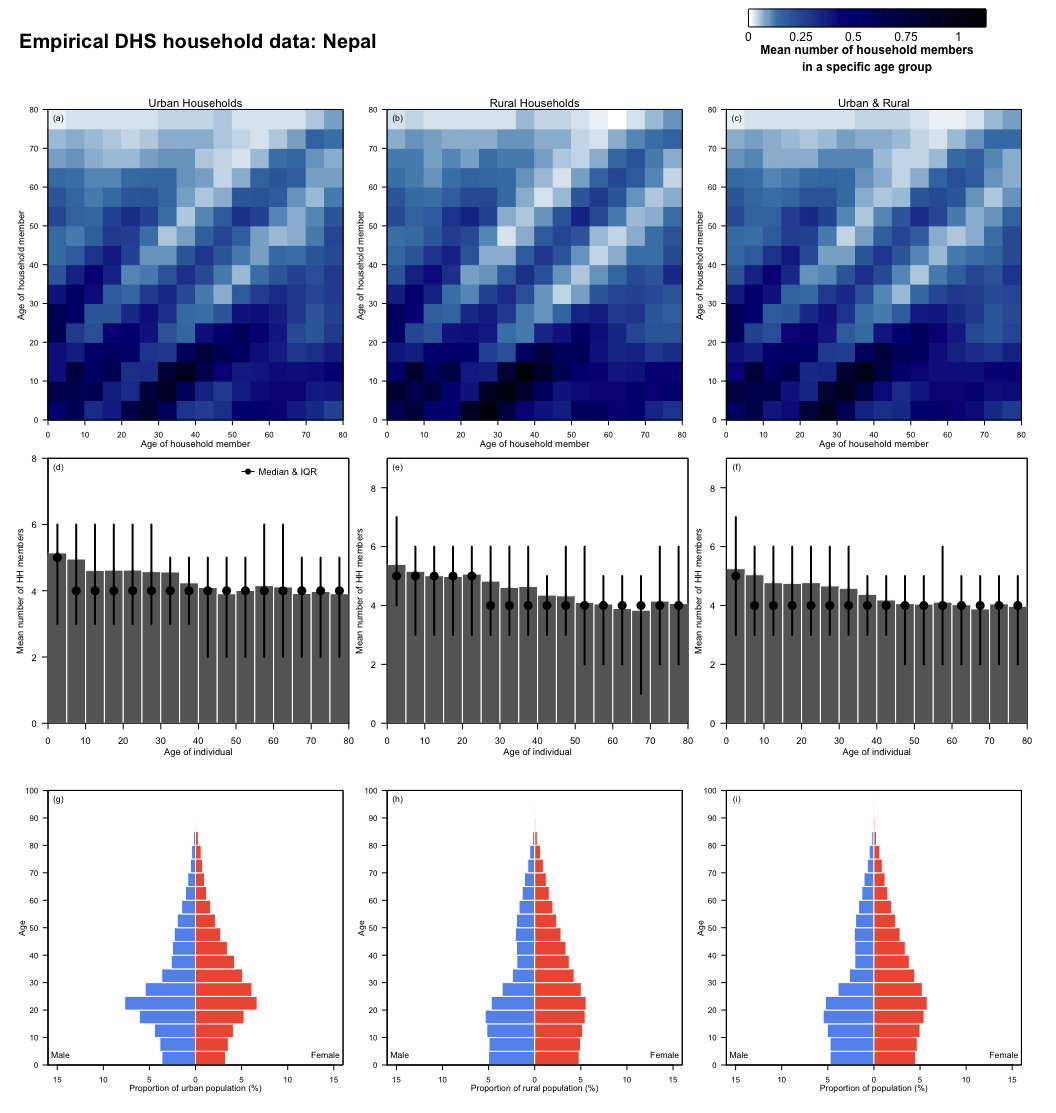

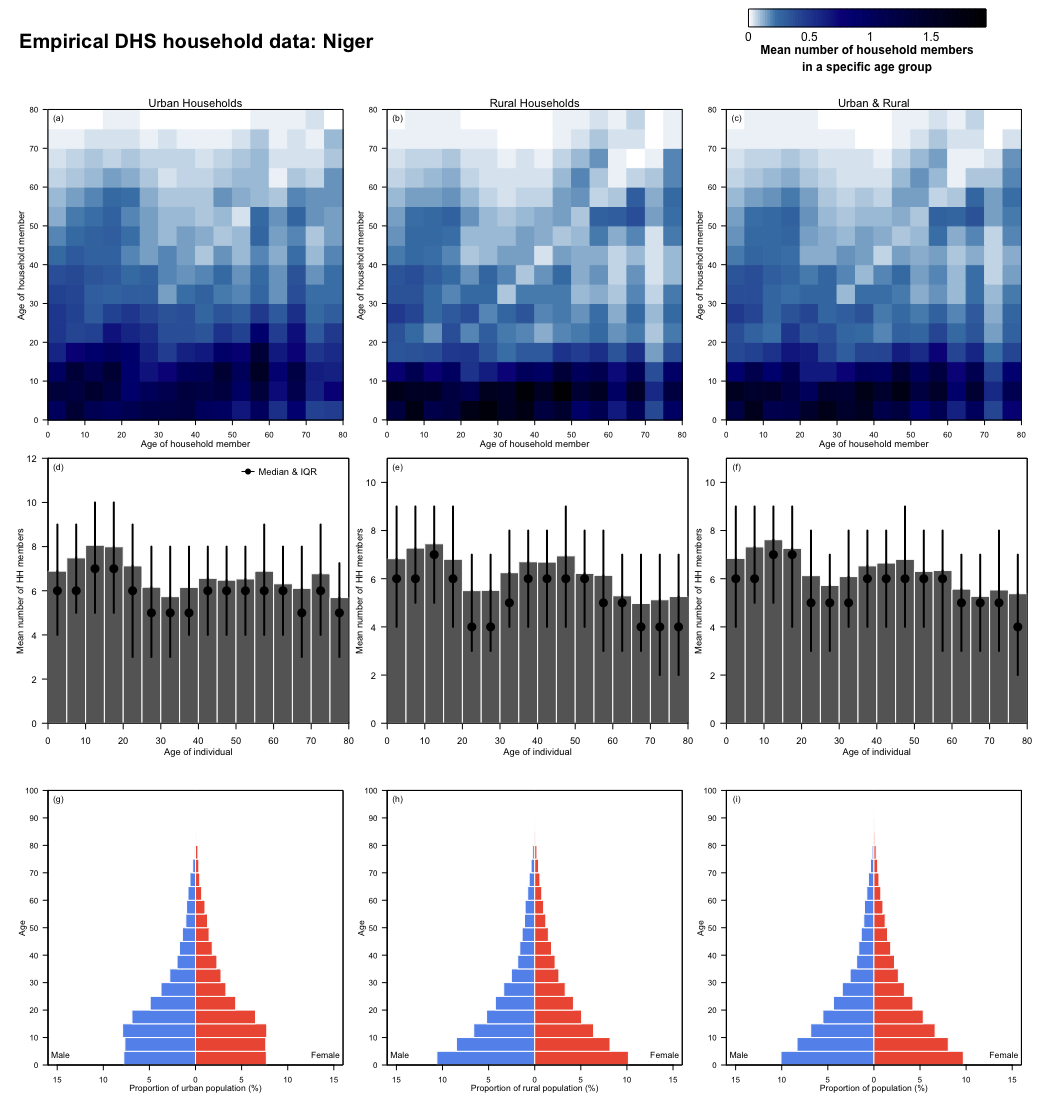

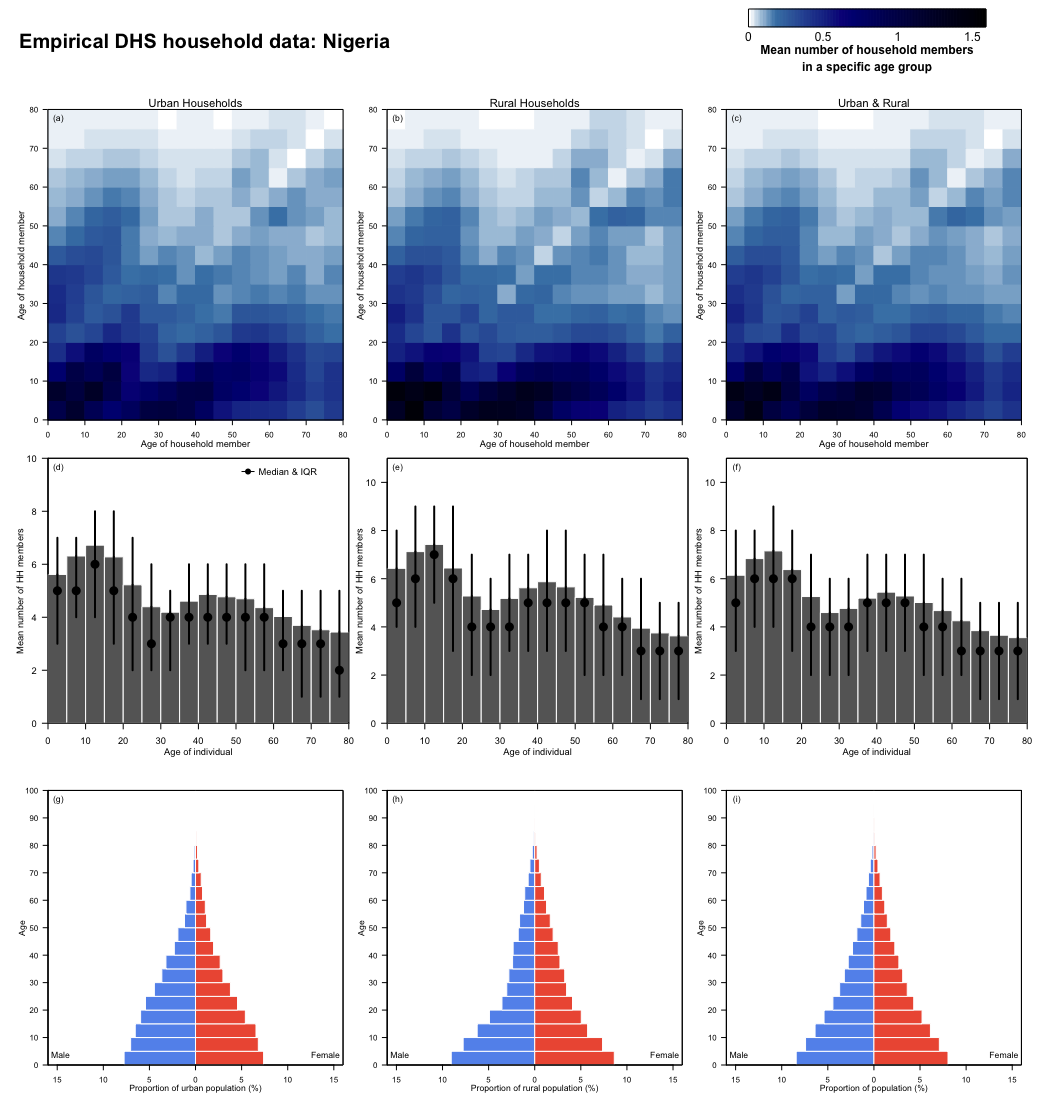

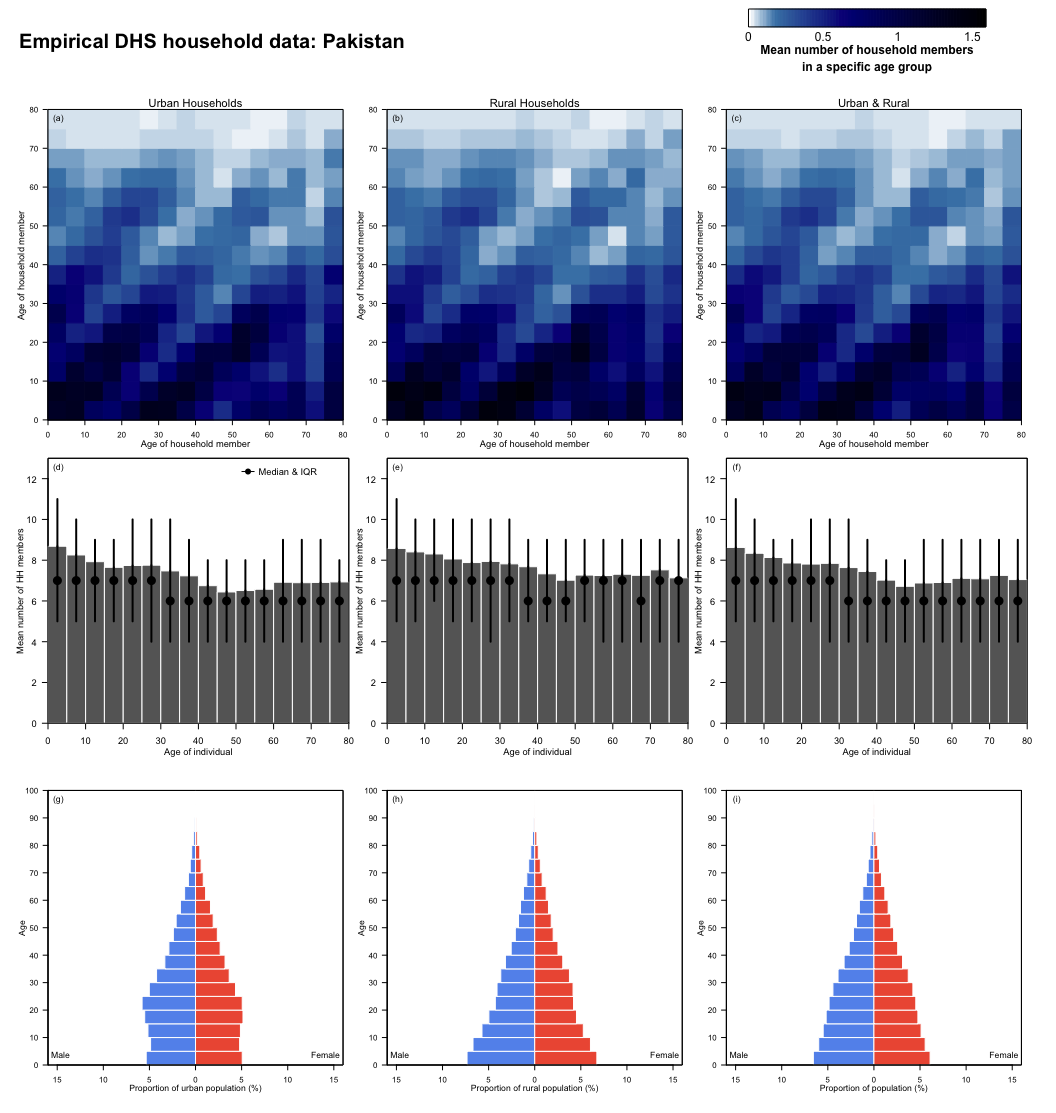

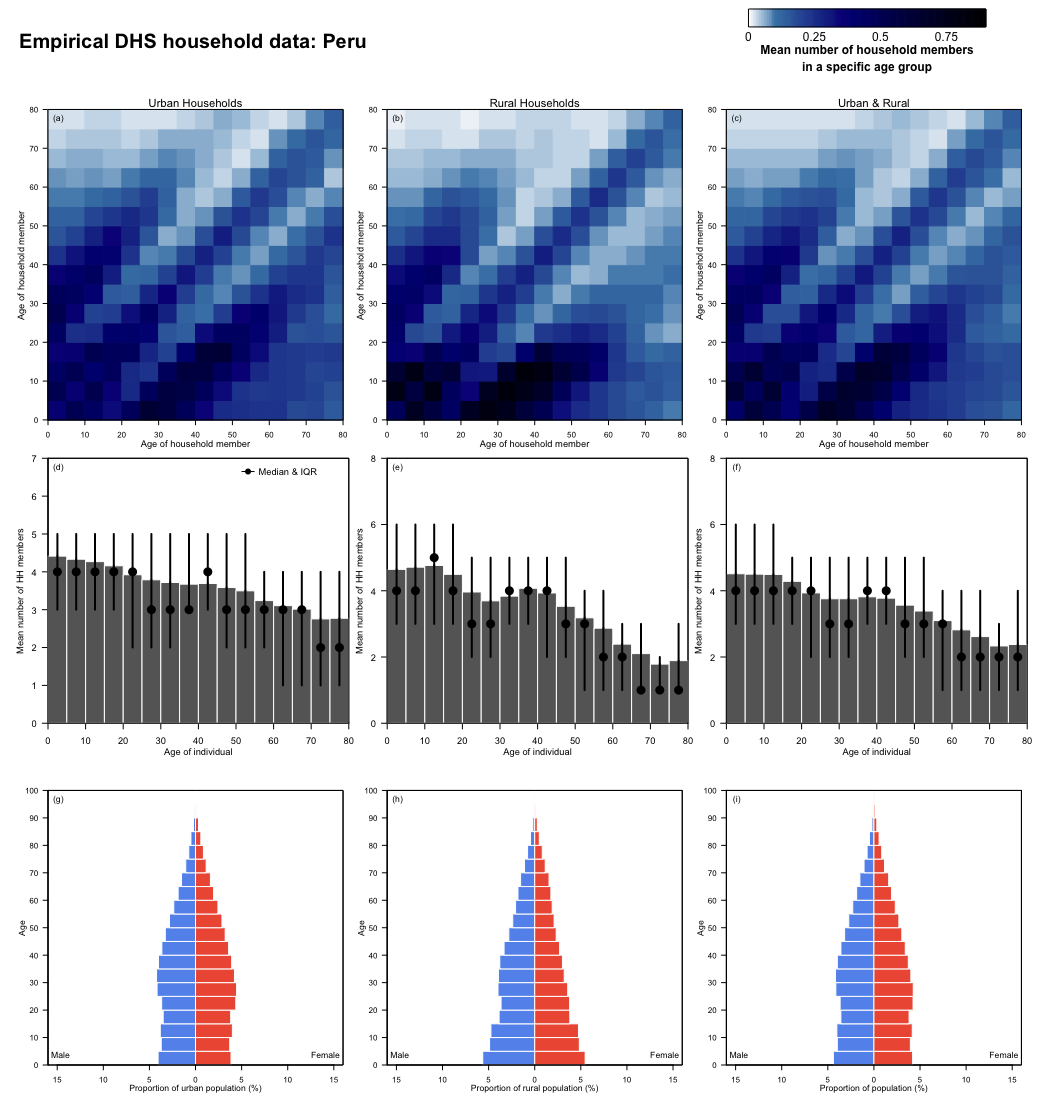

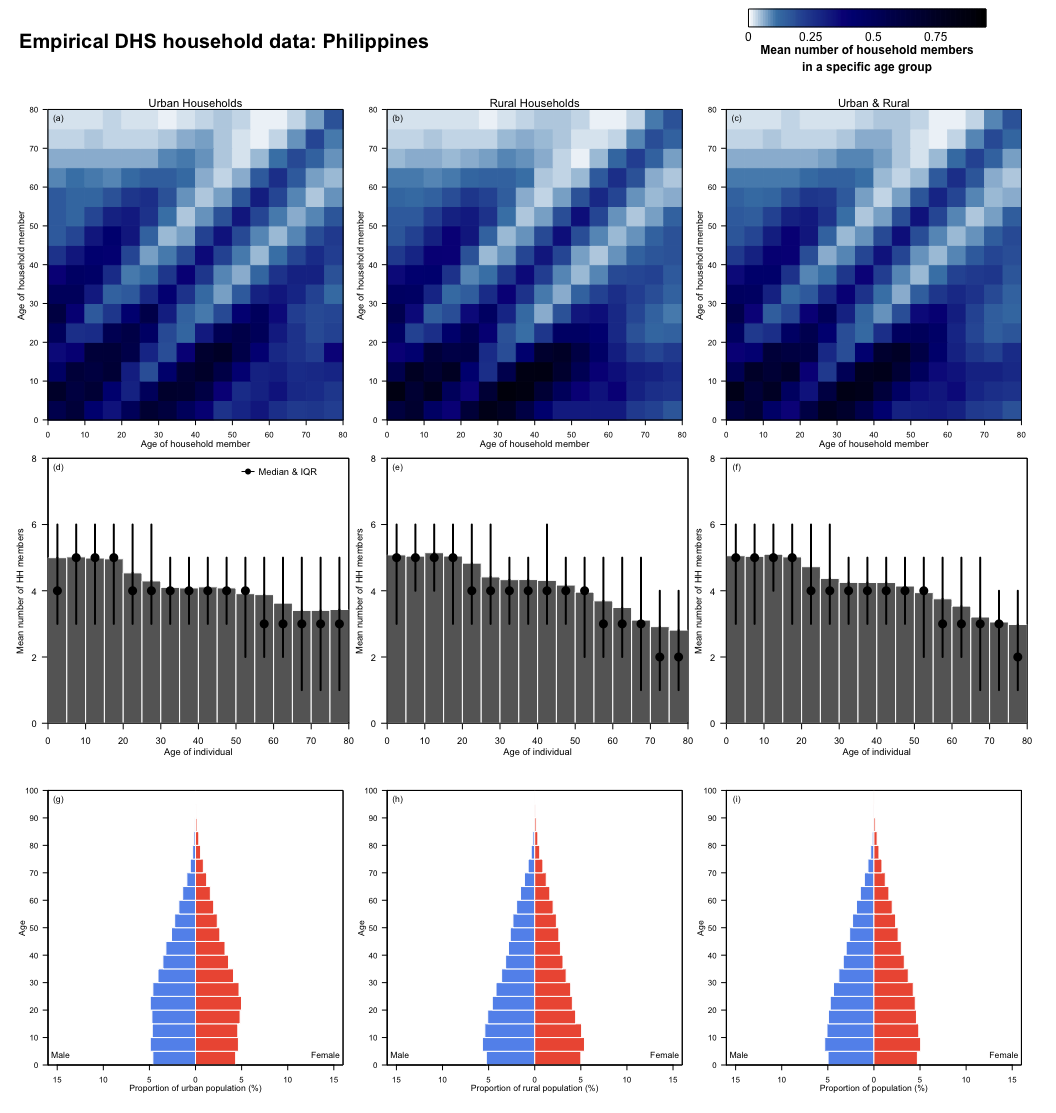

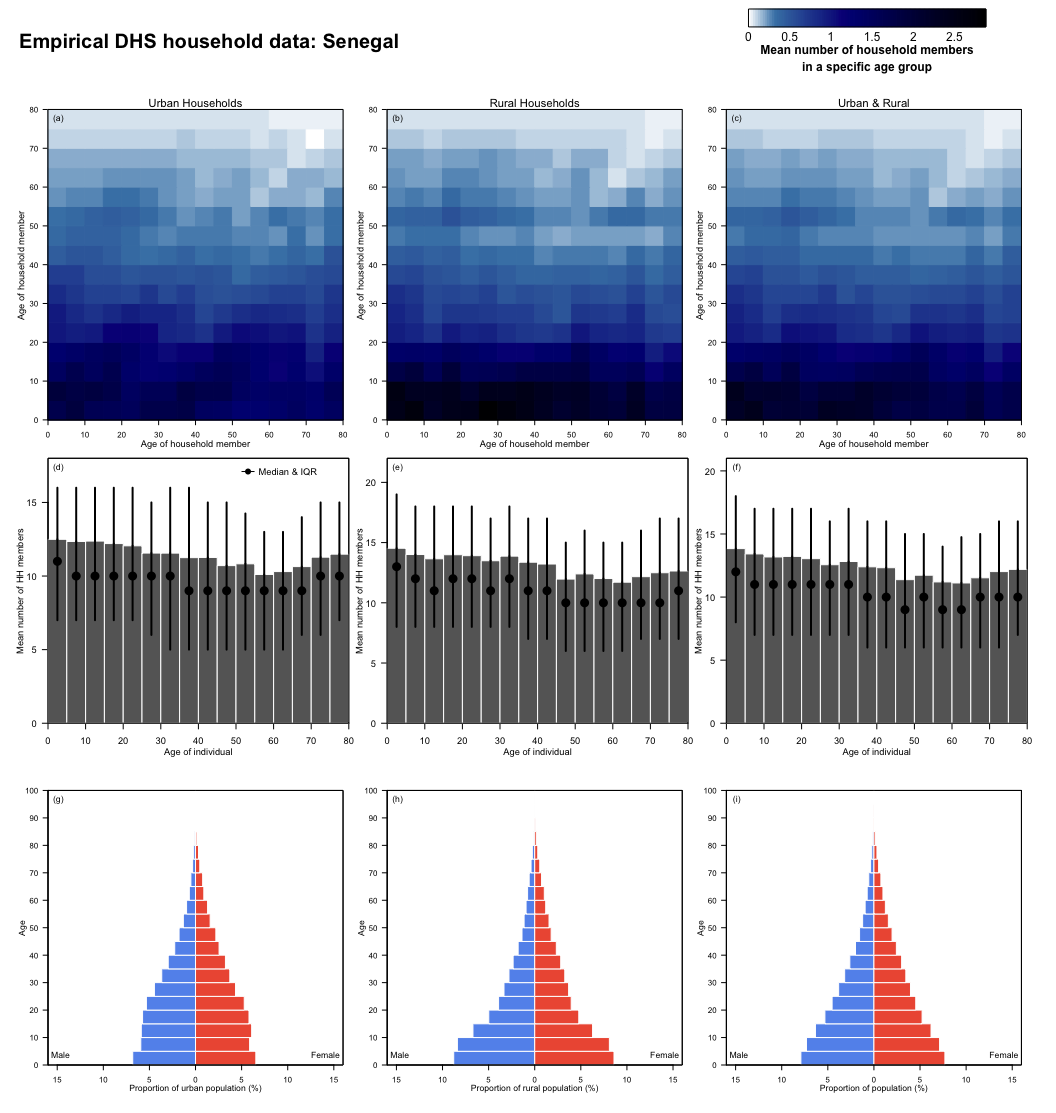

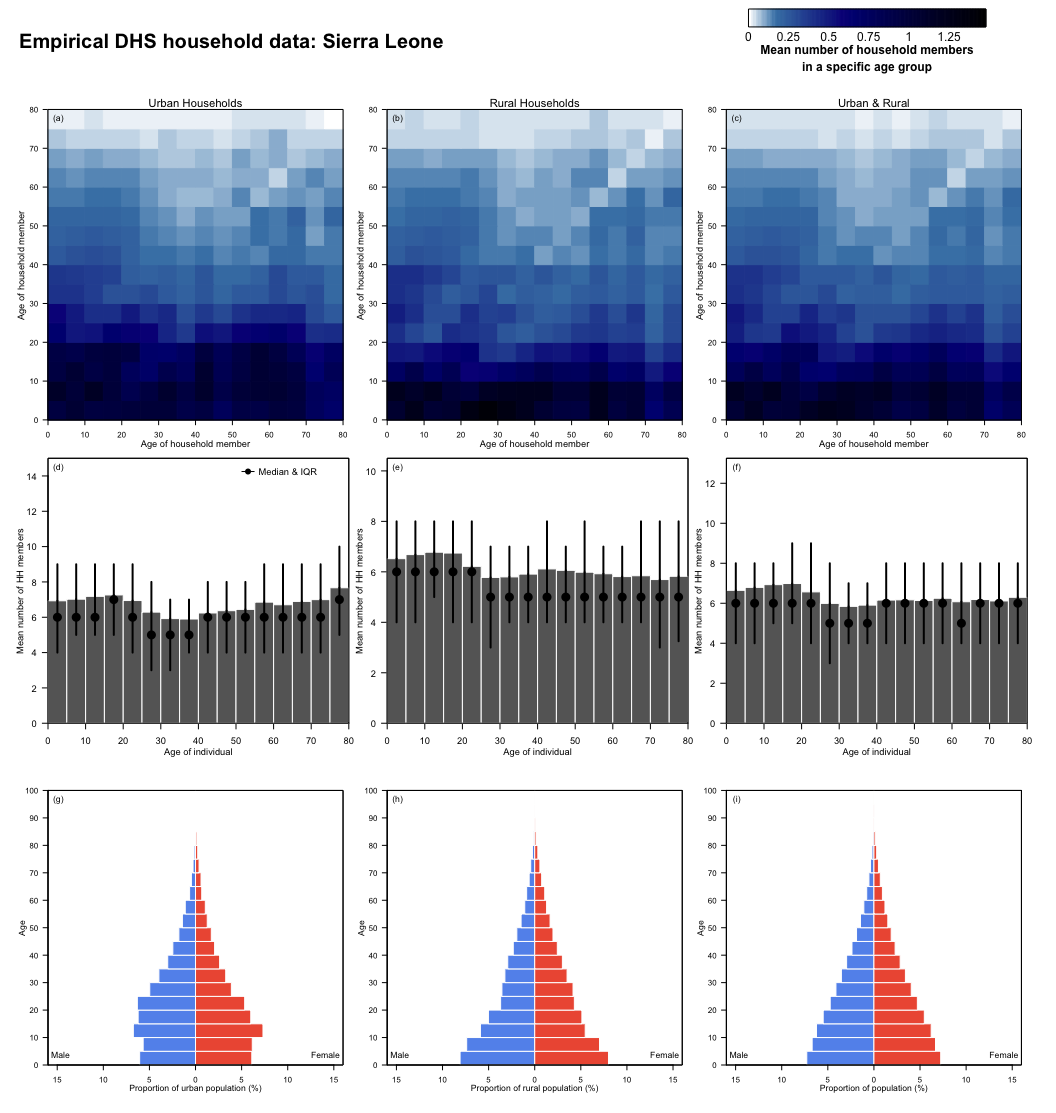

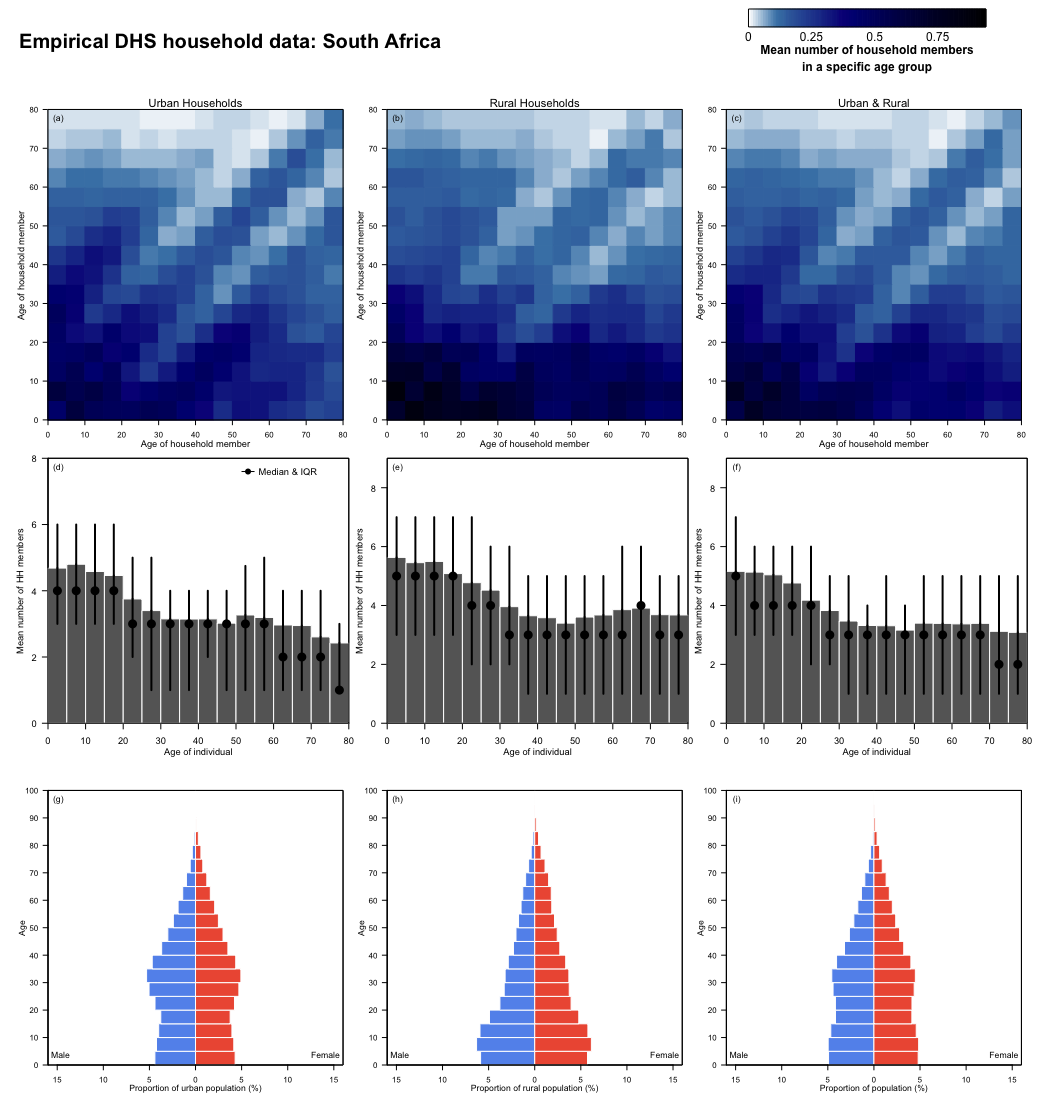

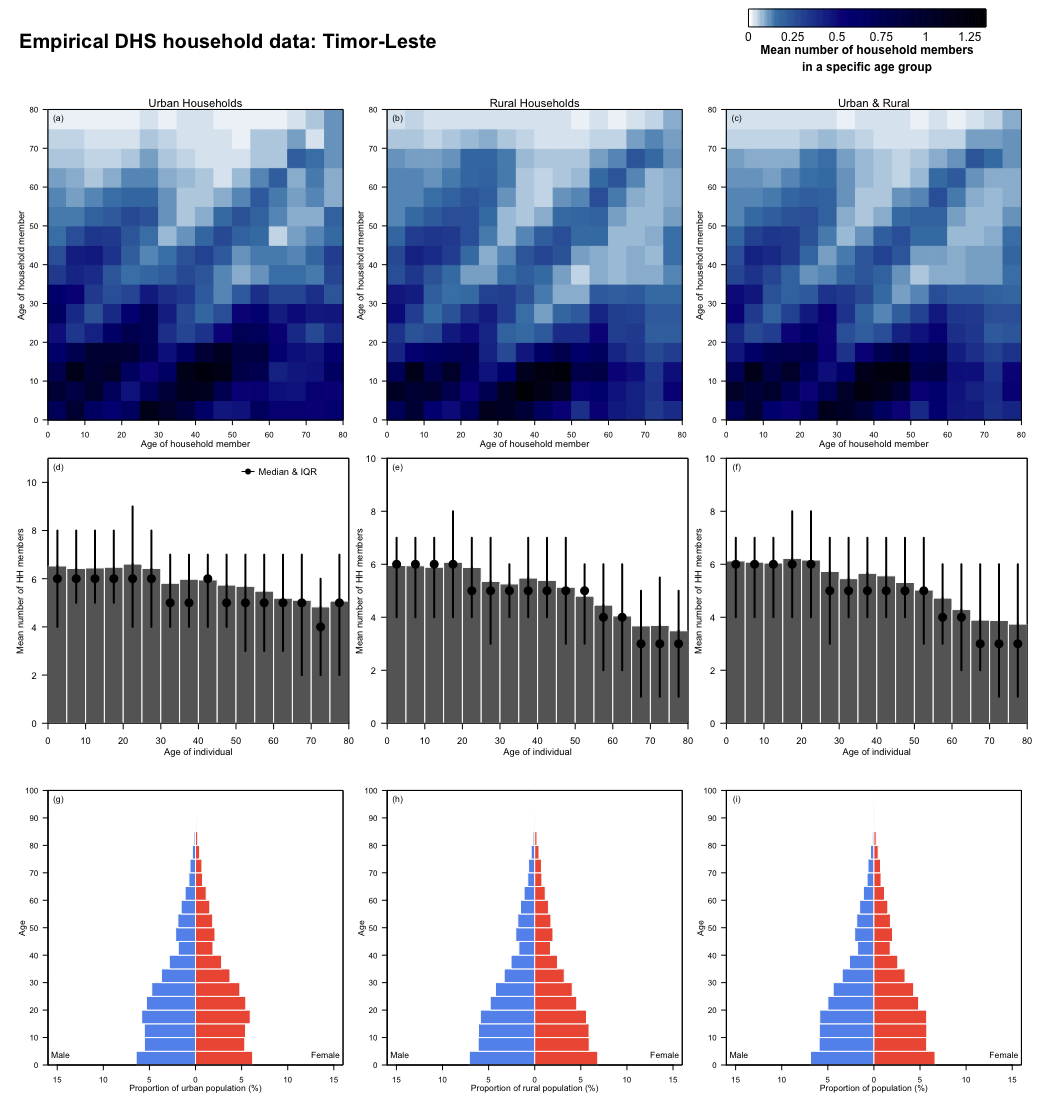

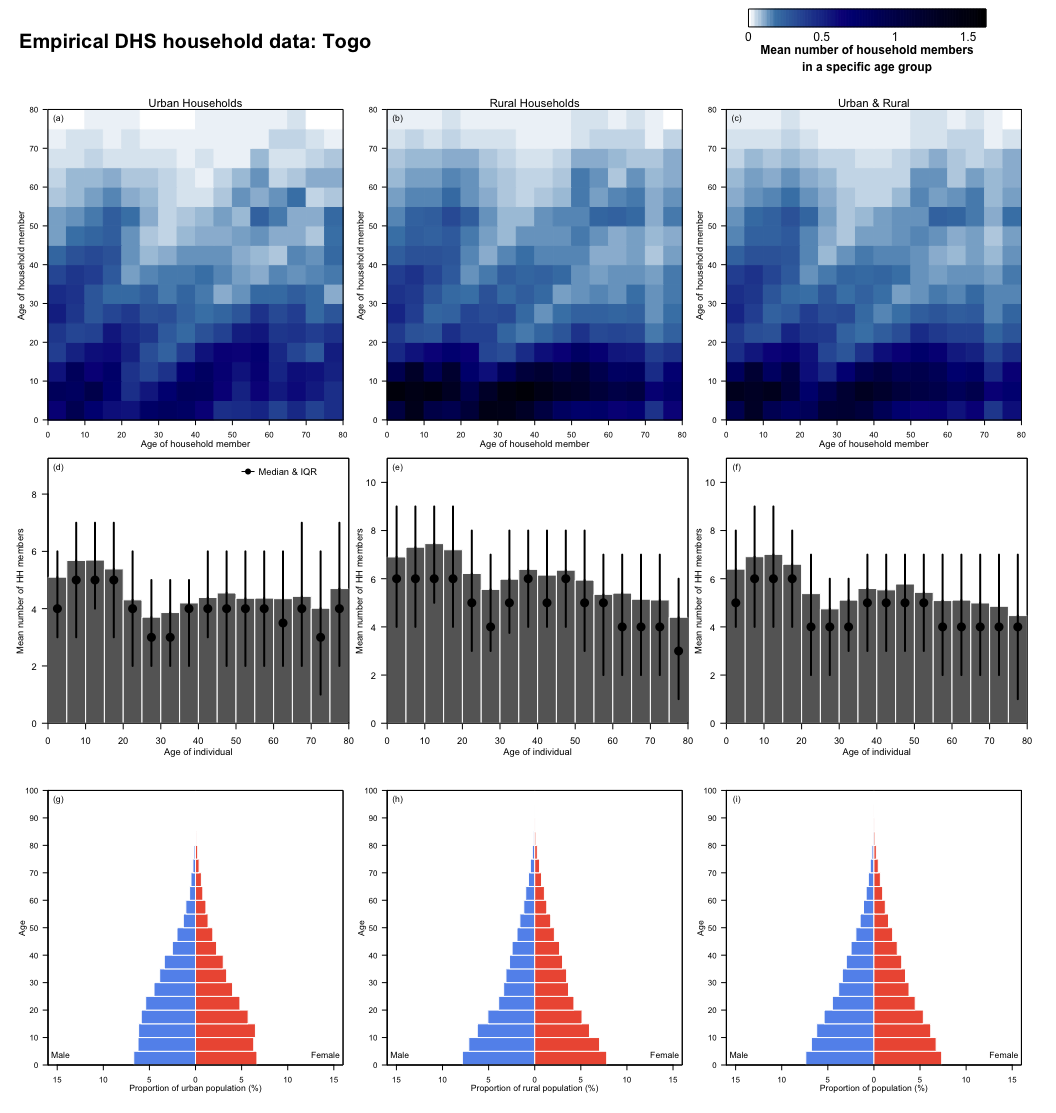

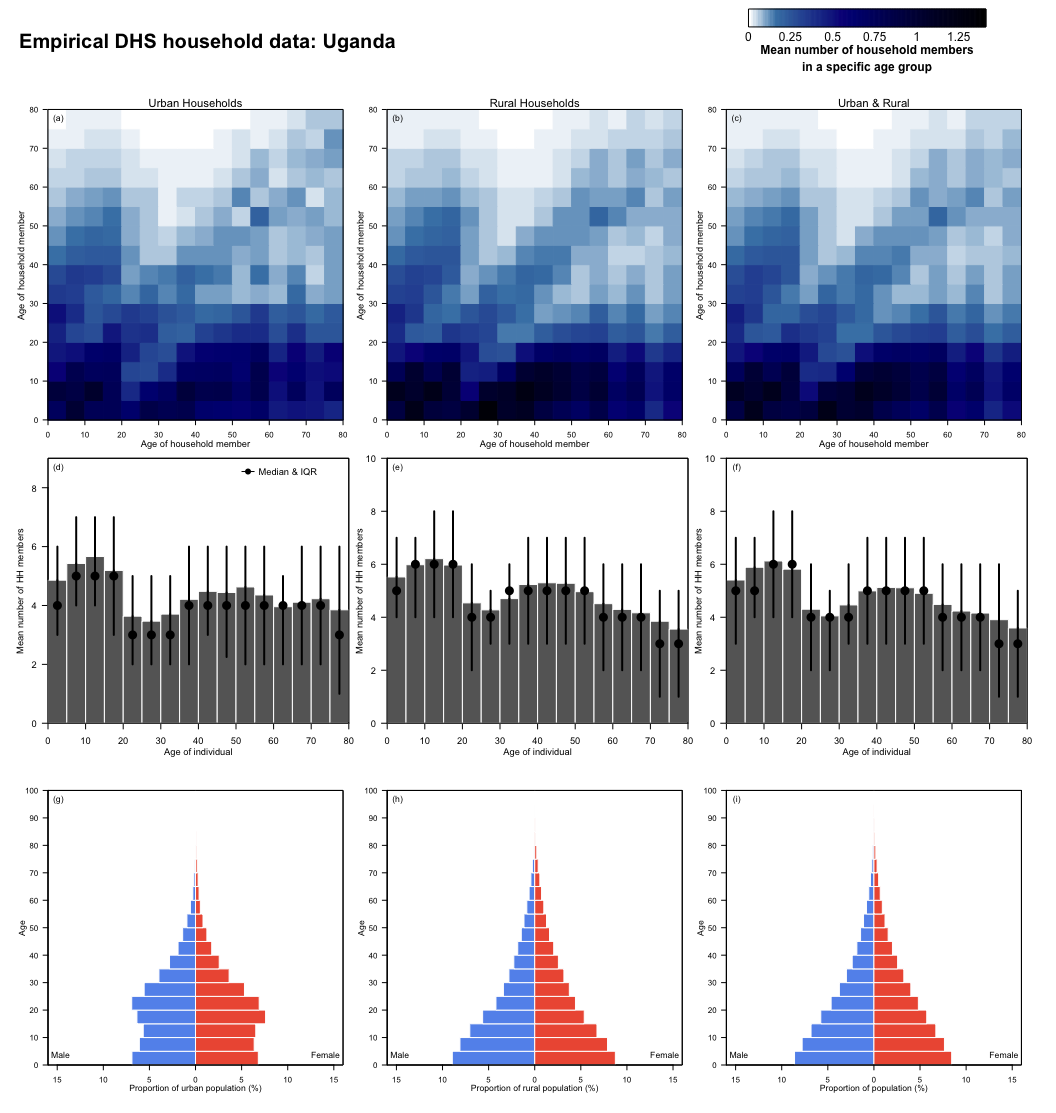

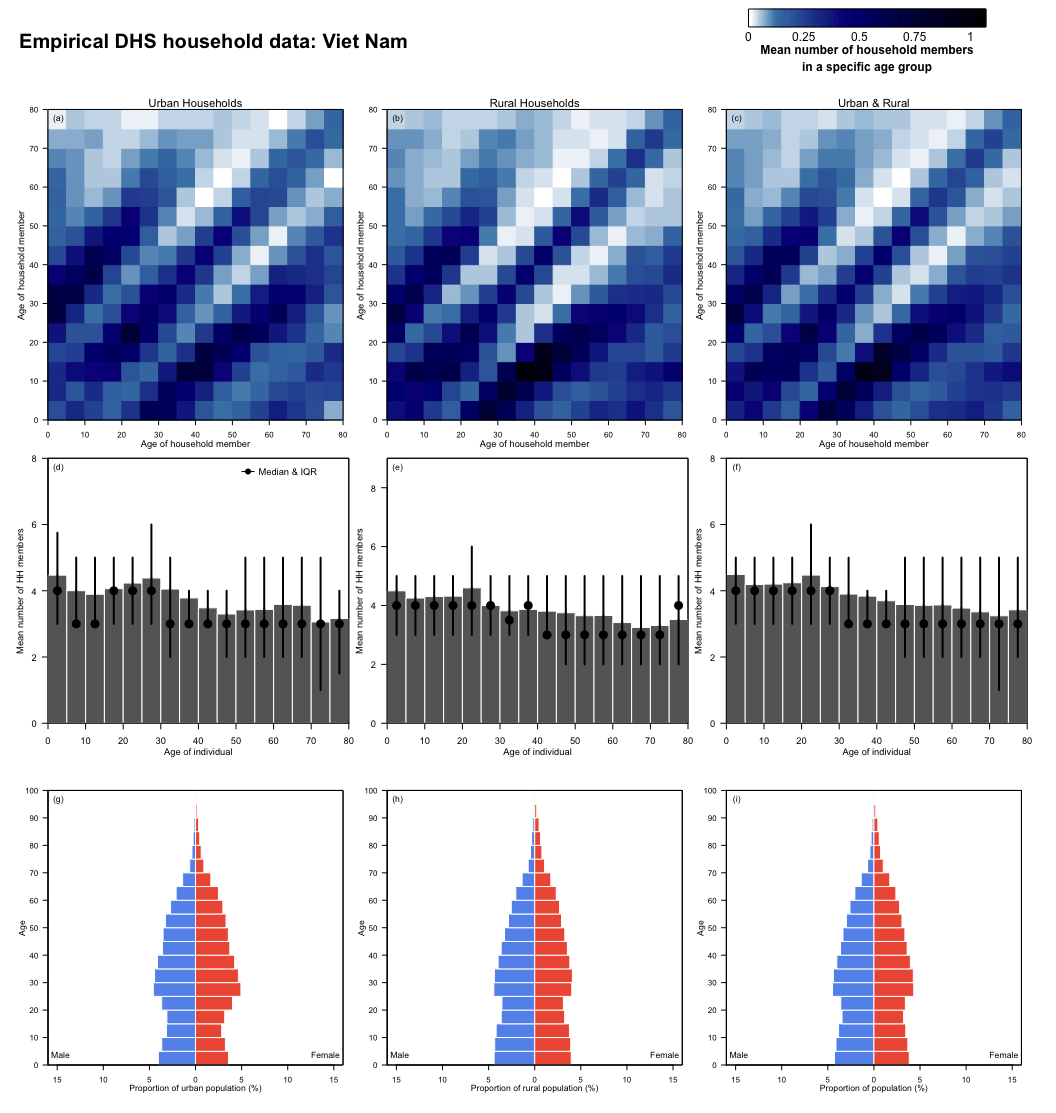

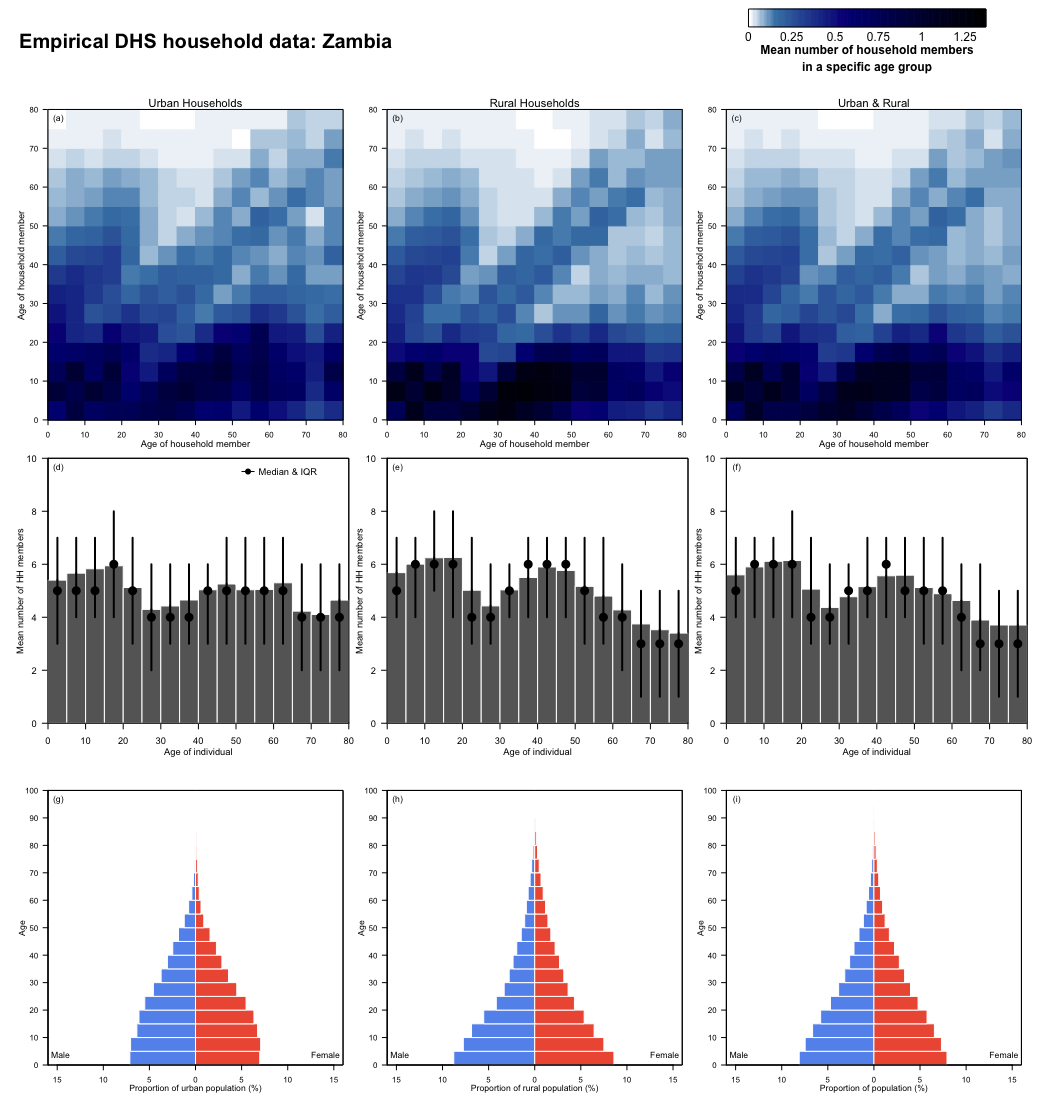

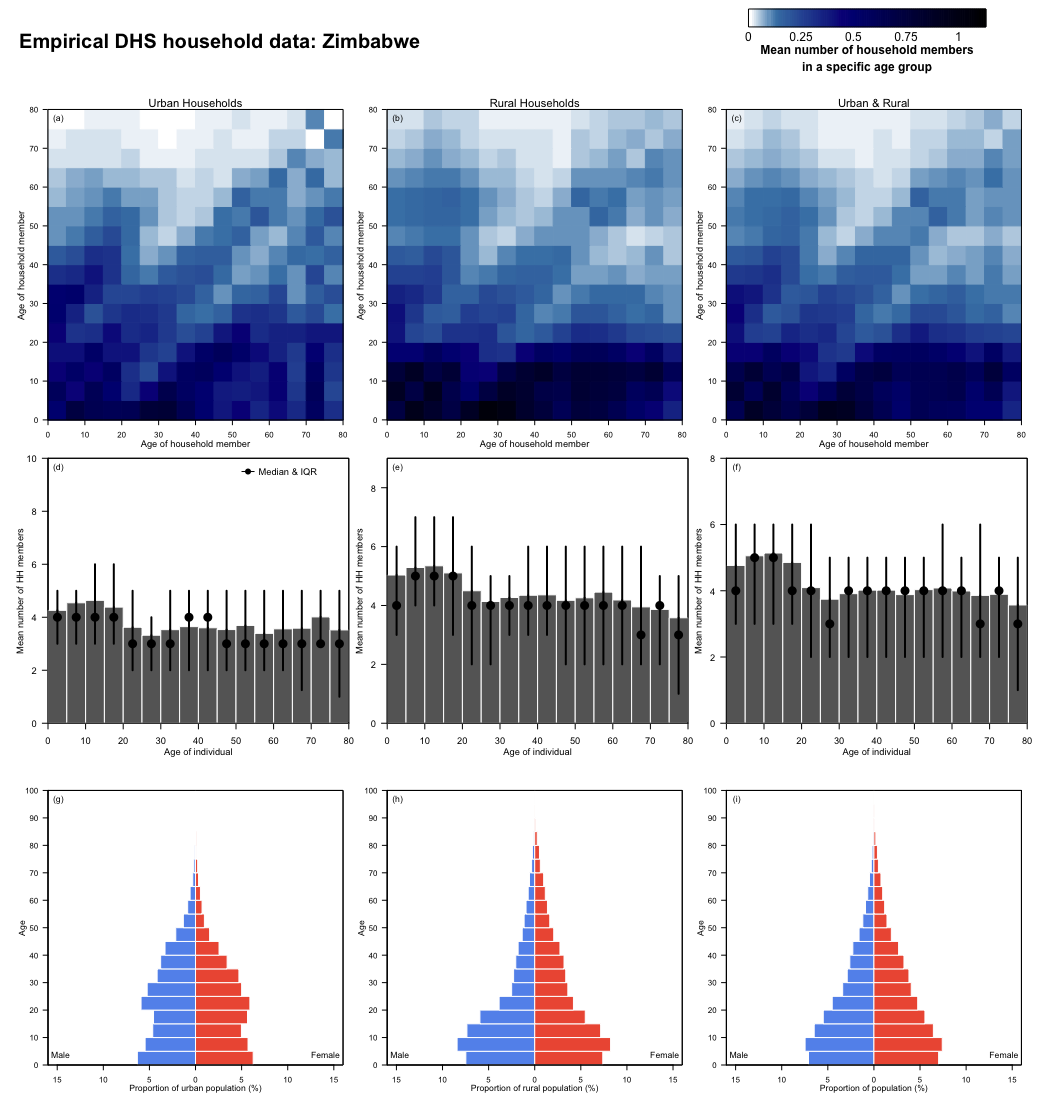


1. Correspondence to [mark.jit@lshtm.ac.uk](mailto:mark.jit@lshtm.ac.uk). [↑](#footnote-ref-1)
