# Supplementary material for: Projecting contact matrices in 177 geographical regions: An update and comparison with empirical data for the COVID-19 era
Source: PLoS Comput Biol. 2021 Jul 26;17(7):e1009098. doi: 10.1371/journal.pcbi.1009098 (PMC8354454; doi:10.1371/journal.pcbi.1009098)
Supplement: S3 Text — (DOCX) [file pcbi.1009098.s003.docx]

Projecting contact matrices in 177 geographical regions: an update and comparison with empirical data for the COVID-19 era

Supplementary Material

Kiesha Prem, Kevin van Zandvoort, Petra Klepac, Rosalind M Eggo, Nicholas G Davies,

Centre for the Mathematical Modelling of Infectious Diseases COVID-19 Working Group,

Alex R Cook, Mark Jit^[[1]](#footnote-1)^

Contents

[B.3. Updated age- and location-specific contact matrices 1](#_Toc144640638)

## Updated age- and location-specific contact matrices

The 2017 synthetic matrices provide validated approximations to age-and-location-specific contact matrices for 152 geographical regions. We have therefore updated these matrices with the most recent data (Demographic Household Surveys, World Bank and UN Population Division) extending the coverage to 177 geographical locations, covering 97.2% of the world’s population.


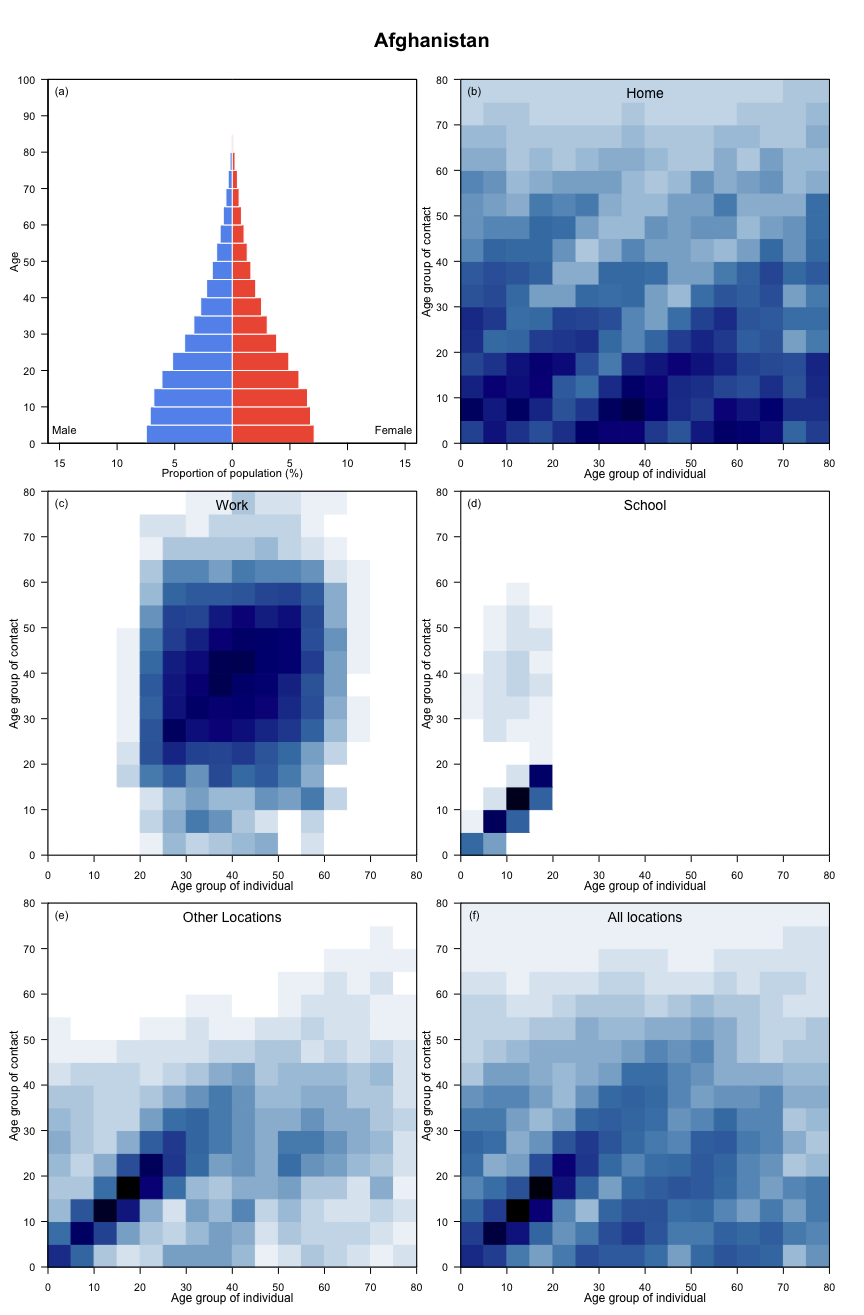

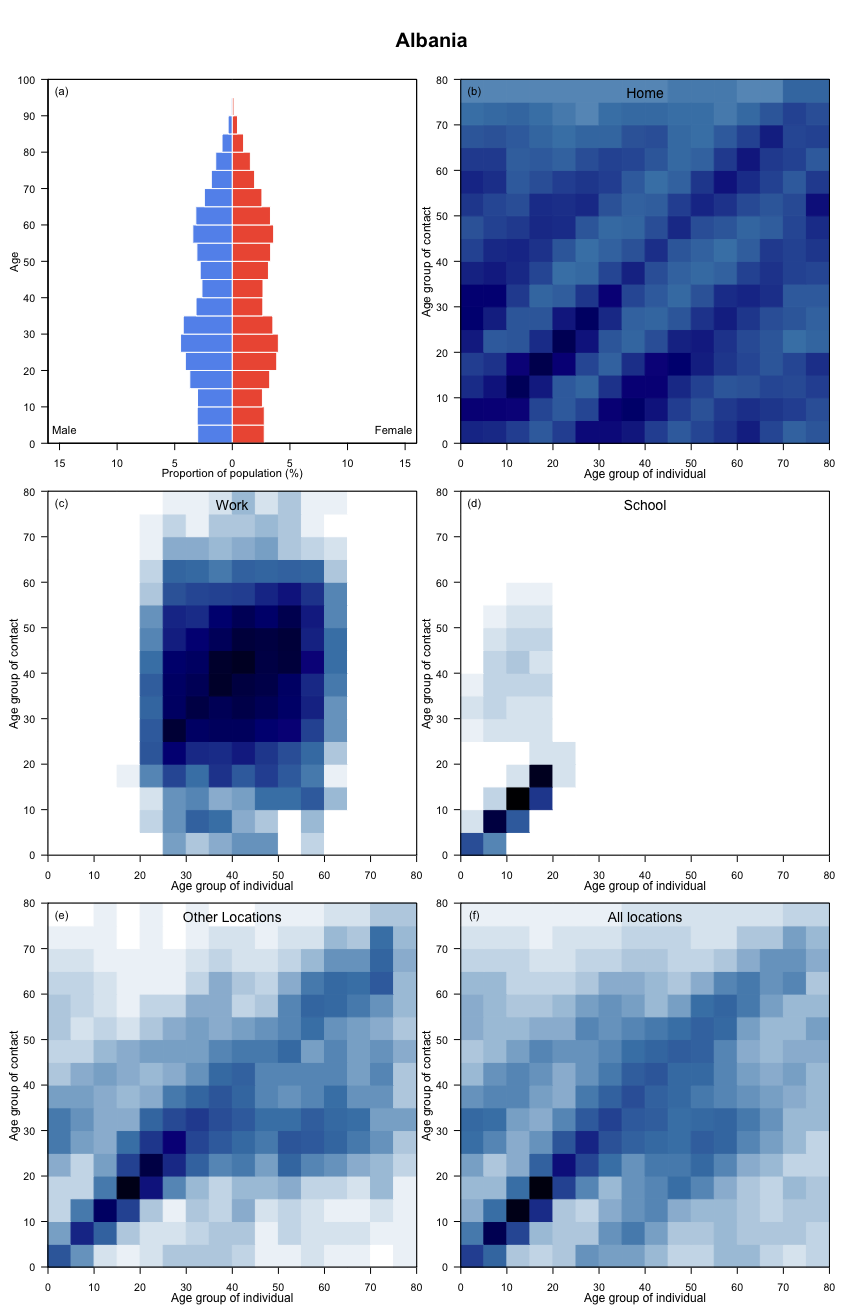

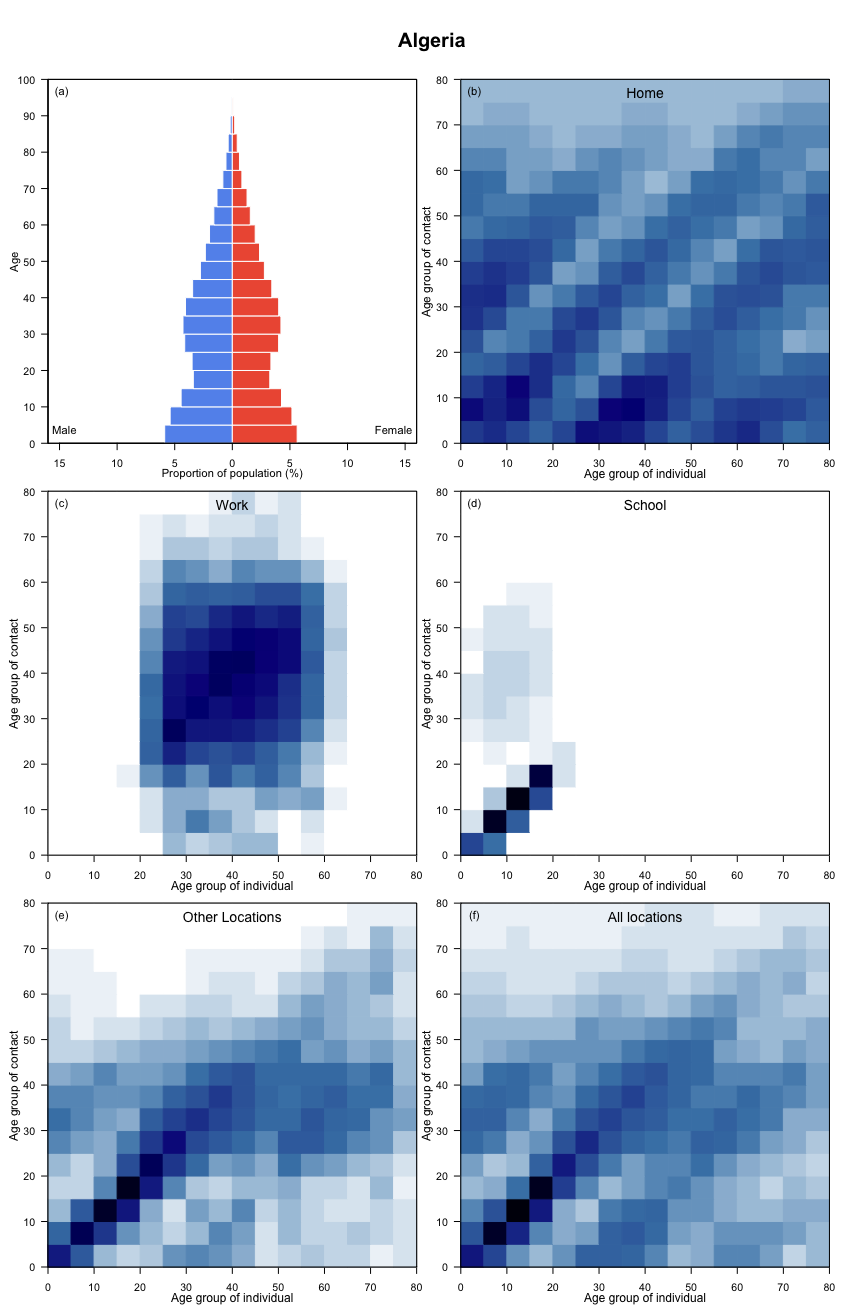

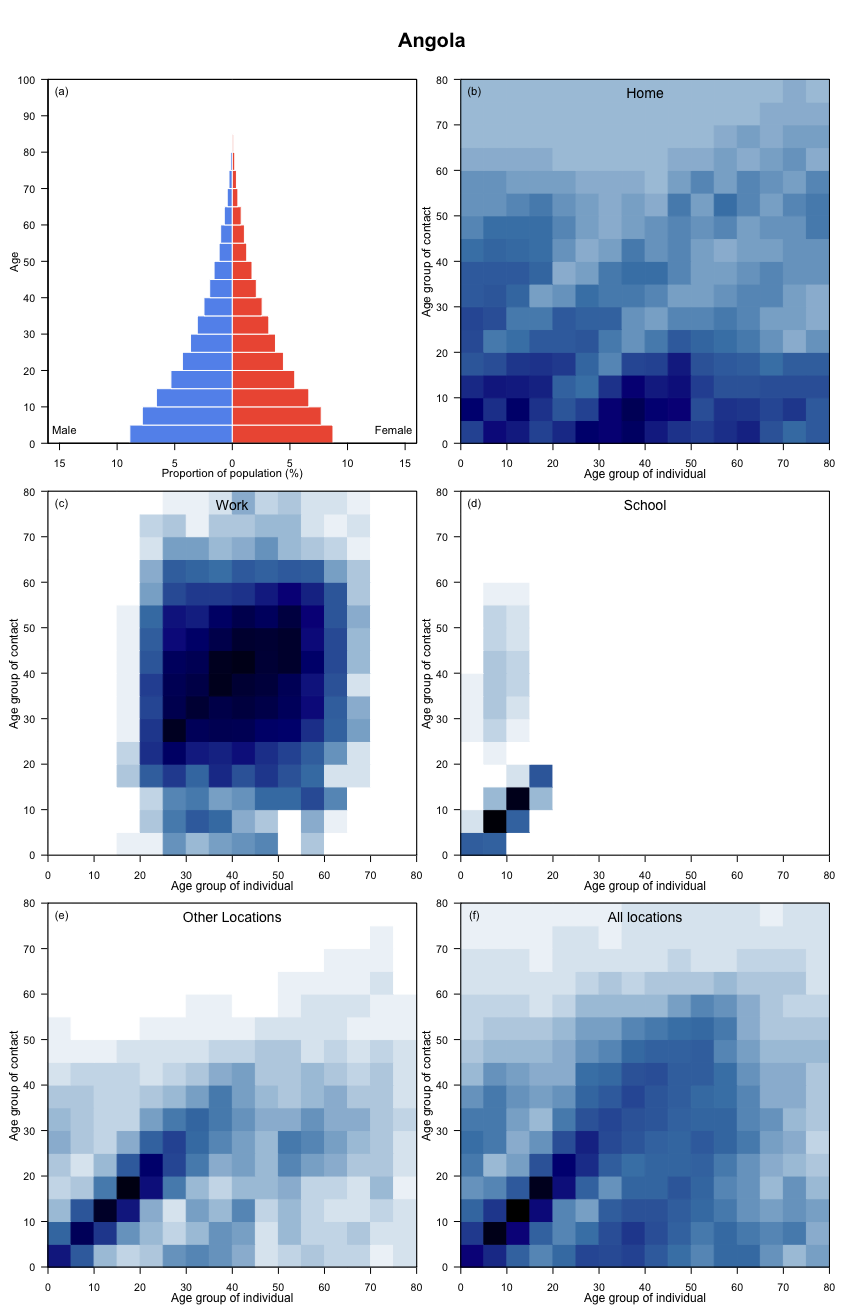

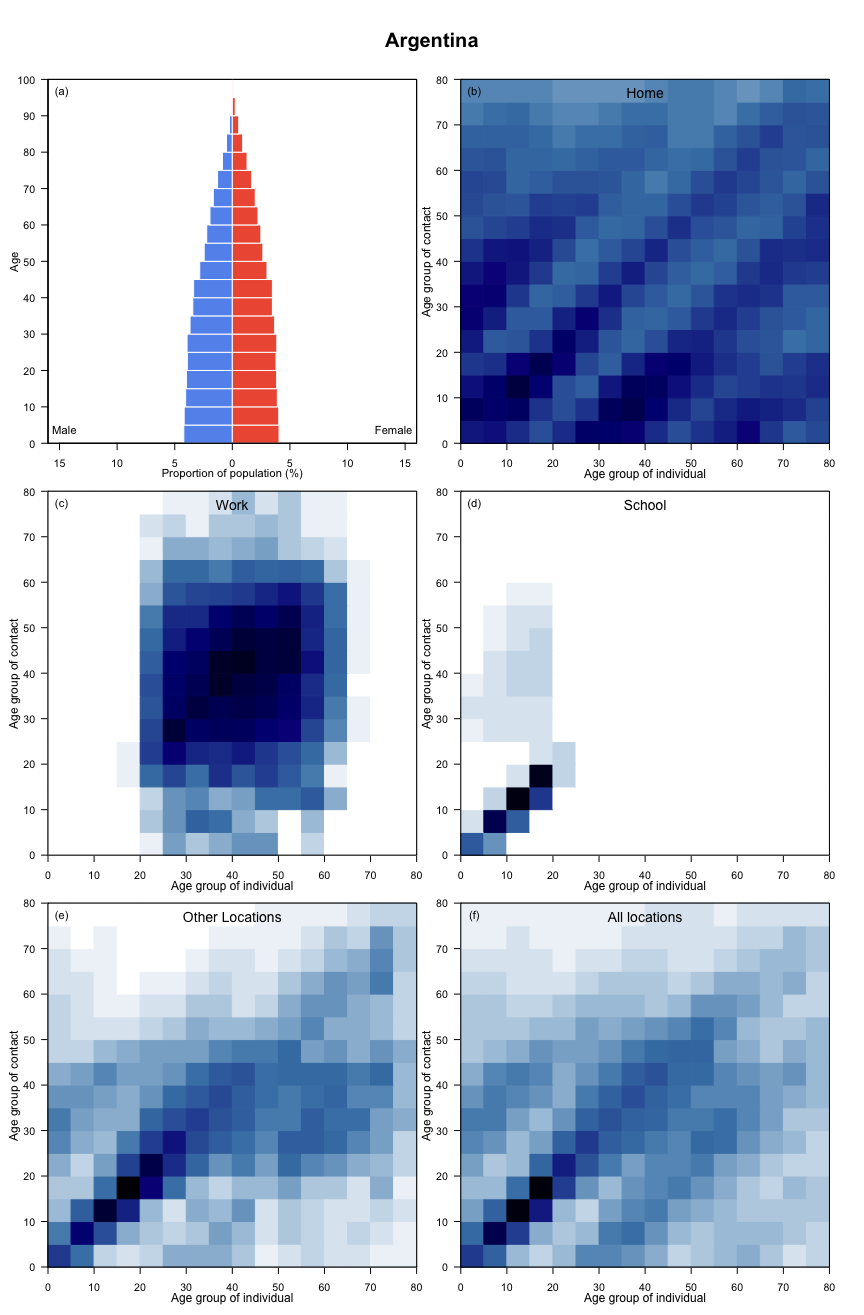

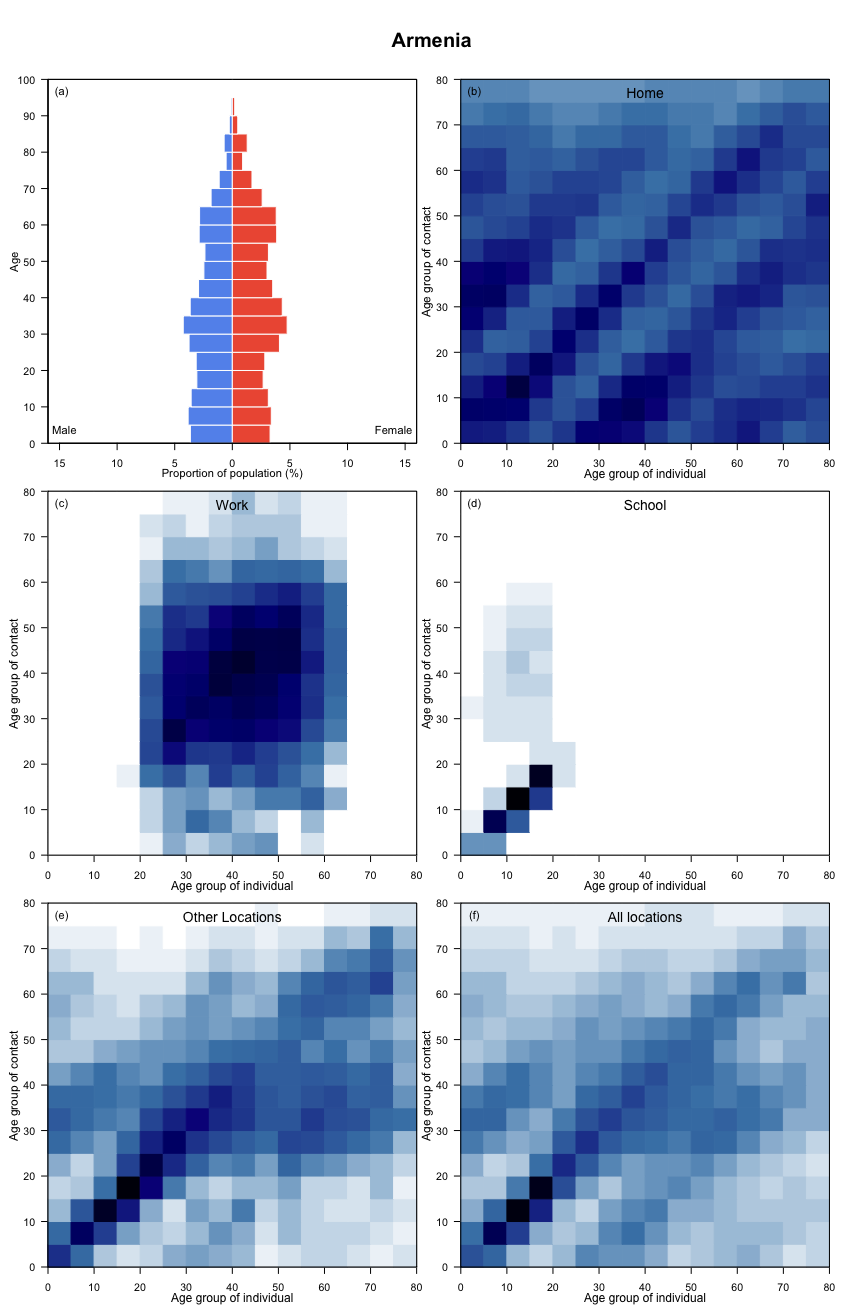

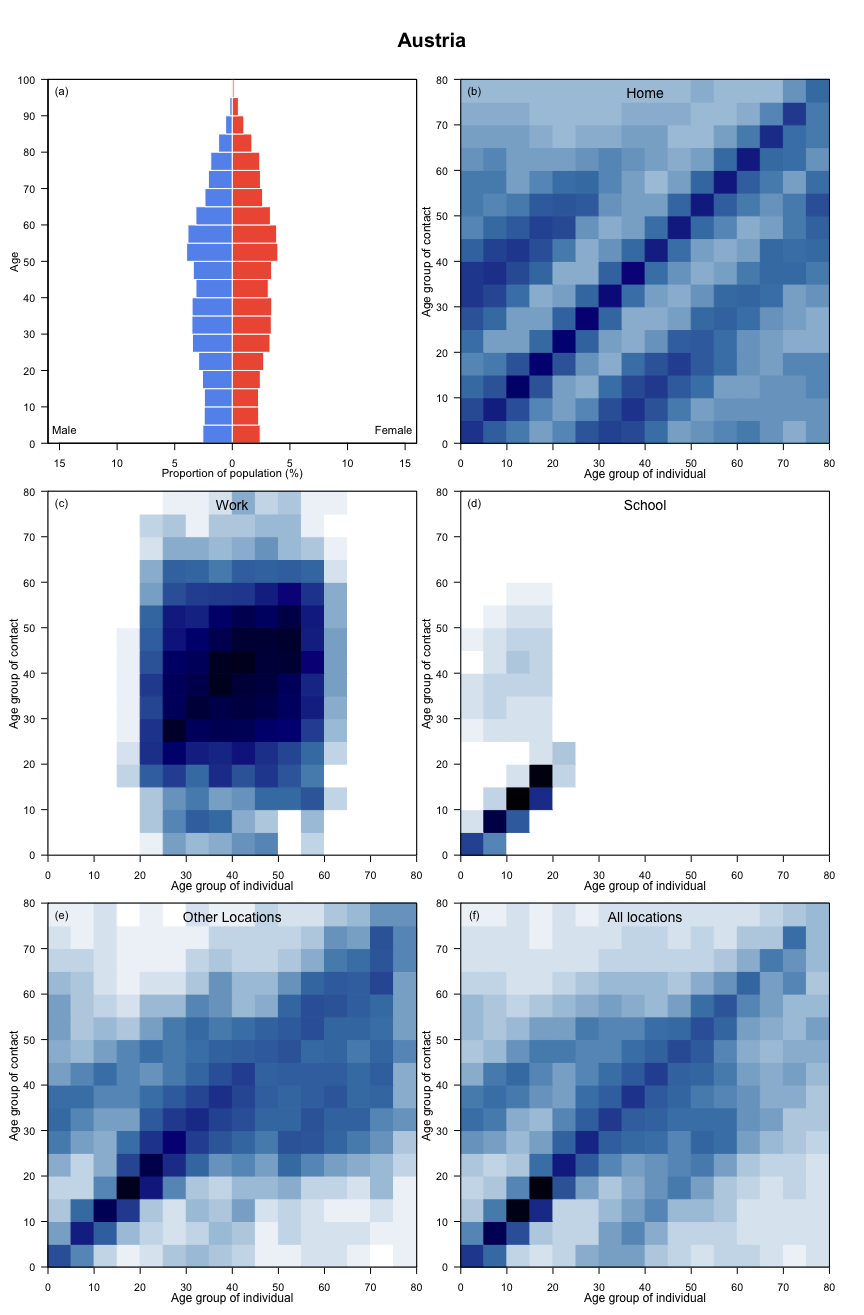

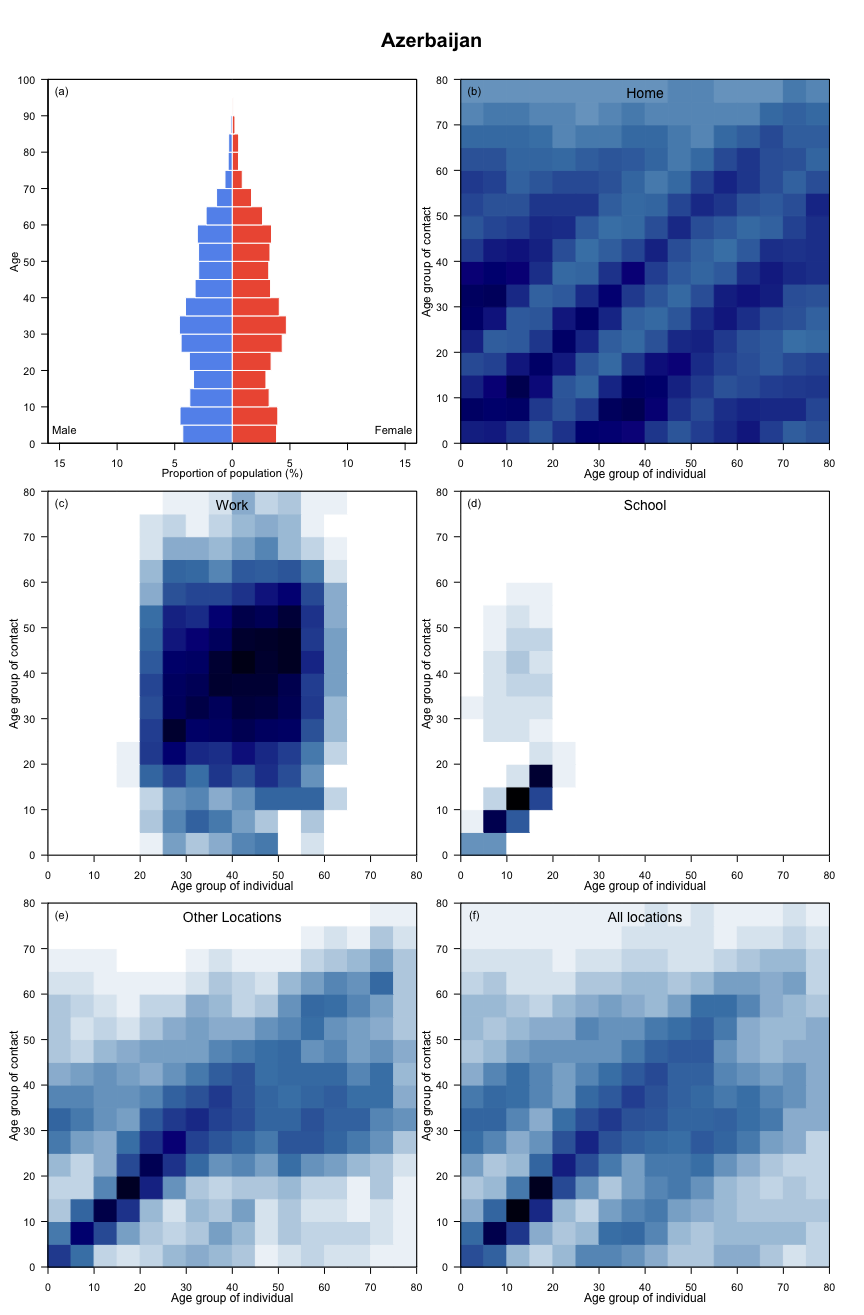

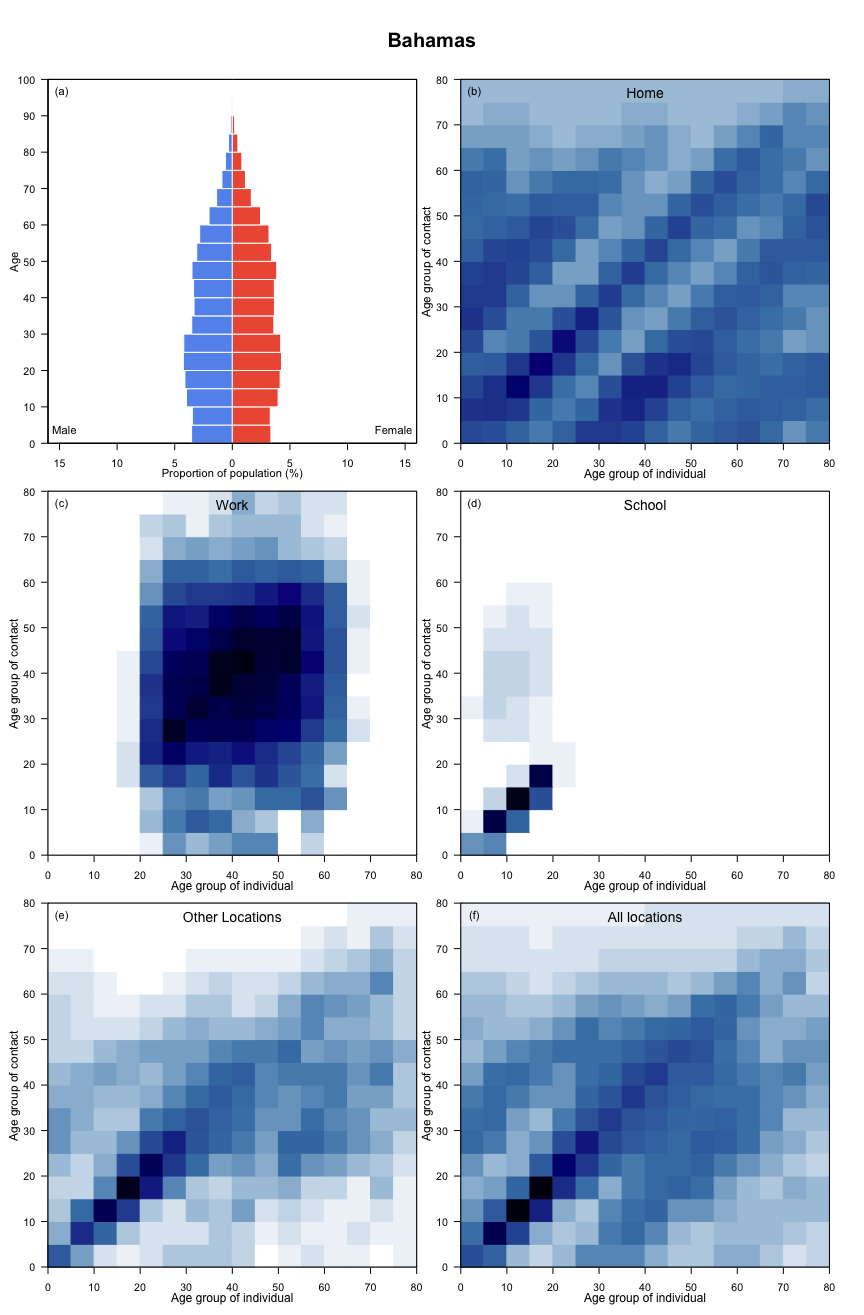

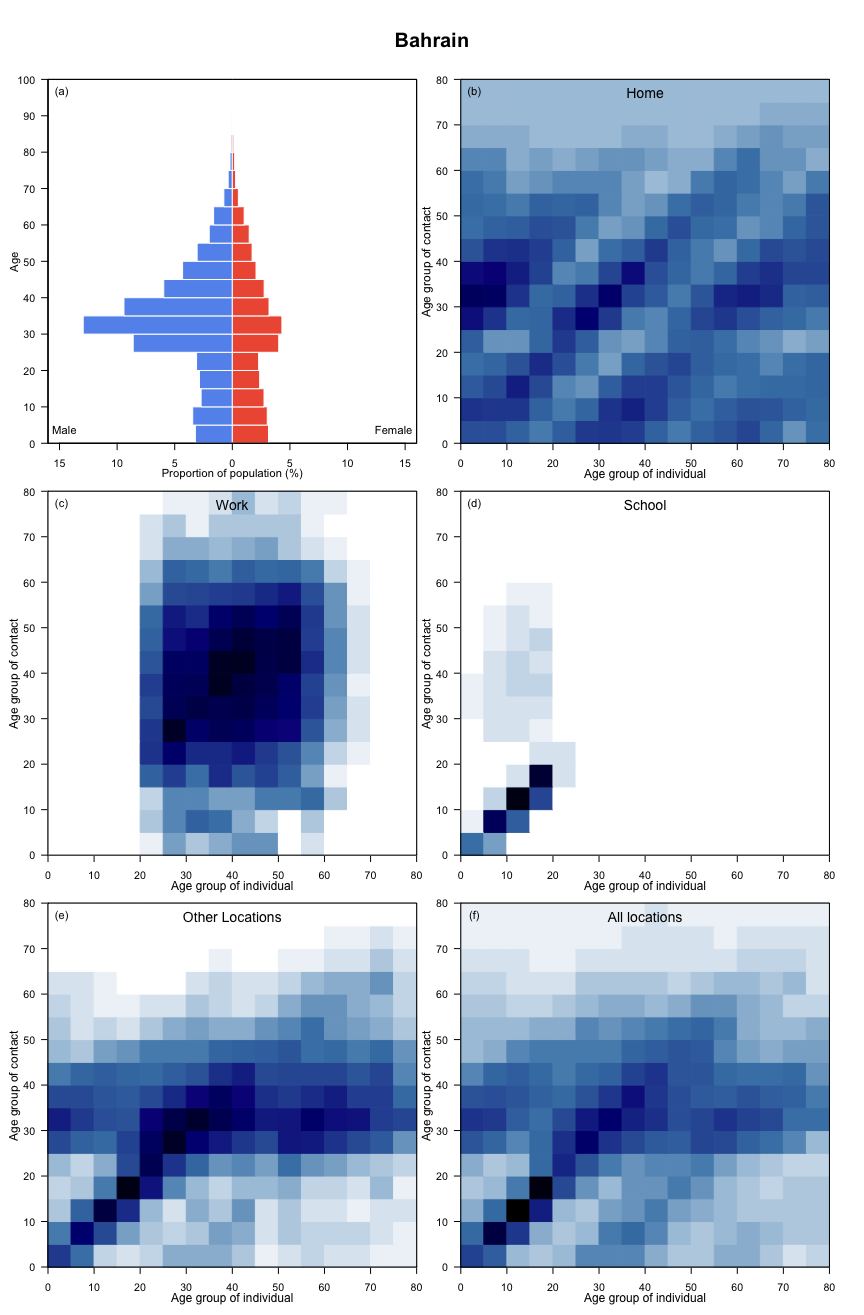

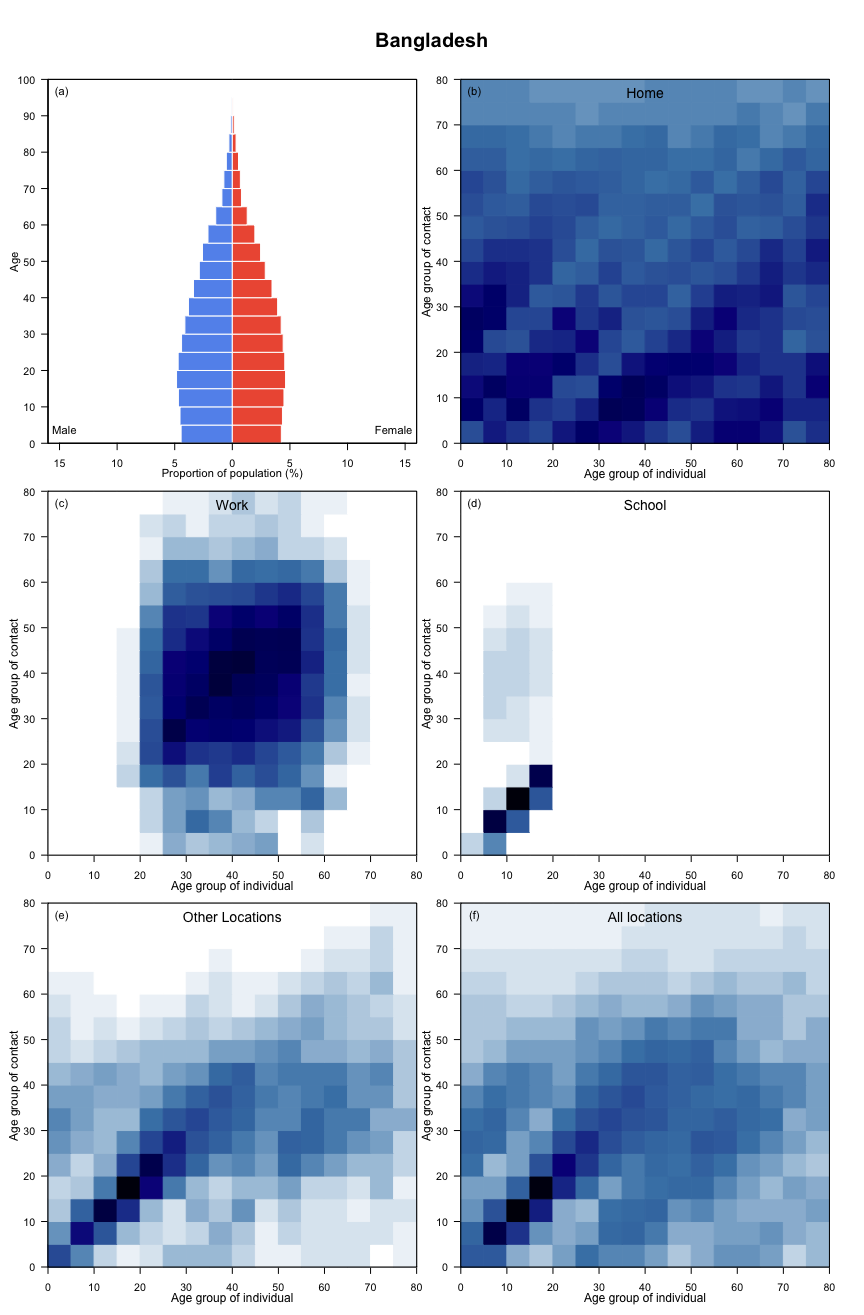

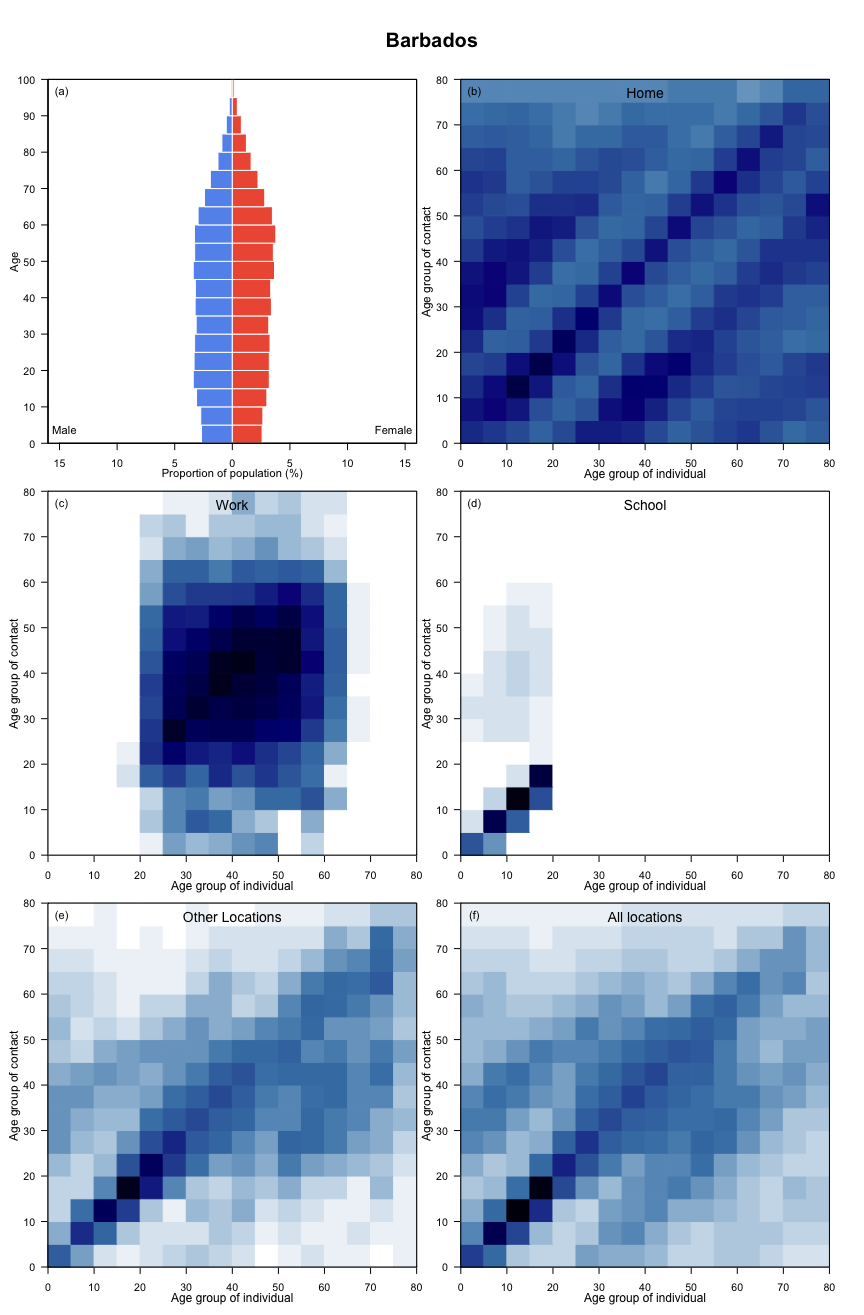

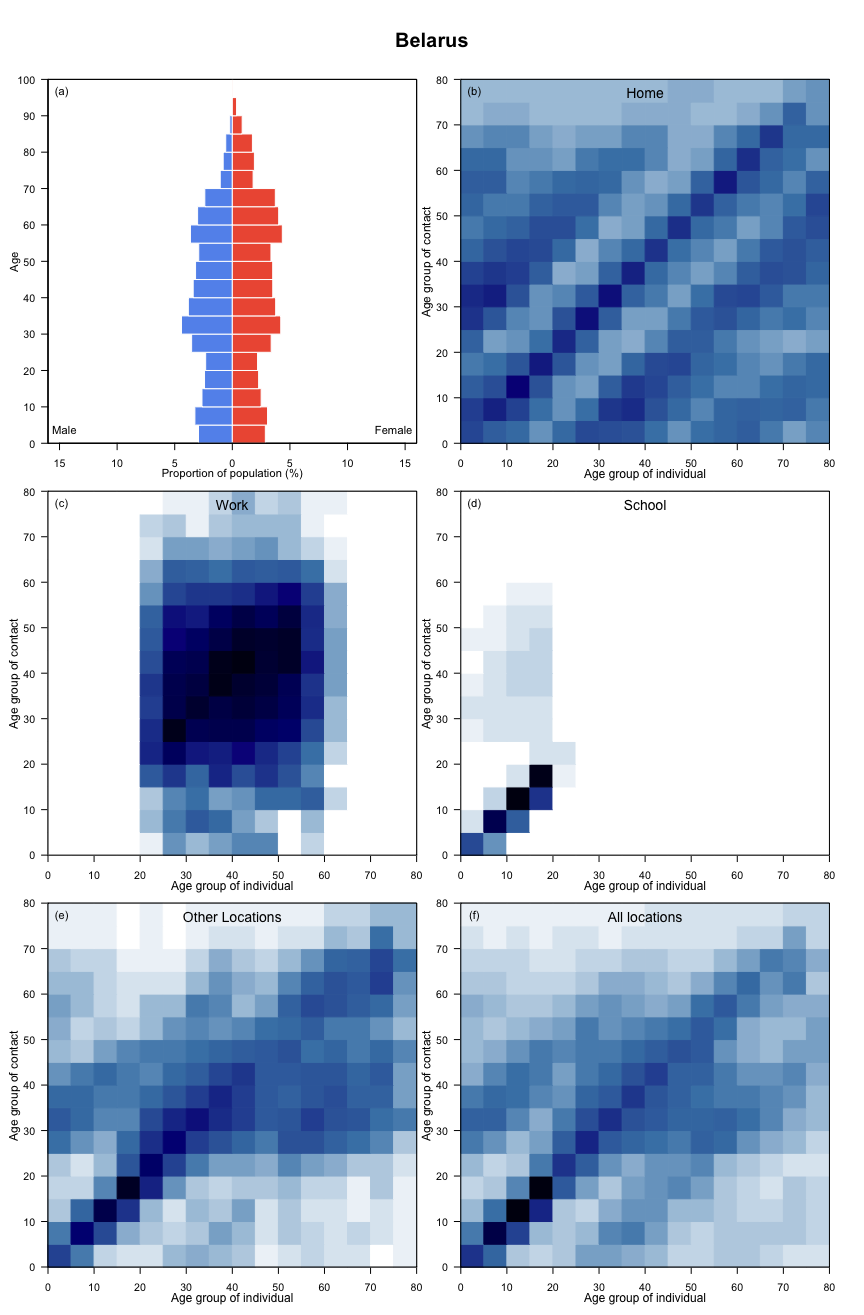

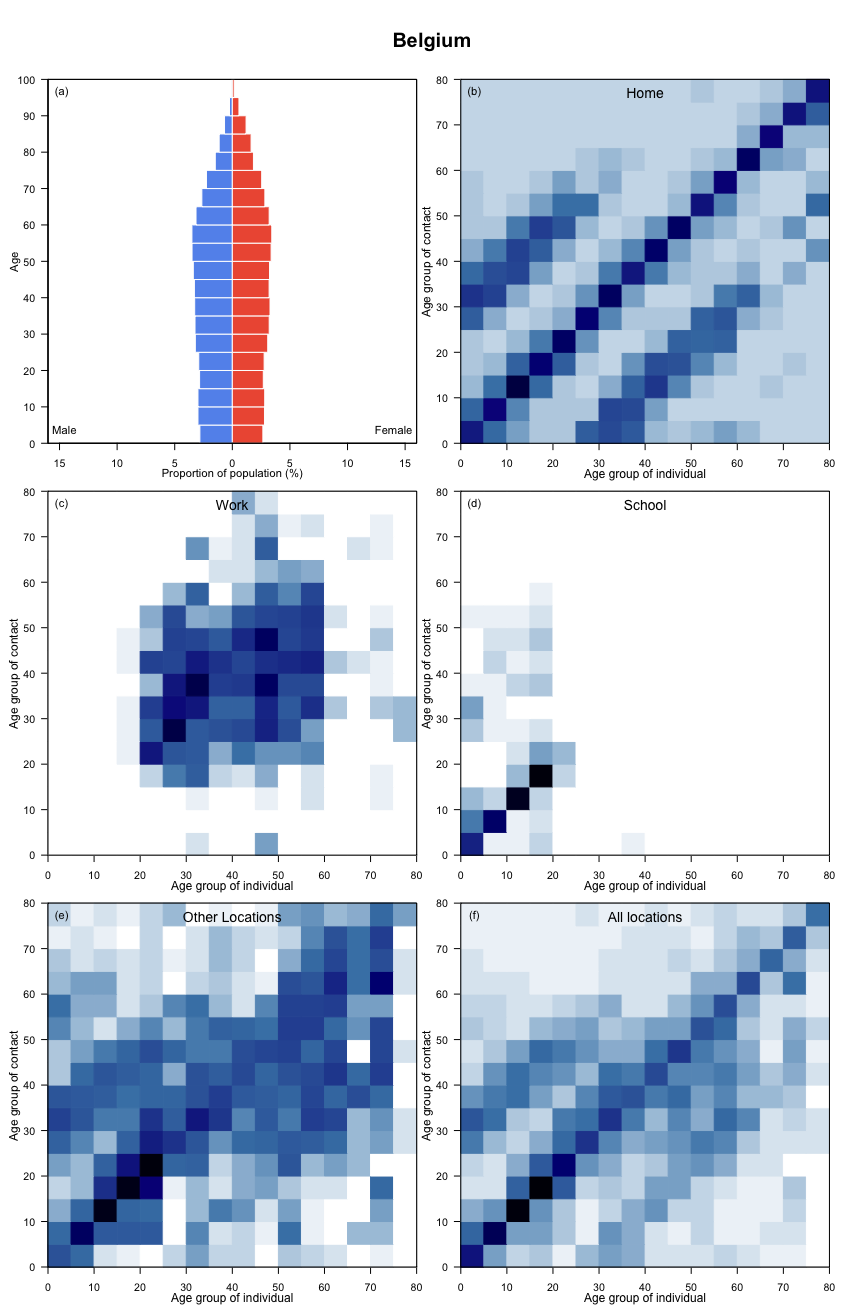

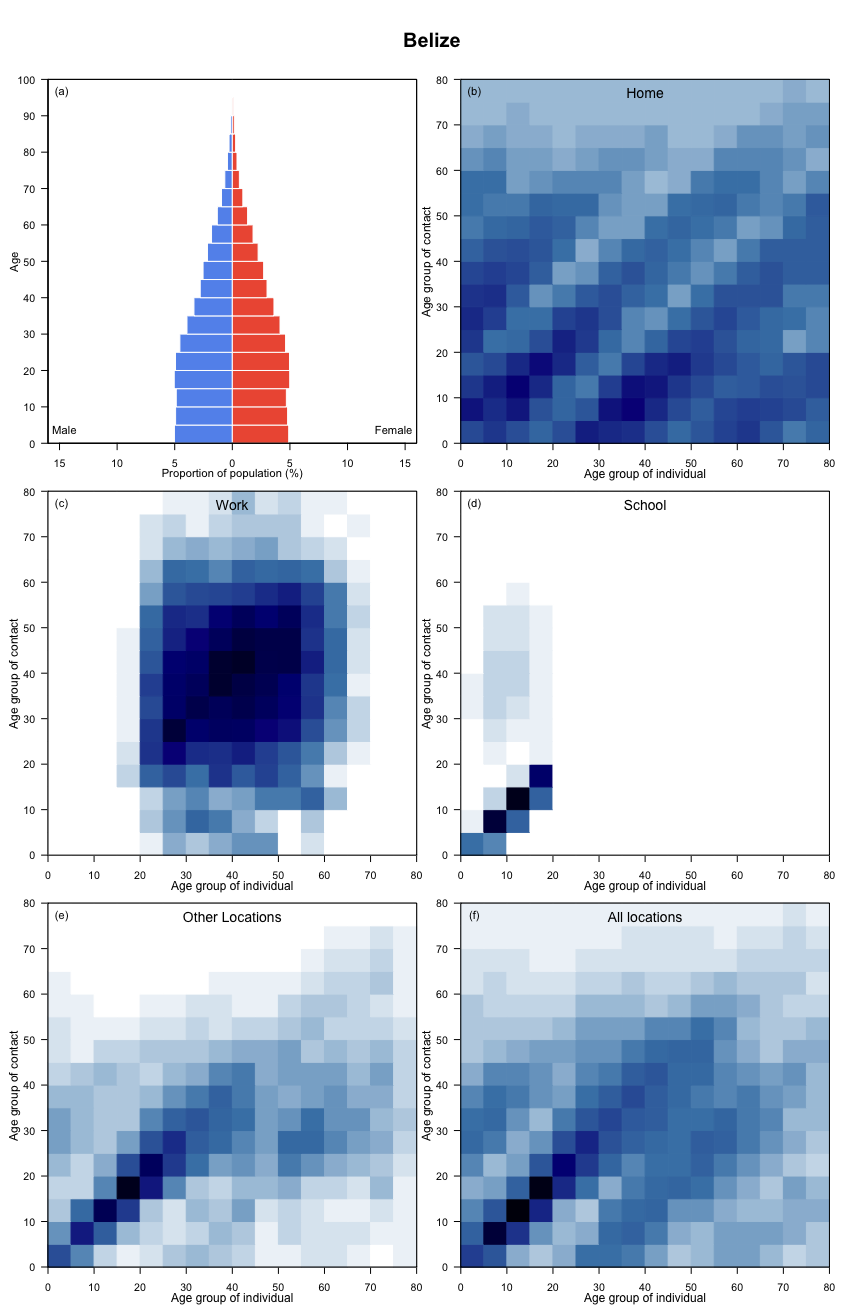

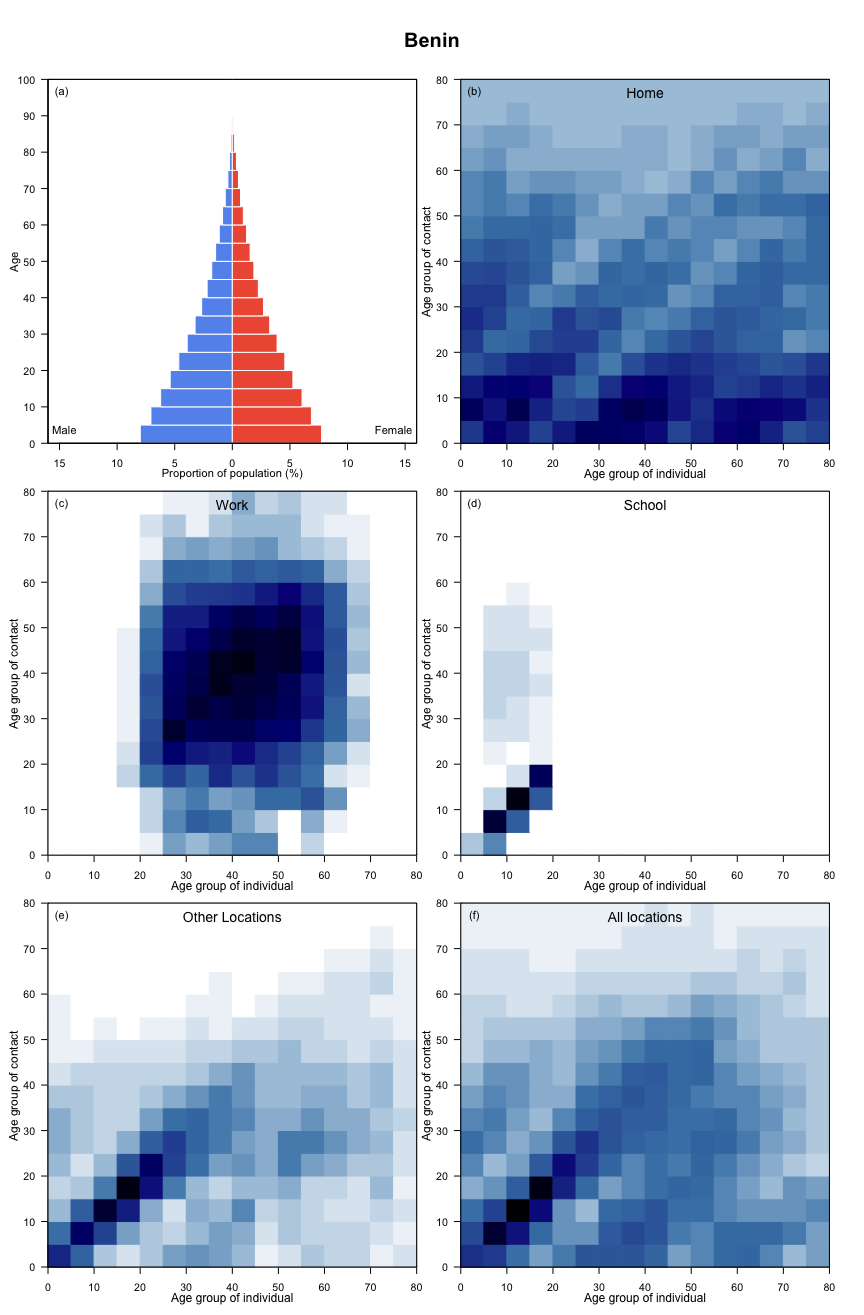

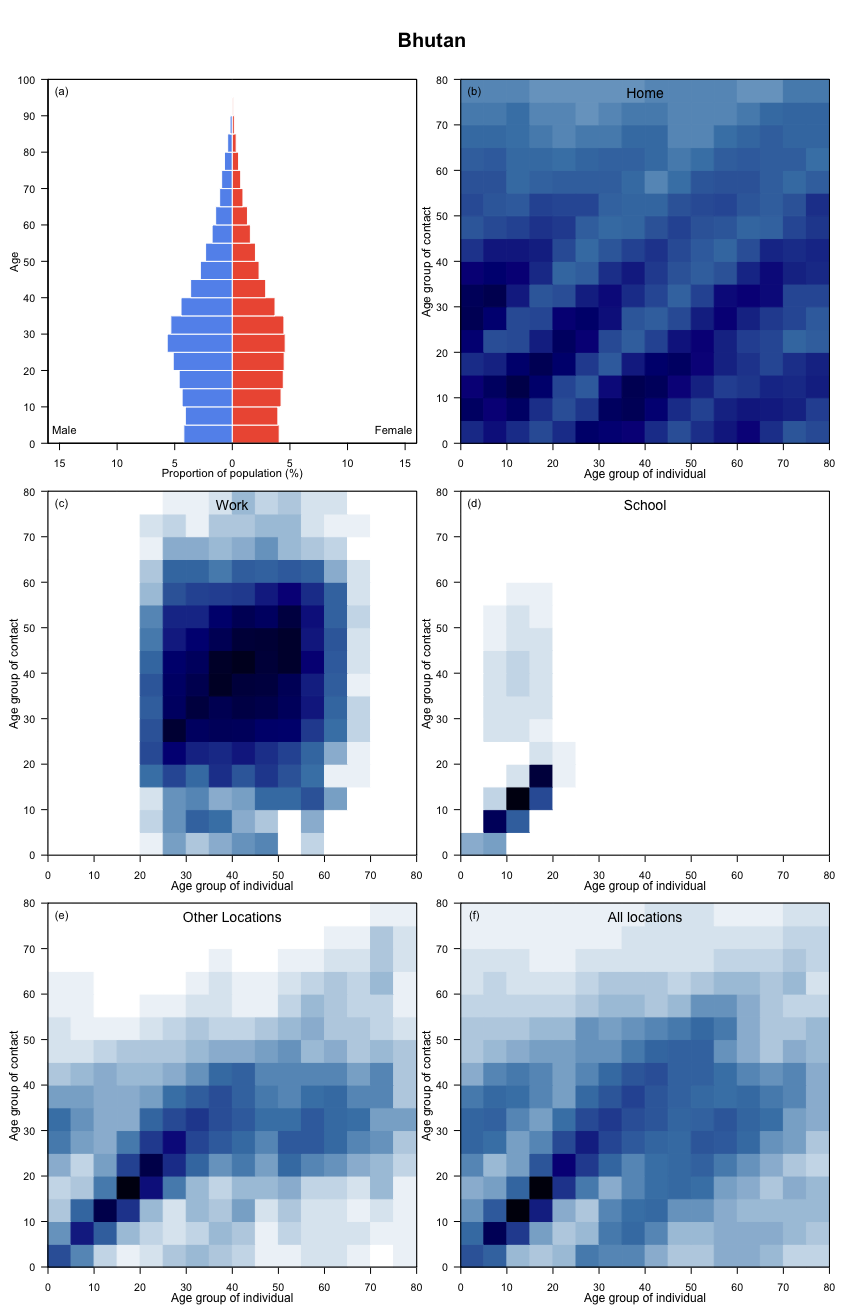

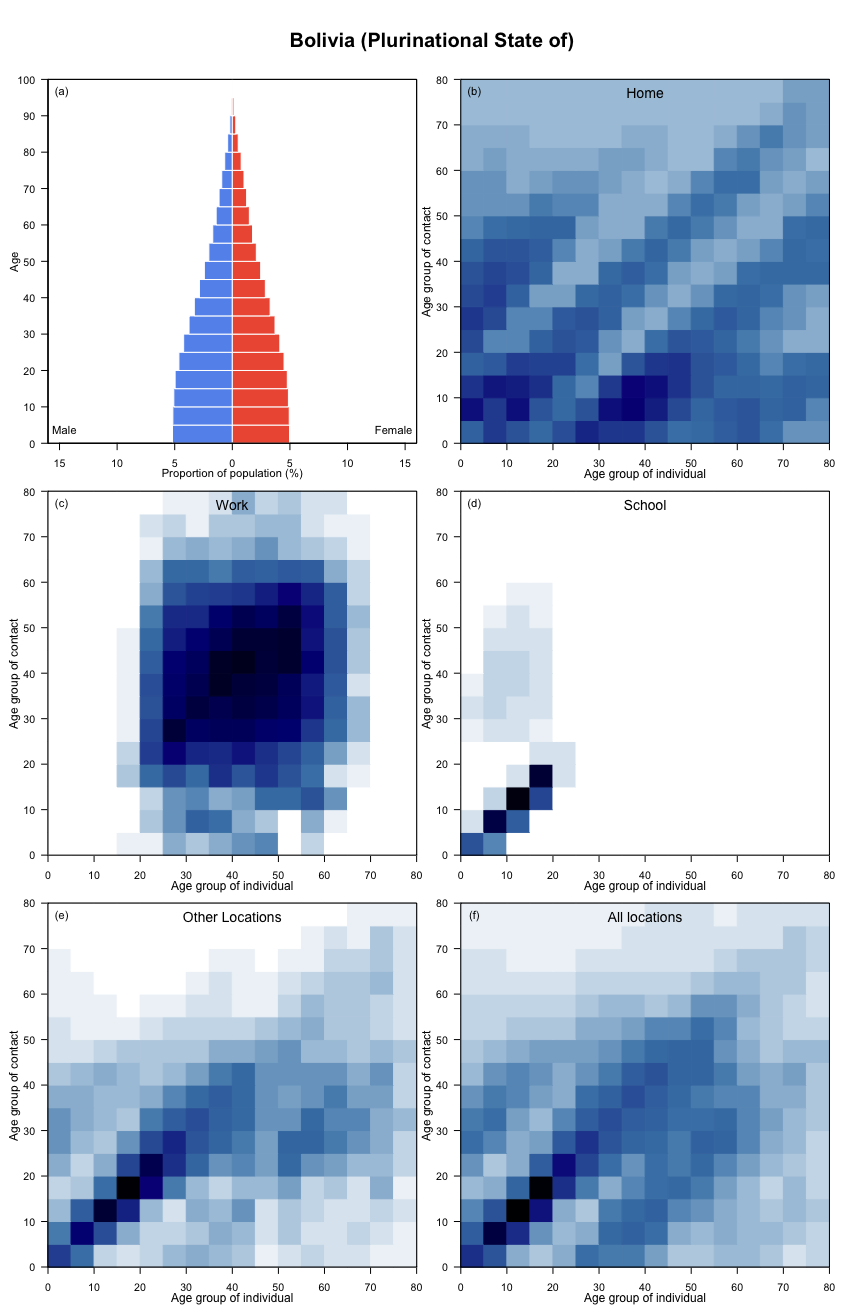

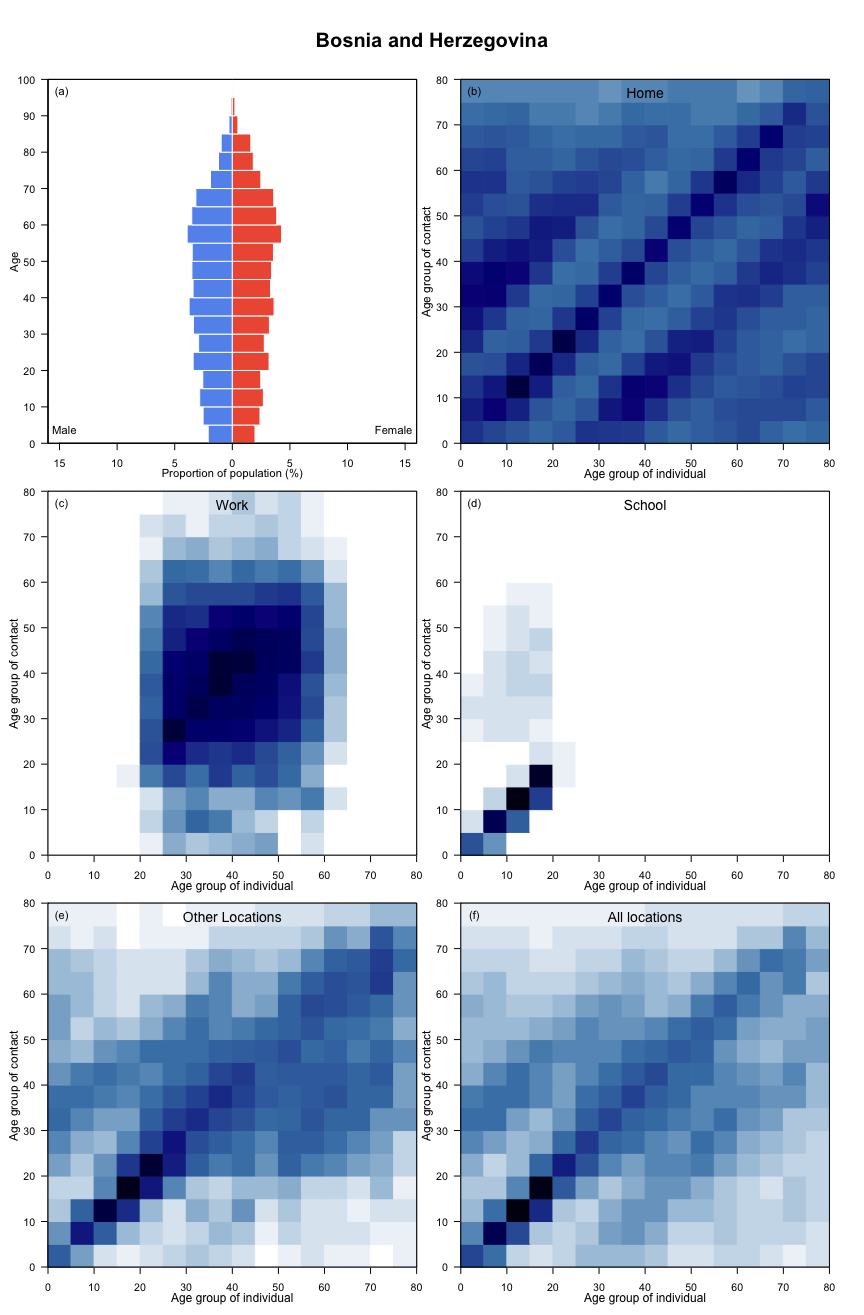

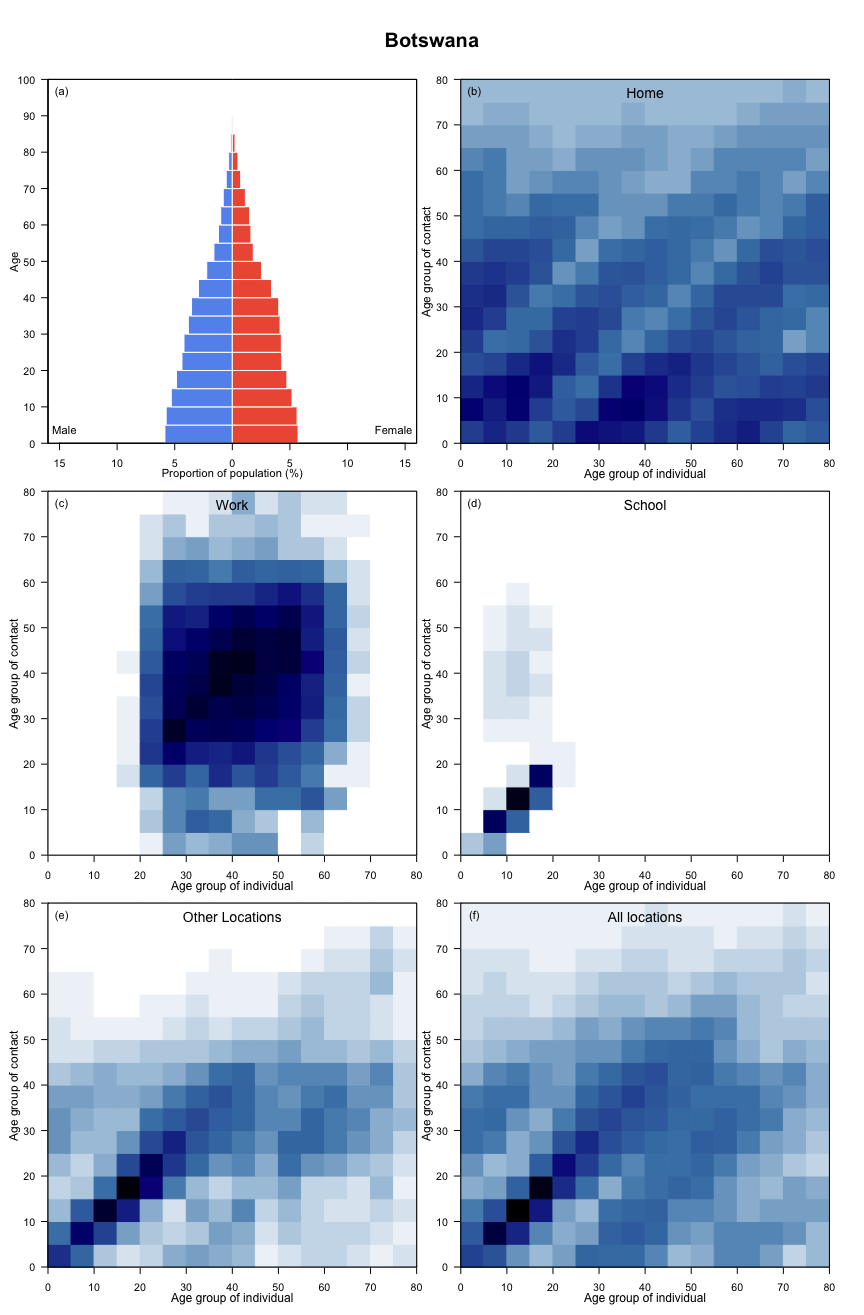

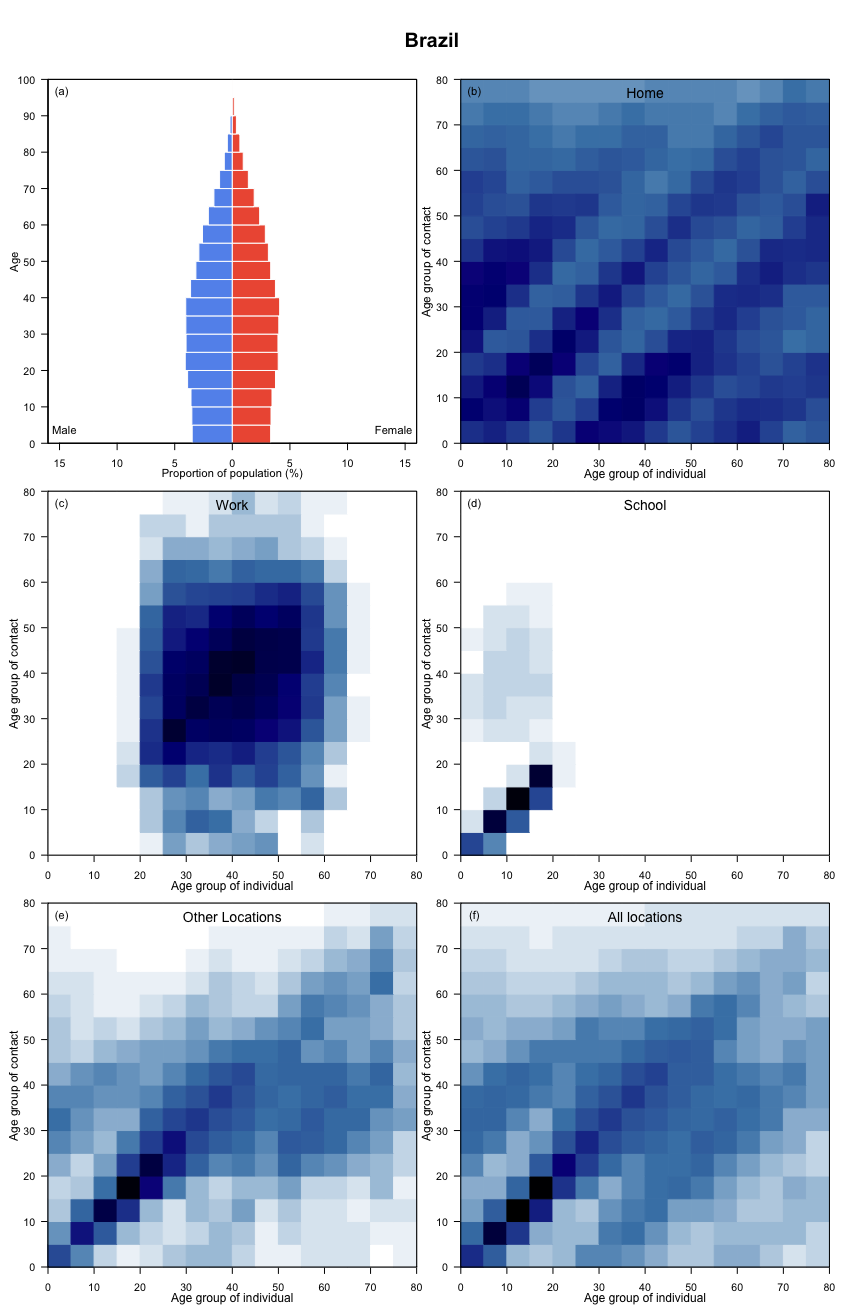

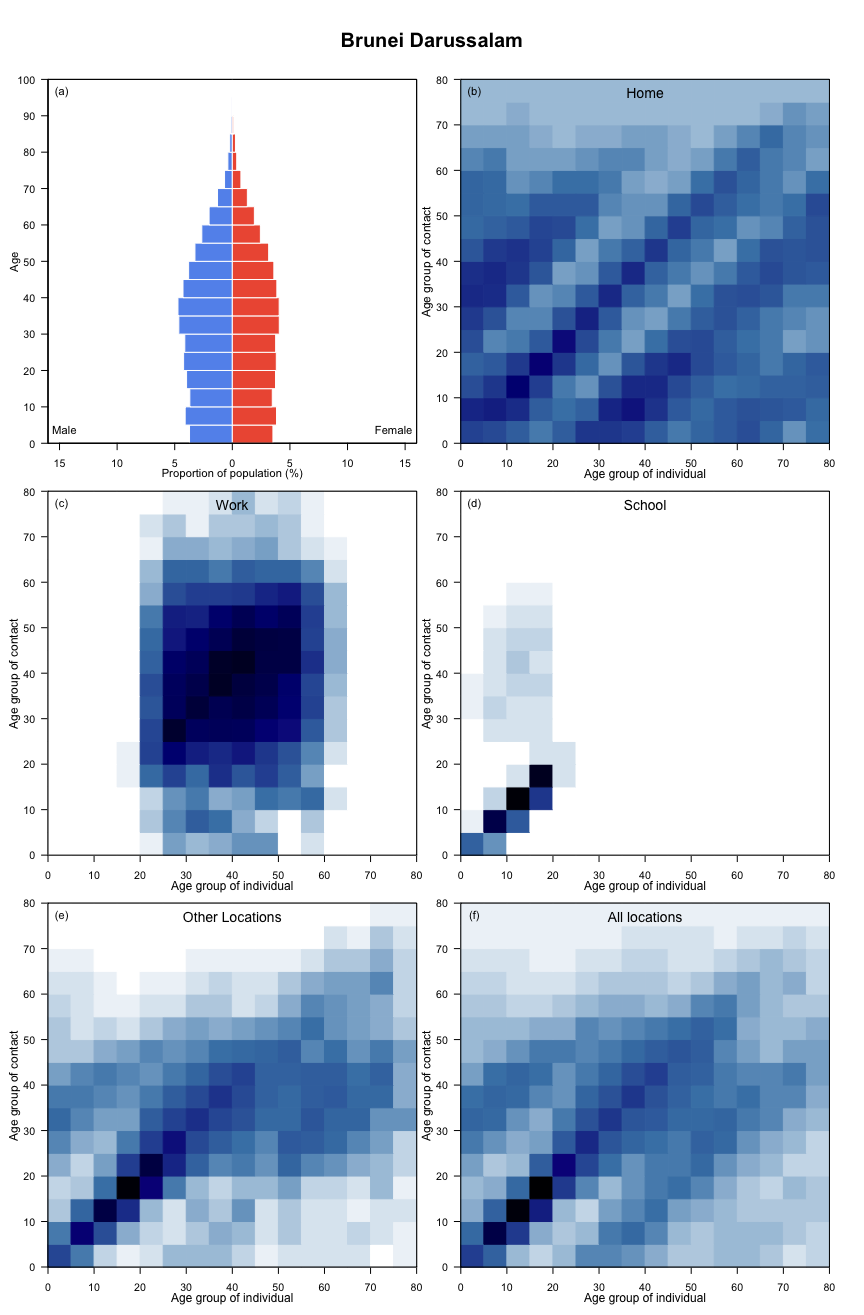

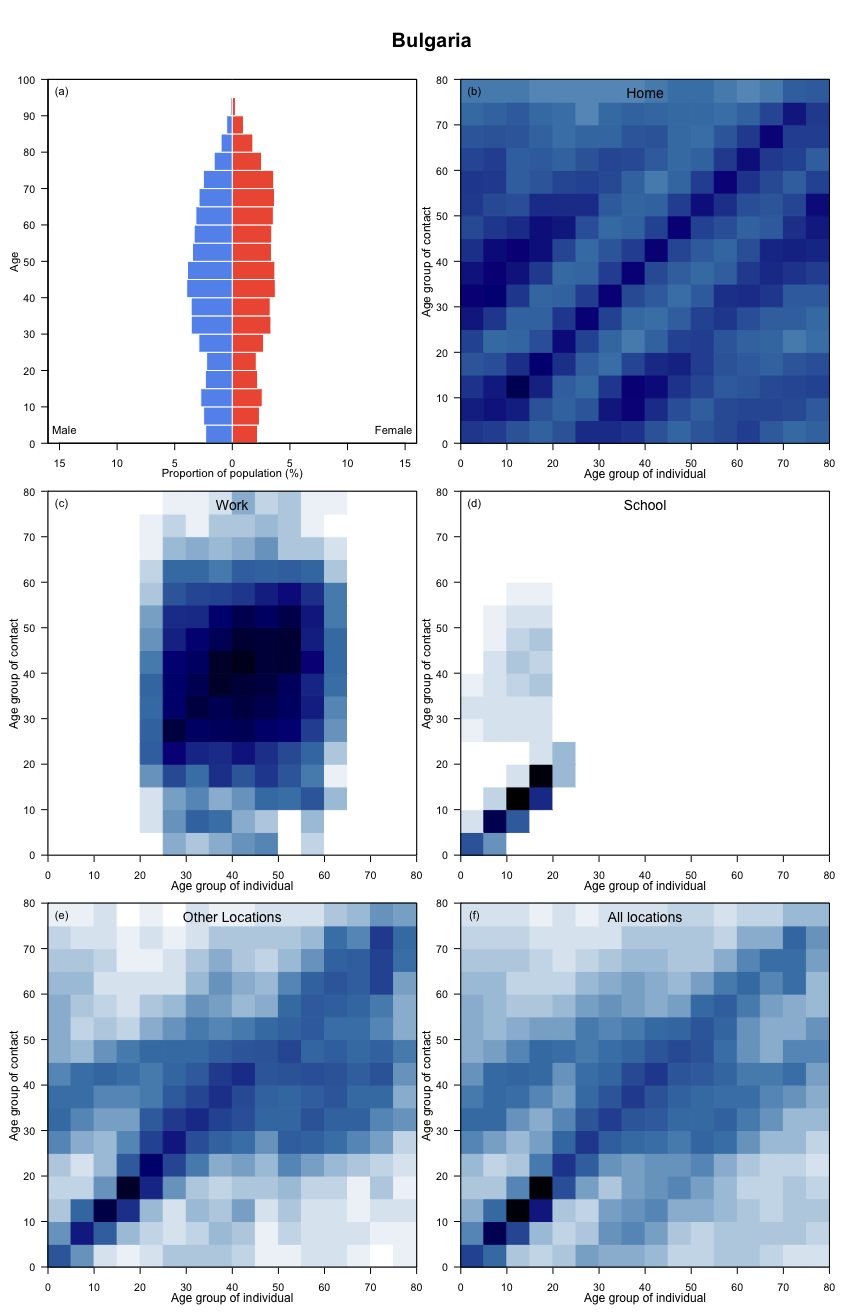

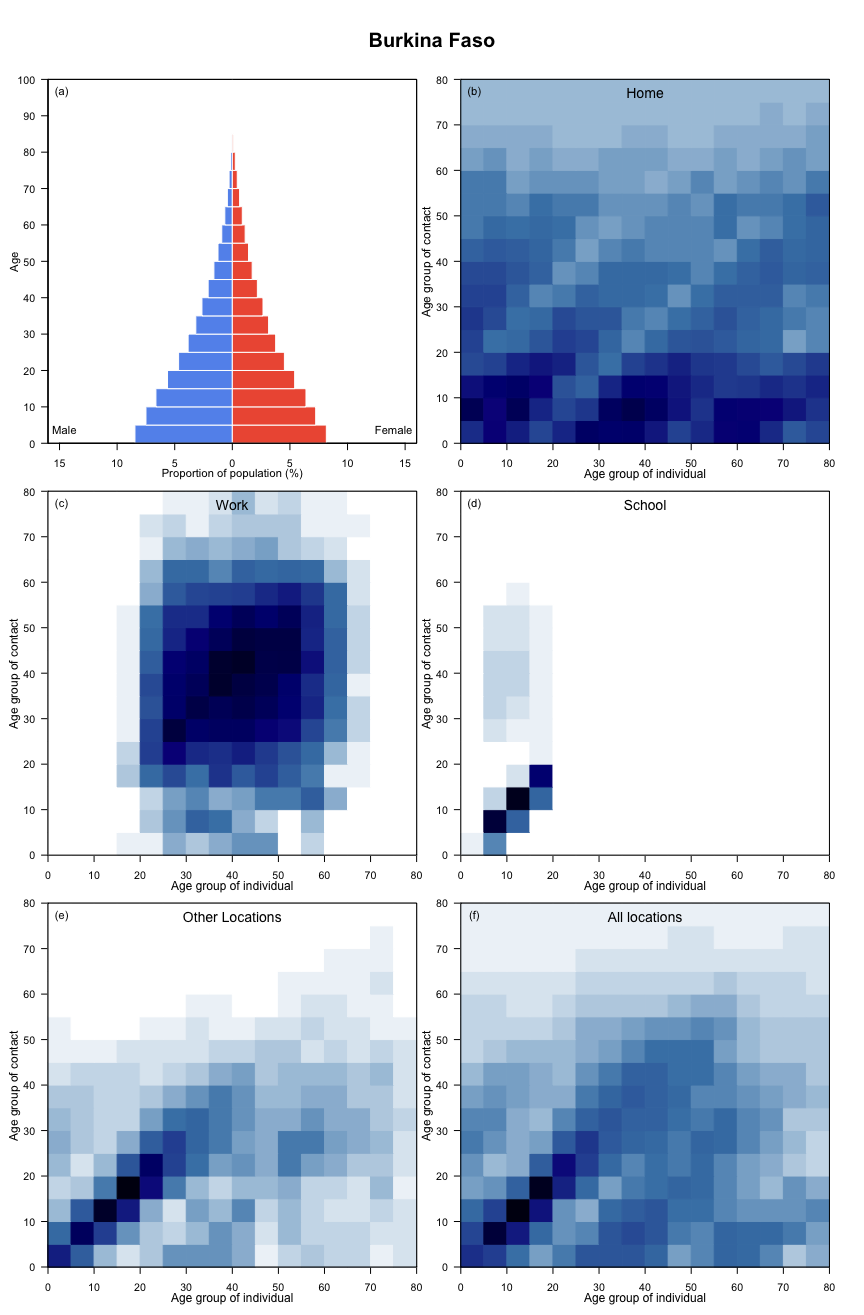

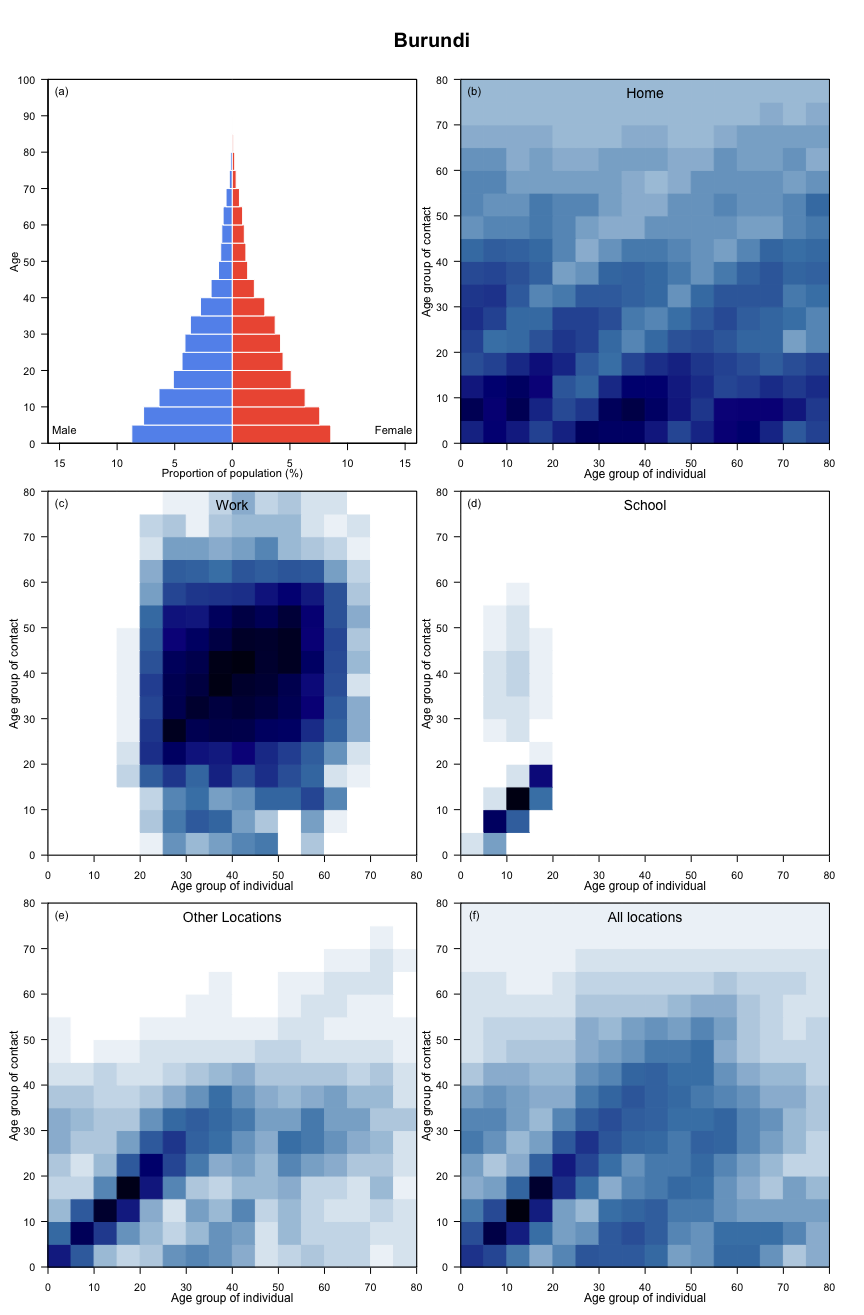

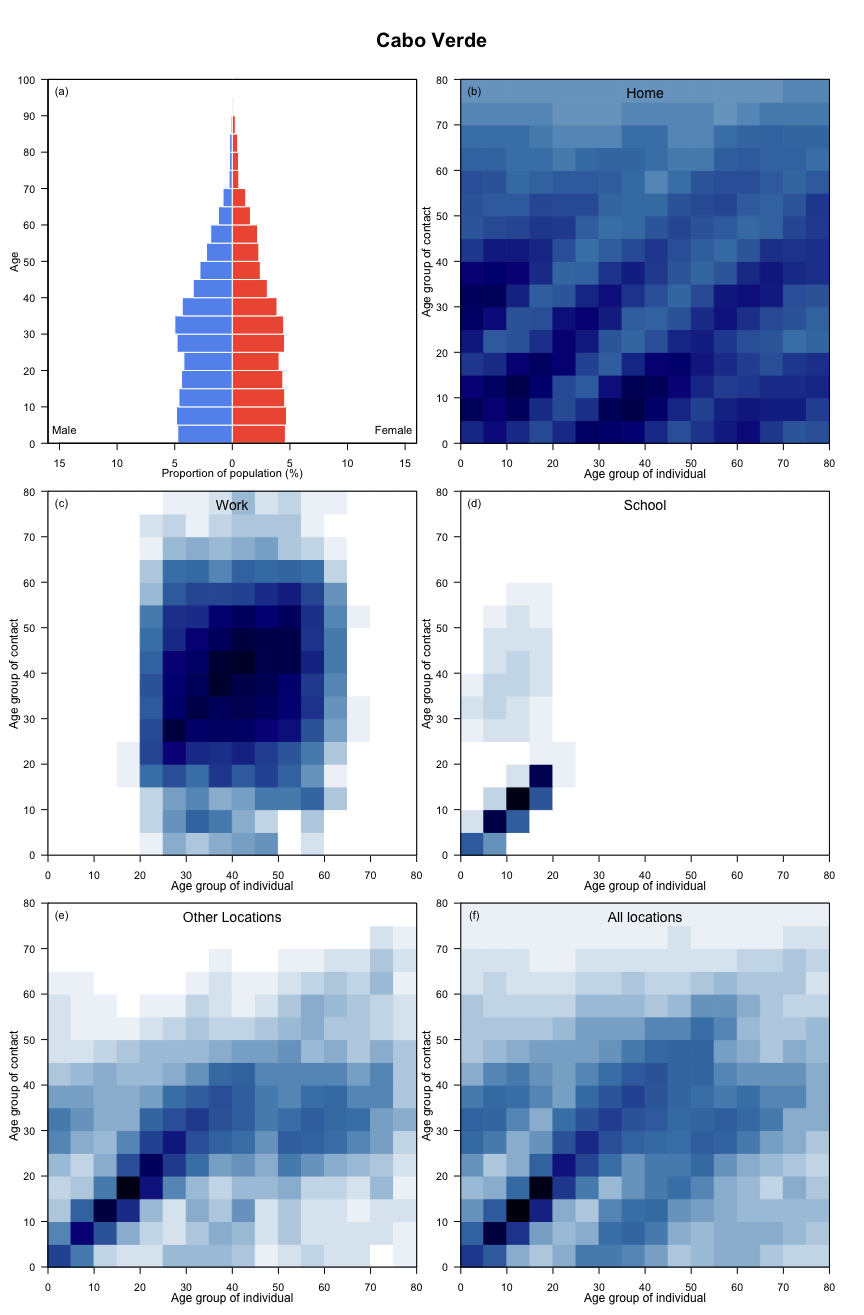

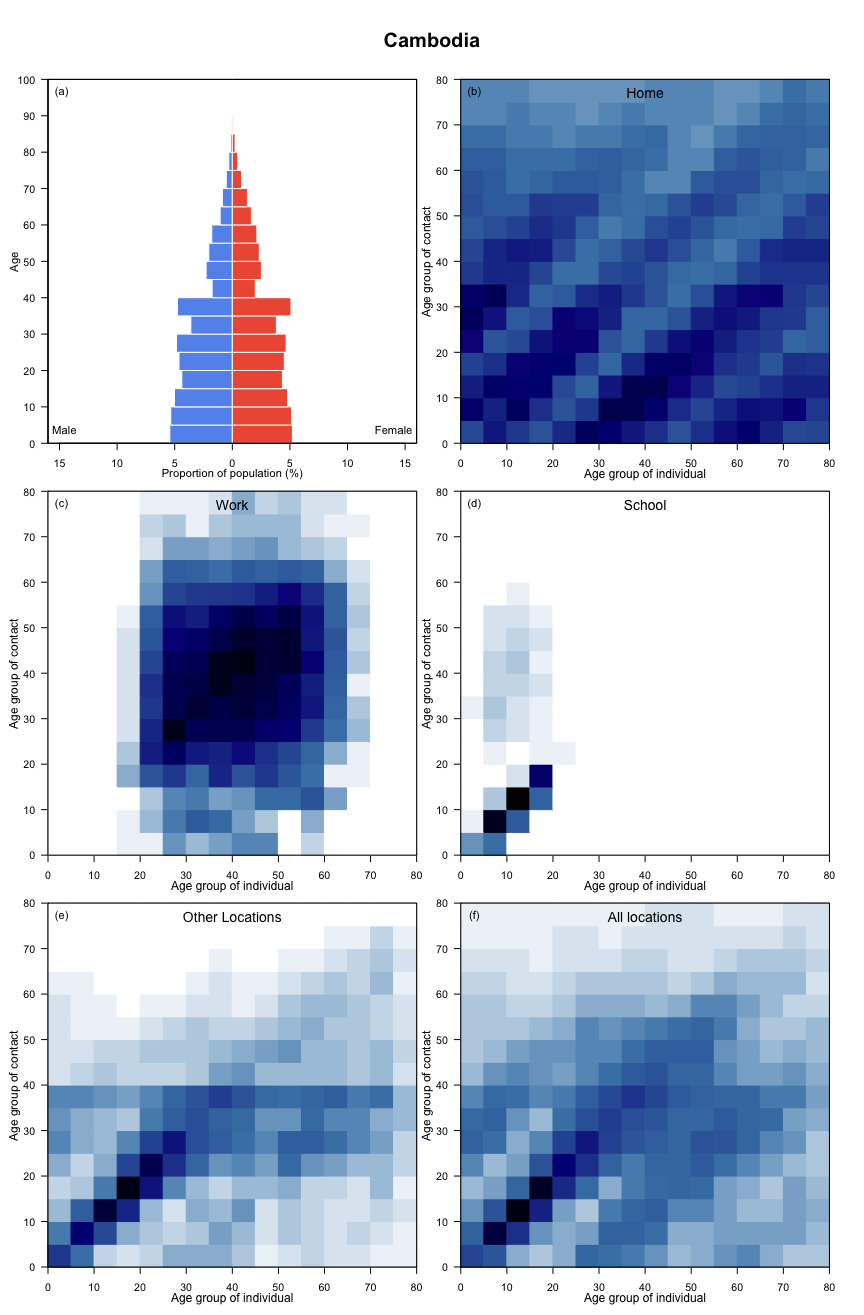

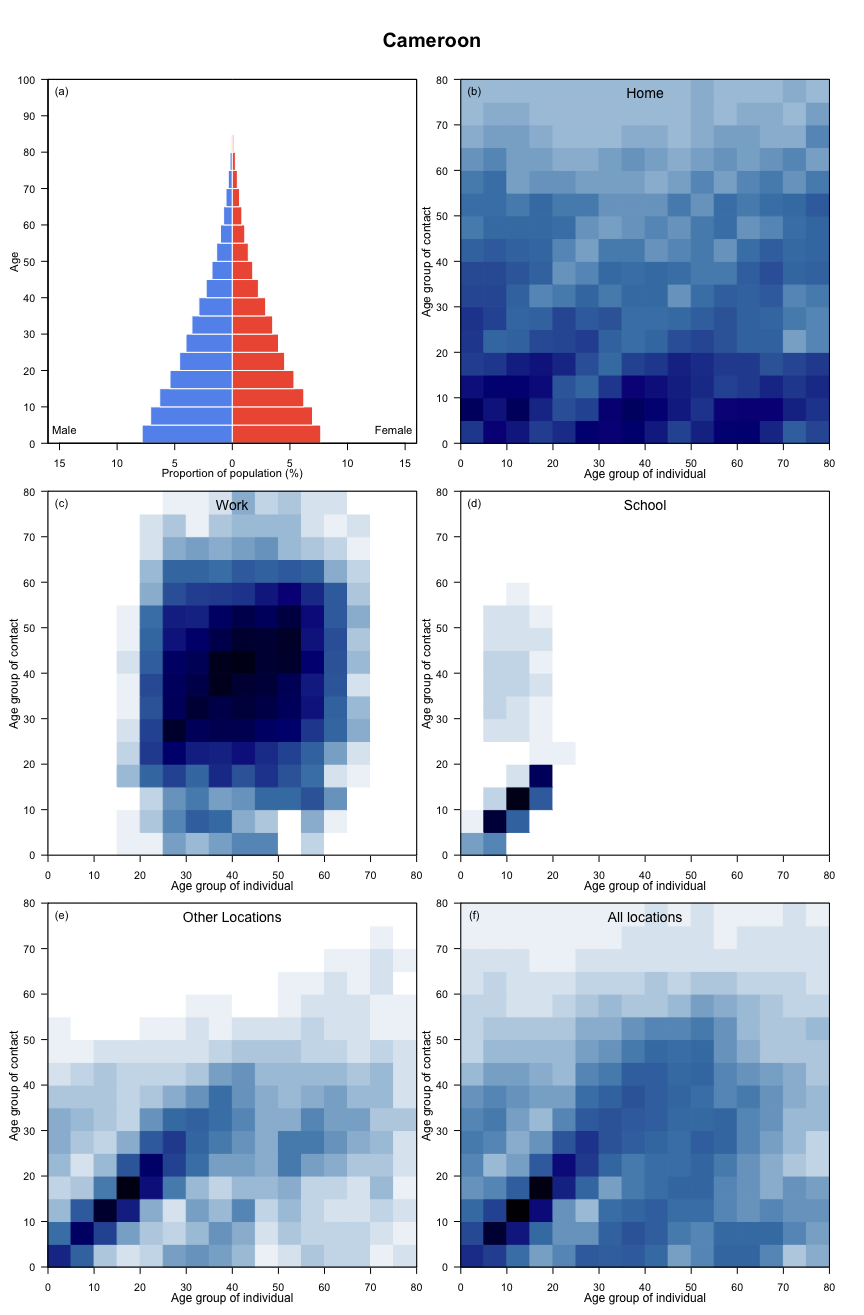

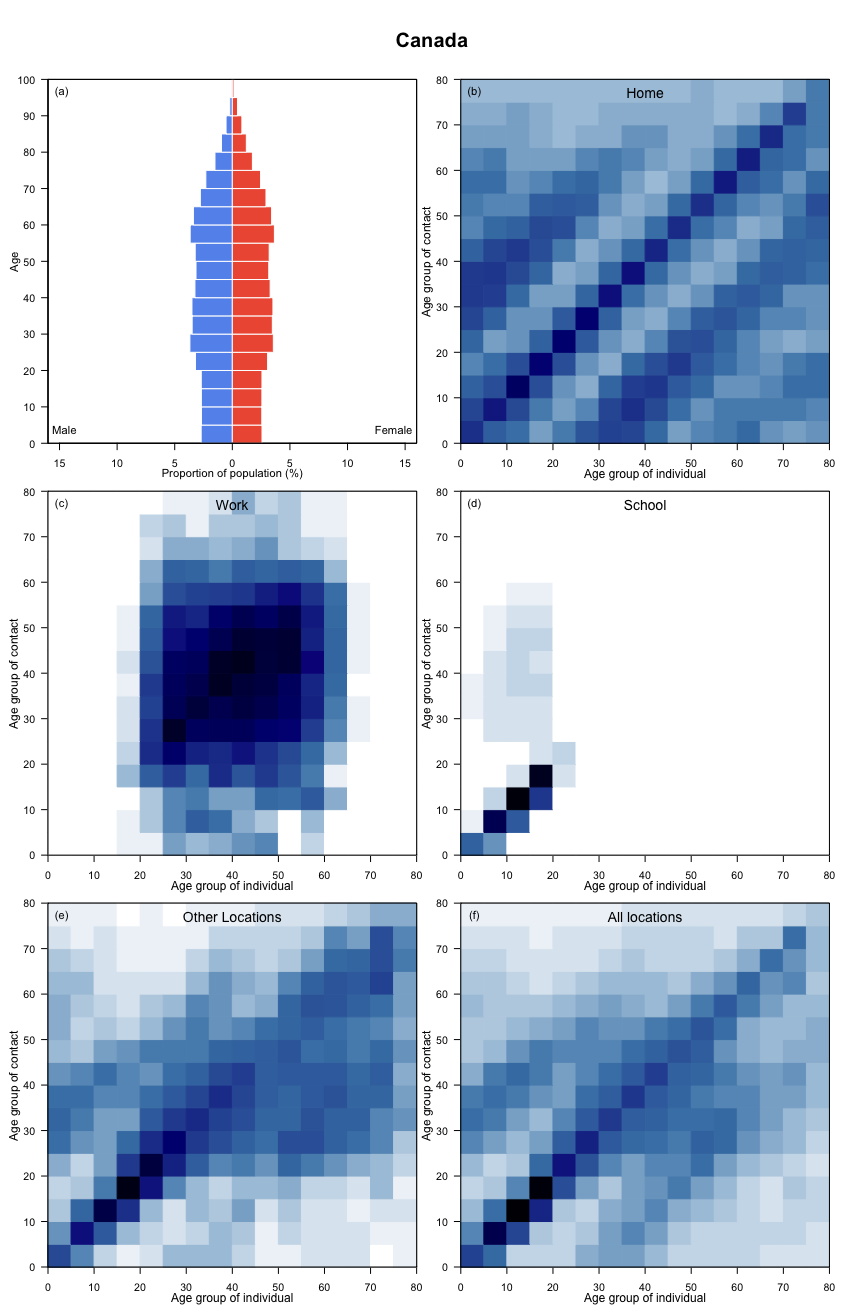

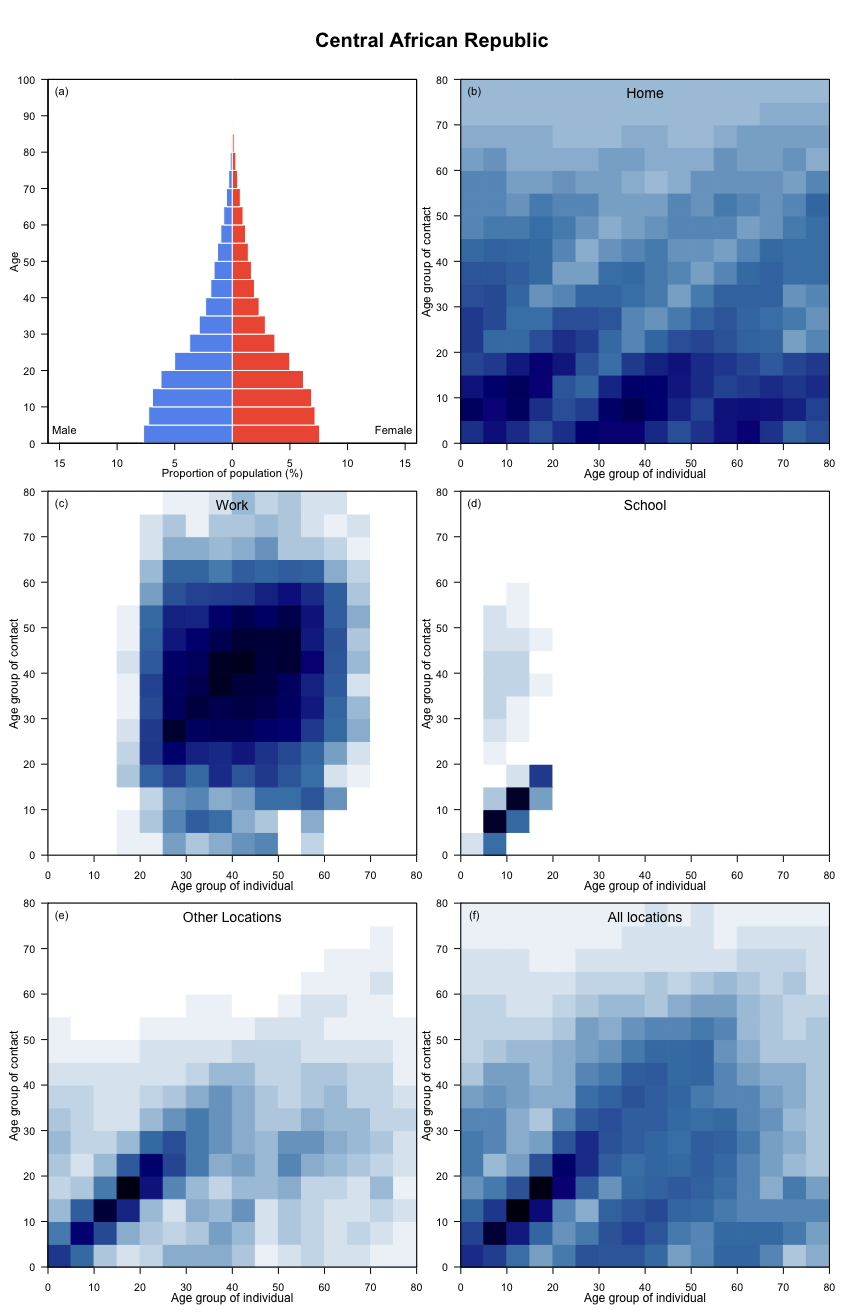

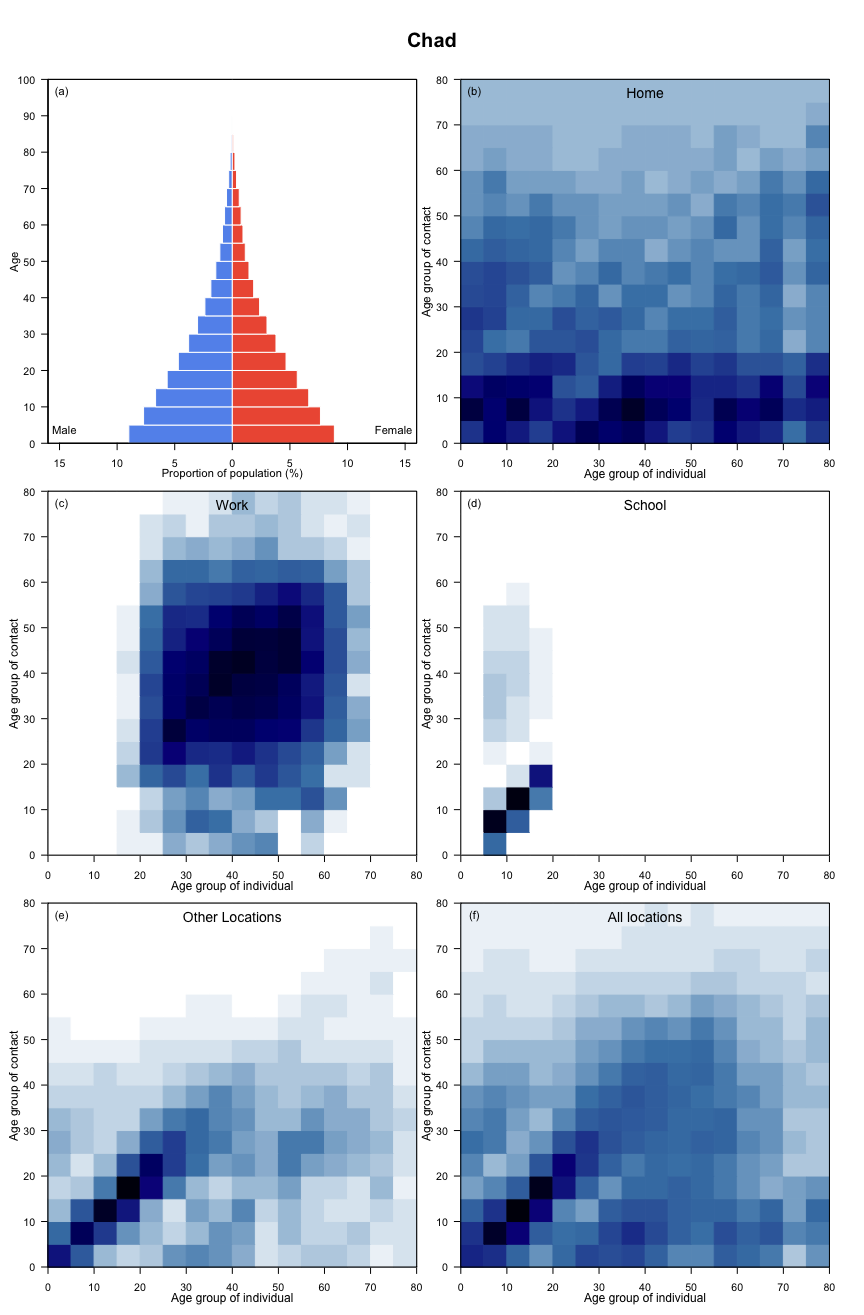

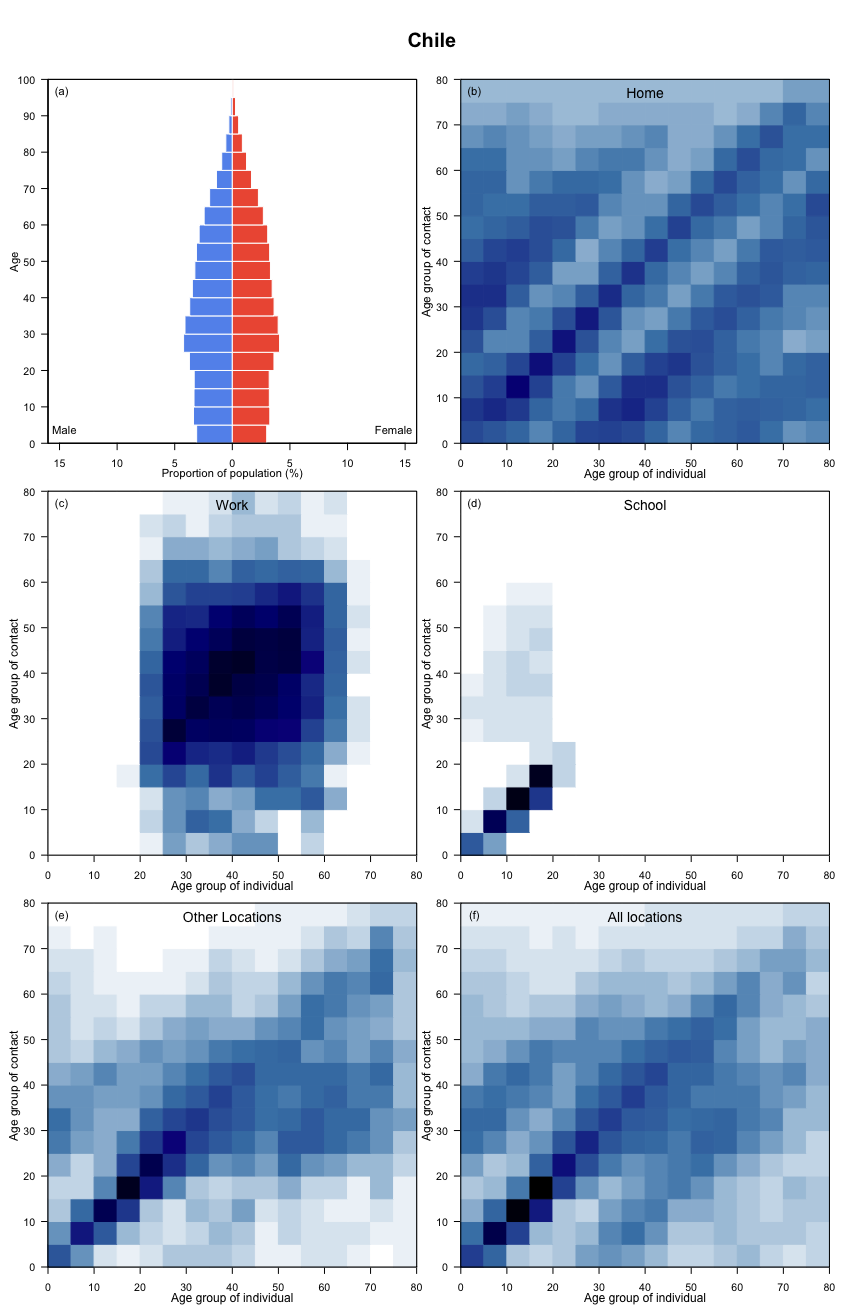

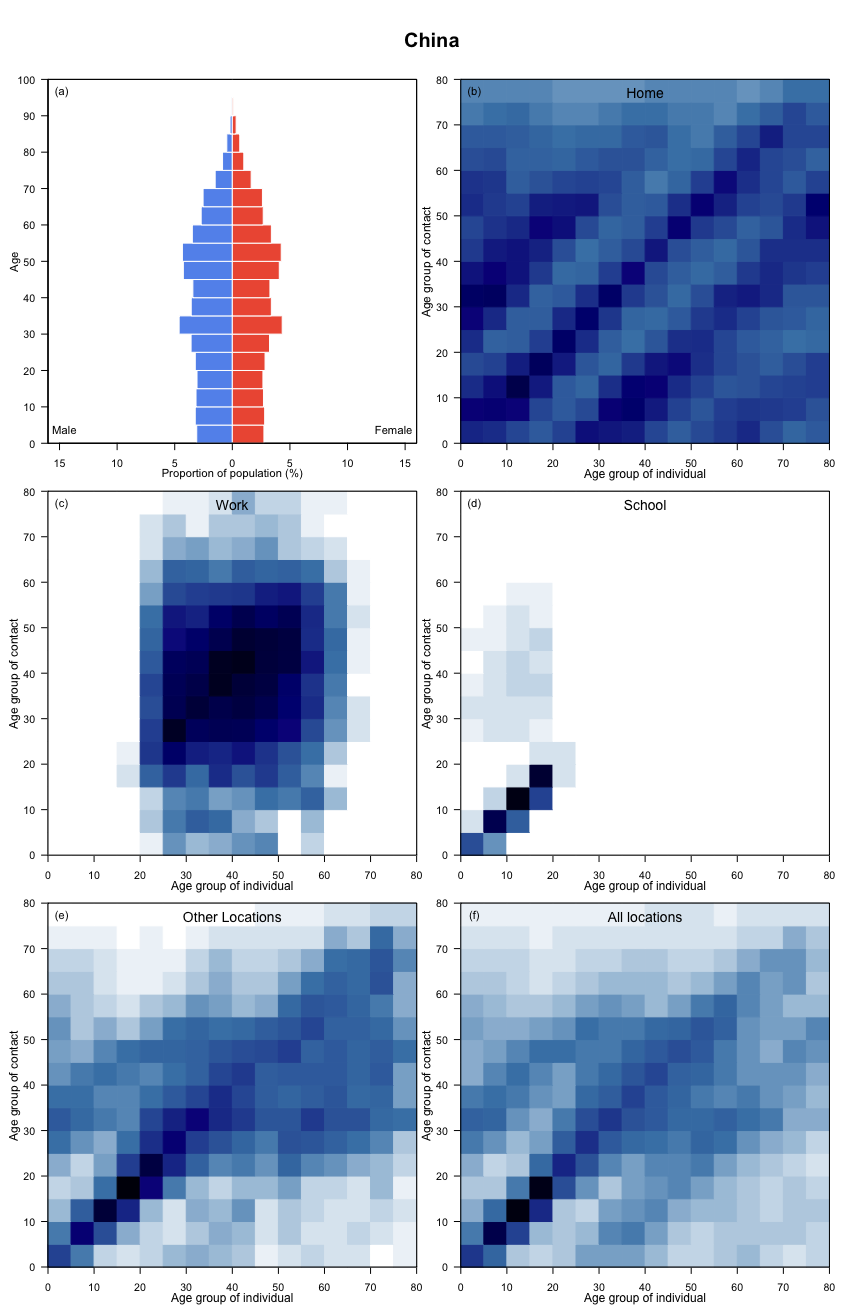

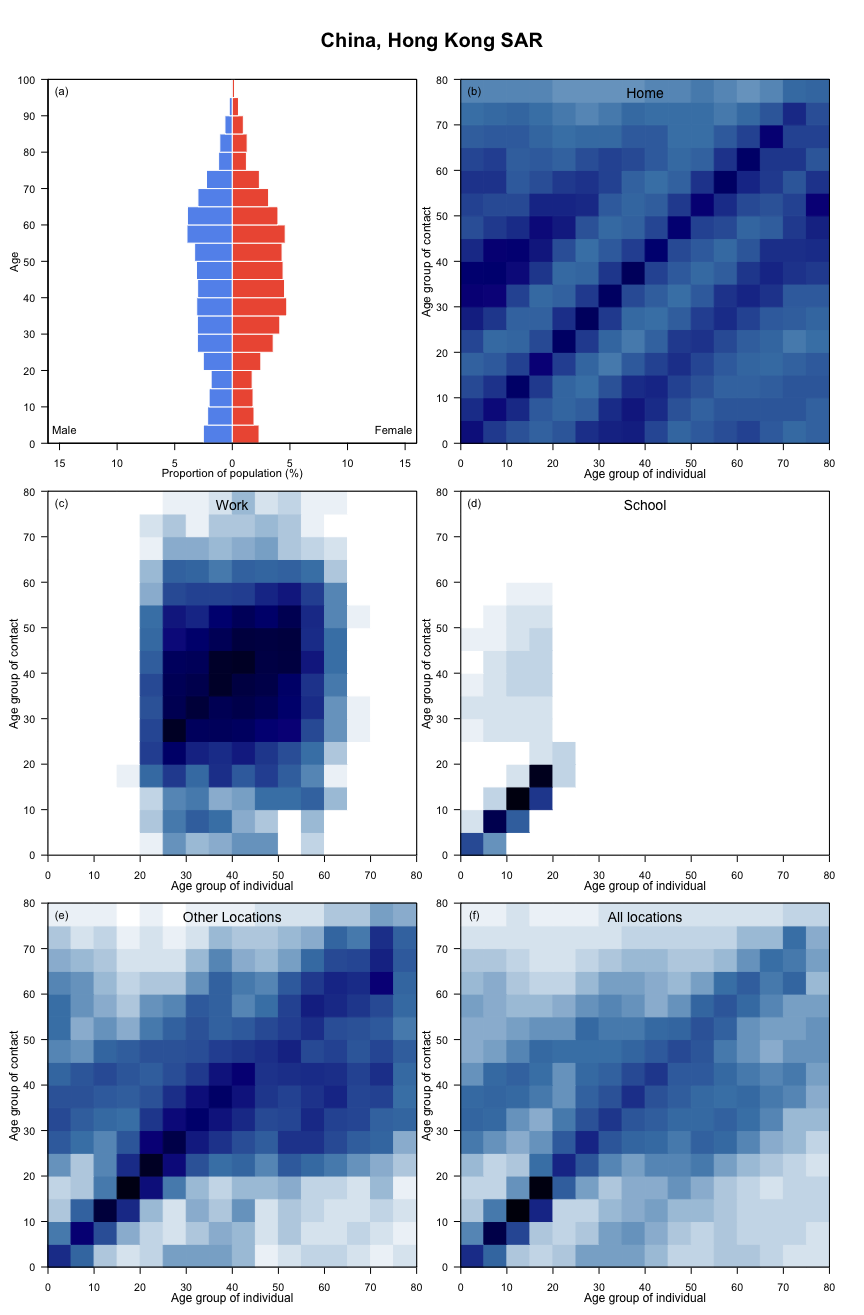

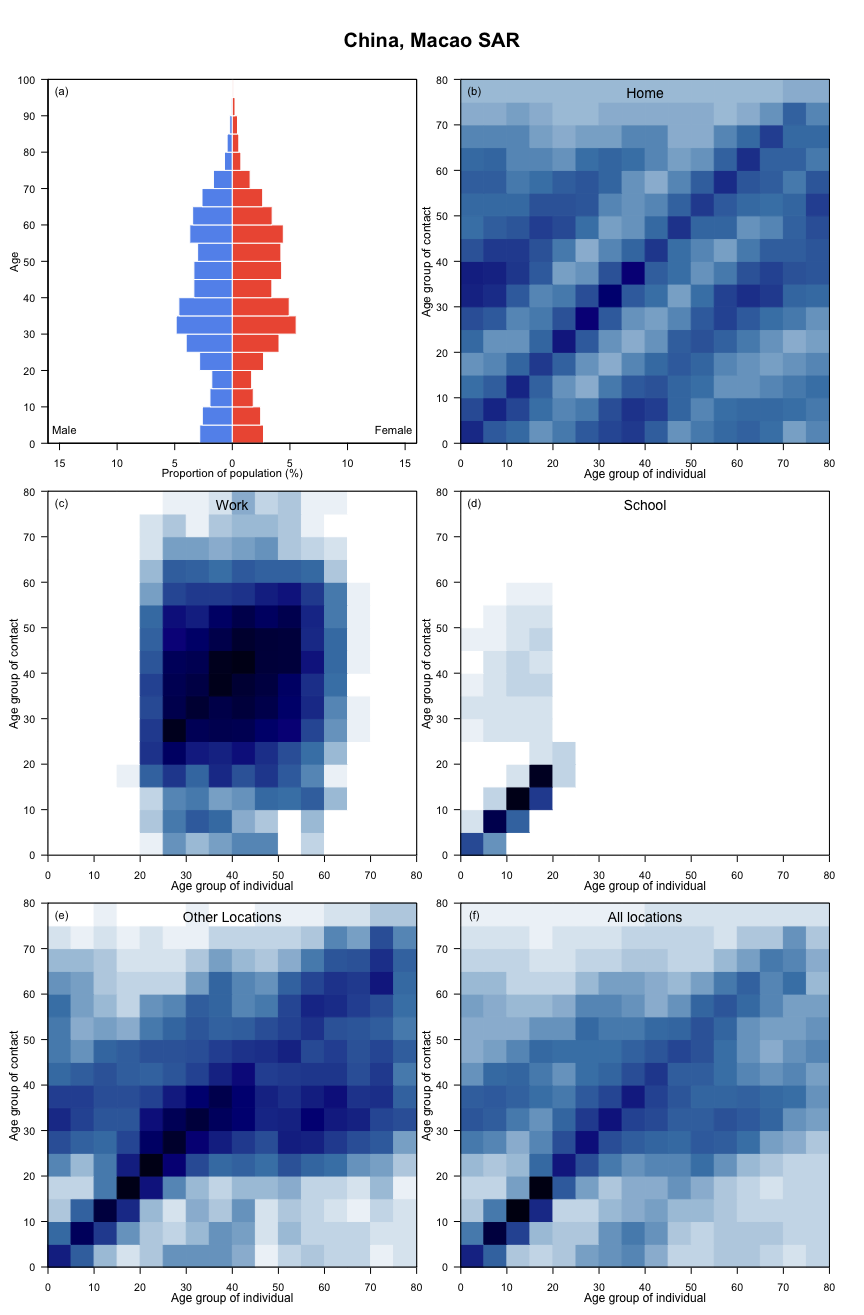

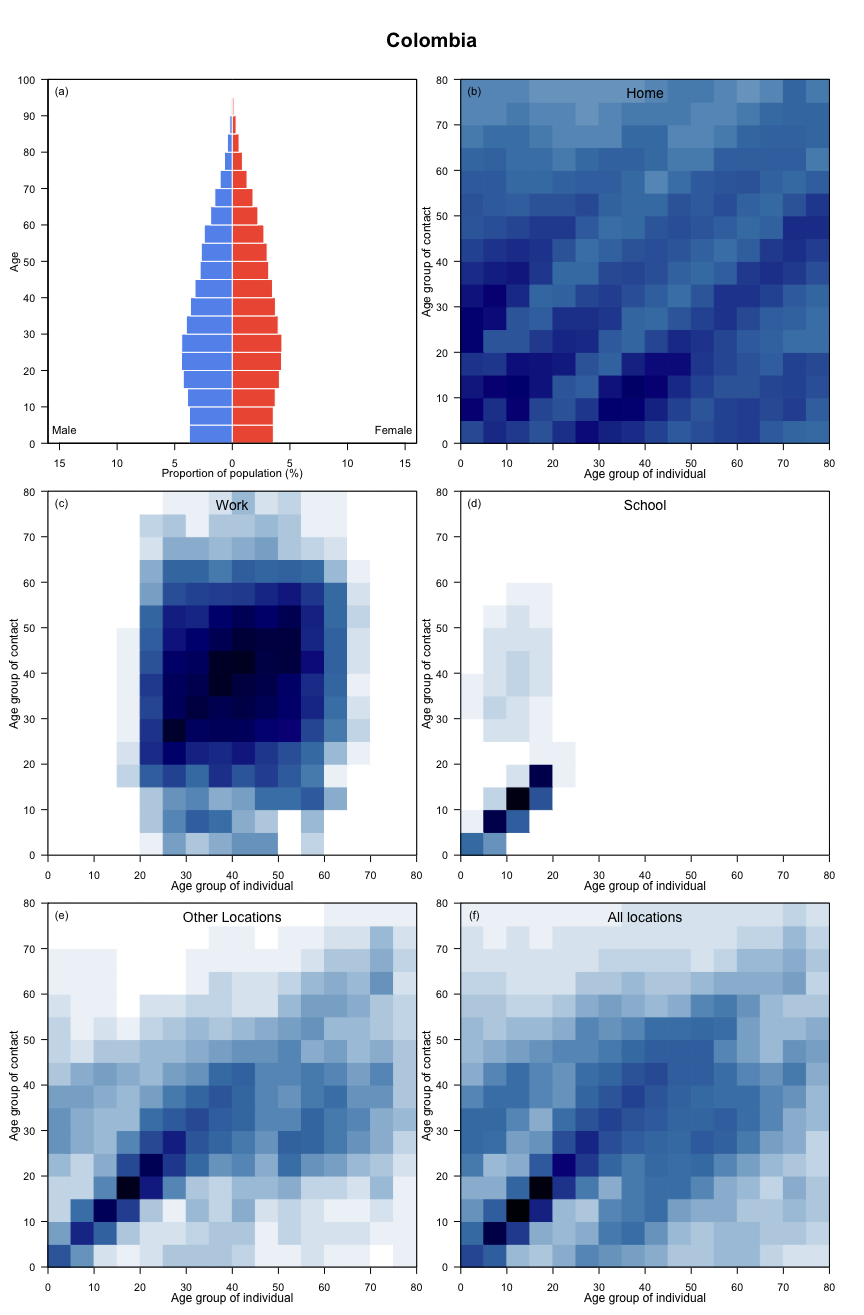

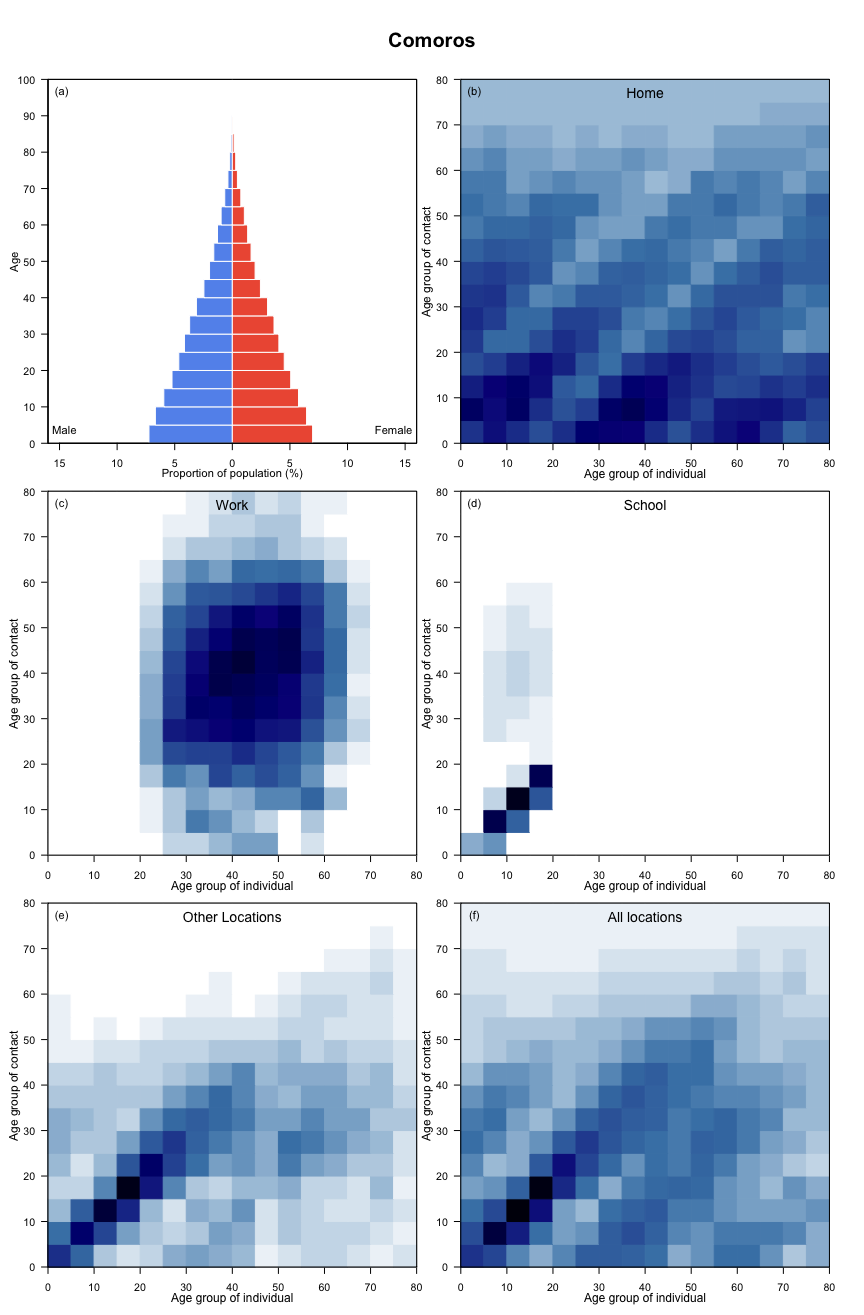

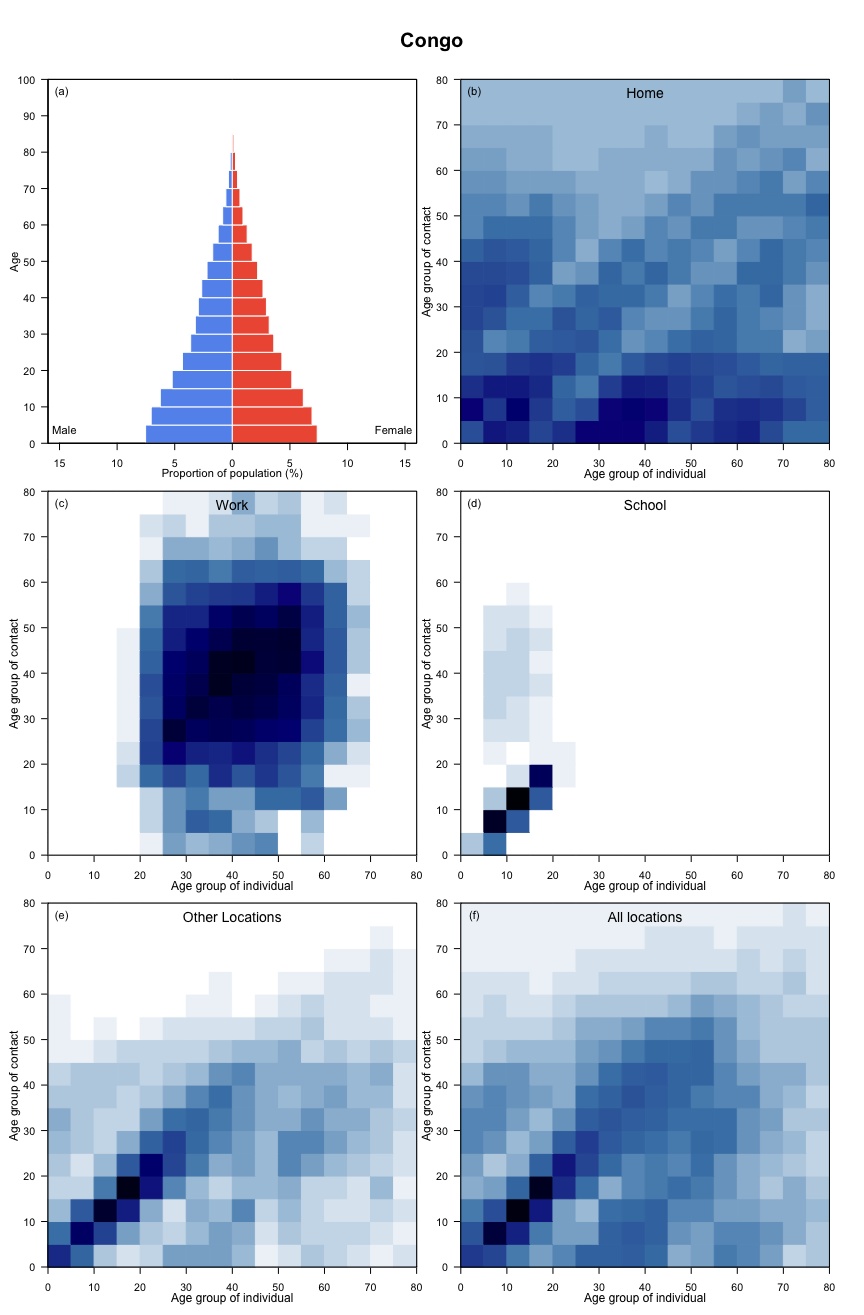

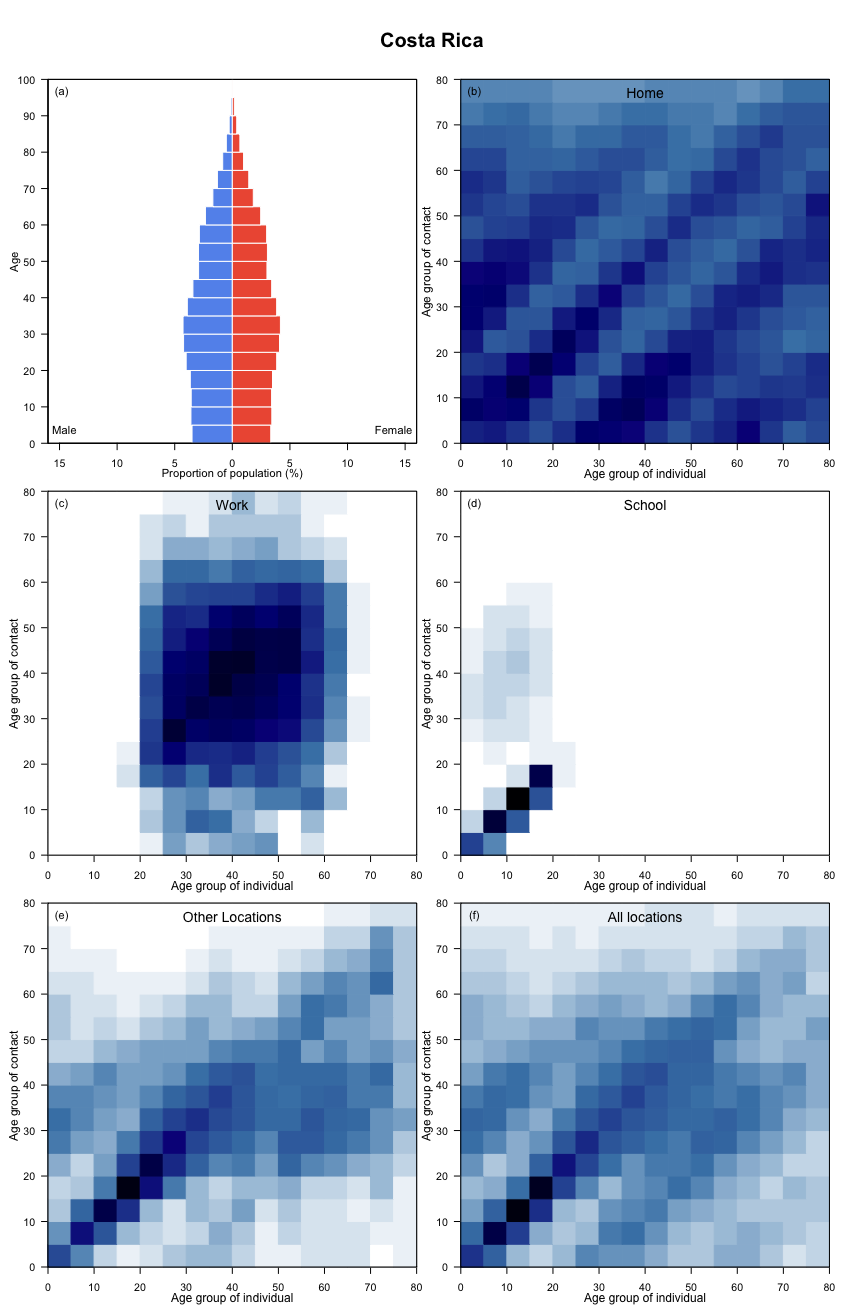

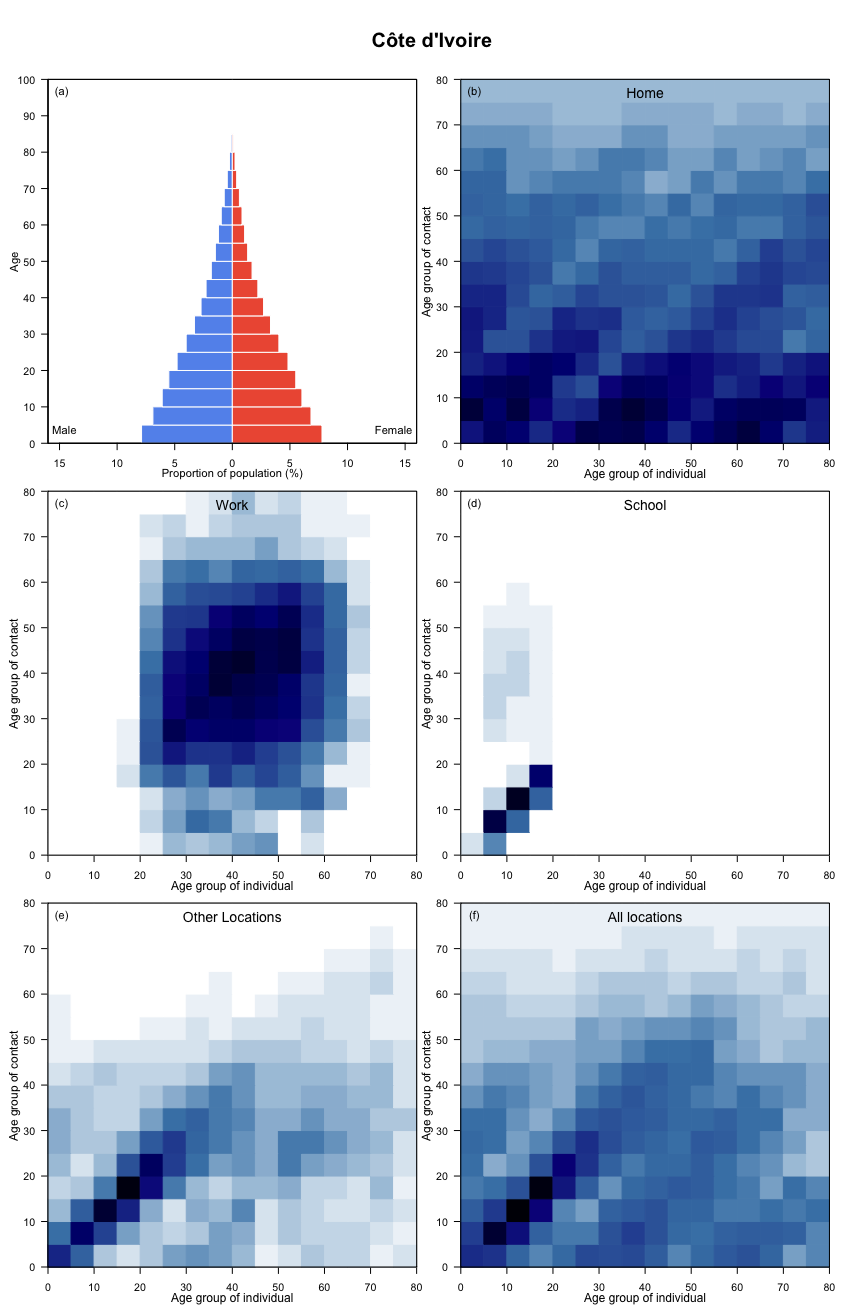

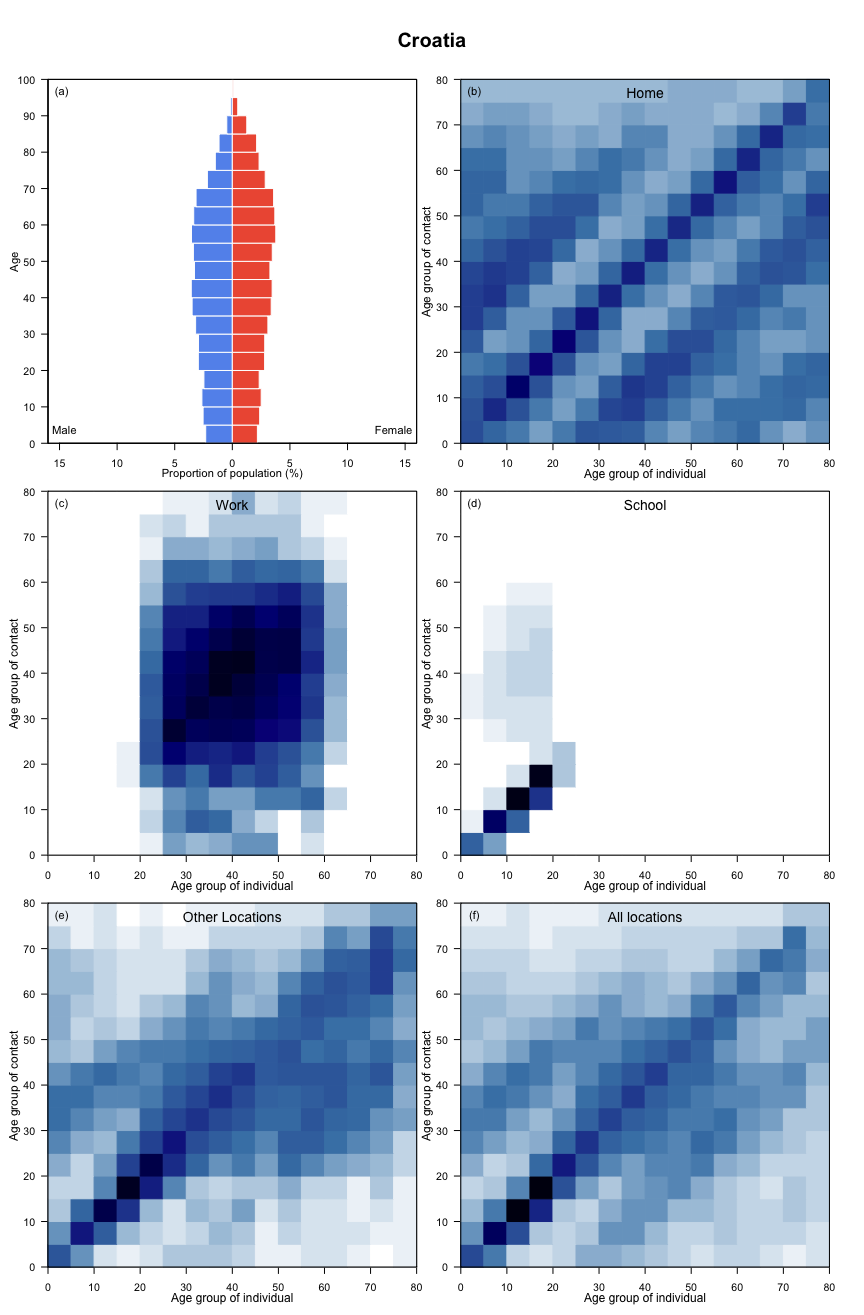

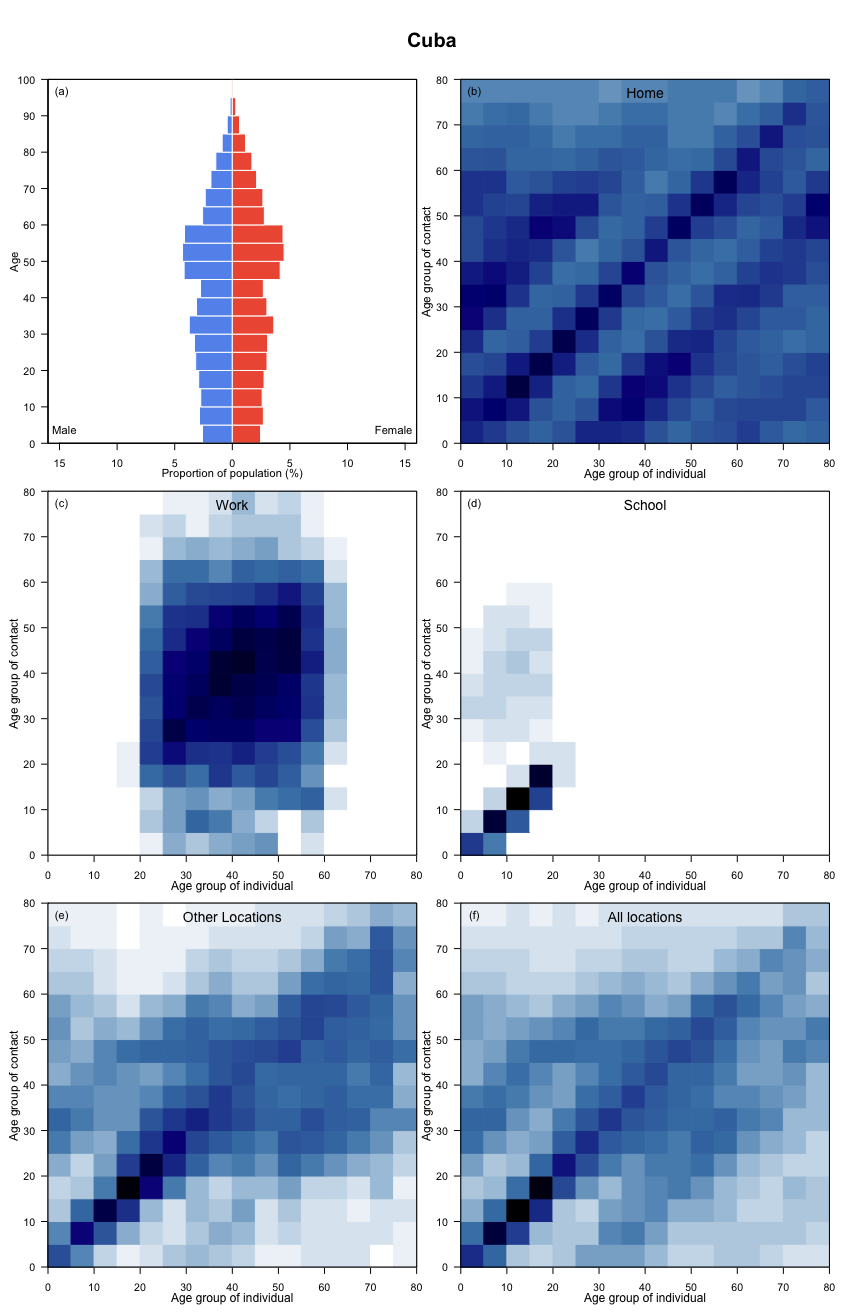

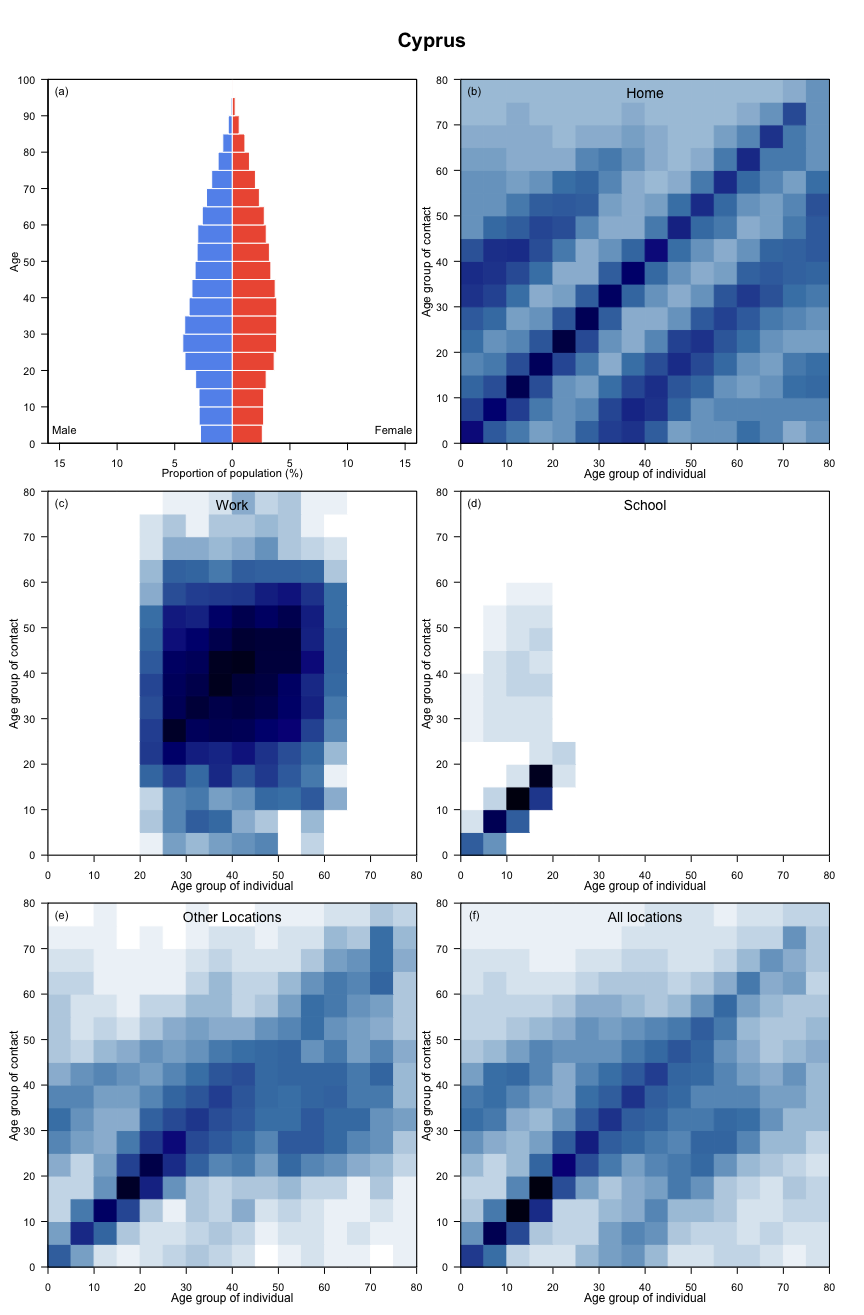

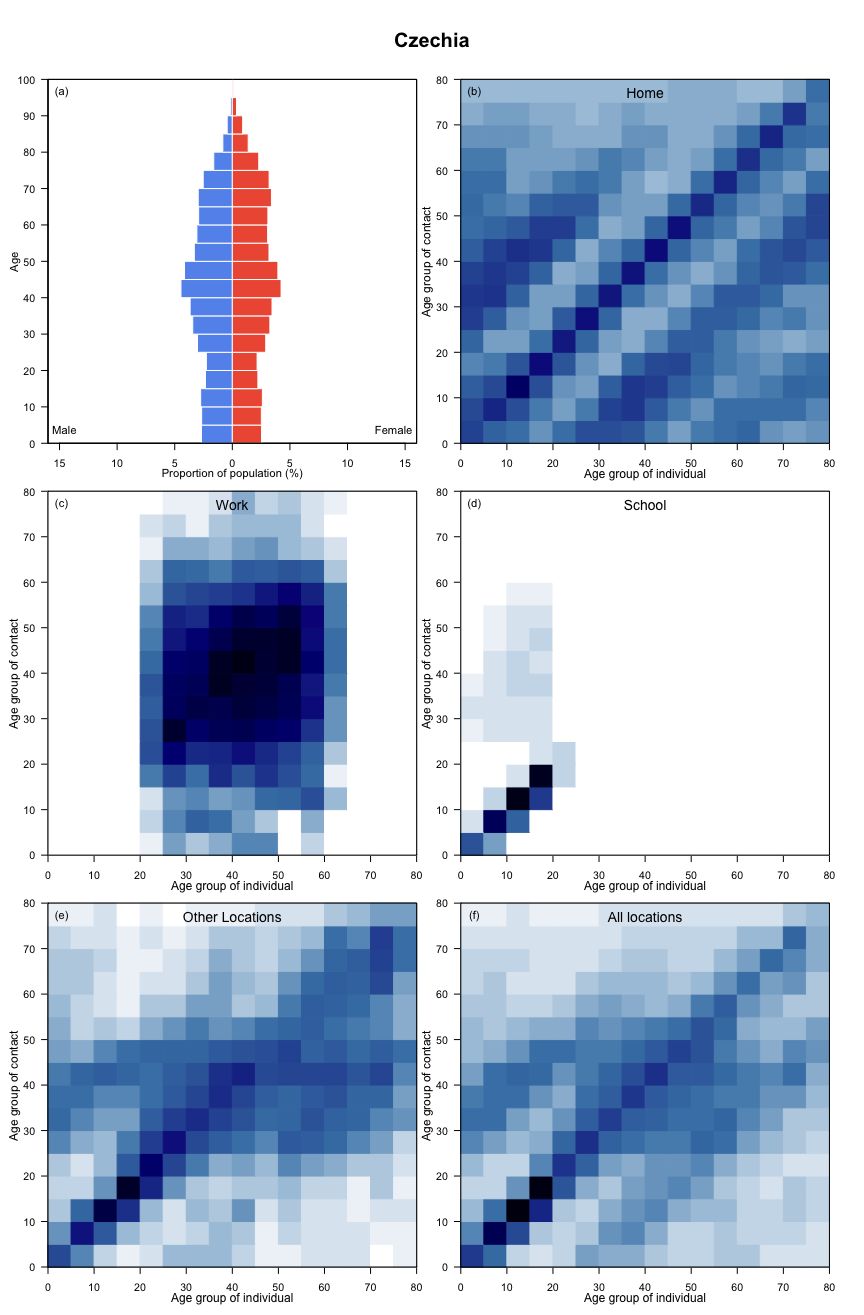

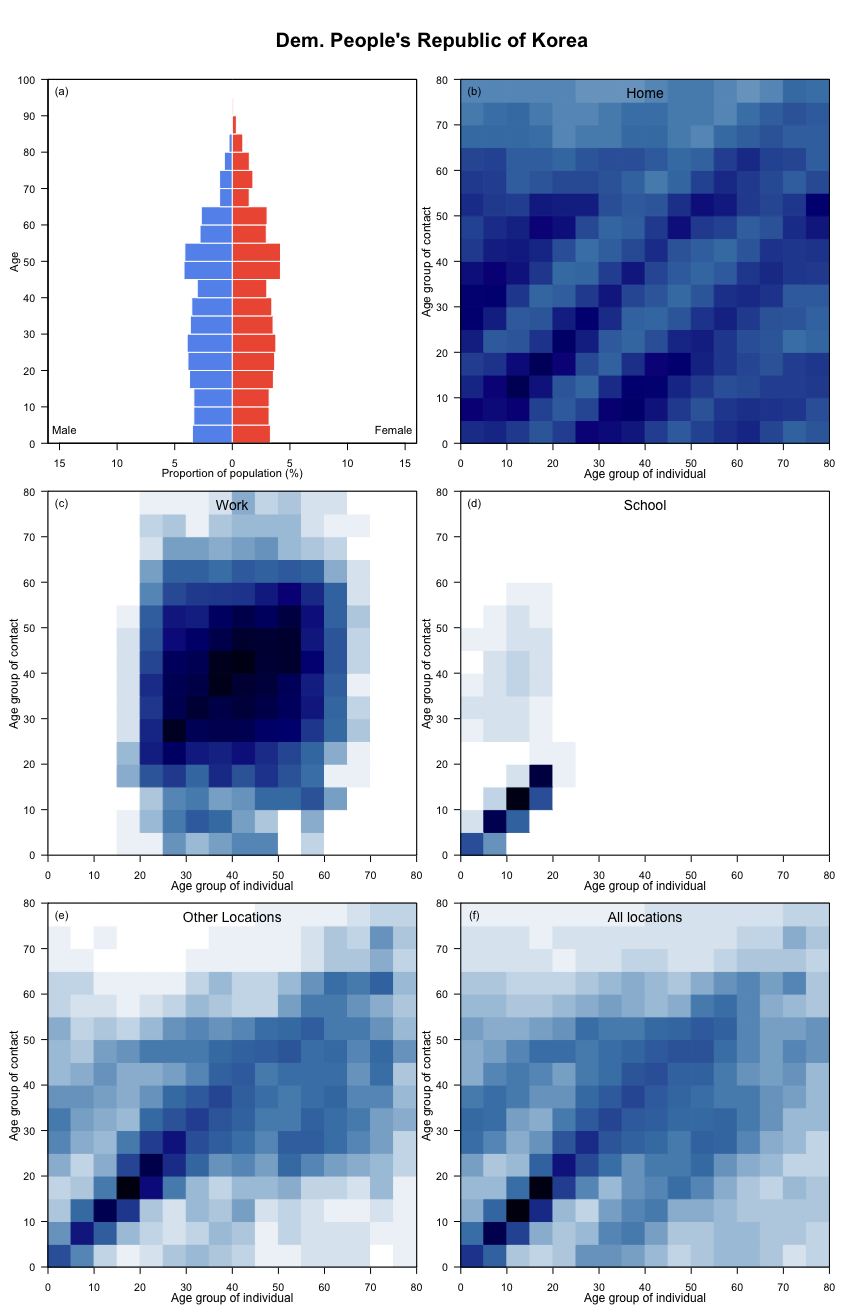


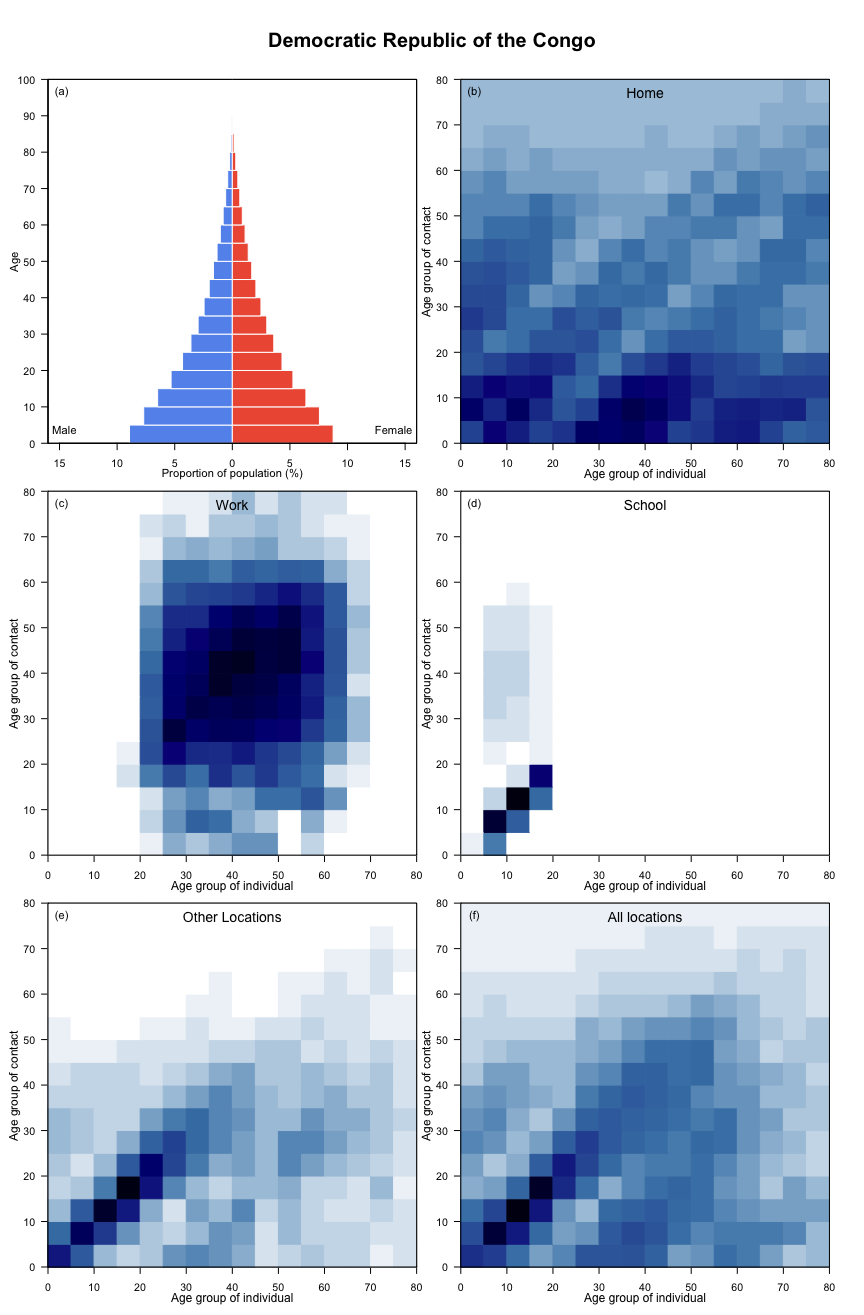

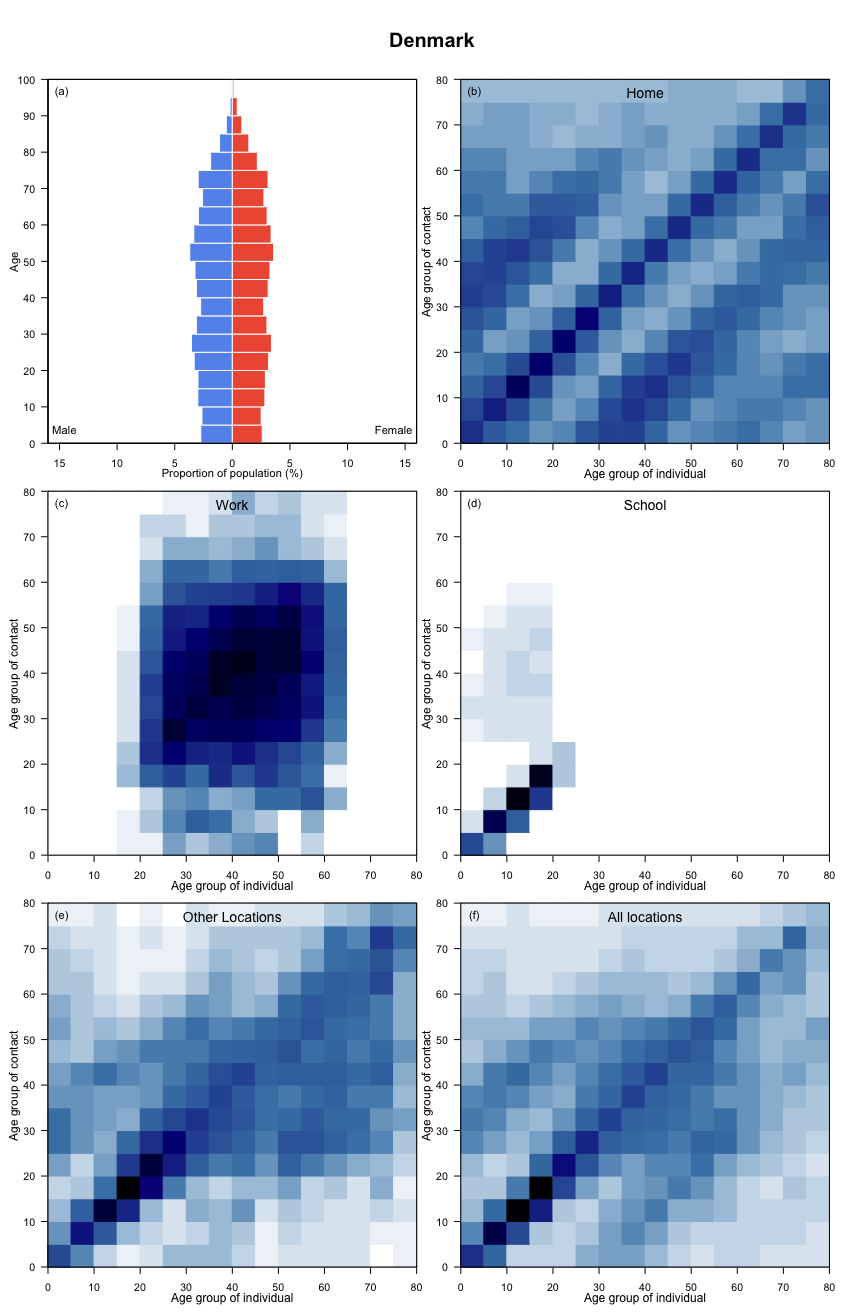

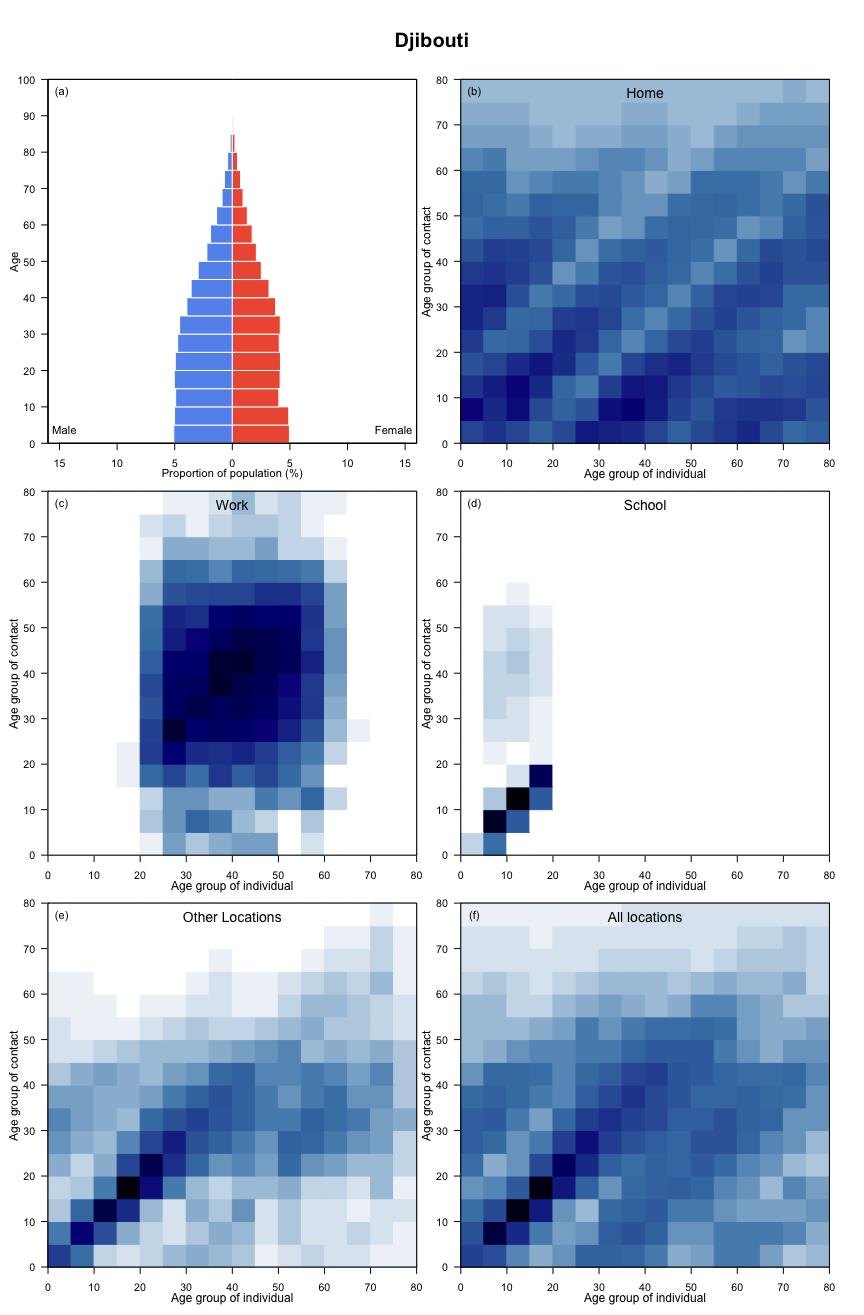

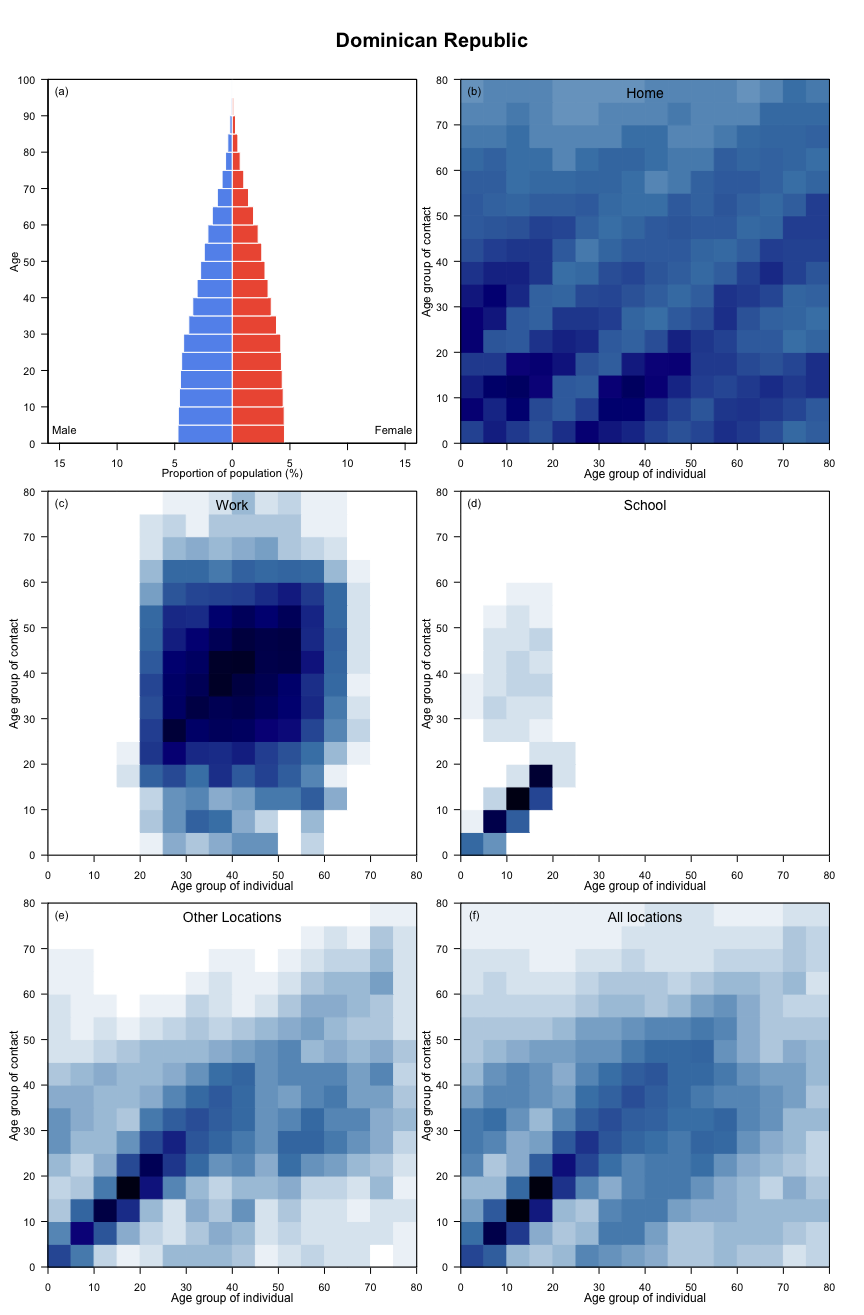

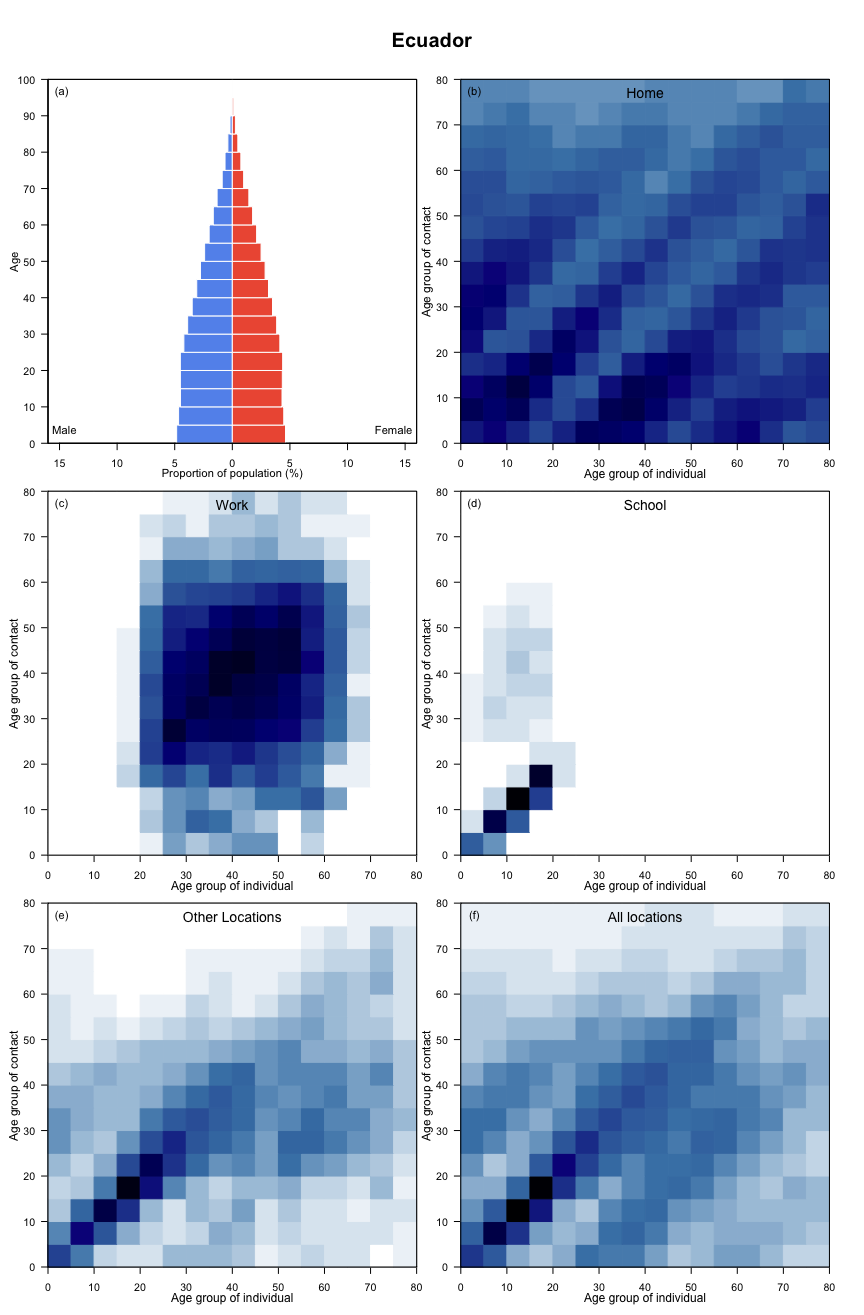

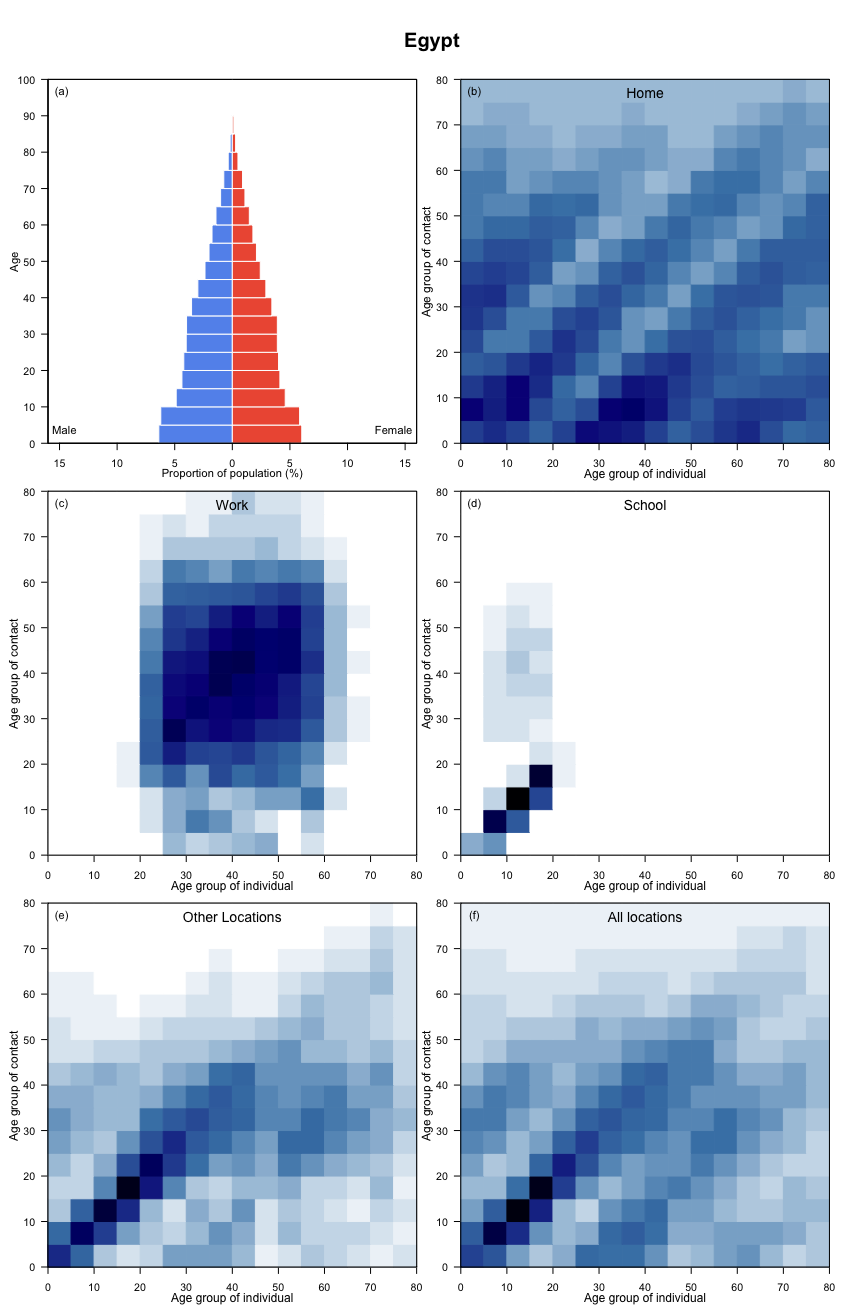

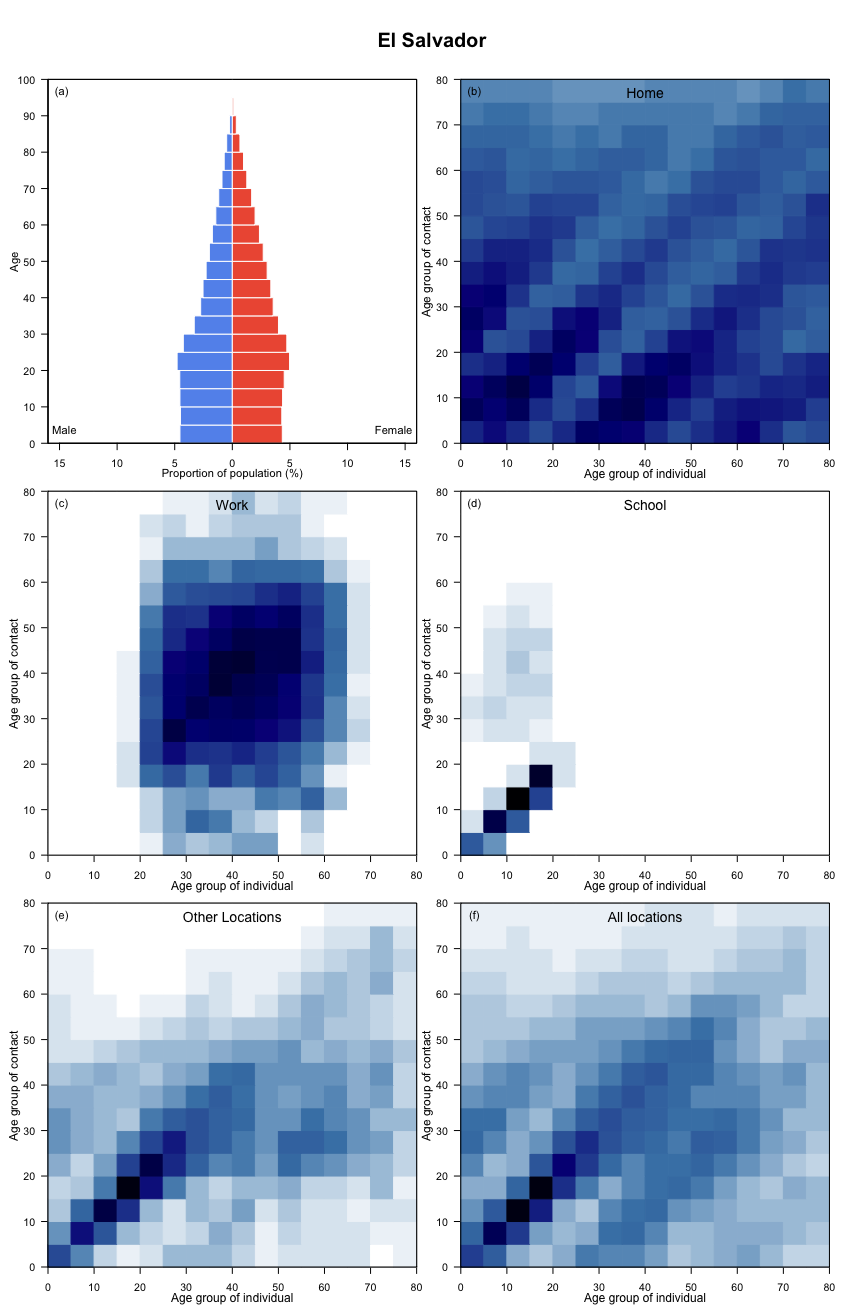

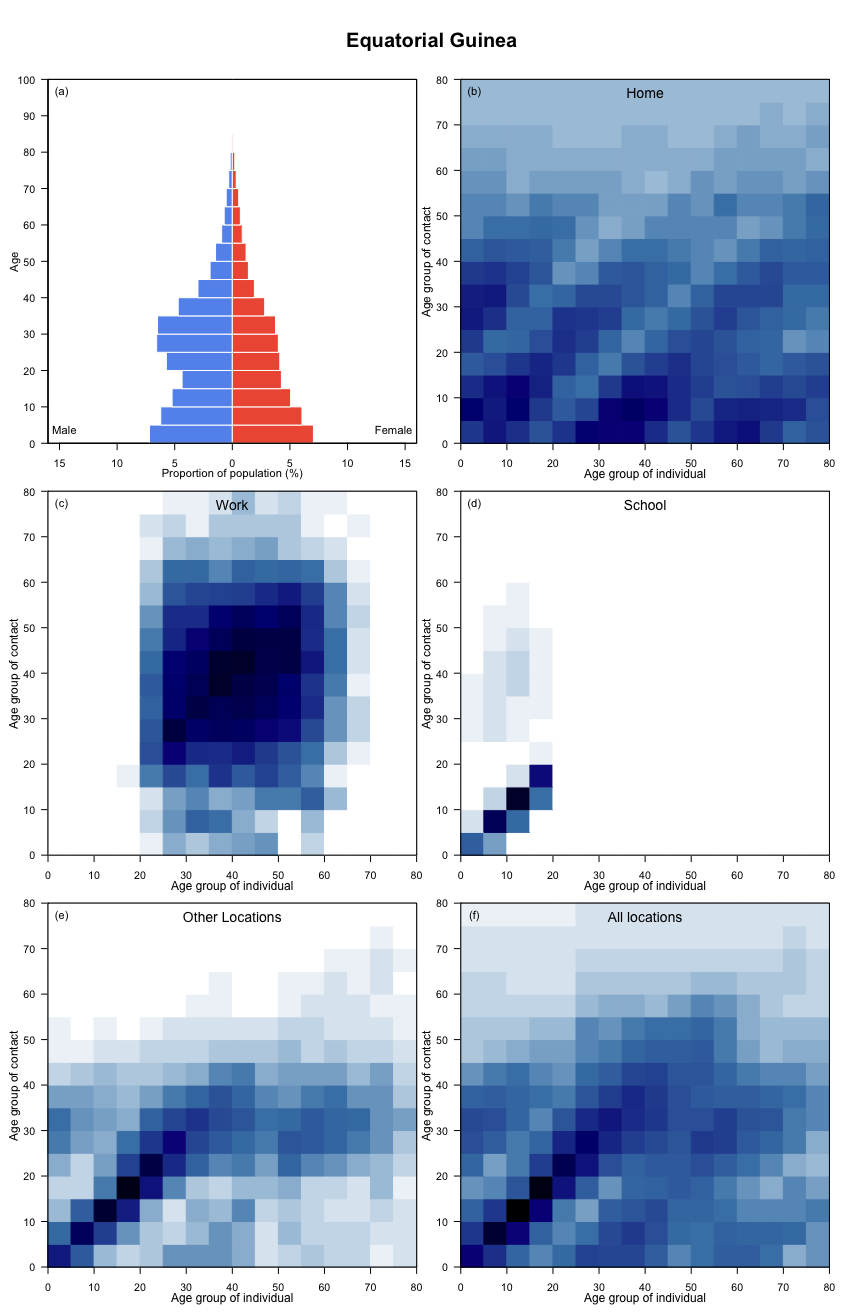

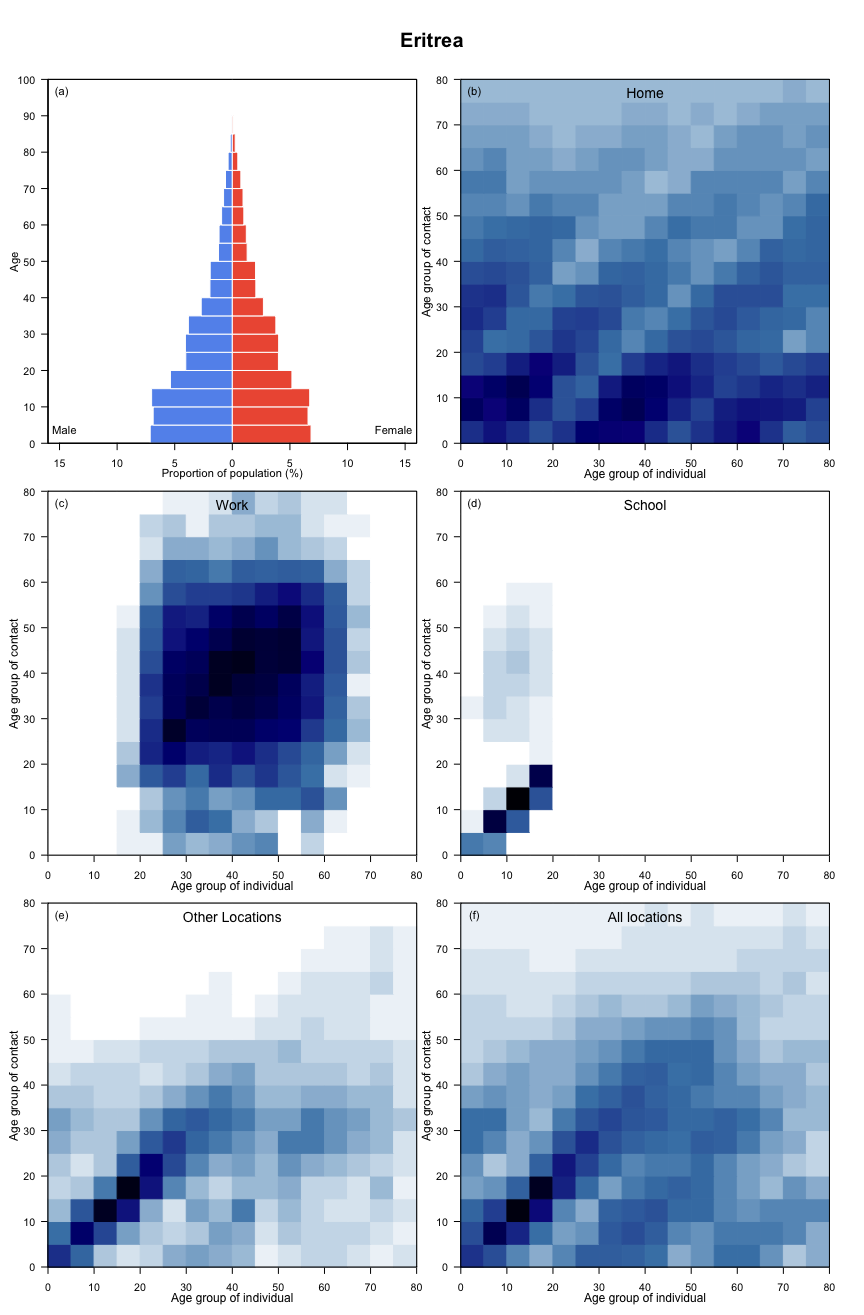

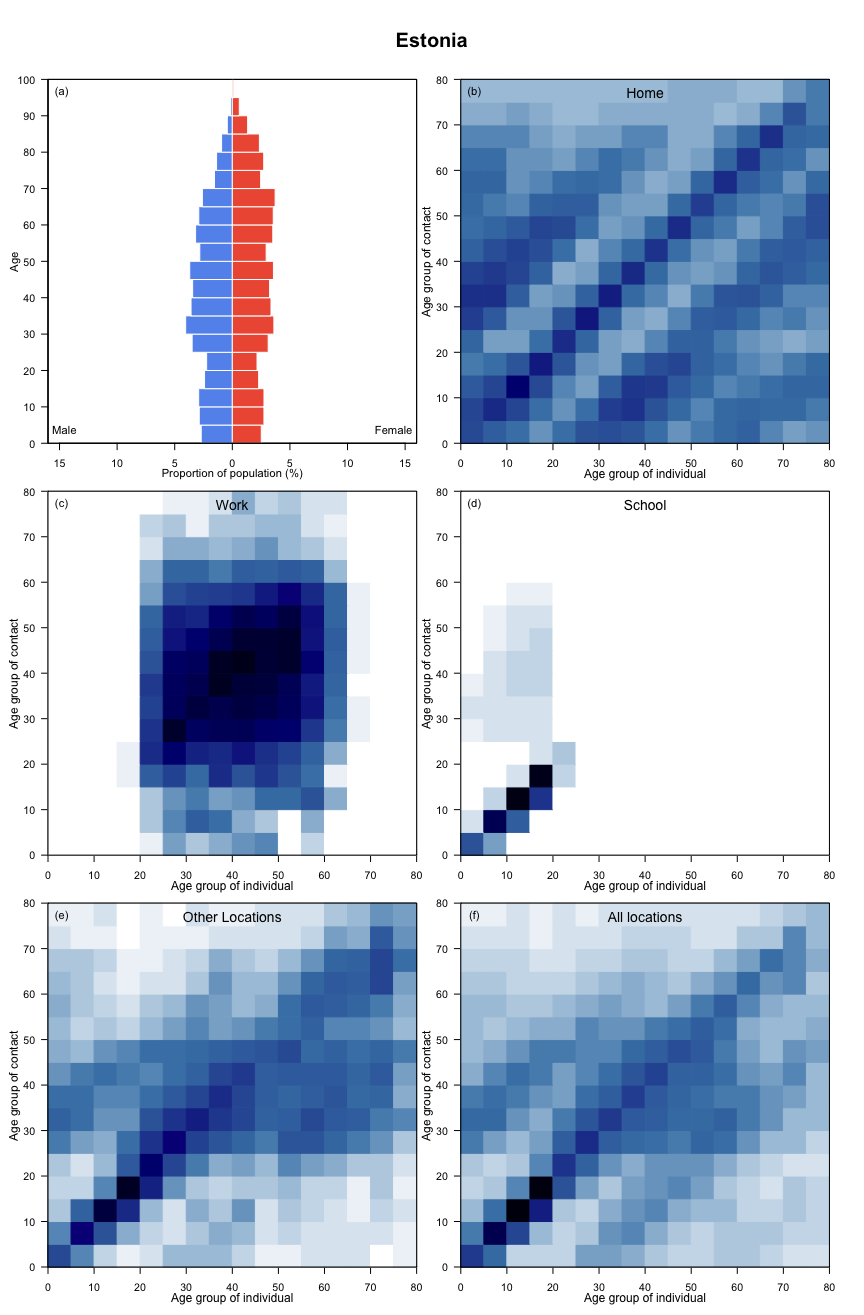

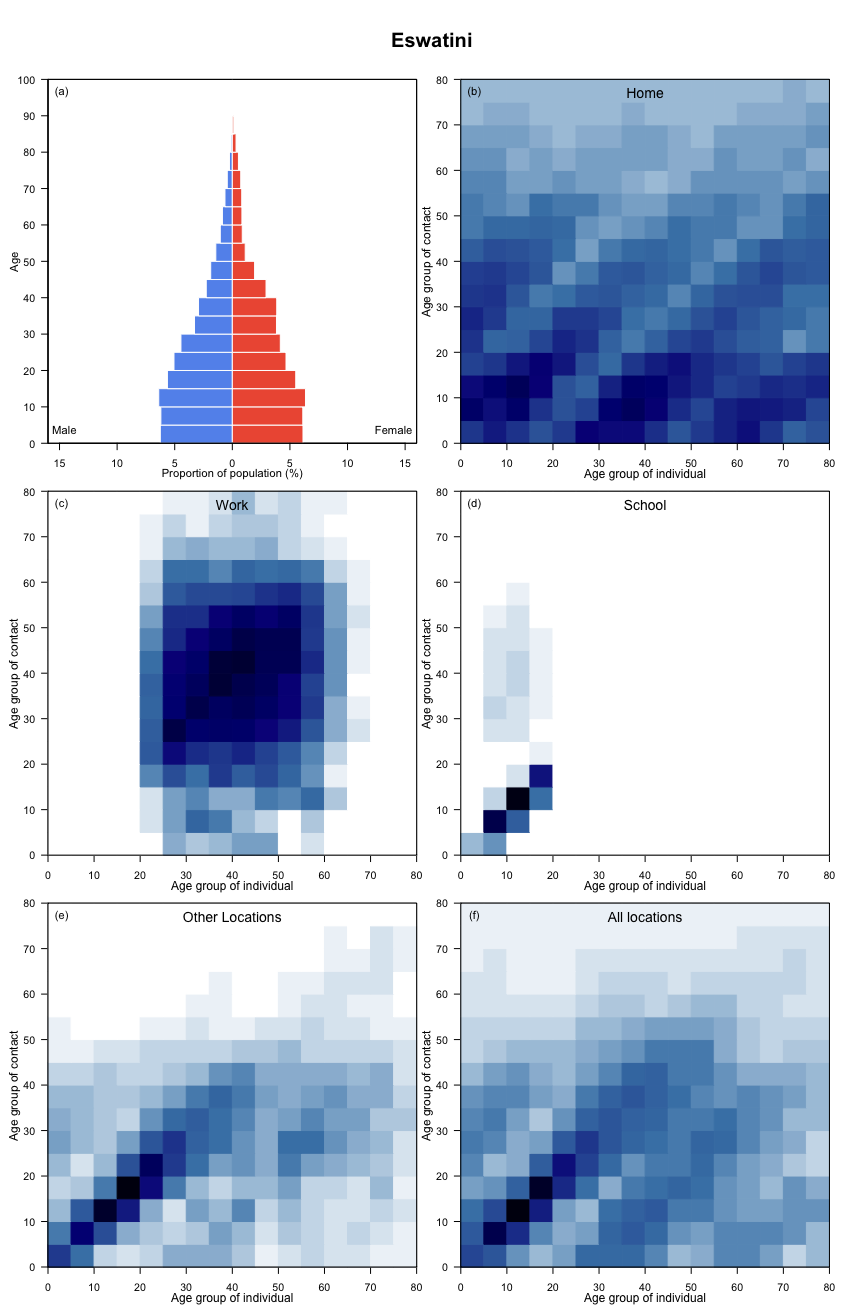

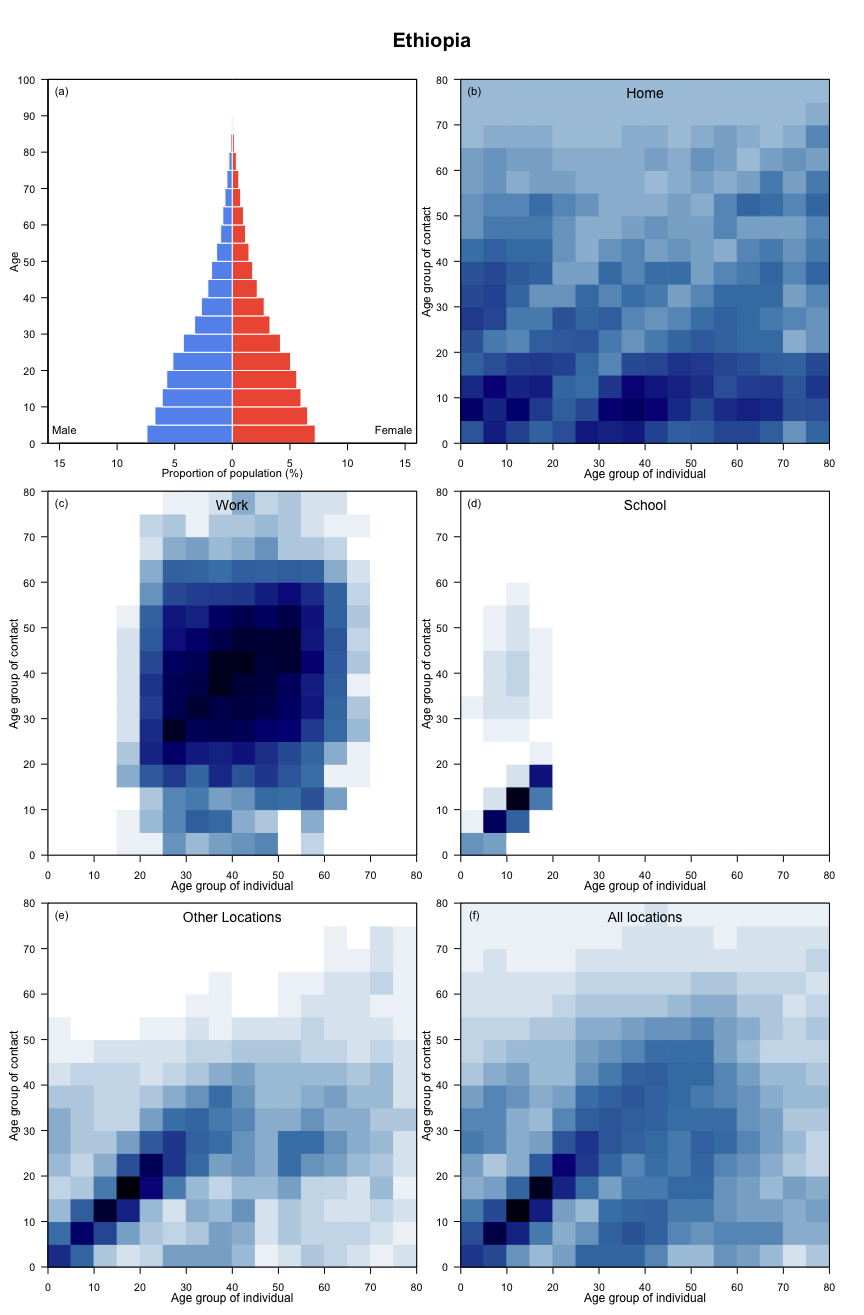

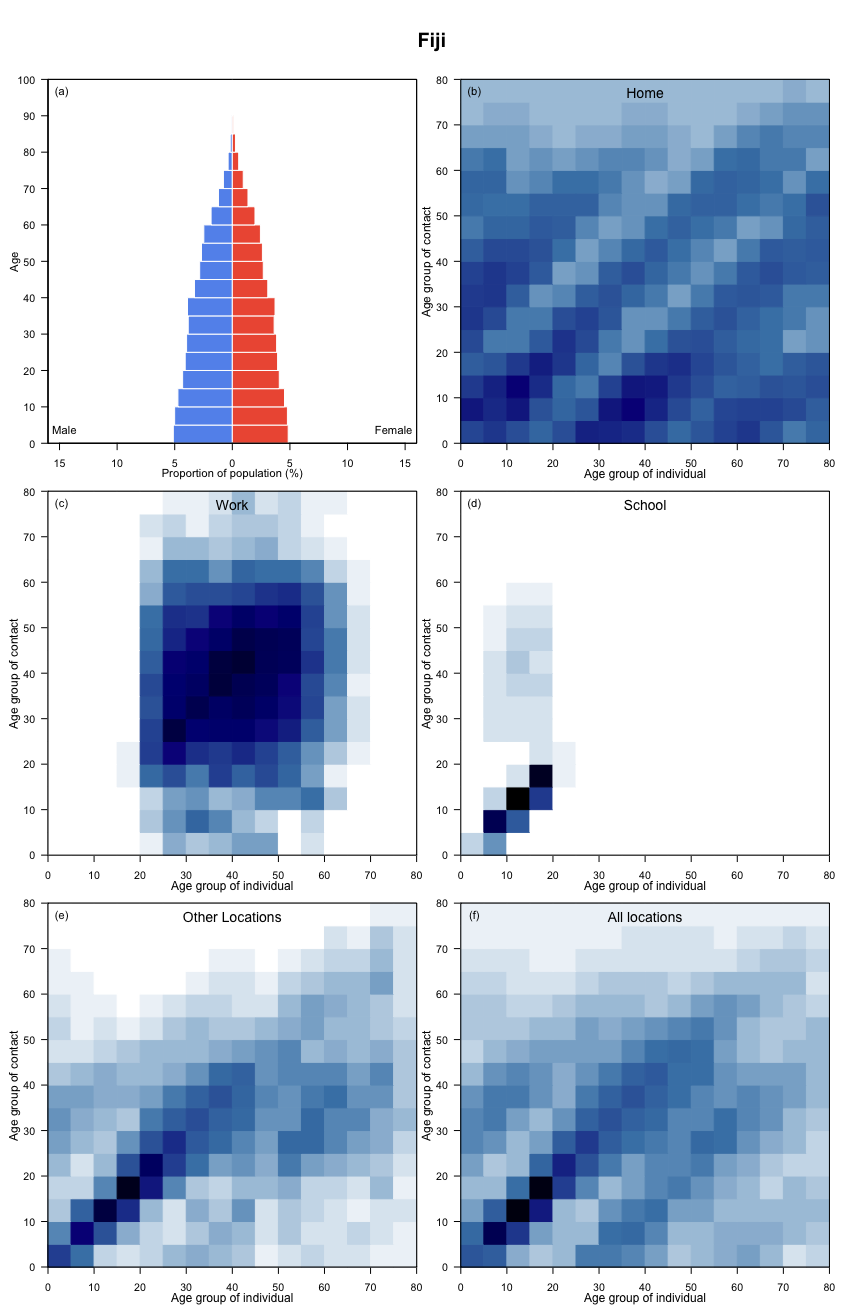

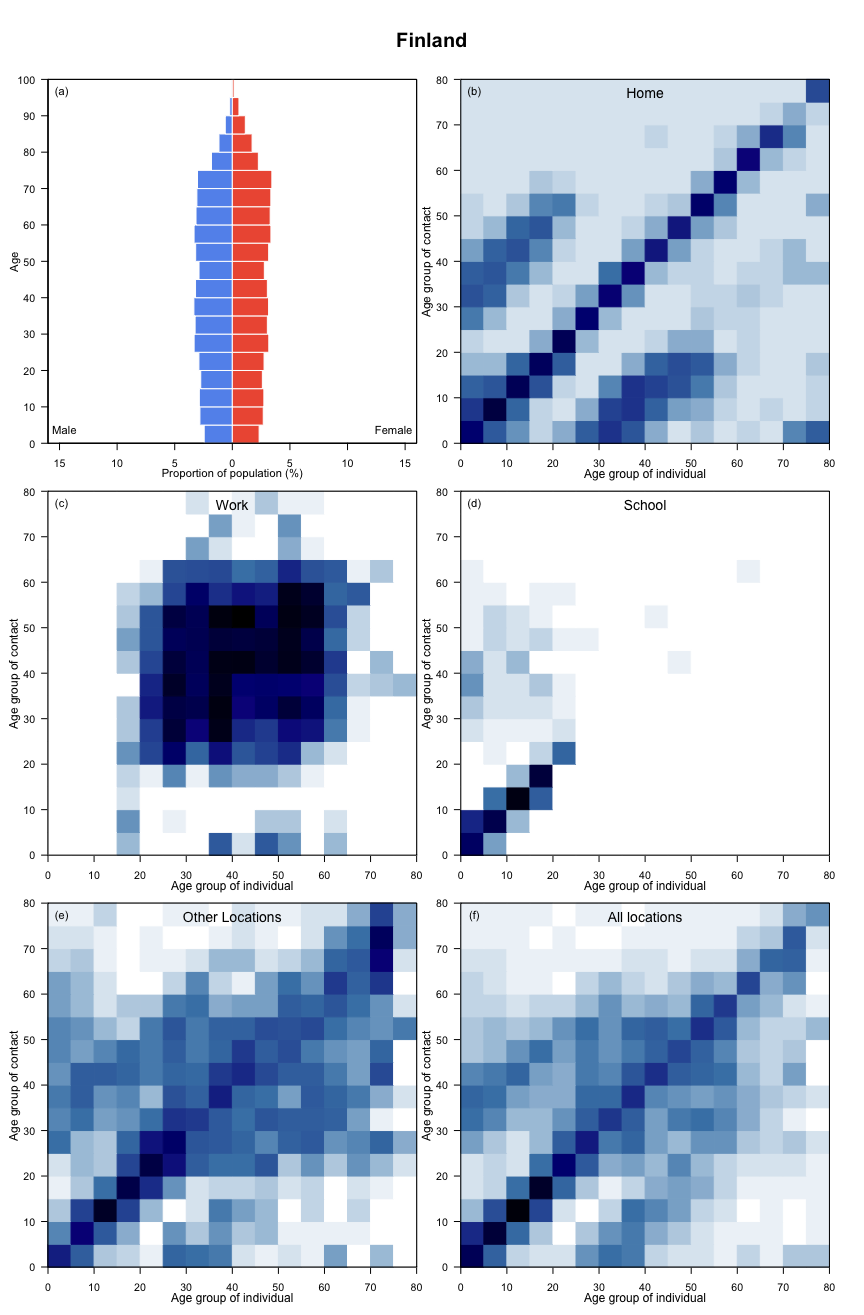

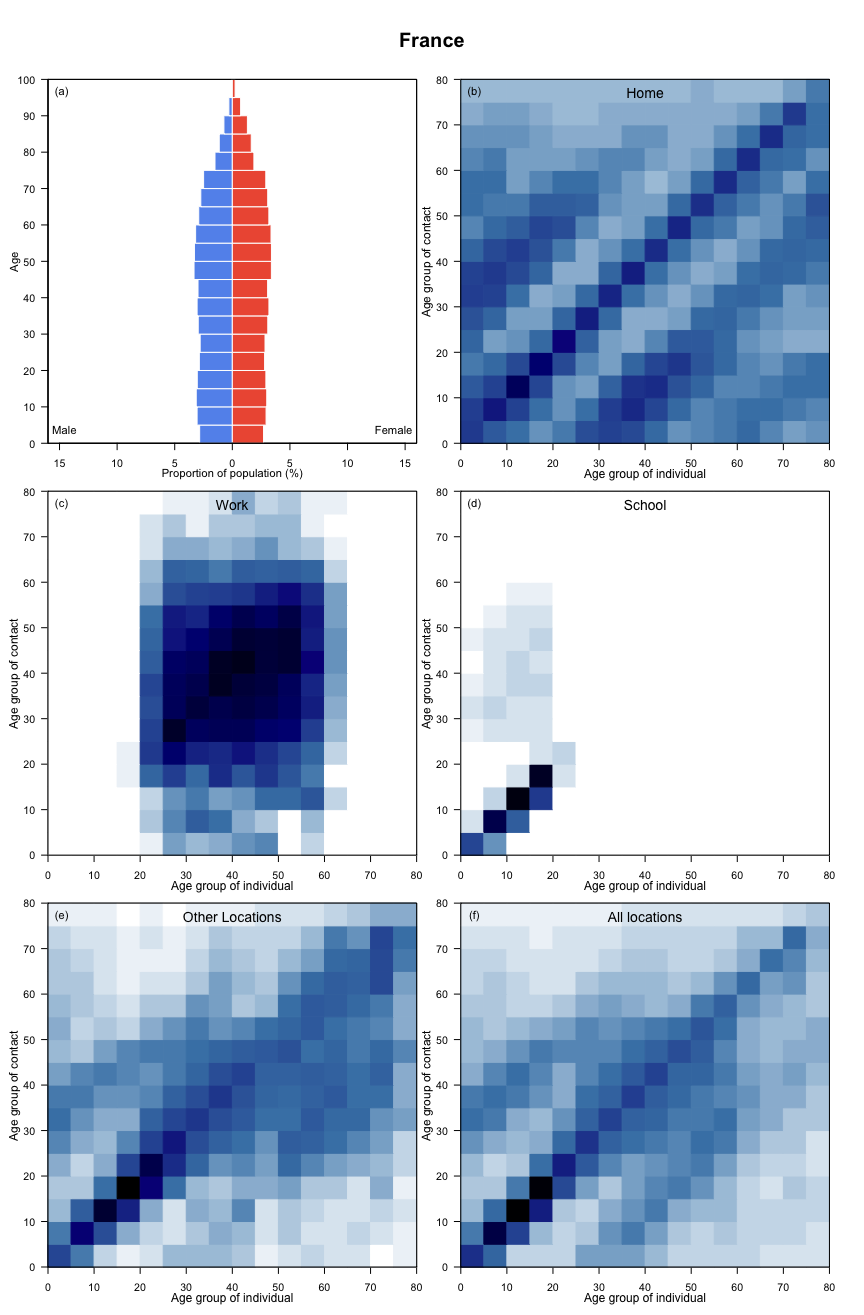

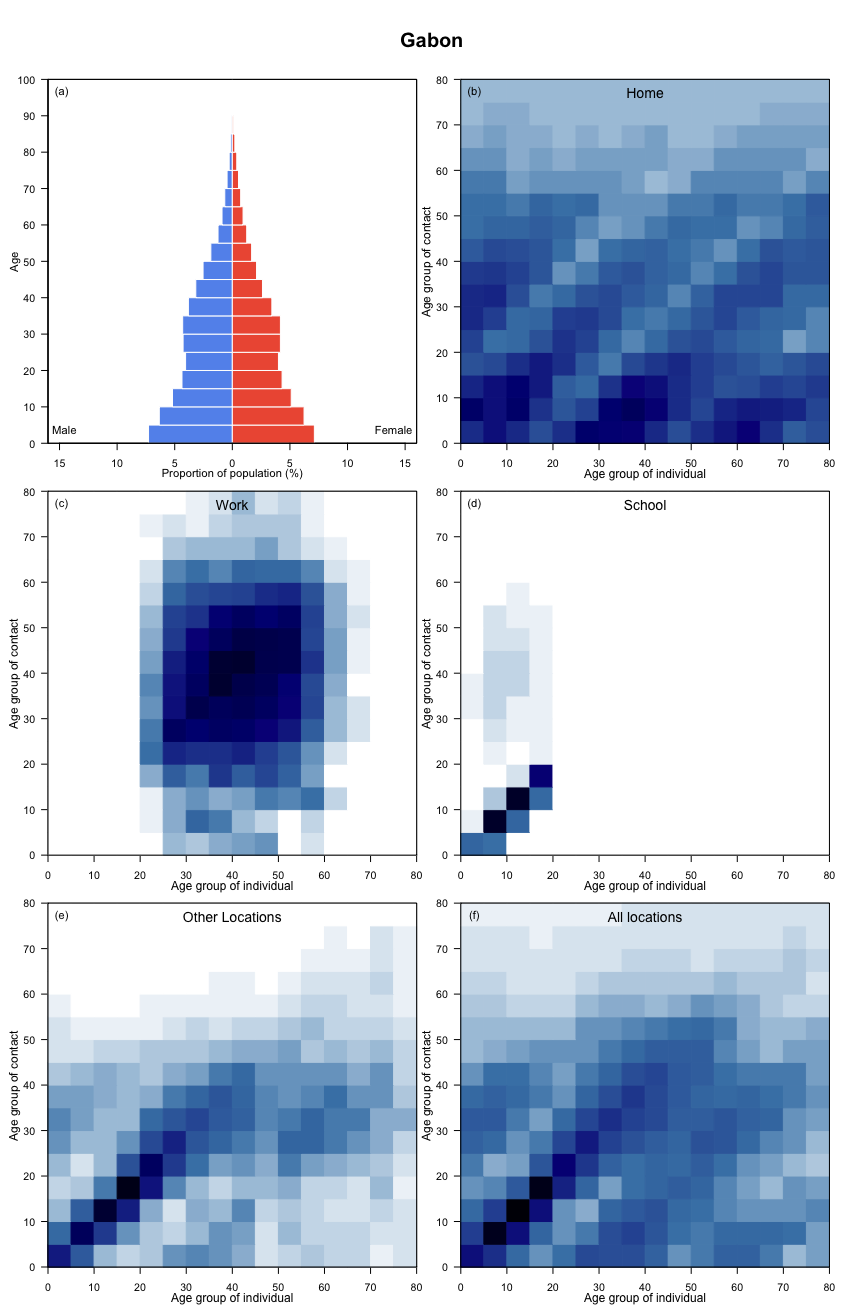

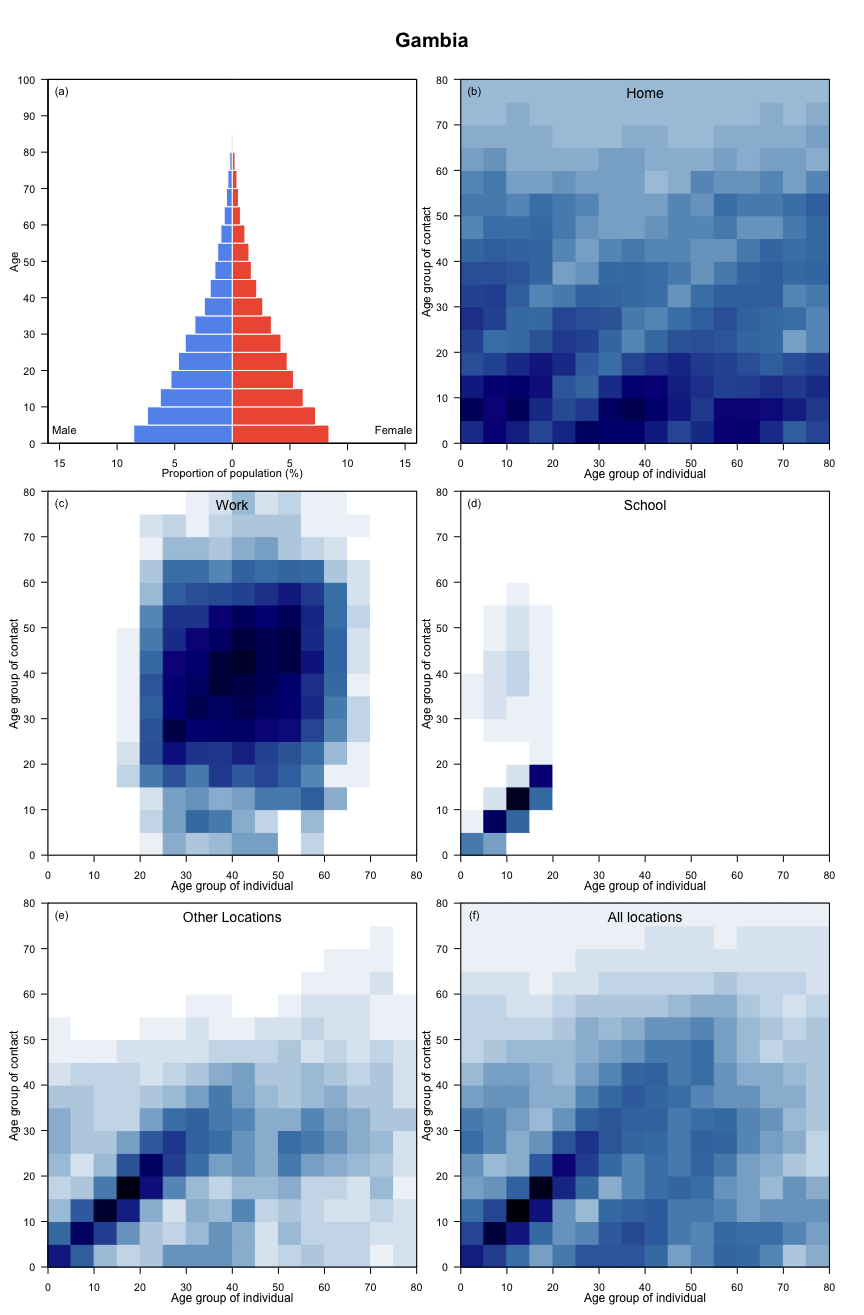

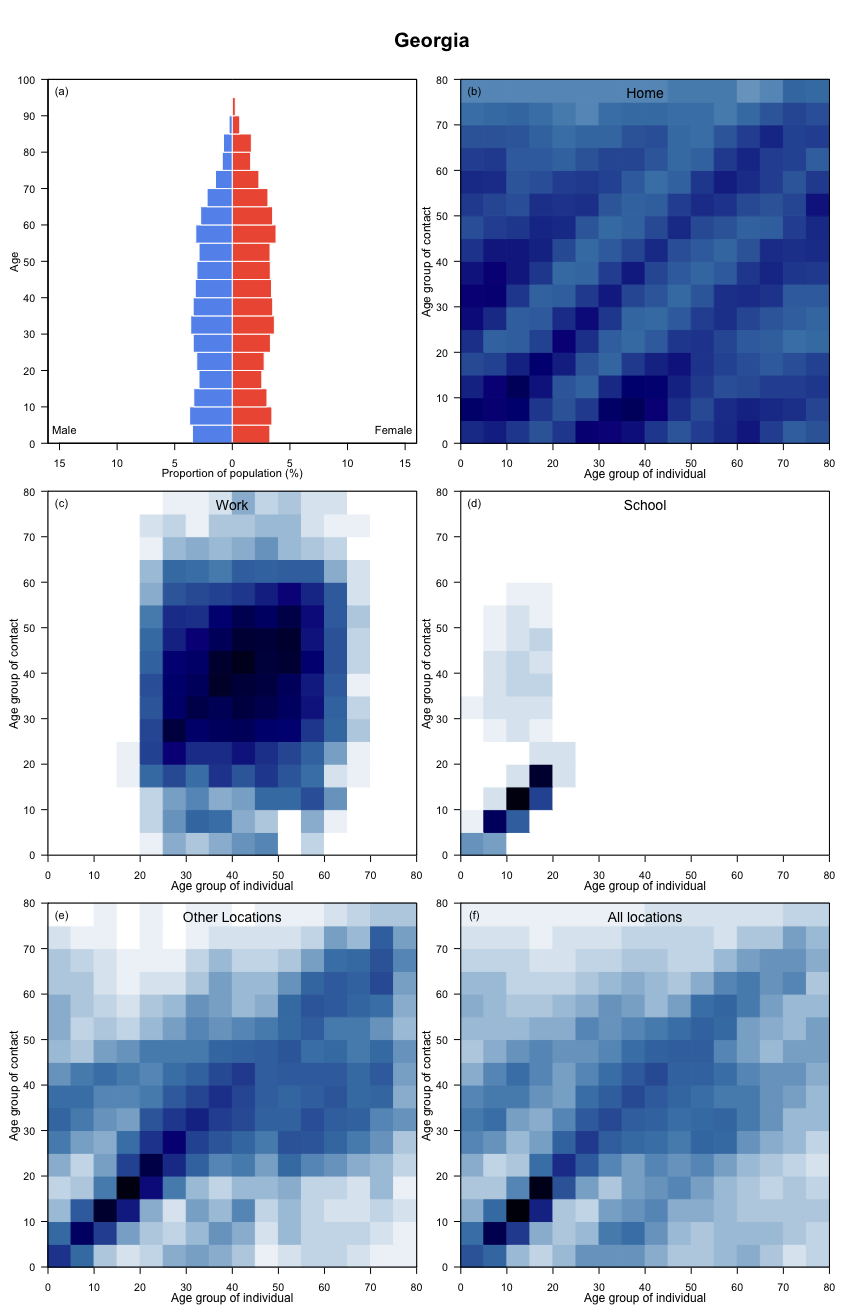

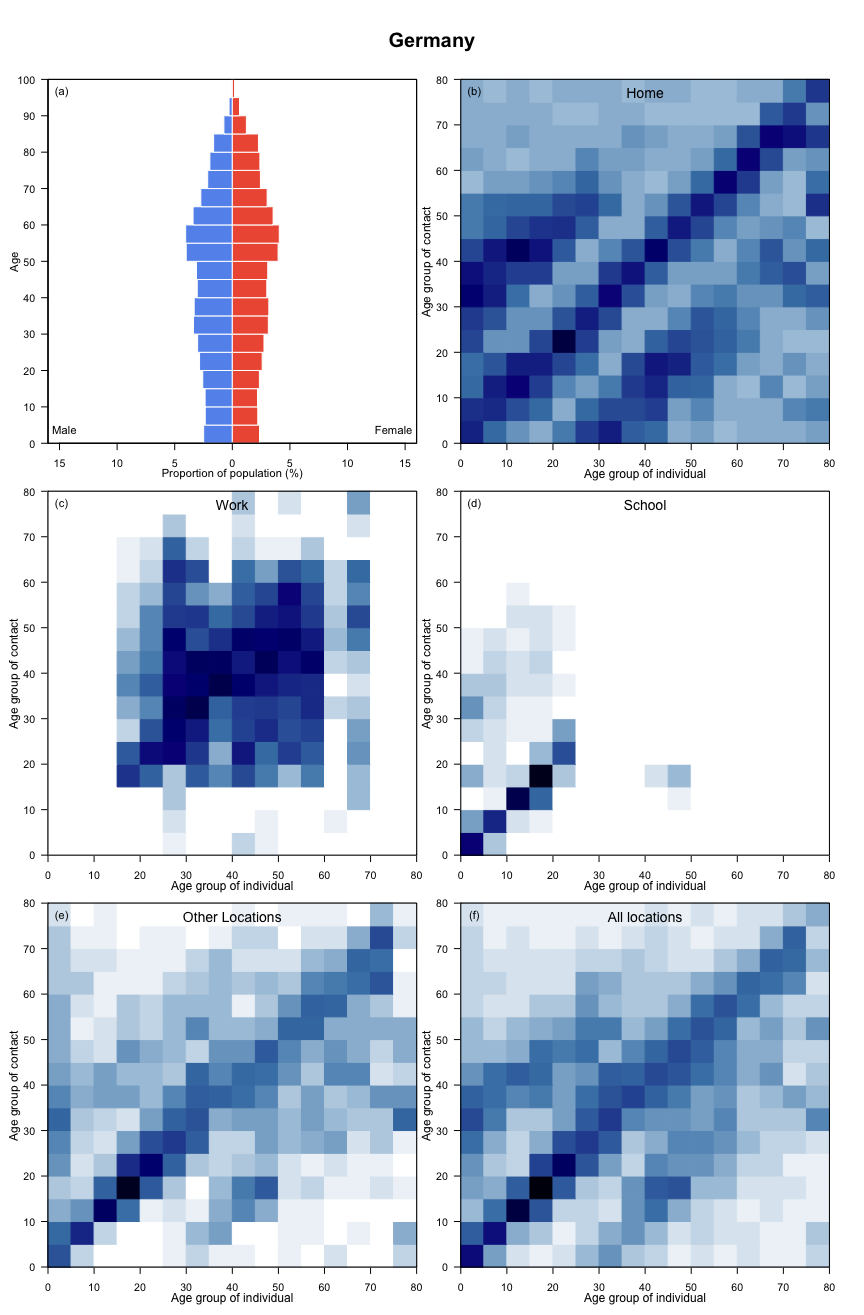

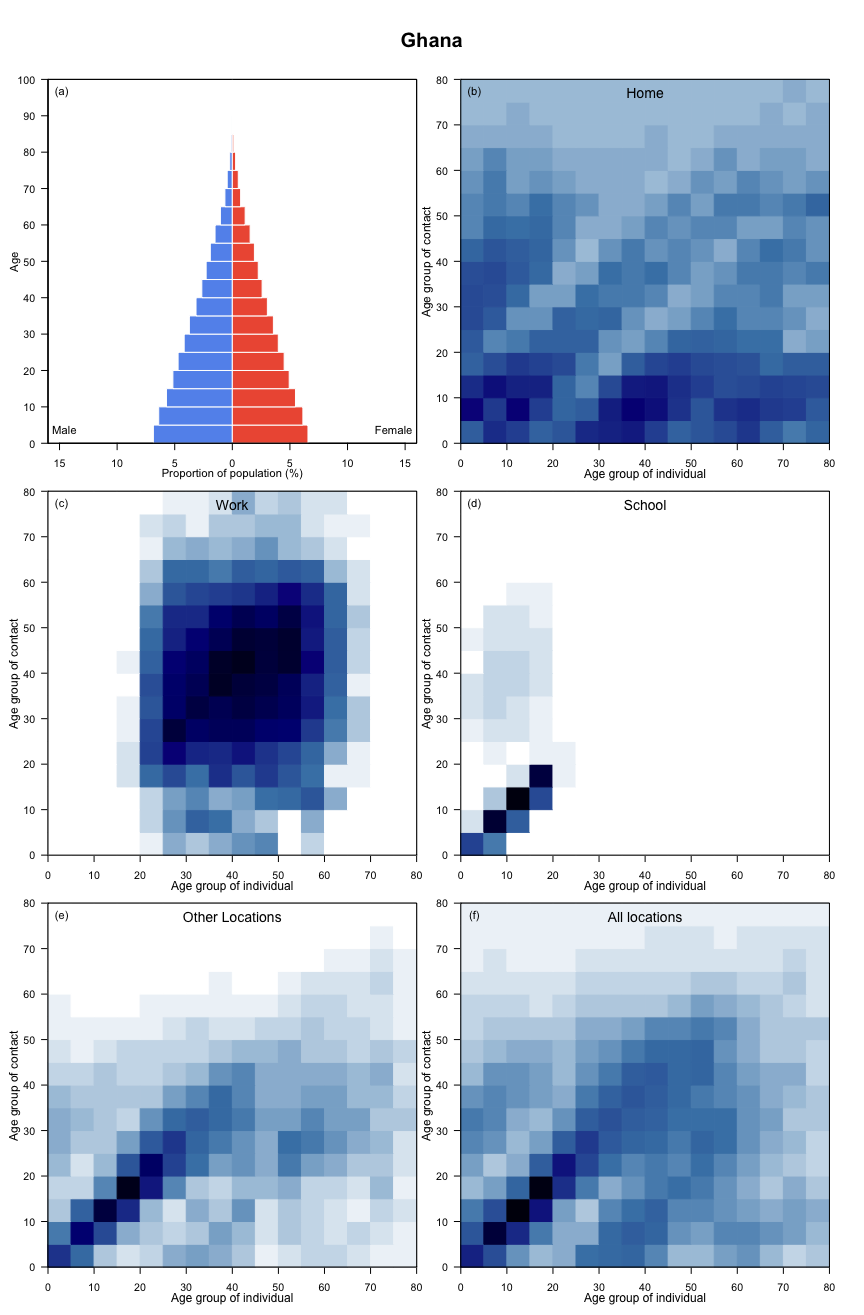

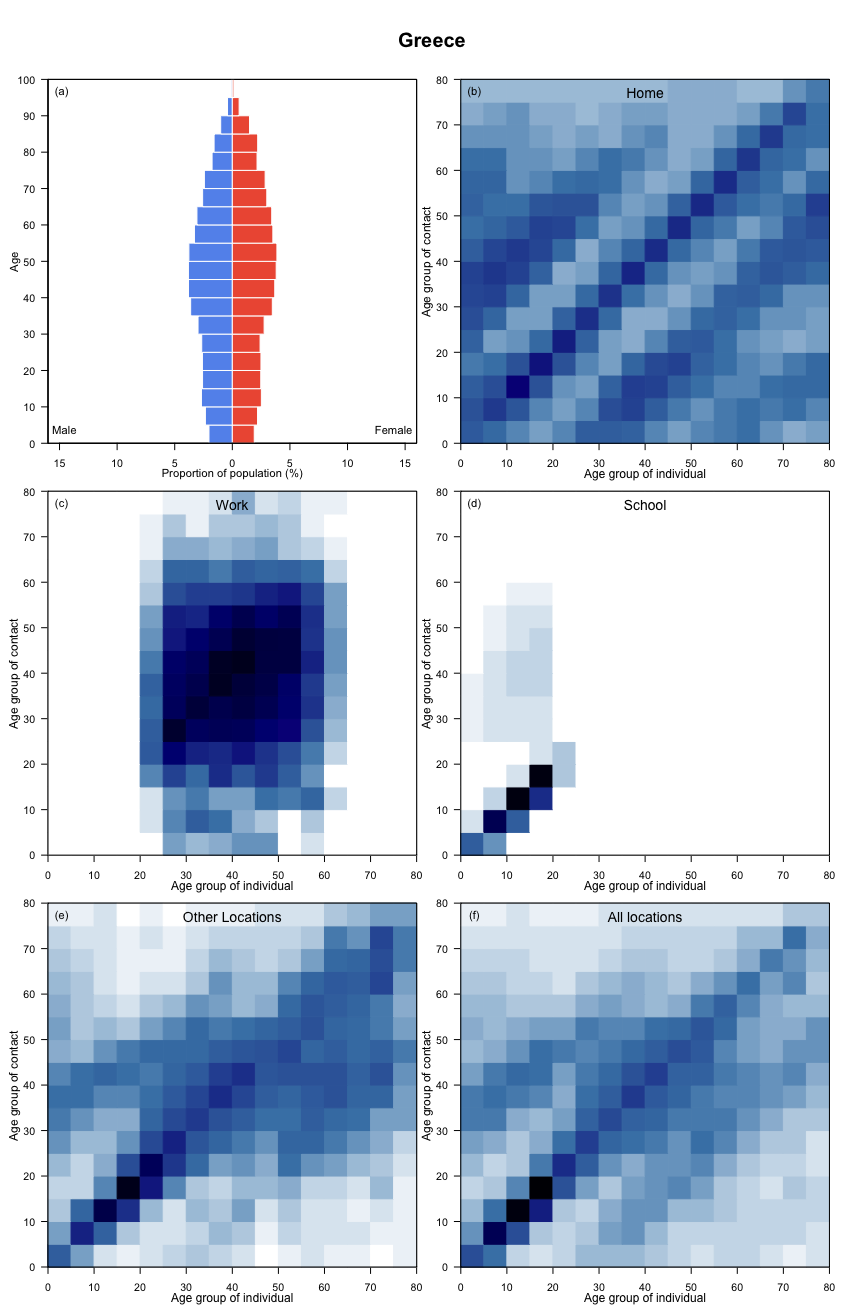

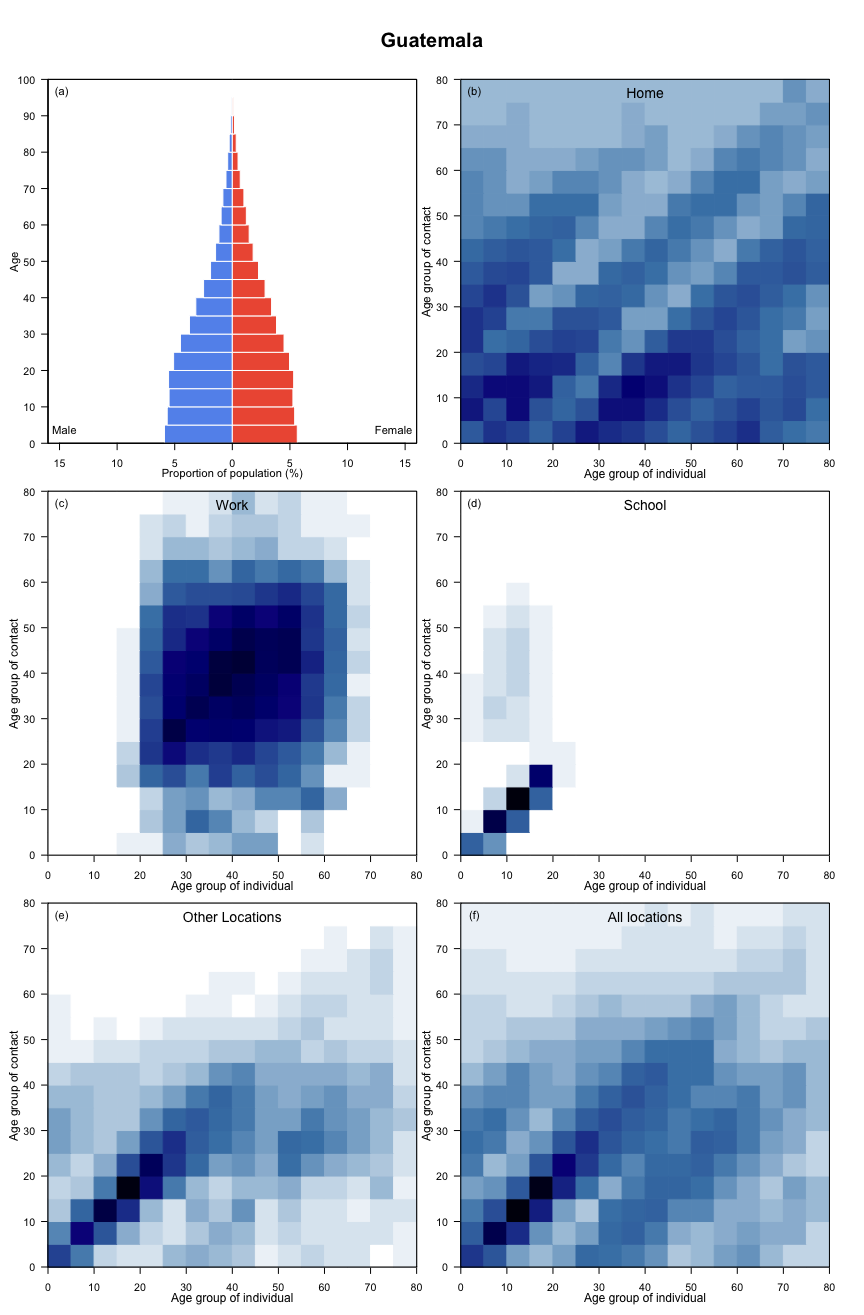


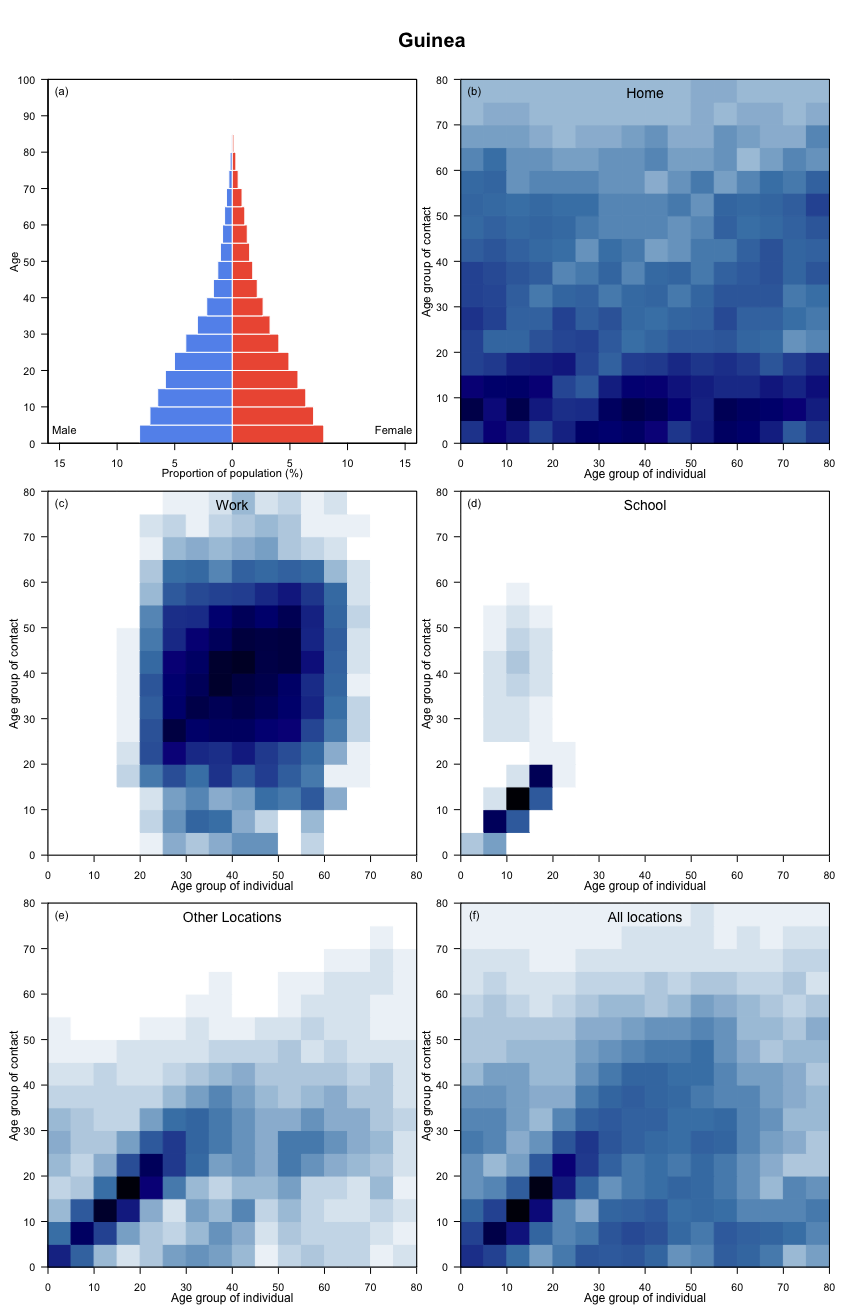

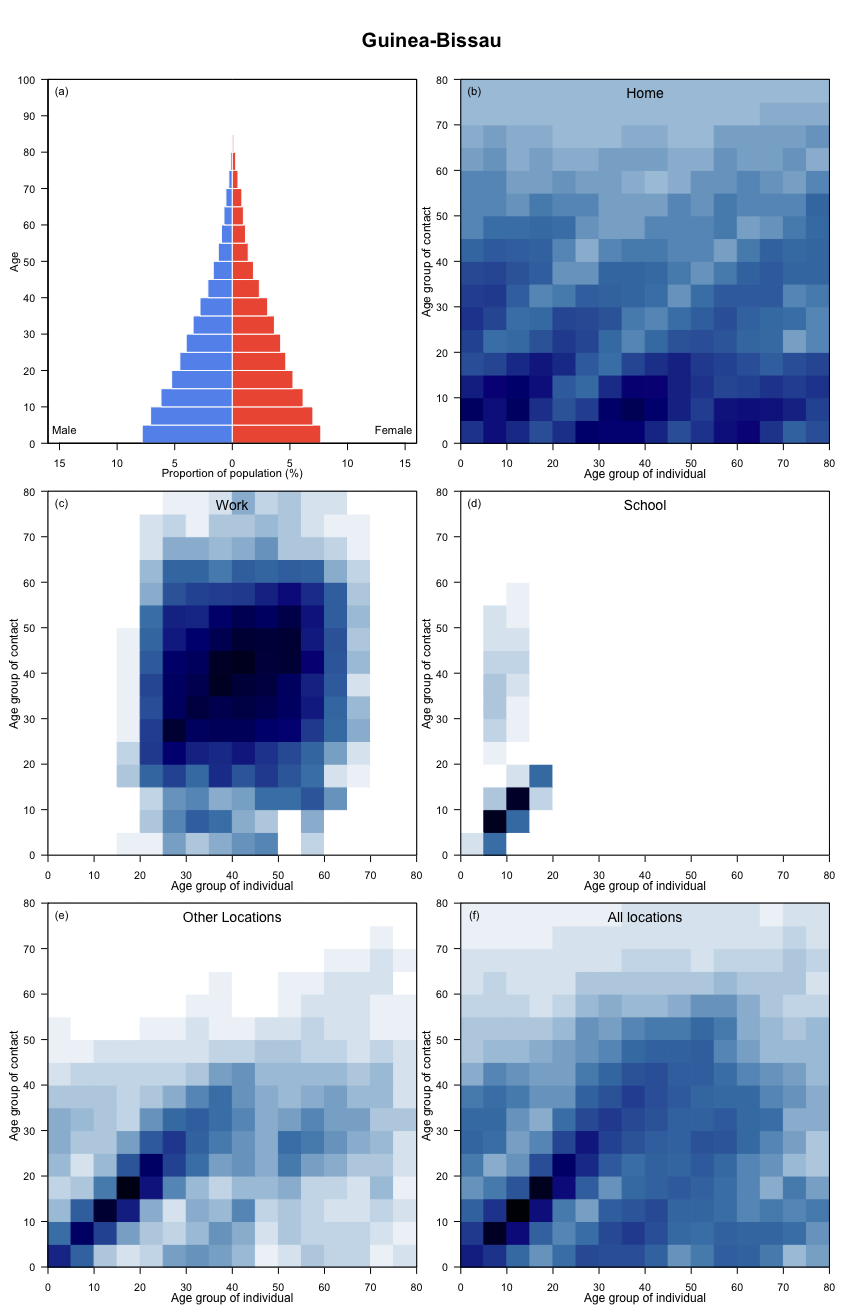

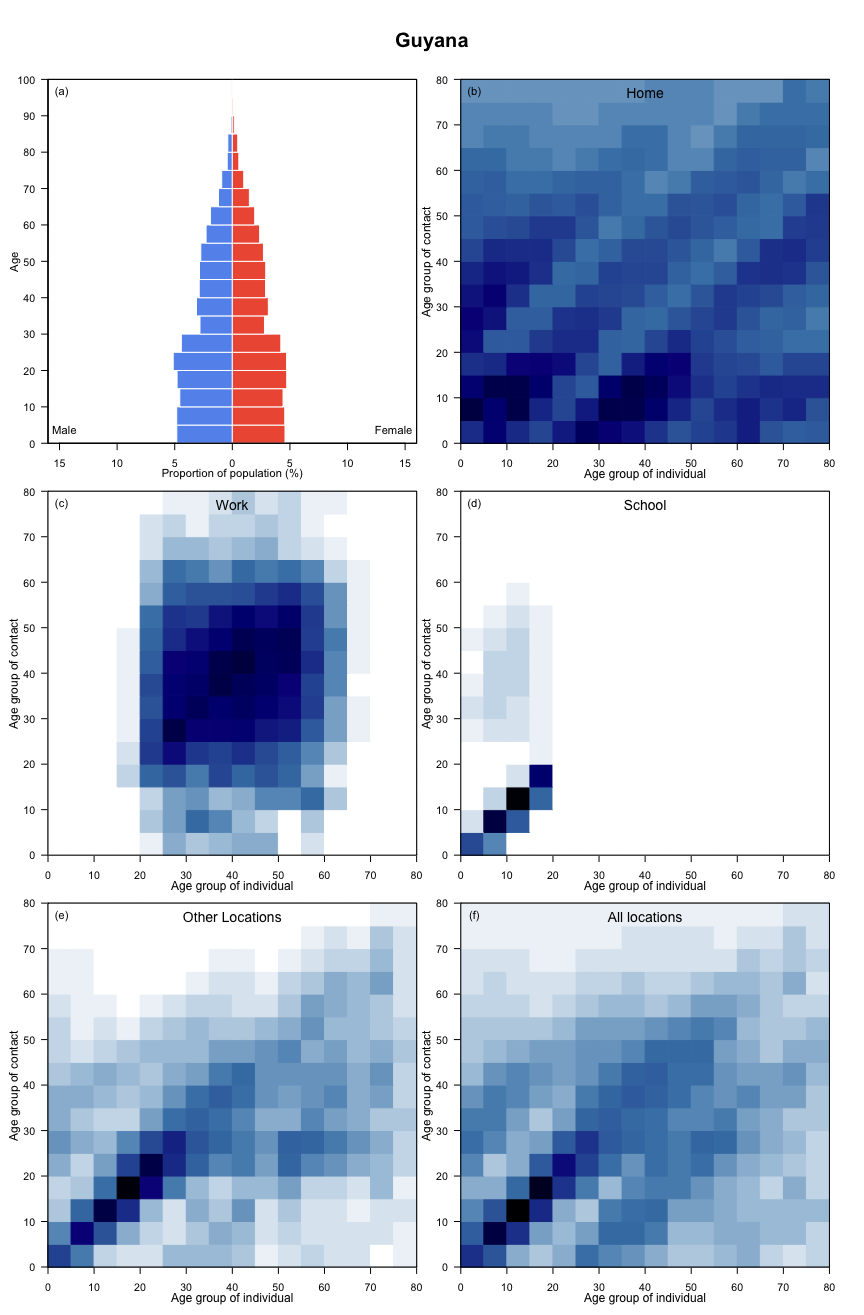

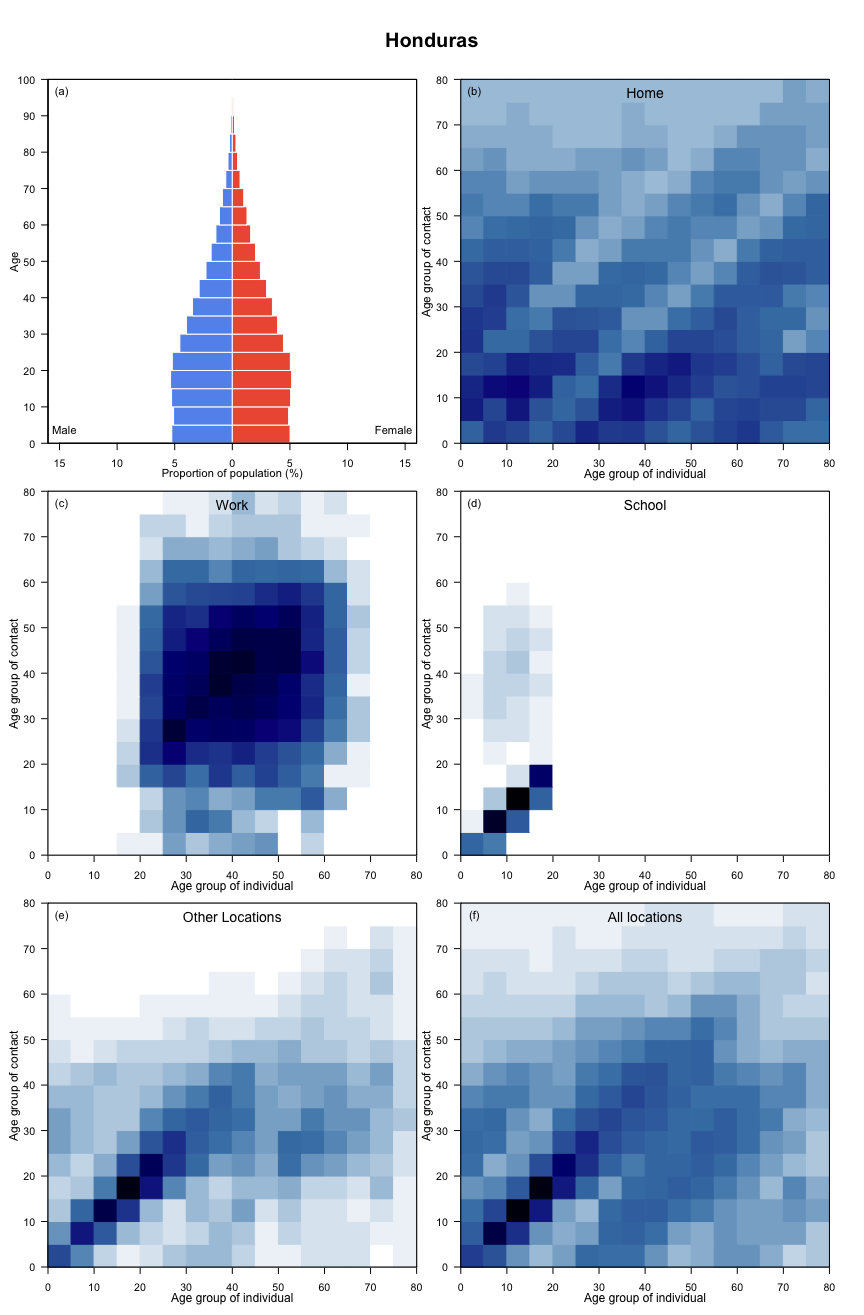

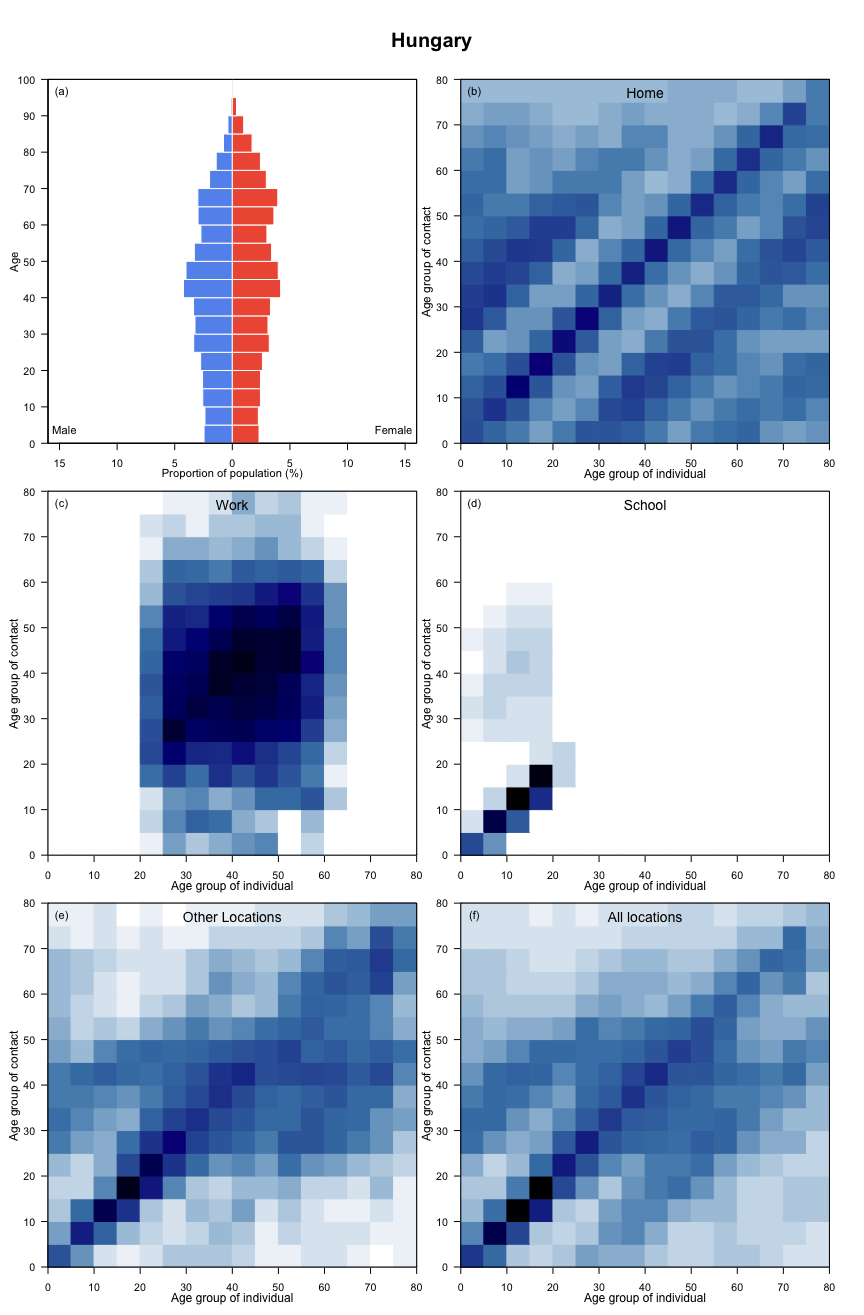

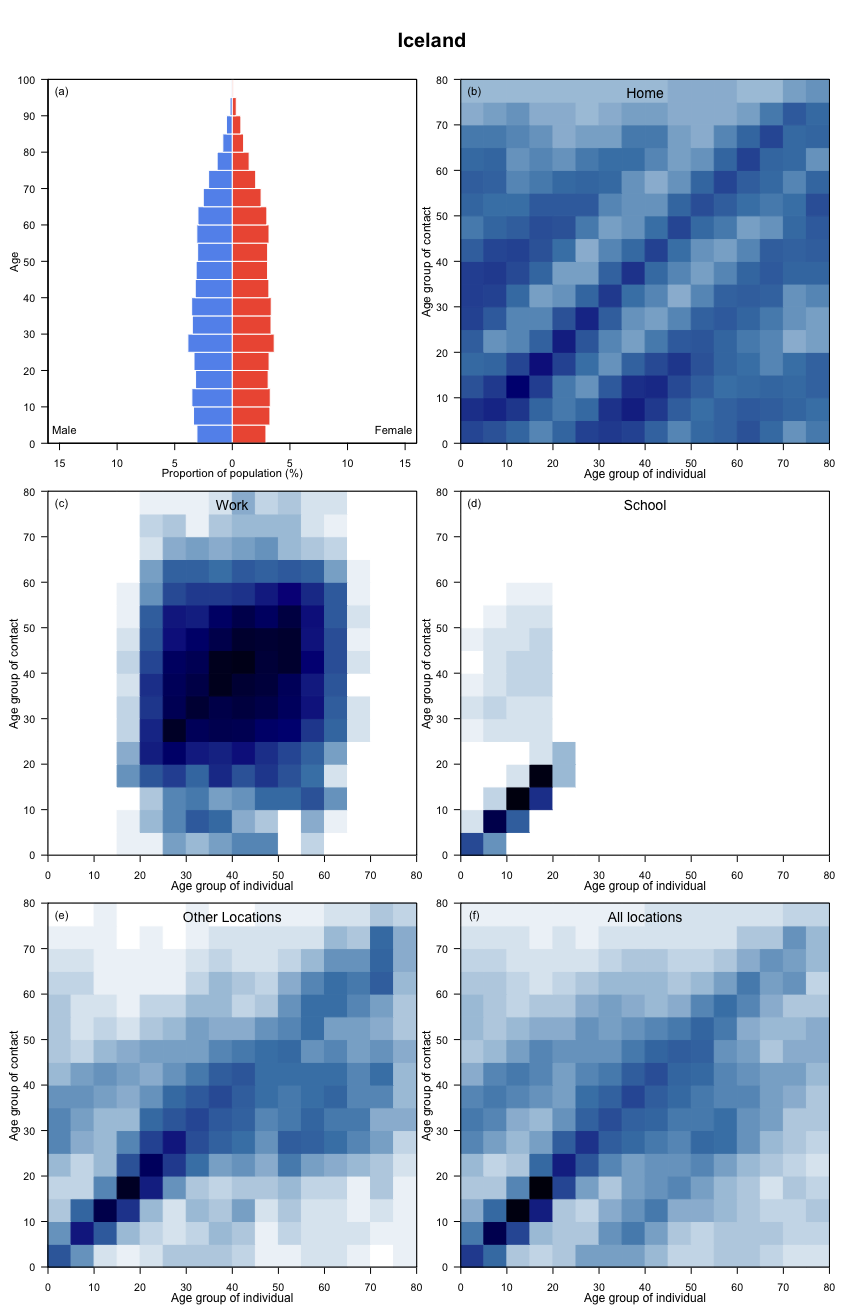

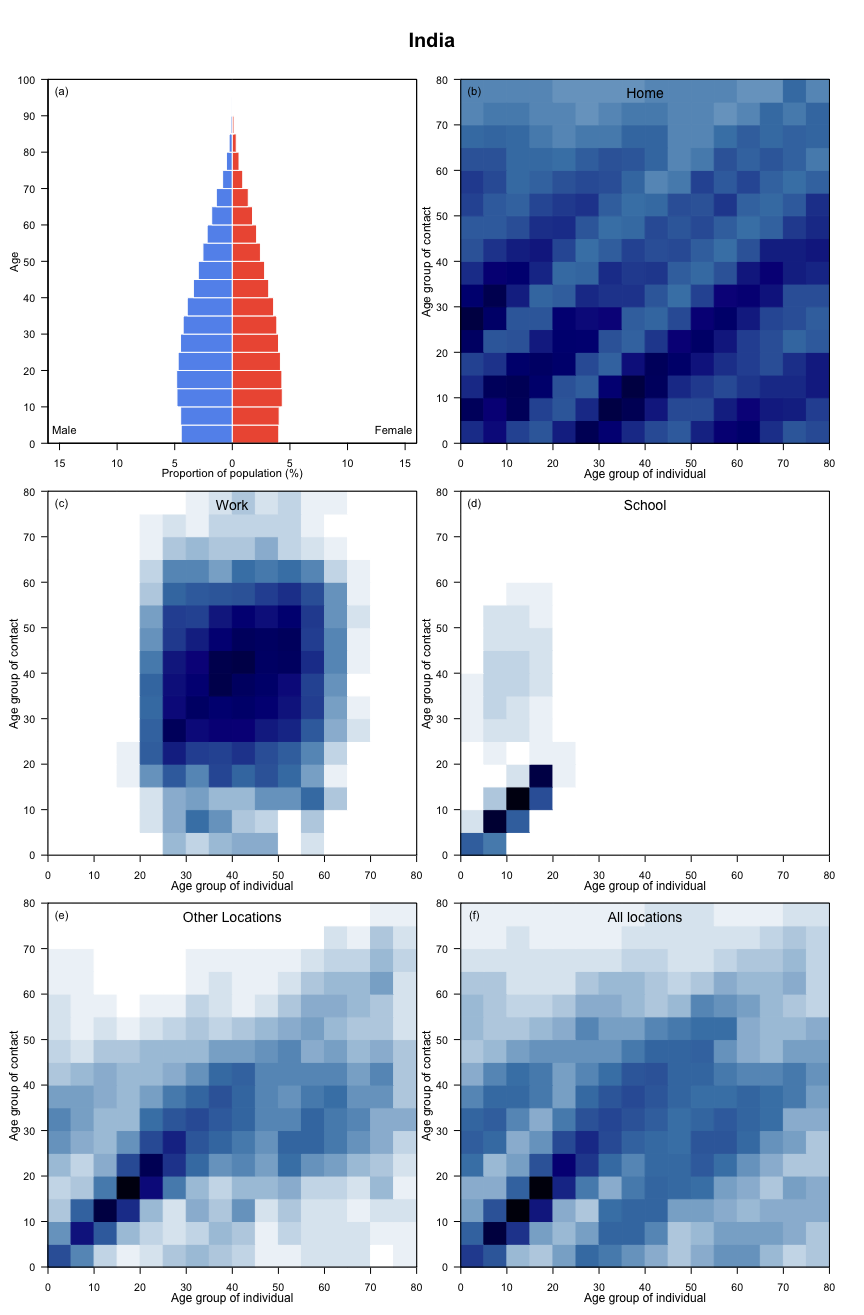

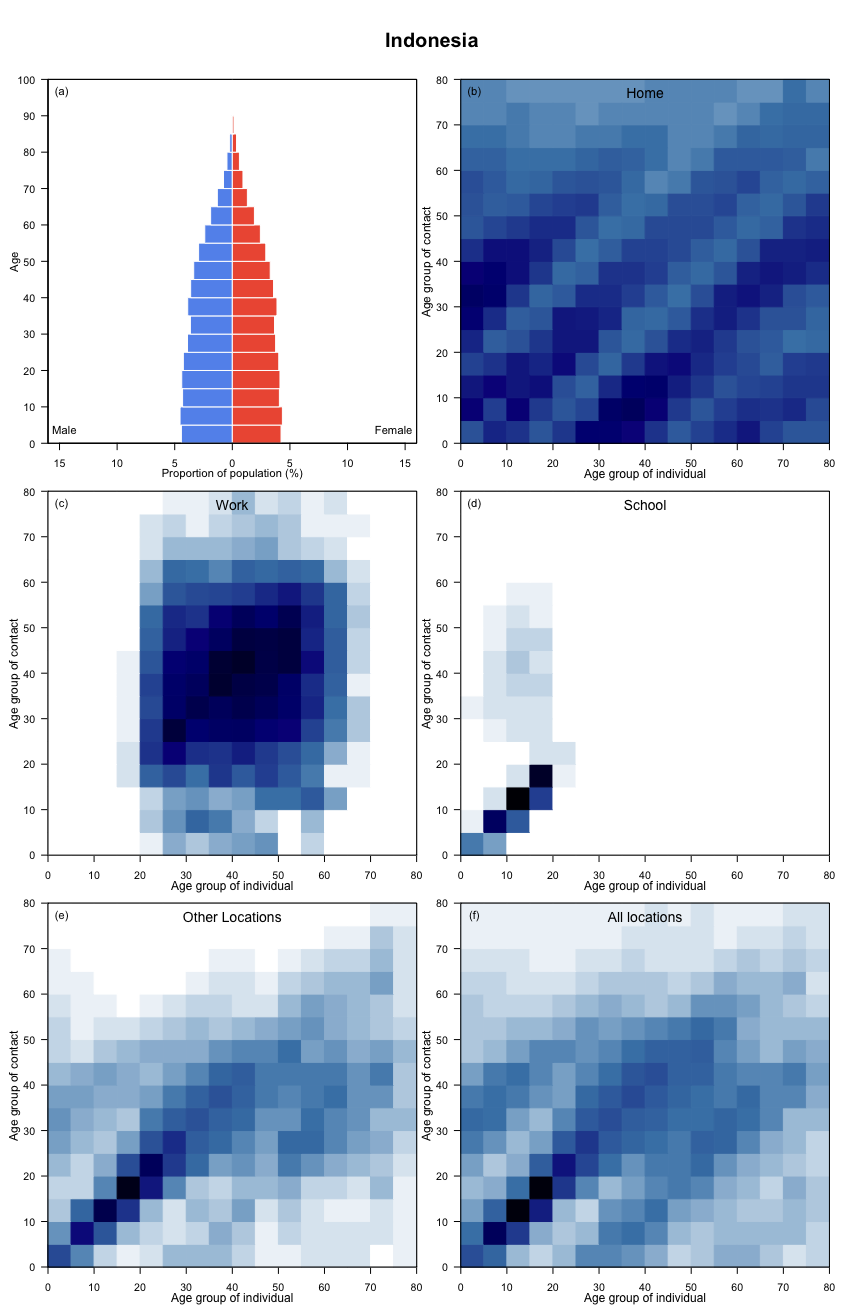

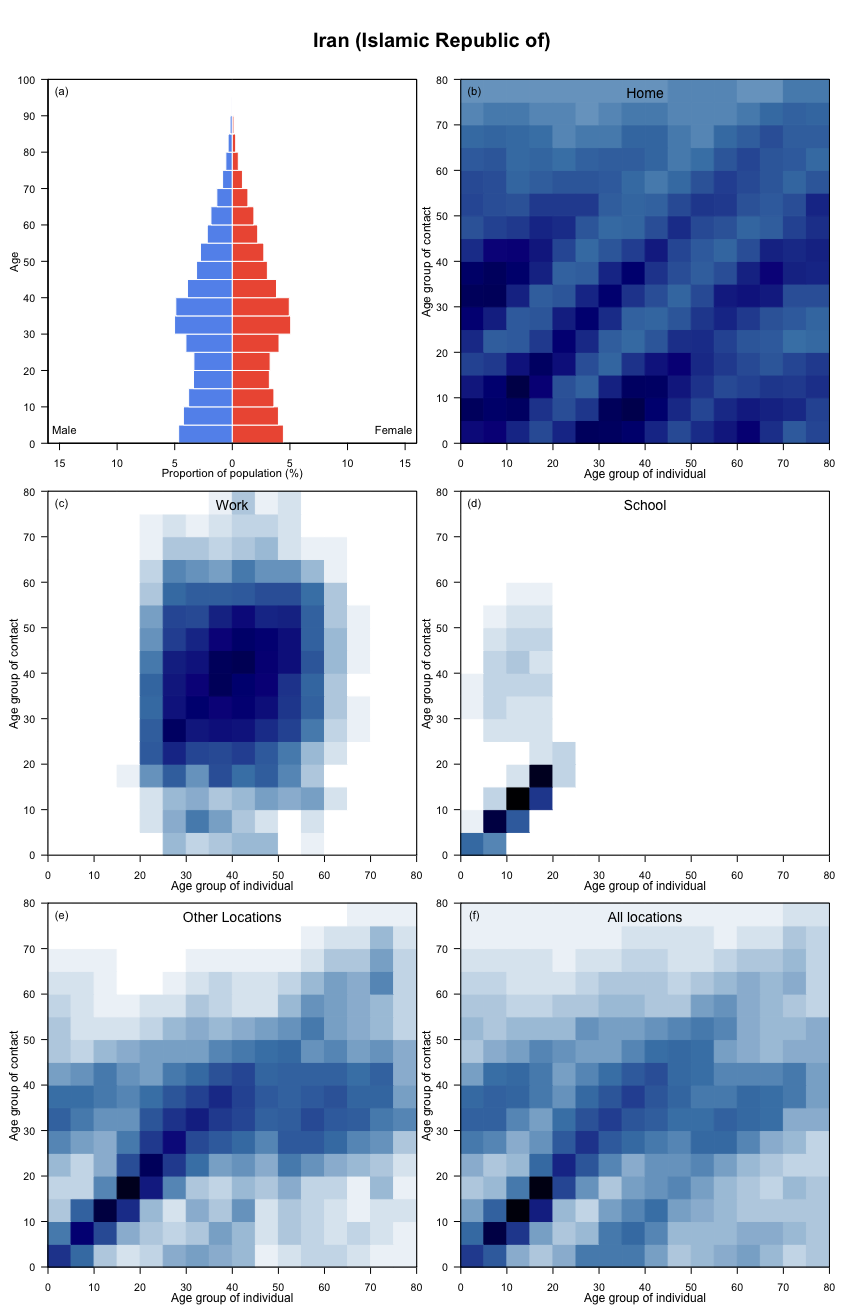

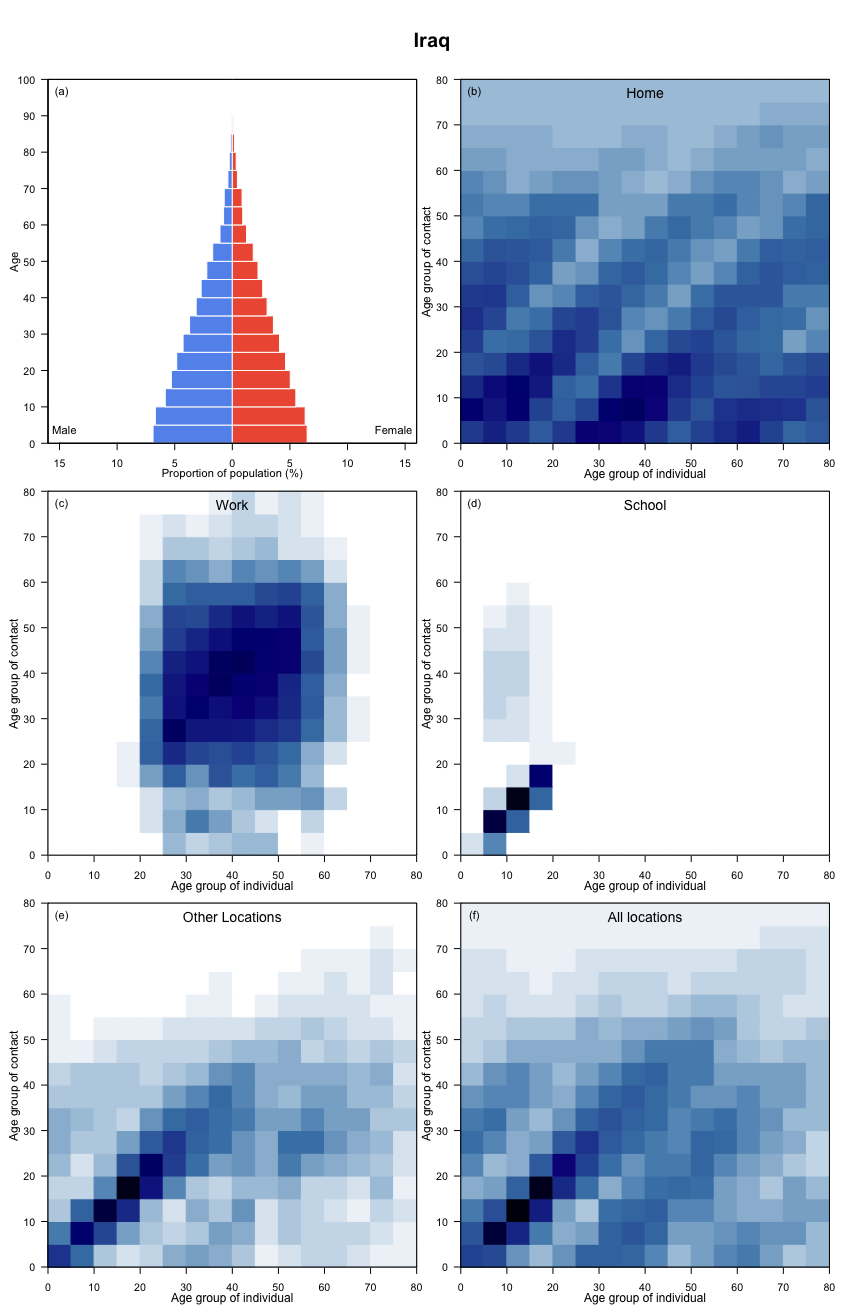

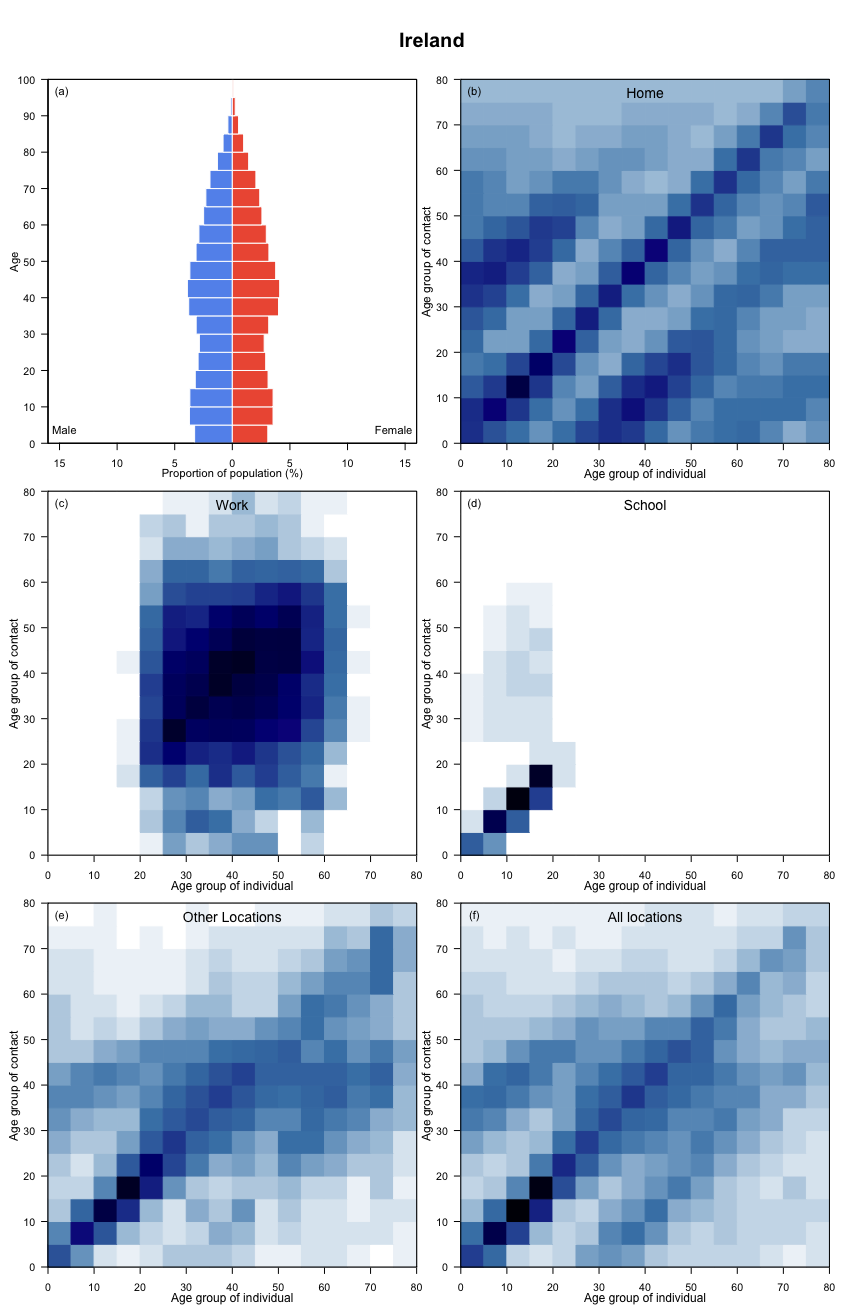

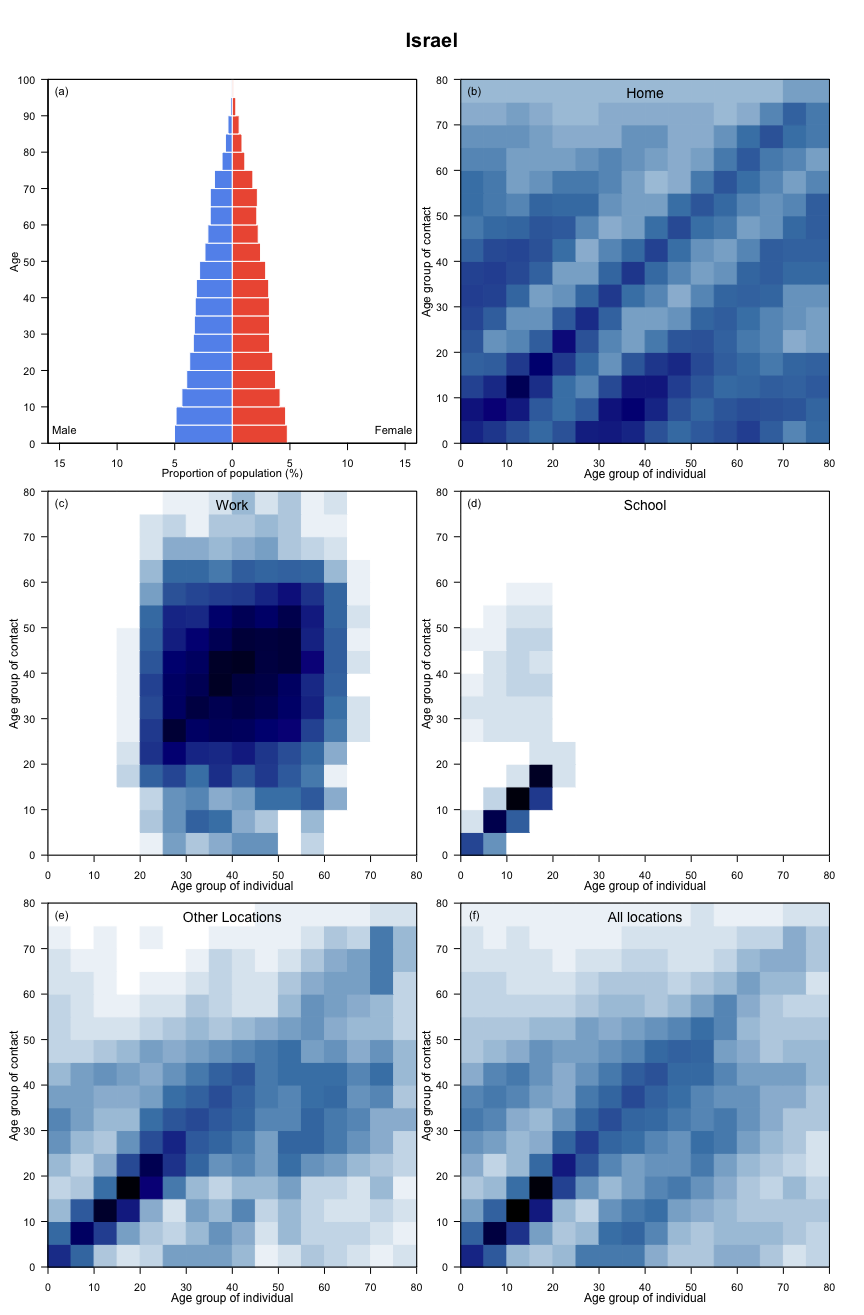

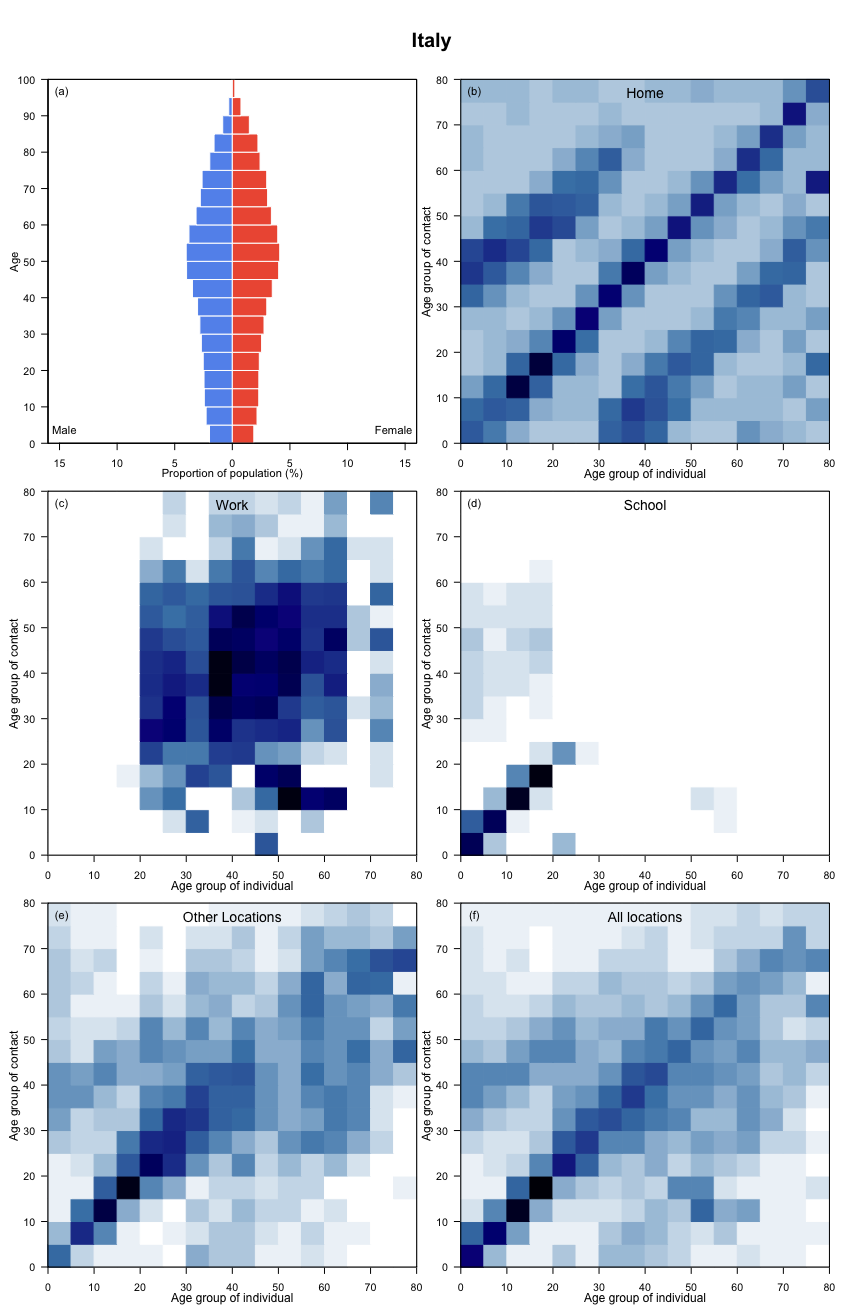

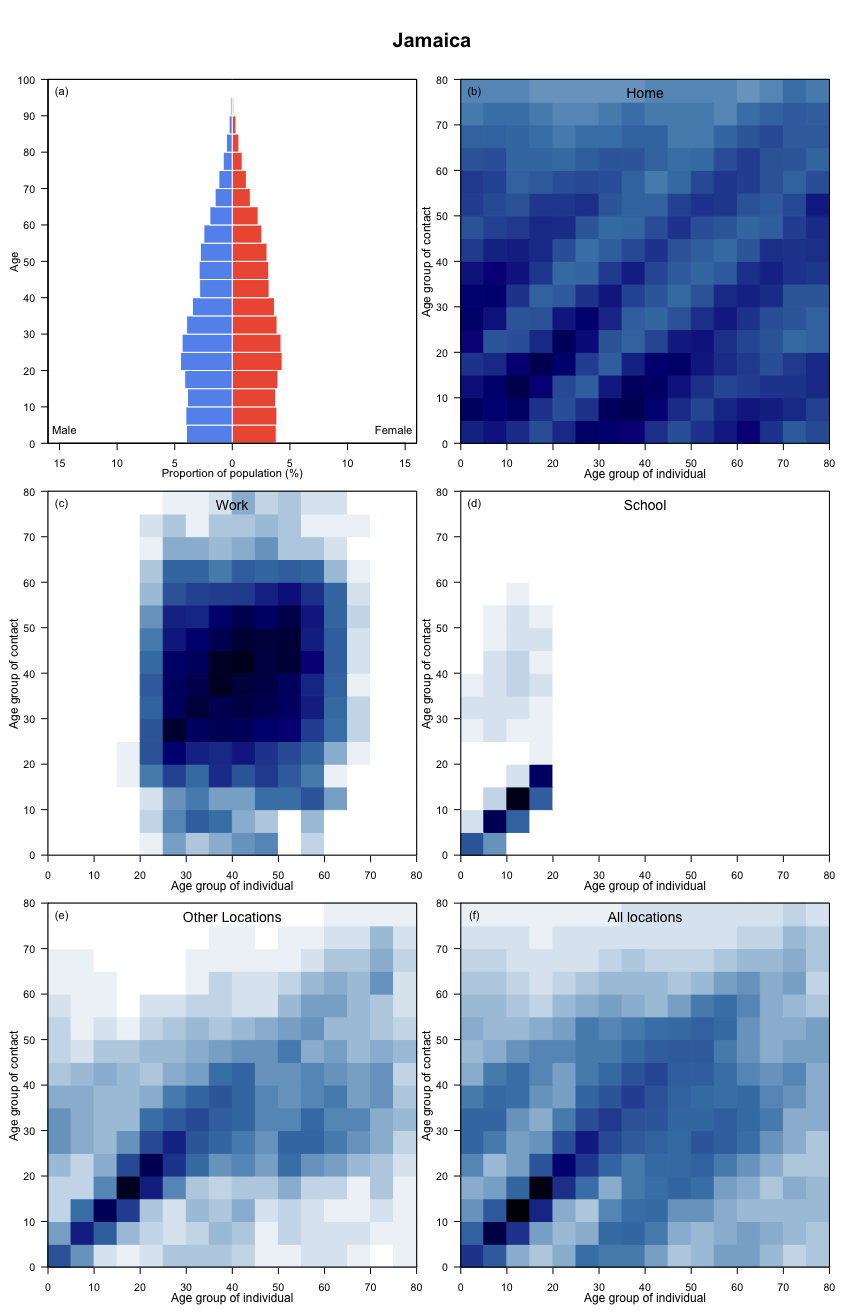

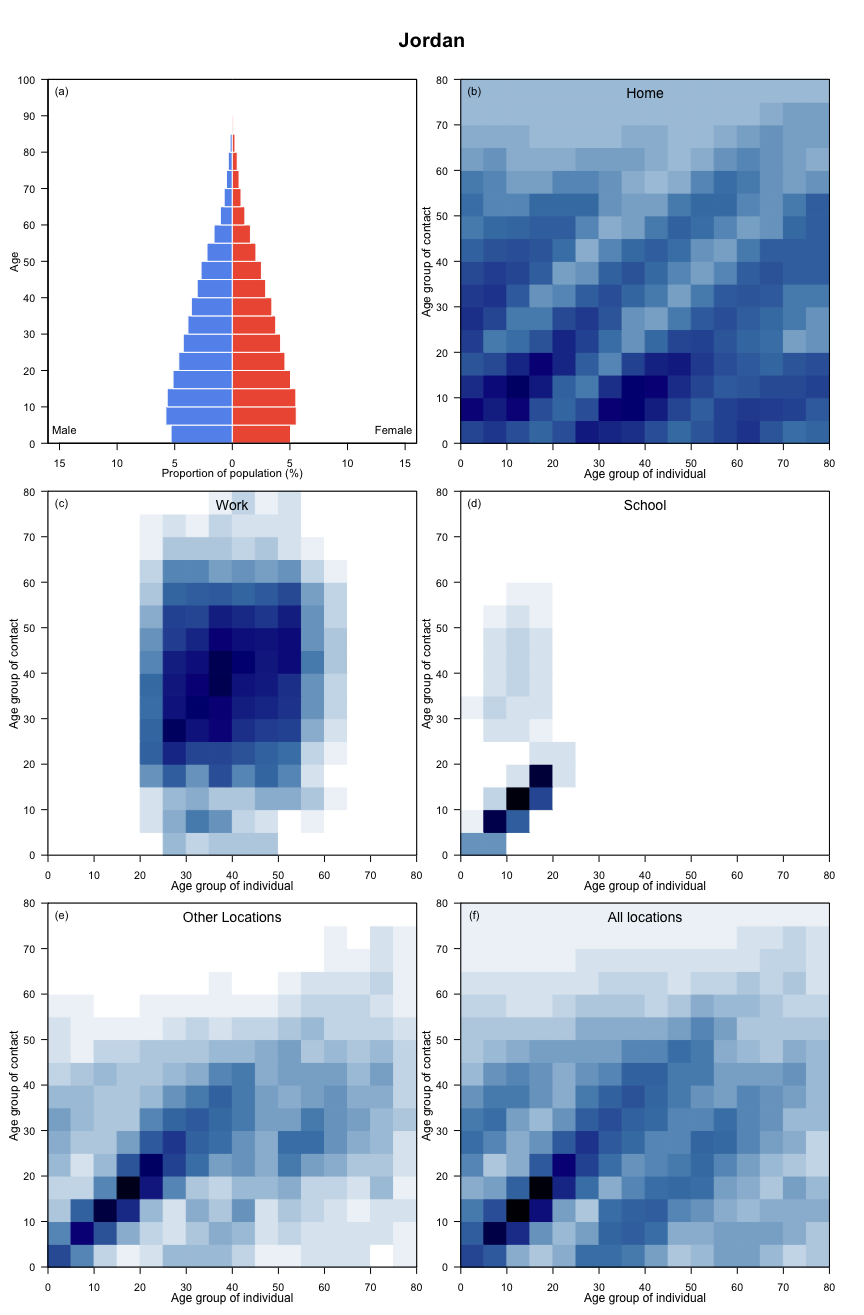


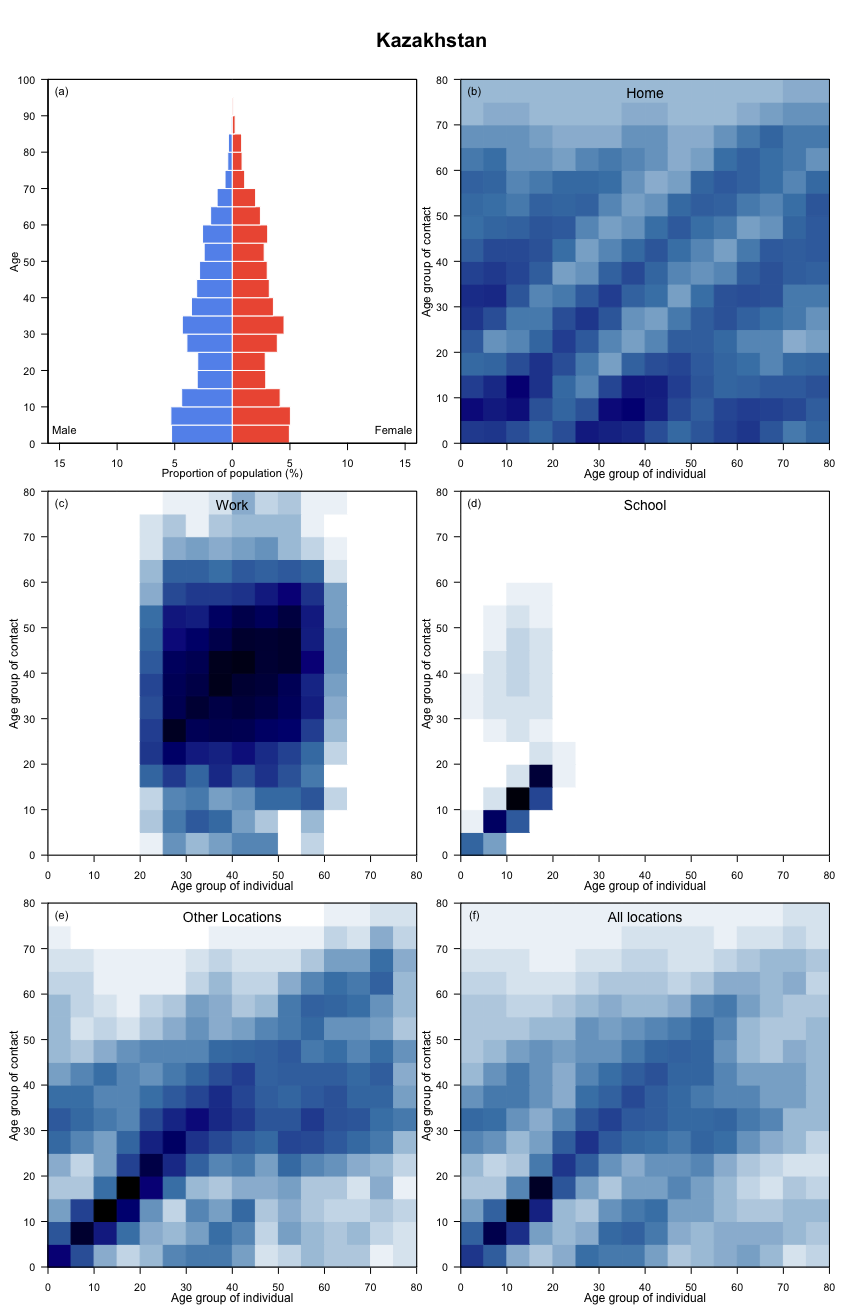

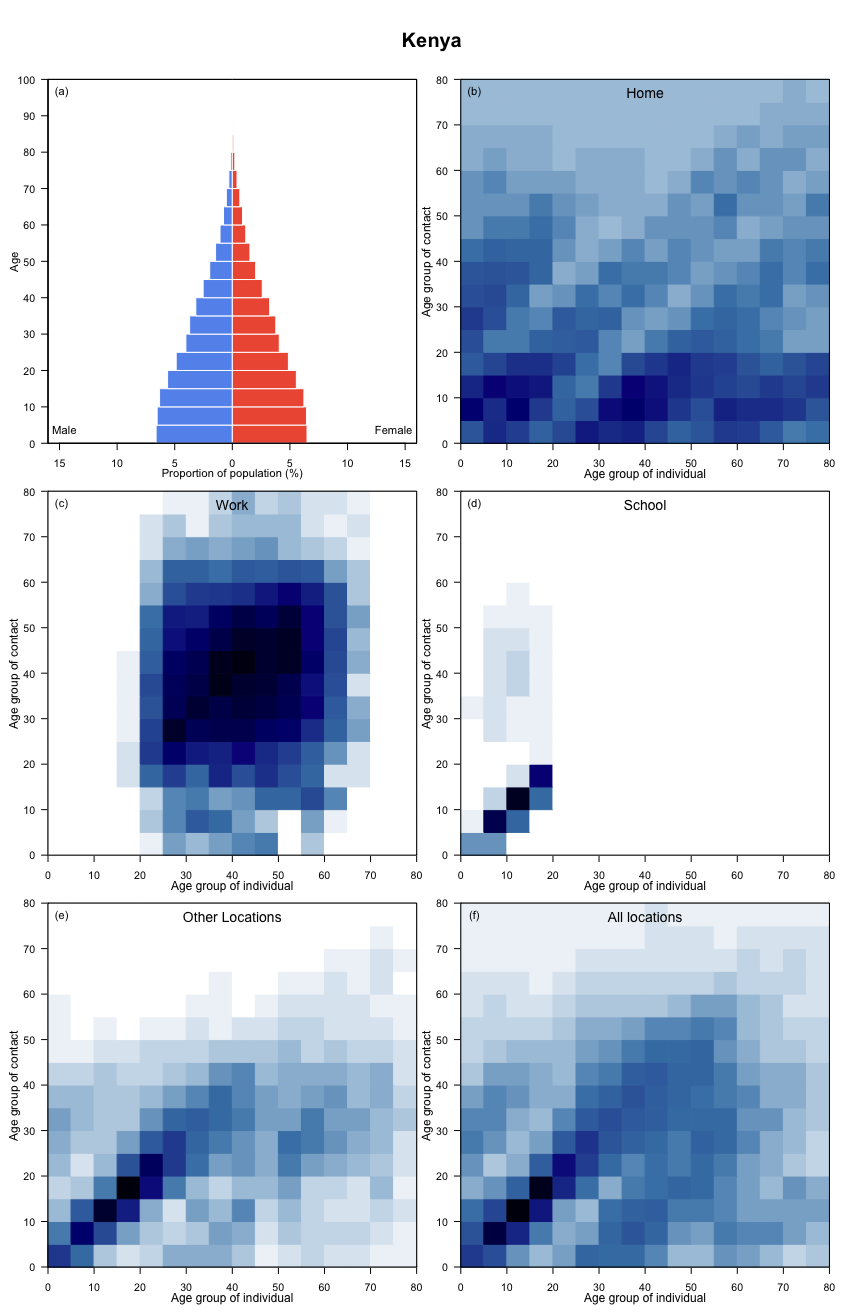

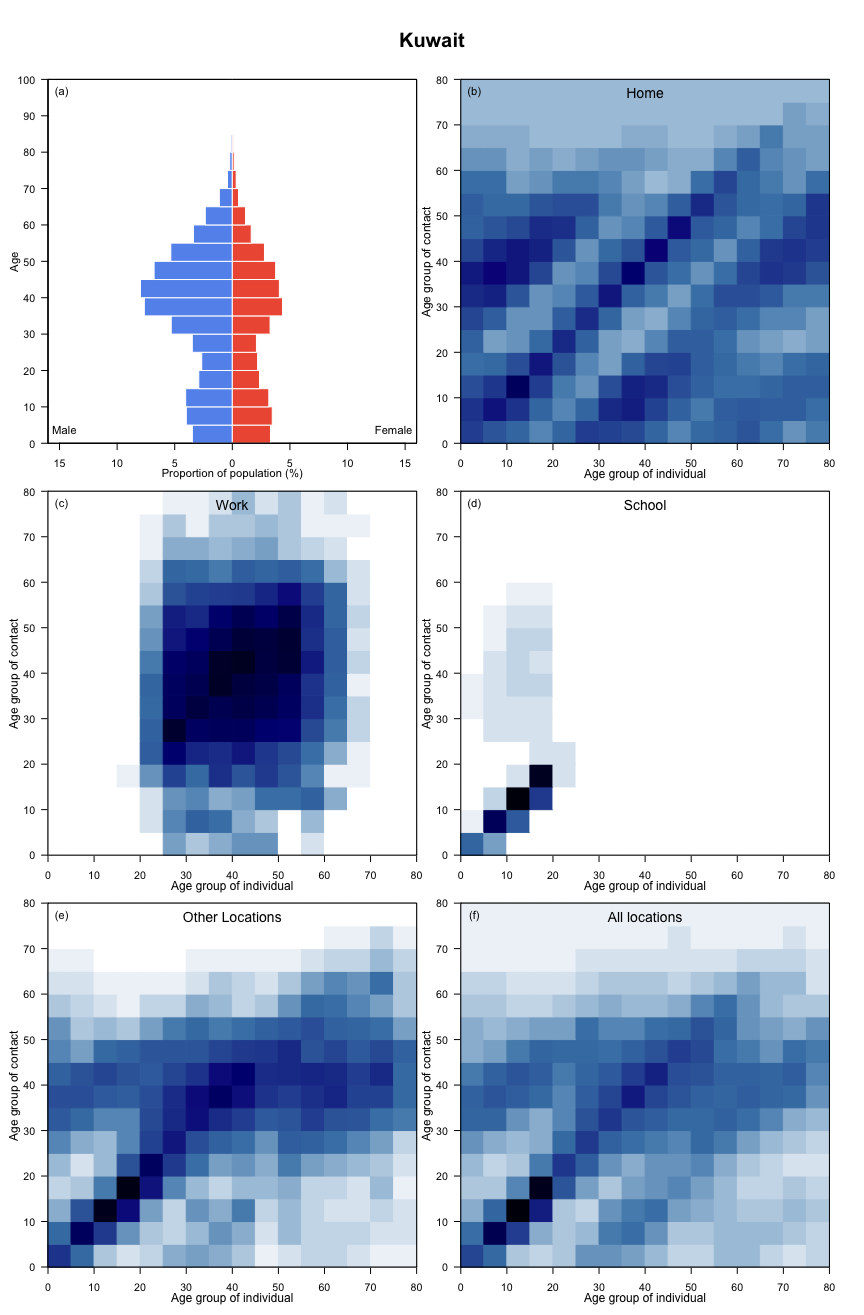

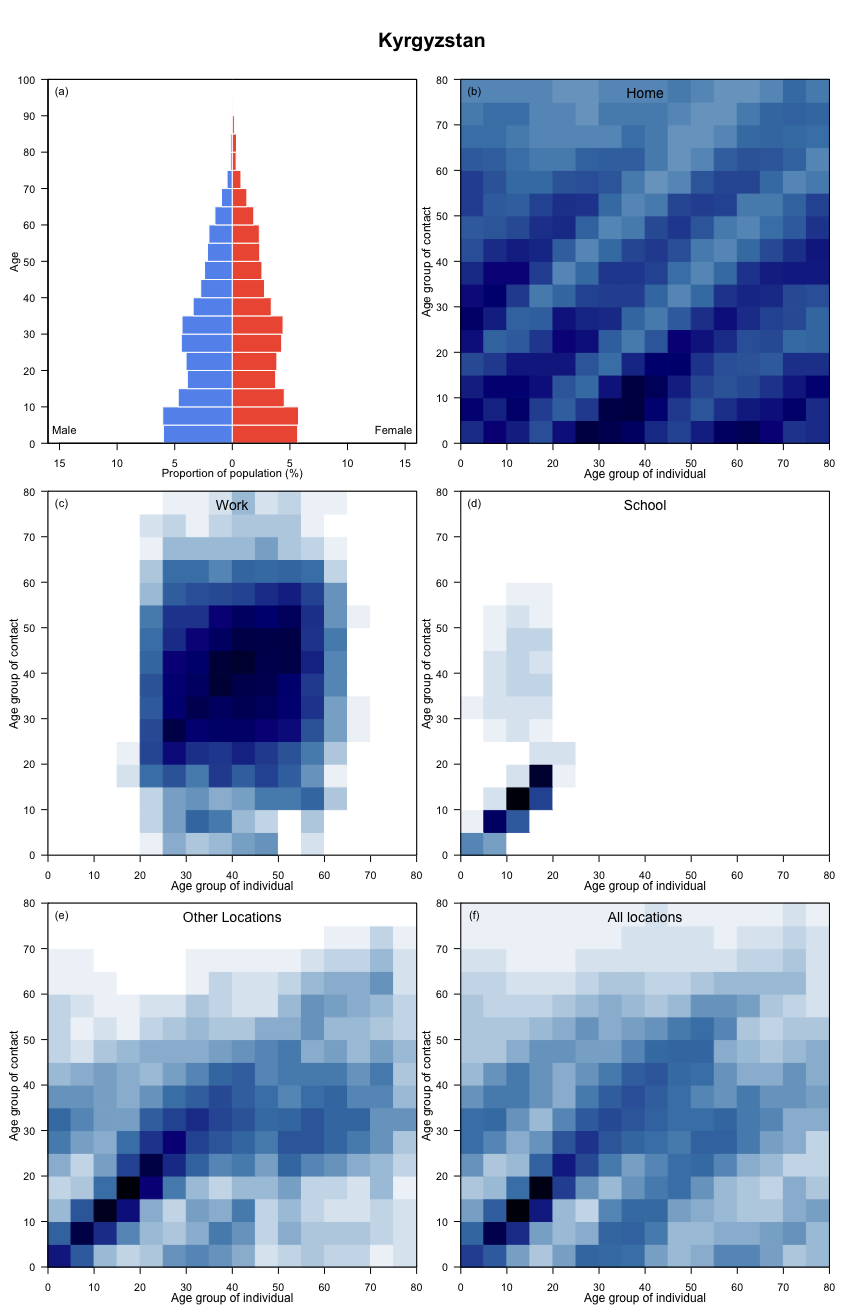

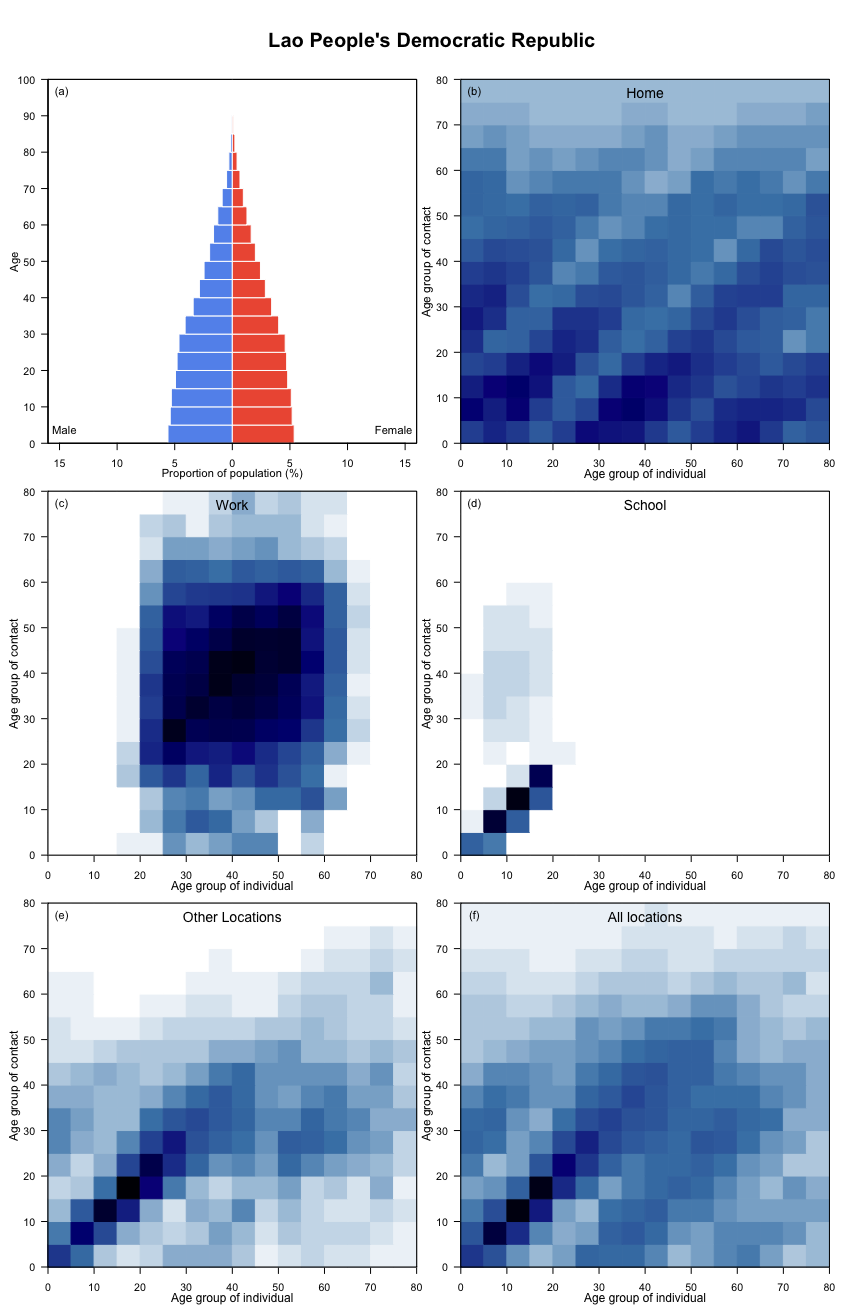

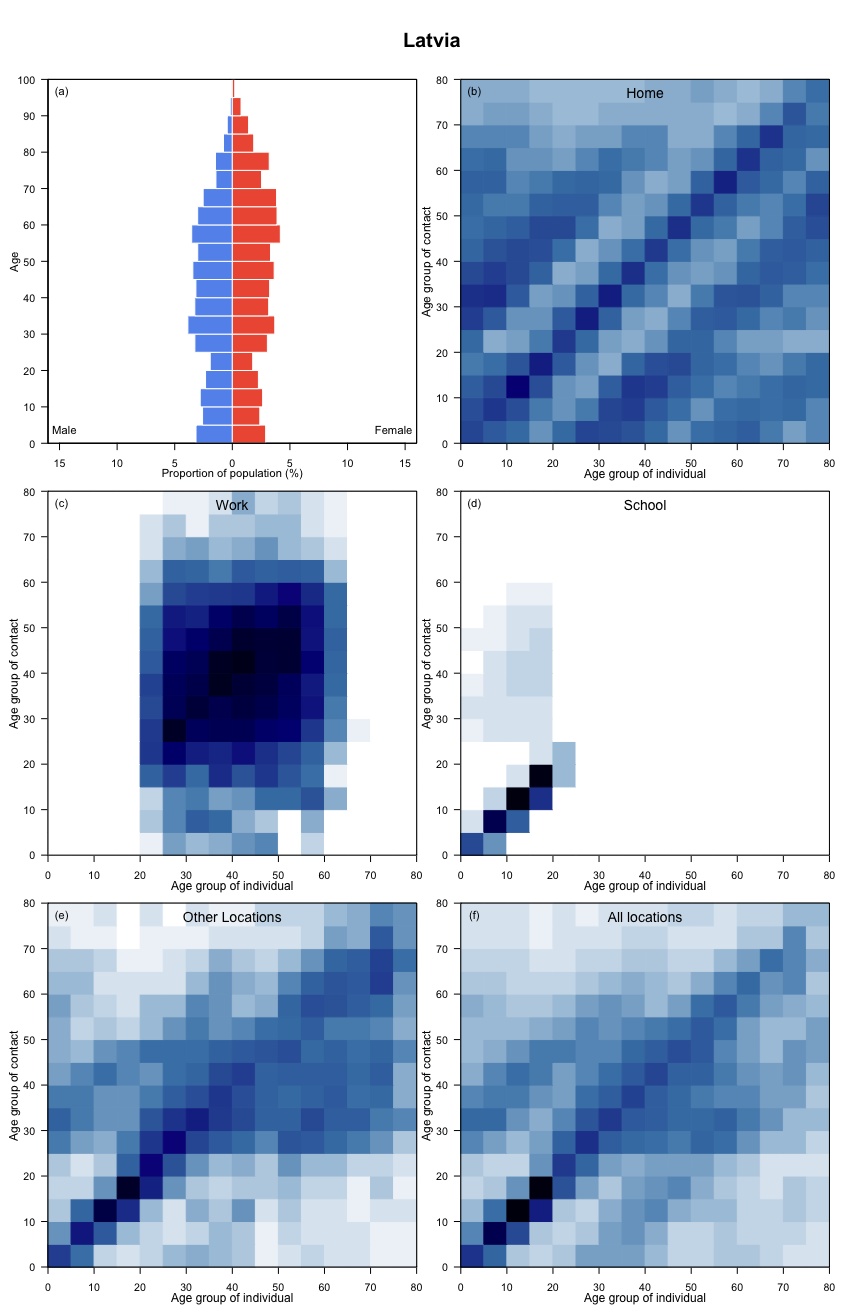

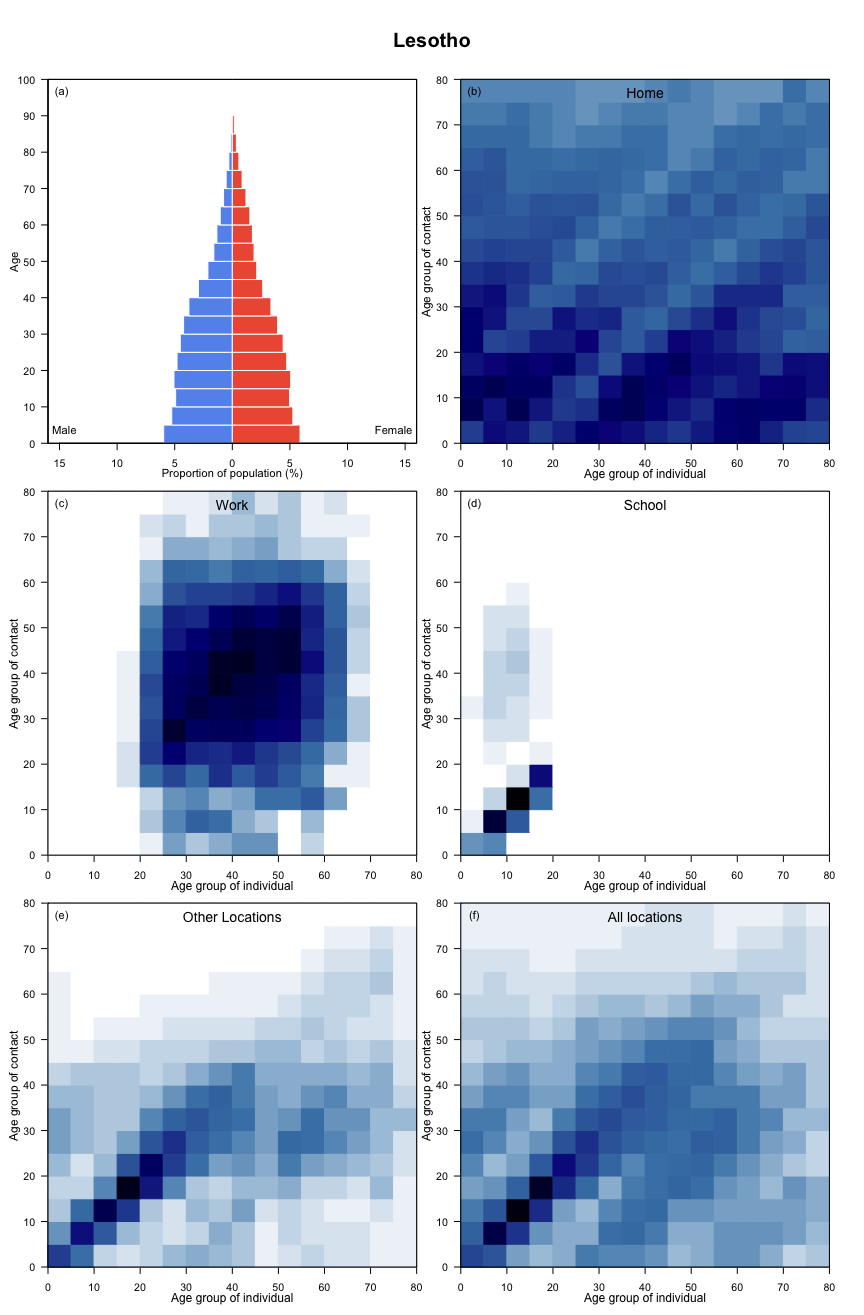

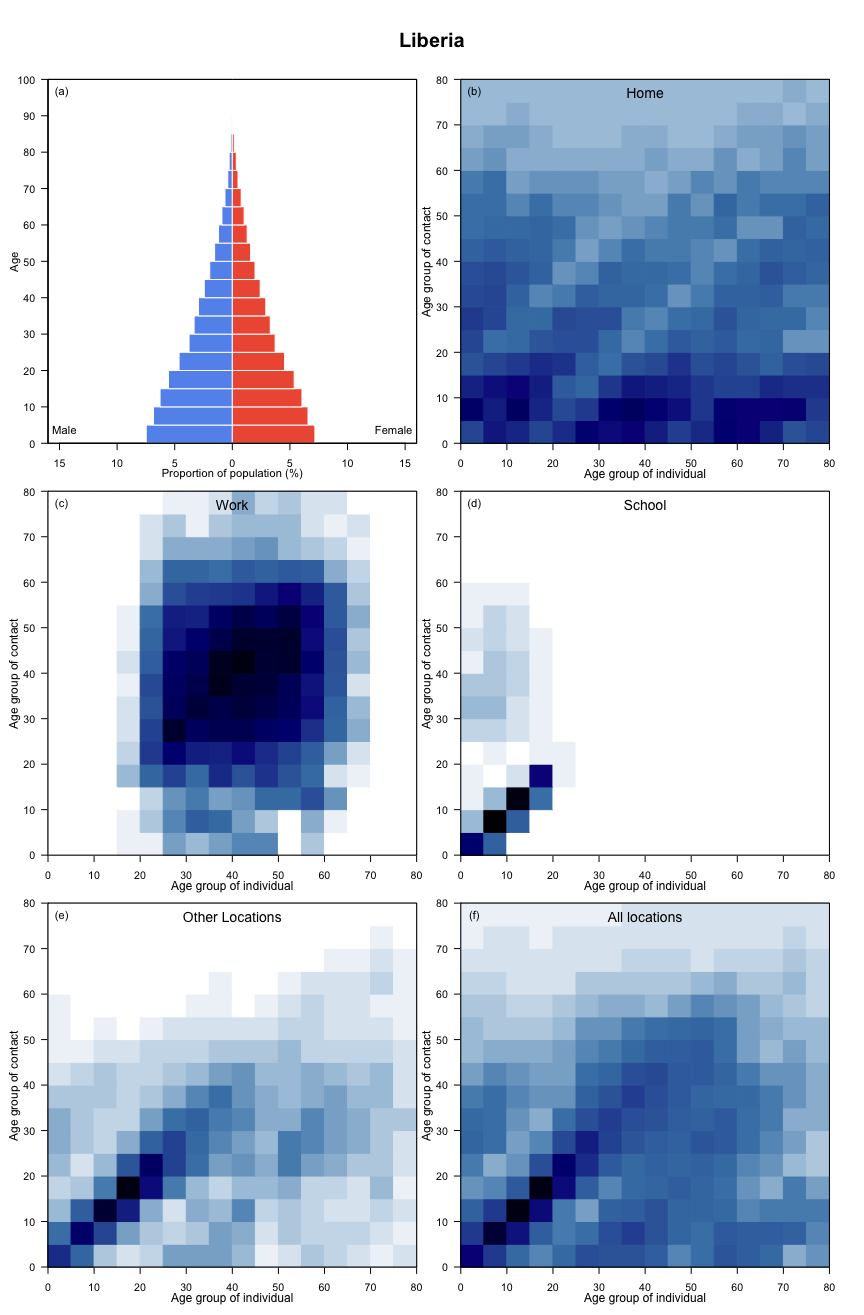

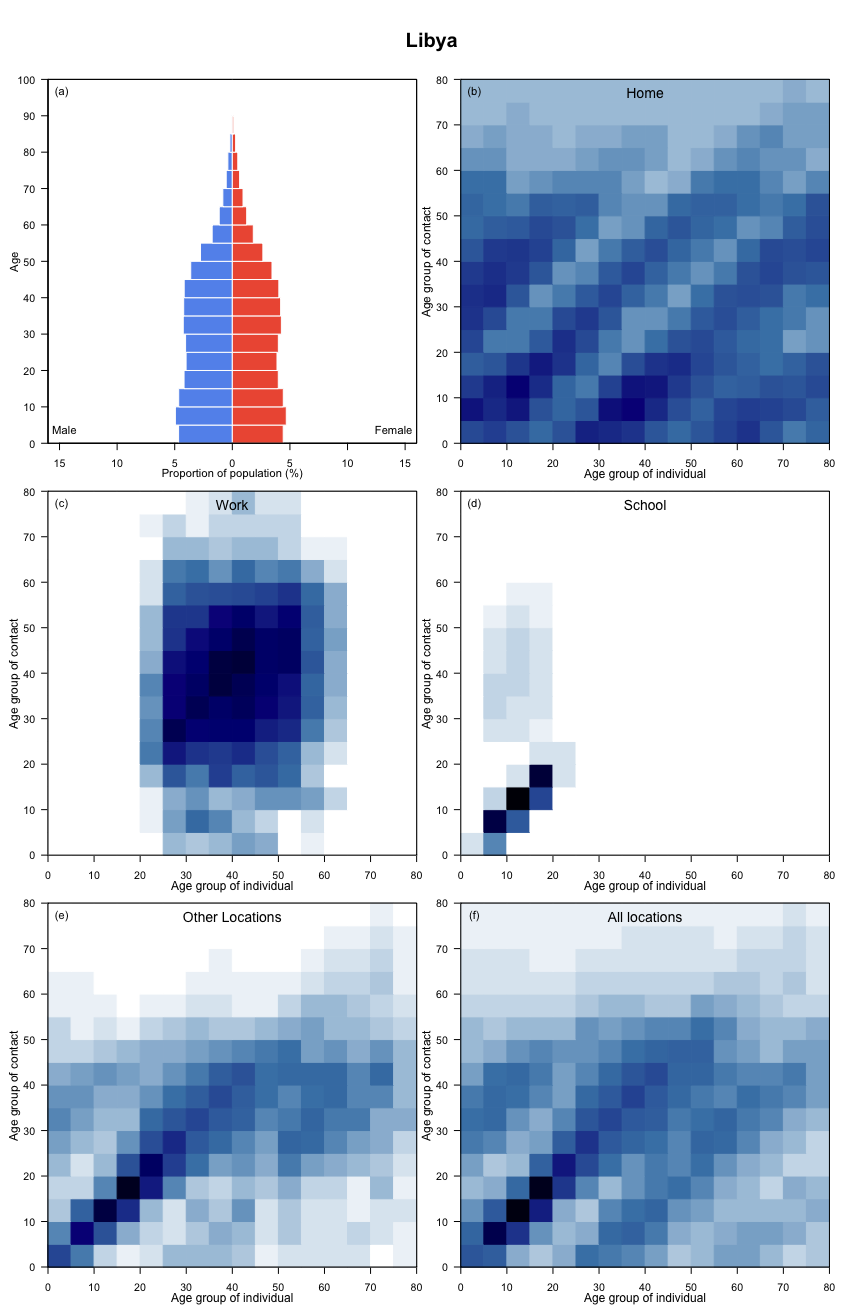

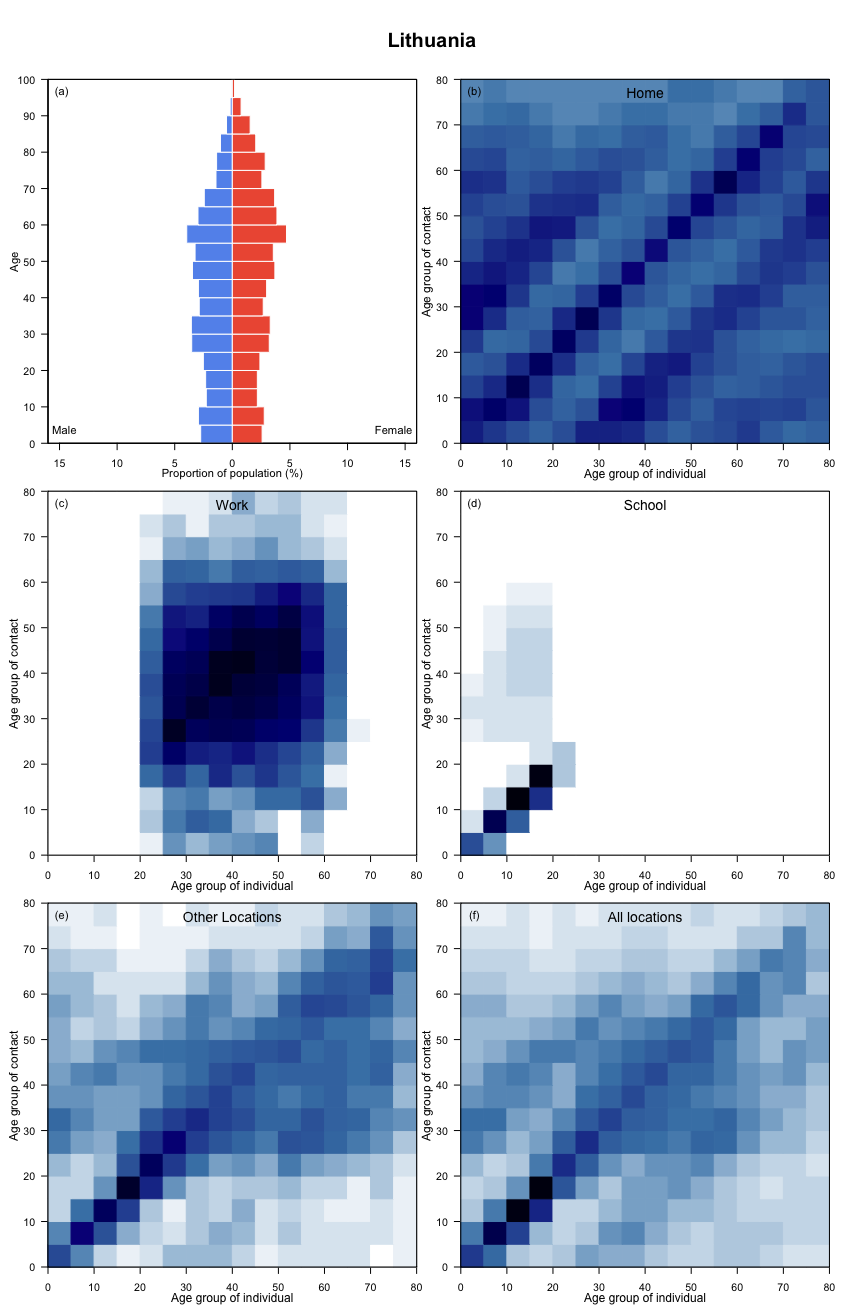

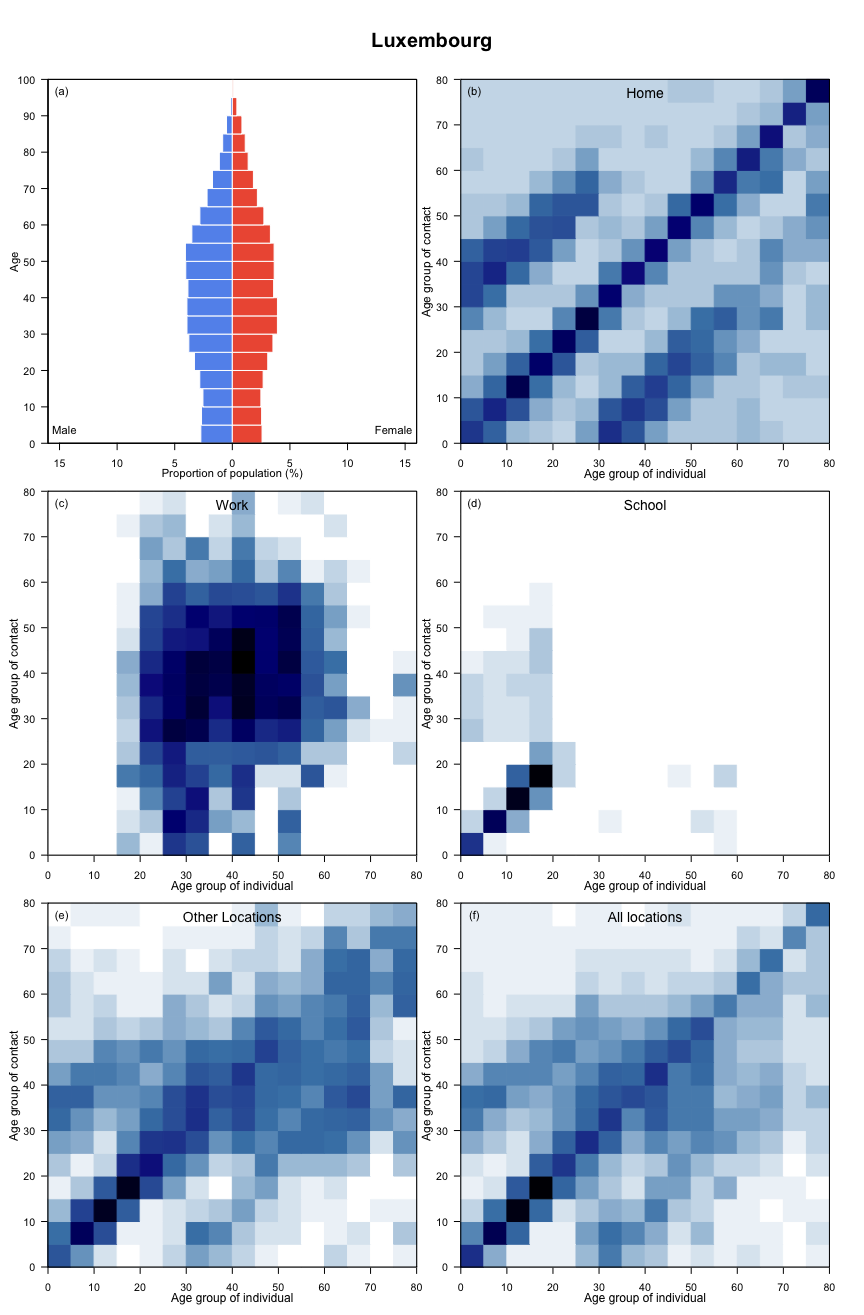

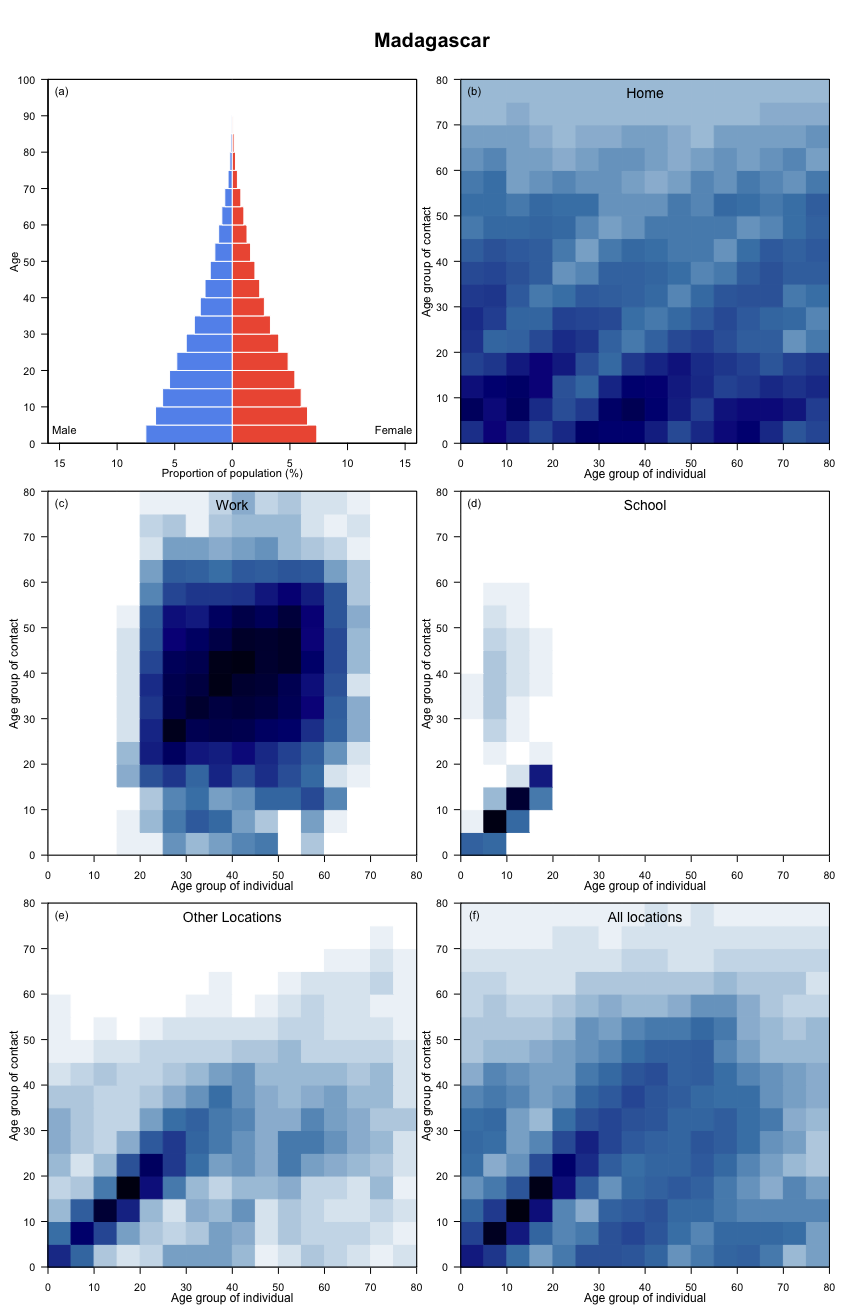

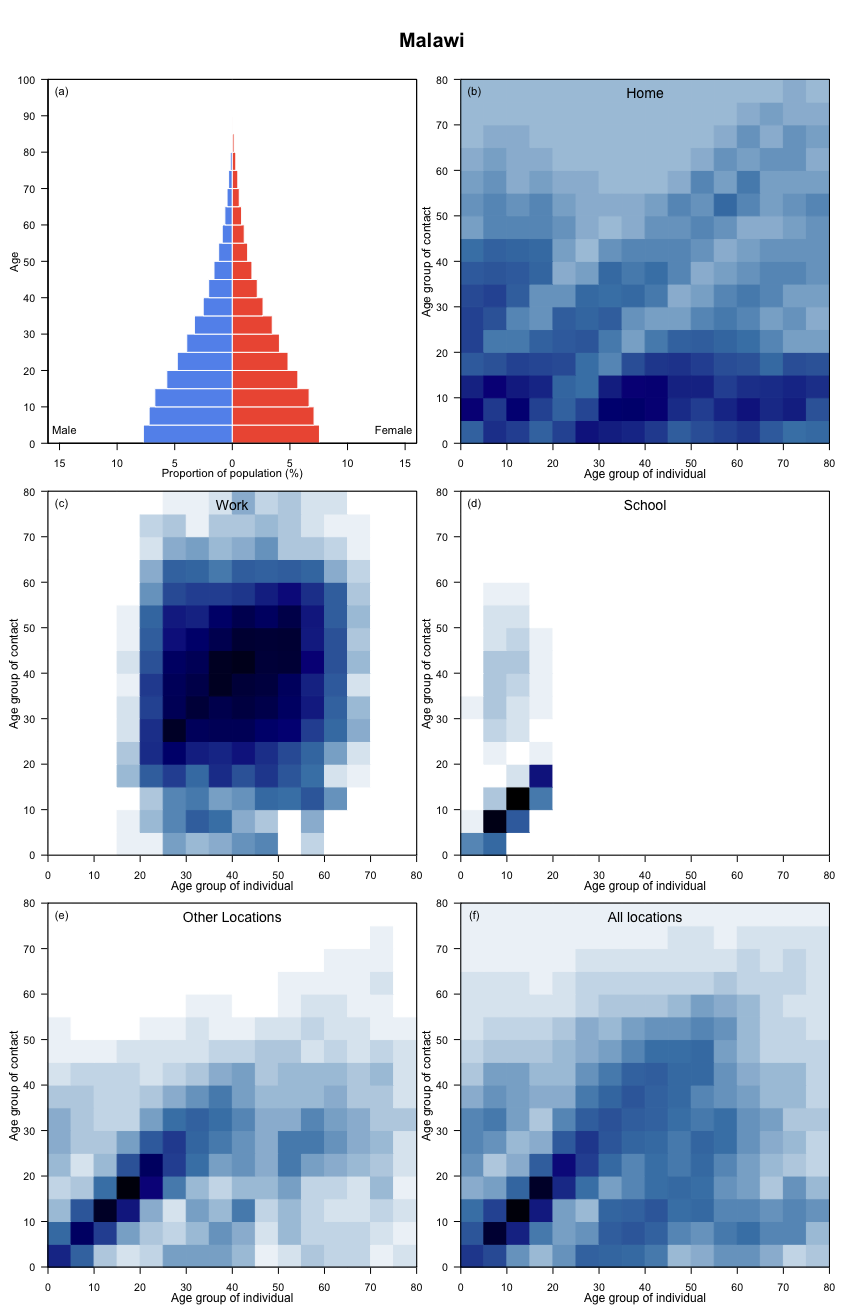

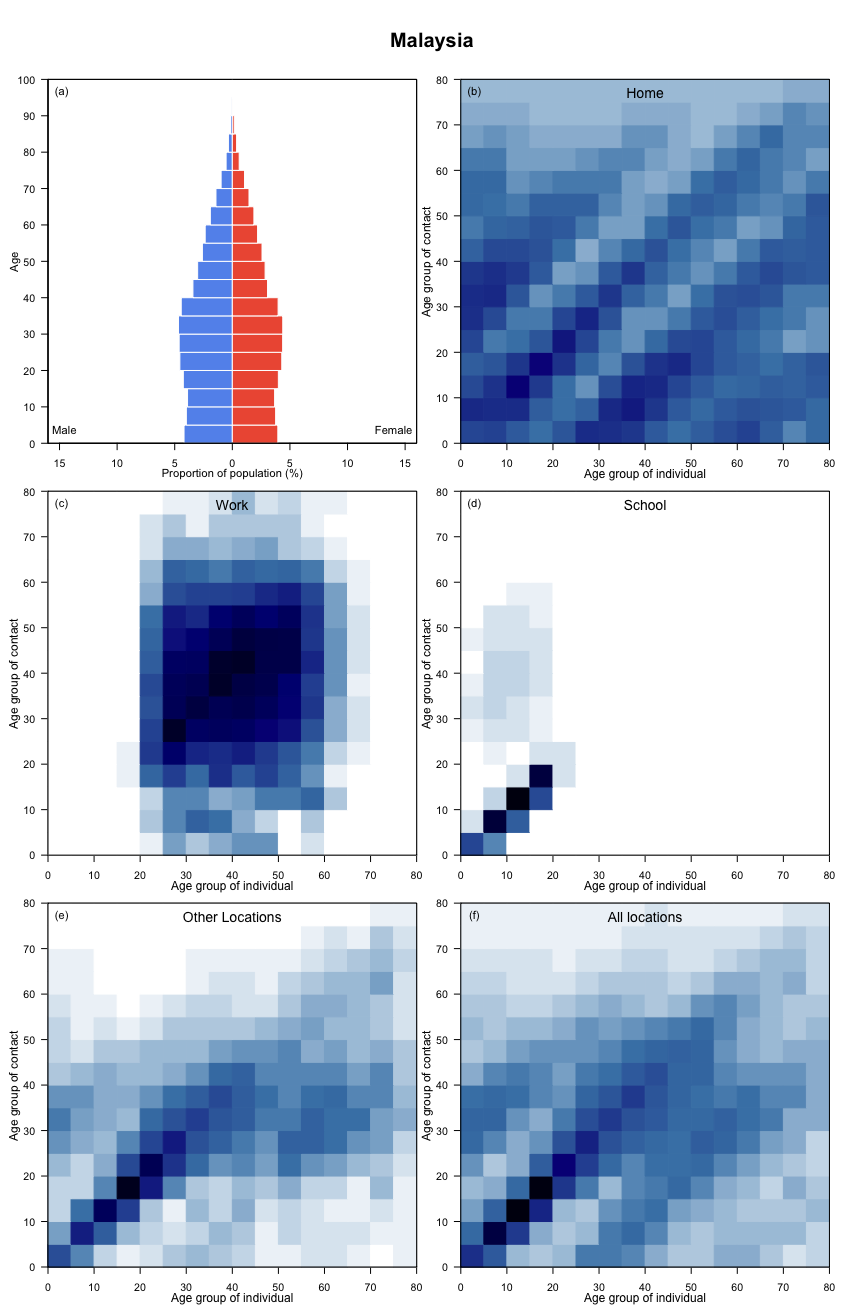

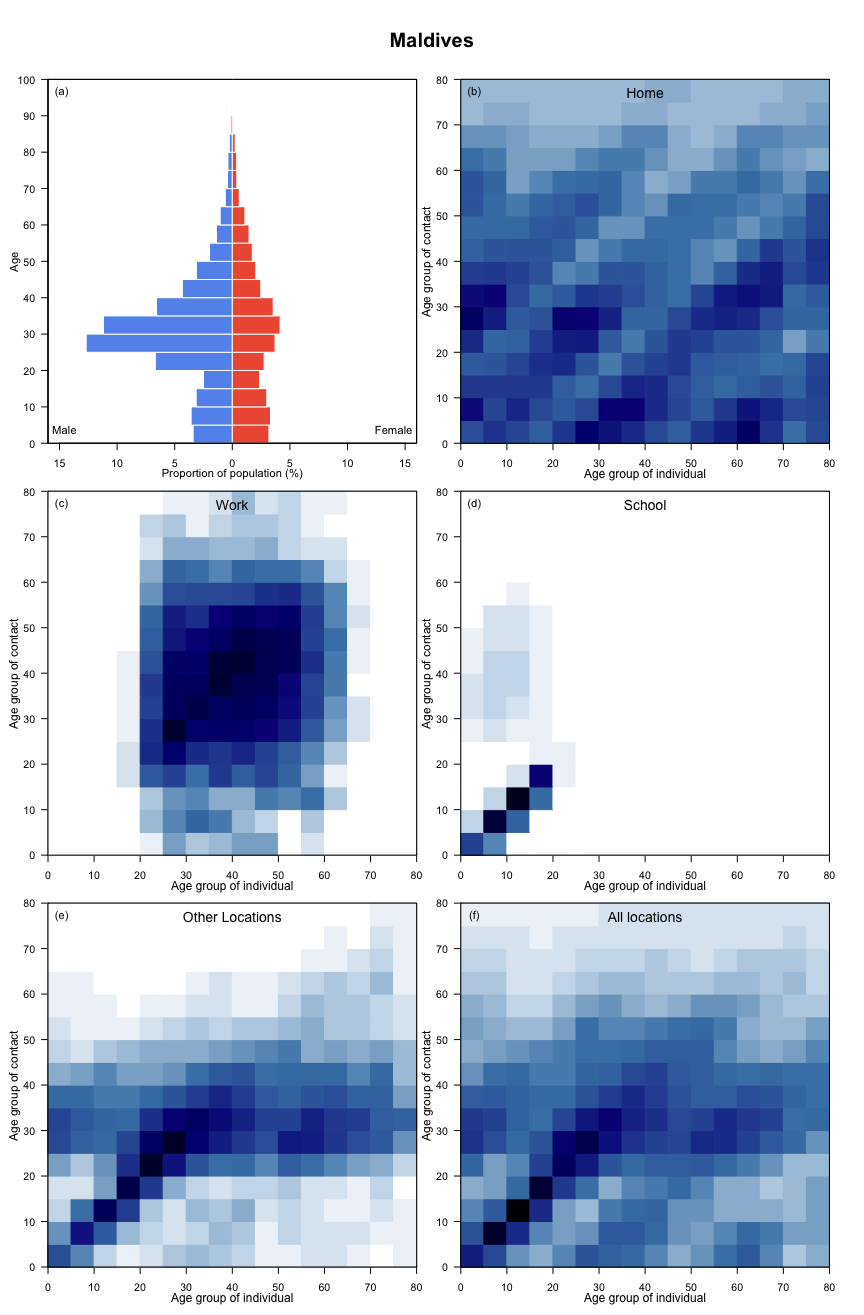

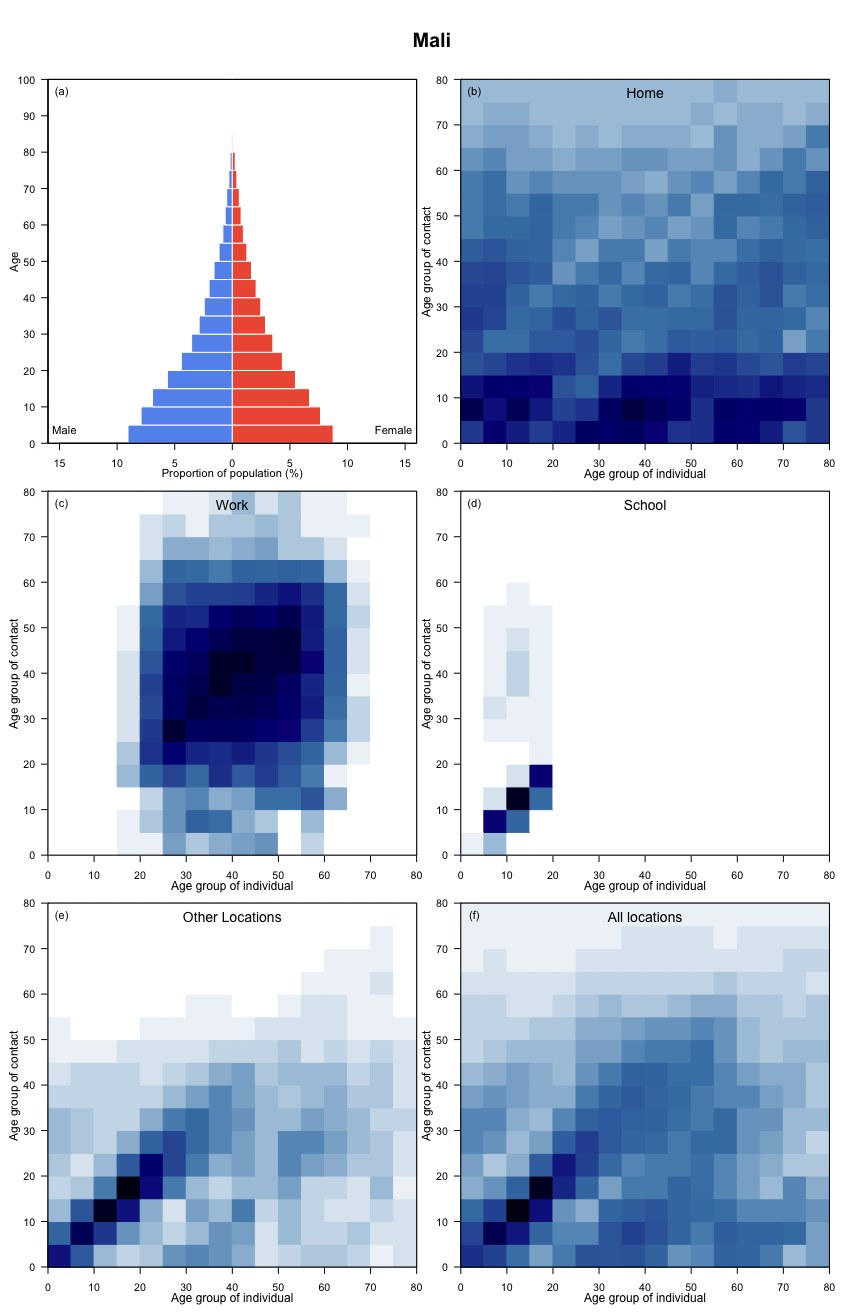

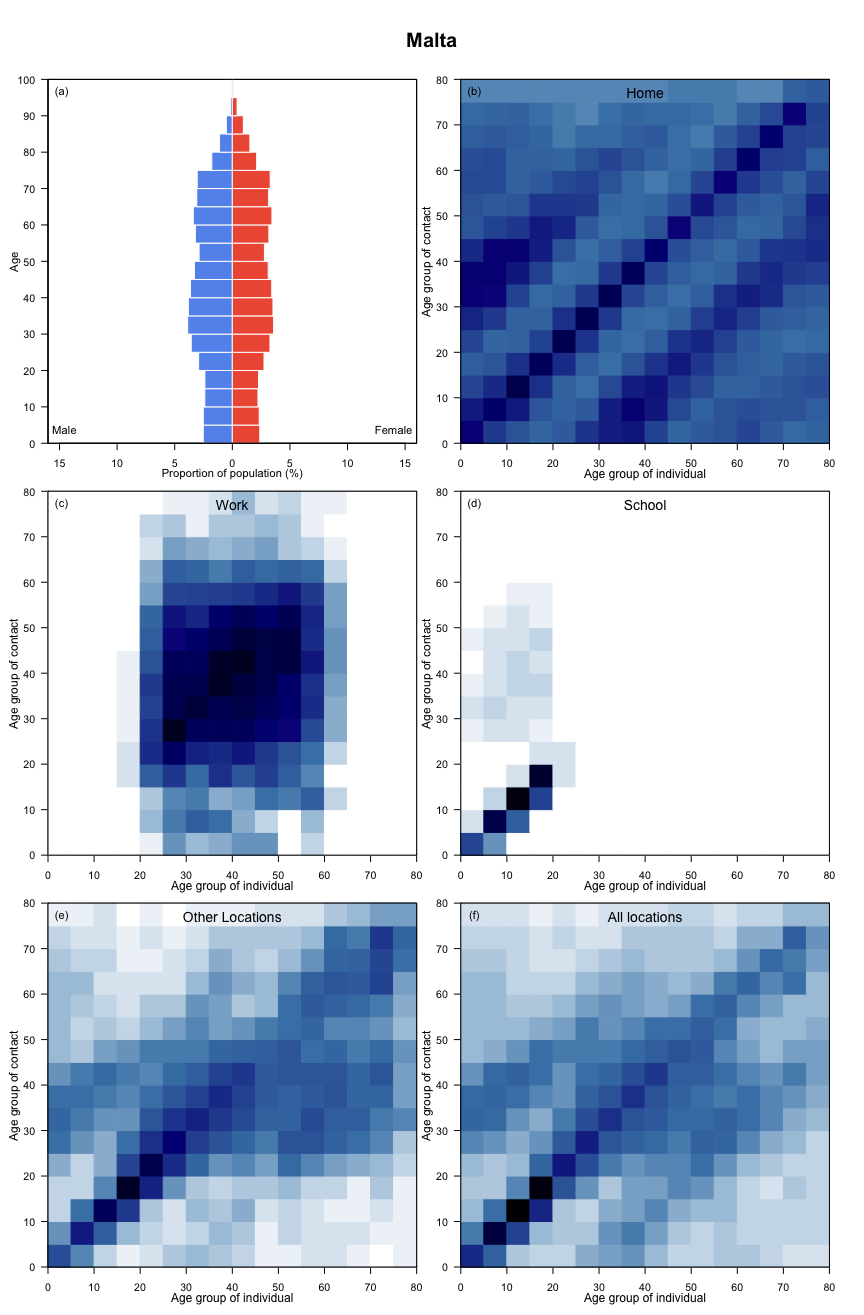

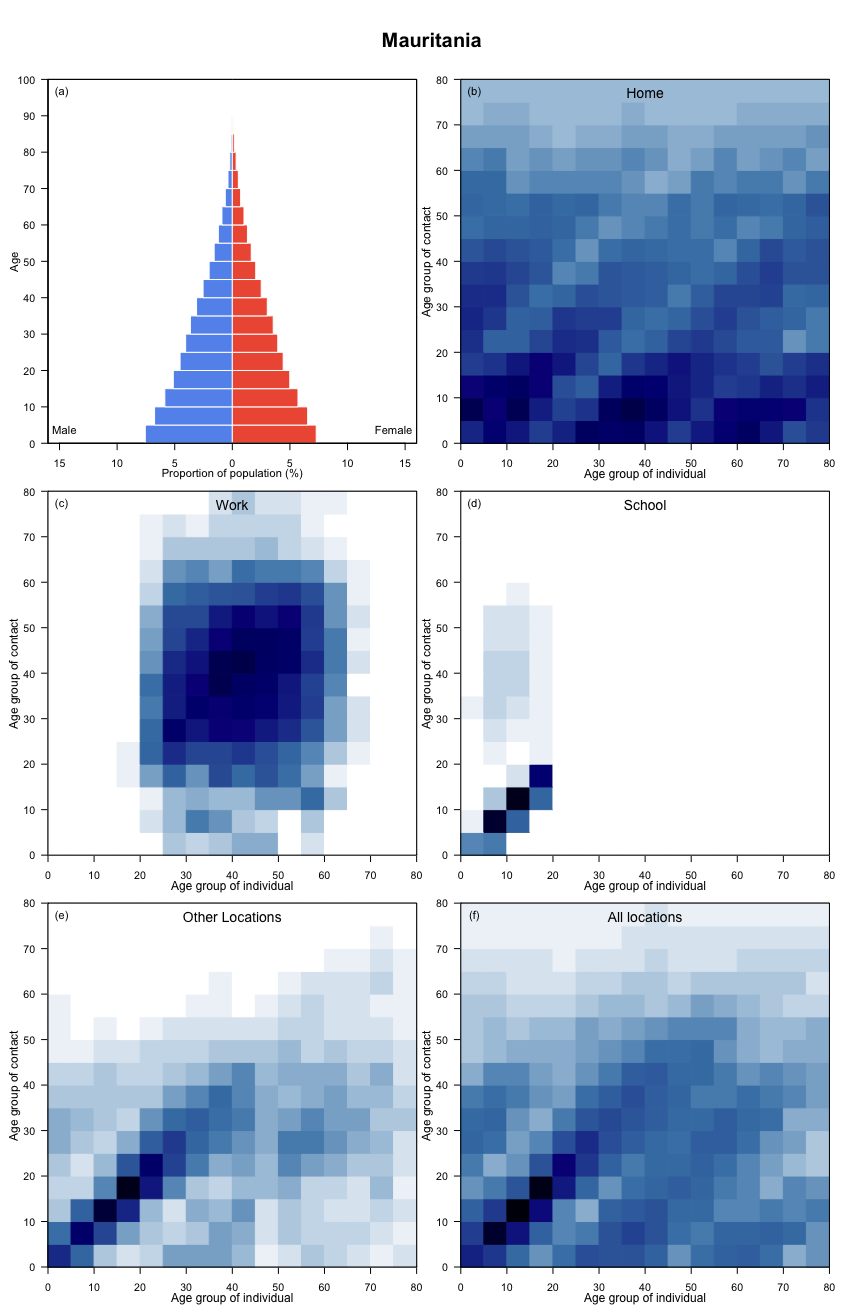


1. Correspondence to [mark.jit@lshtm.ac.uk](mailto:mark.jit@lshtm.ac.uk). [↑](#footnote-ref-1)
