# Supplementary material for: Projecting contact matrices in 177 geographical regions: An update and comparison with empirical data for the COVID-19 era
Source: PLoS Comput Biol. 2021 Jul 26;17(7):e1009098. doi: 10.1371/journal.pcbi.1009098 (PMC8354454; doi:10.1371/journal.pcbi.1009098)
Supplement: S4 Text — (DOCX) [file pcbi.1009098.s004.docx]

Projecting contact matrices in 177 geographical regions: an update and comparison with empirical data for the COVID-19 era

Supplementary Material: Results III (comparisons)

Kiesha Prem, Kevin van Zandvoort, Petra Klepac, Rosalind M Eggo, Nicholas G Davies,

Centre for the Mathematical Modelling of Infectious Diseases COVID-19 Working Group,

Alex R Cook, Mark Jit^[[1]](#footnote-1)^

Contents

[List of Tables 1](#_Toc144639834)

[List of Figures 1](#_Toc144639835)

[B.4. Comparison of the empirical and 2017 synthetic age-specific contact matrices in ten geographical regions 2](#_Toc144639836)

[B.5. Age-specific infection attack rate of COVID-19 and comparison of the empirical and updated synthetic age-specific contact matrices in ten geographical regions 5](#_Toc144639837)

[B.6. Degree of symmetry for empirical and synthetic matrices. 9](#_Toc144639838)

# List of Tables

| D | Possible reason for discrepancies in age-specific infection attack rates........................................257 |
| --- | --- |
| E | Degree of symmetry for empirical and synthetic matrices..............................................................259 |

# List of Figures

| D | Age-specific infection attack rate of COVID-19..............................................................................255 |
| --- | --- |
| E | Percentage change in case attack rate of COVID-19....................................................................256 |

## Comparison of the empirical and 2017 synthetic age-specific contact matrices in ten geographical regions

The 2017 synthetic matrices provide validated approximations to age-and-location-specific contact matrices for 152 geographical regions. We compare the normalised empirical matrices with to the normalised 2017 synthetic matrices.


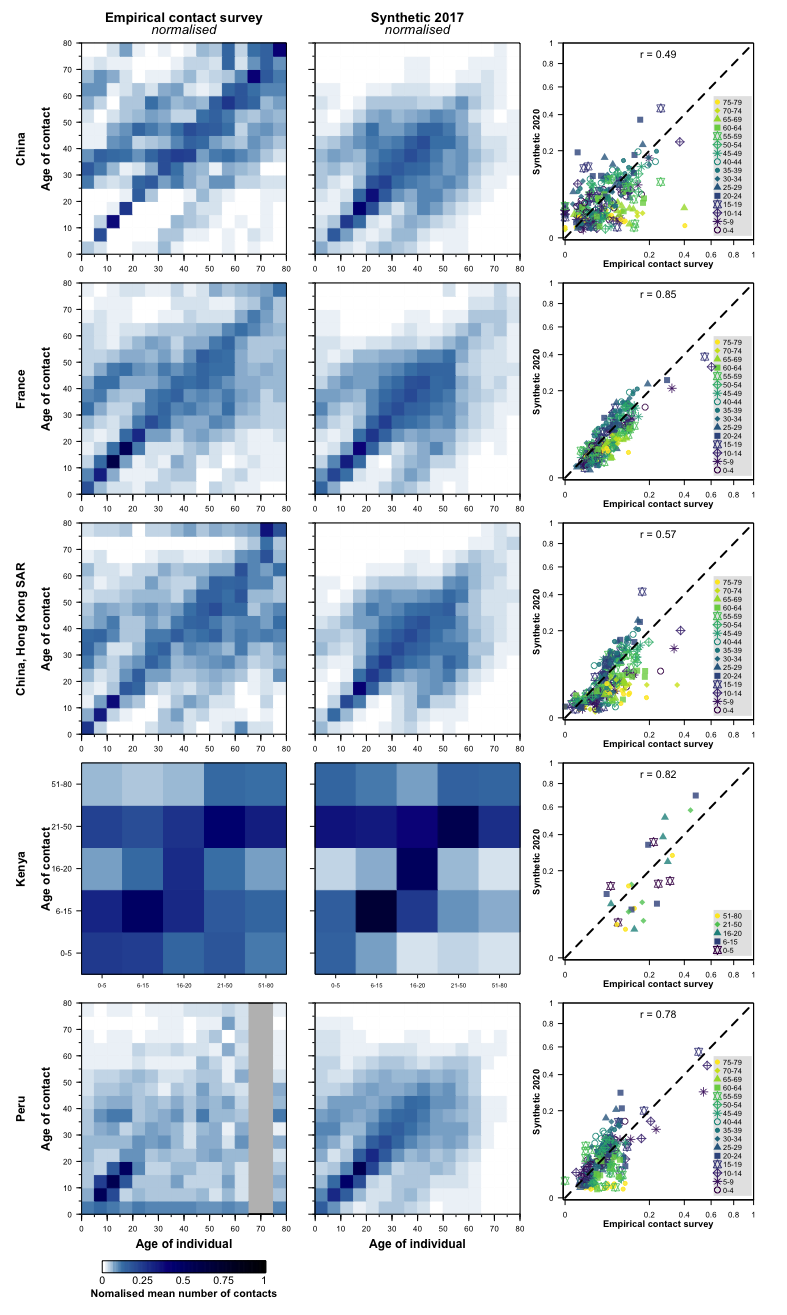

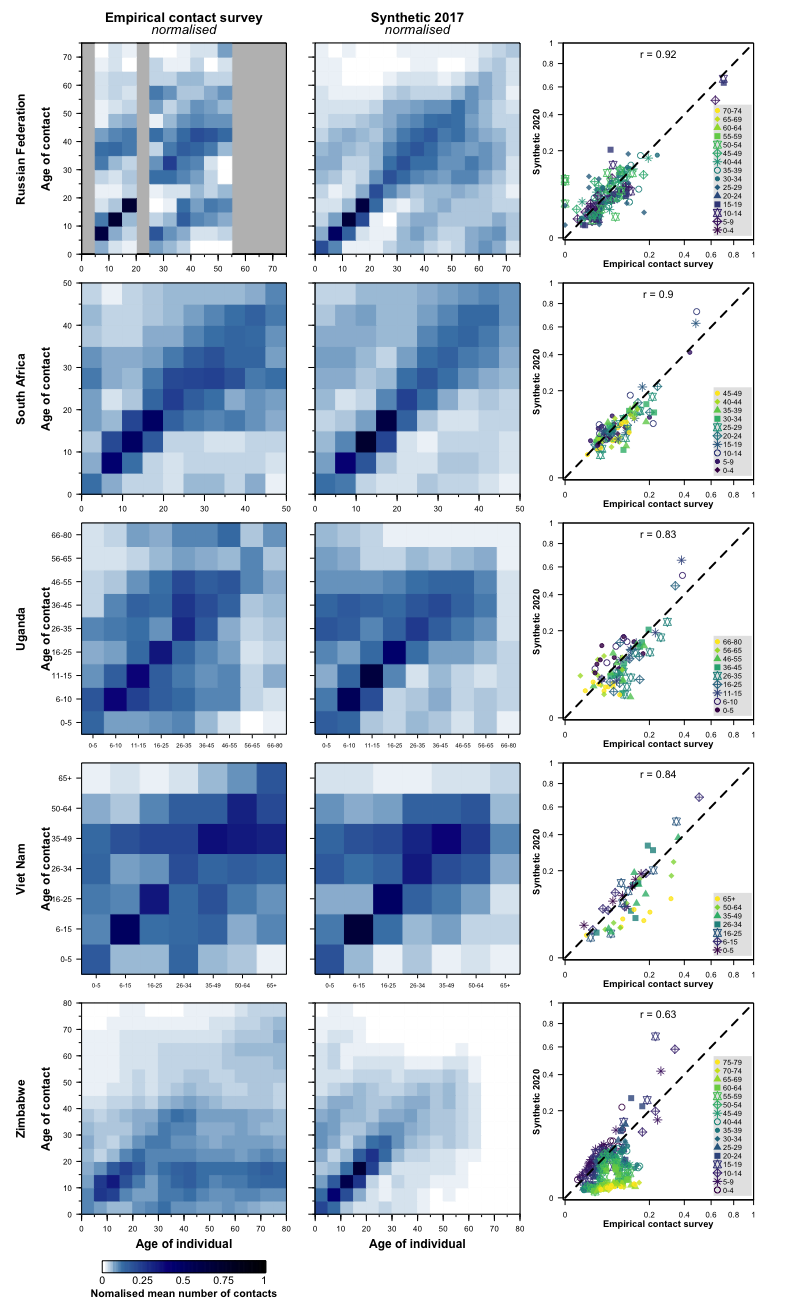


## Age-specific infection attack rate of COVID-19 and comparison of the empirical and updated synthetic age-specific contact matrices in ten geographical regions

The age-specific infection attack rate for the unmitigated epidemic under different contact matrices is shown in the boxplots with boxes bounded by the interquartile range (25^th^ and 75^th^ percentiles), the median in white and, whiskers spanning the 2.5–97.5^th^ percentiles (**Figure D**). Six contact matrices were considered in the COVID-19 modelling: the empirically-constructed contact matrices at the study-year and adjusted for the 2020 population, the 2017 synthetic matrices, and the updated synthetic matrices at the national, rural, or urban settings. Models parameterised with the empirical and synthetic matrices generated similar findings with few differences observed in the age-specific infection attack rate (**Figure D**) in age groups where the empirical matrices have missing or aggregated age groups (**Table D**). This finding means that synthetic contact matrices may be used in modelling outbreaks in settings for which empirical studies have yet to be conducted.

| 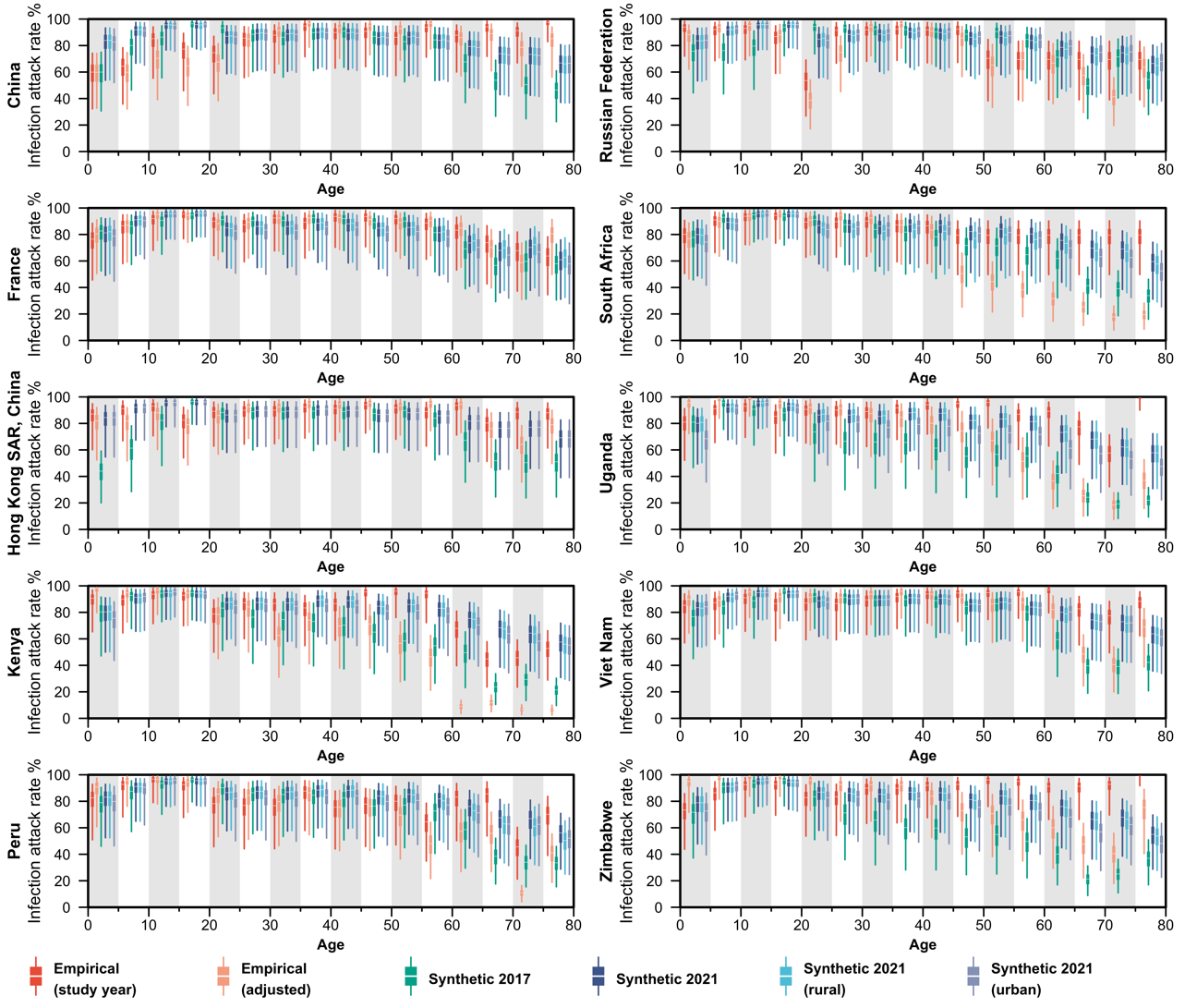 |
| --- |
| **Figure D.** Age-specific infection attack rate of COVID-19. |

The percentage change in case attack rate in each of the three intervention scenarios—20% physical distancing, 50% physical distancing, and lockdown—against the unmitigated epidemic under different contact matrices is shown in the boxplots with boxes bounded by the interquartile range (25^th^ and 75^th^ percentiles), median in white and, whiskers spanning the 2.5–97.5^th^ percentiles (**Figure E**). Six contact matrices were considered in the COVID-19 modelling: the empirically-constructed contact matrices at the study-year and adjusted for the 2020 population, the 2017 synthetic matrices, and the updated synthetic matrices at the national, rural, or urban settings.

| 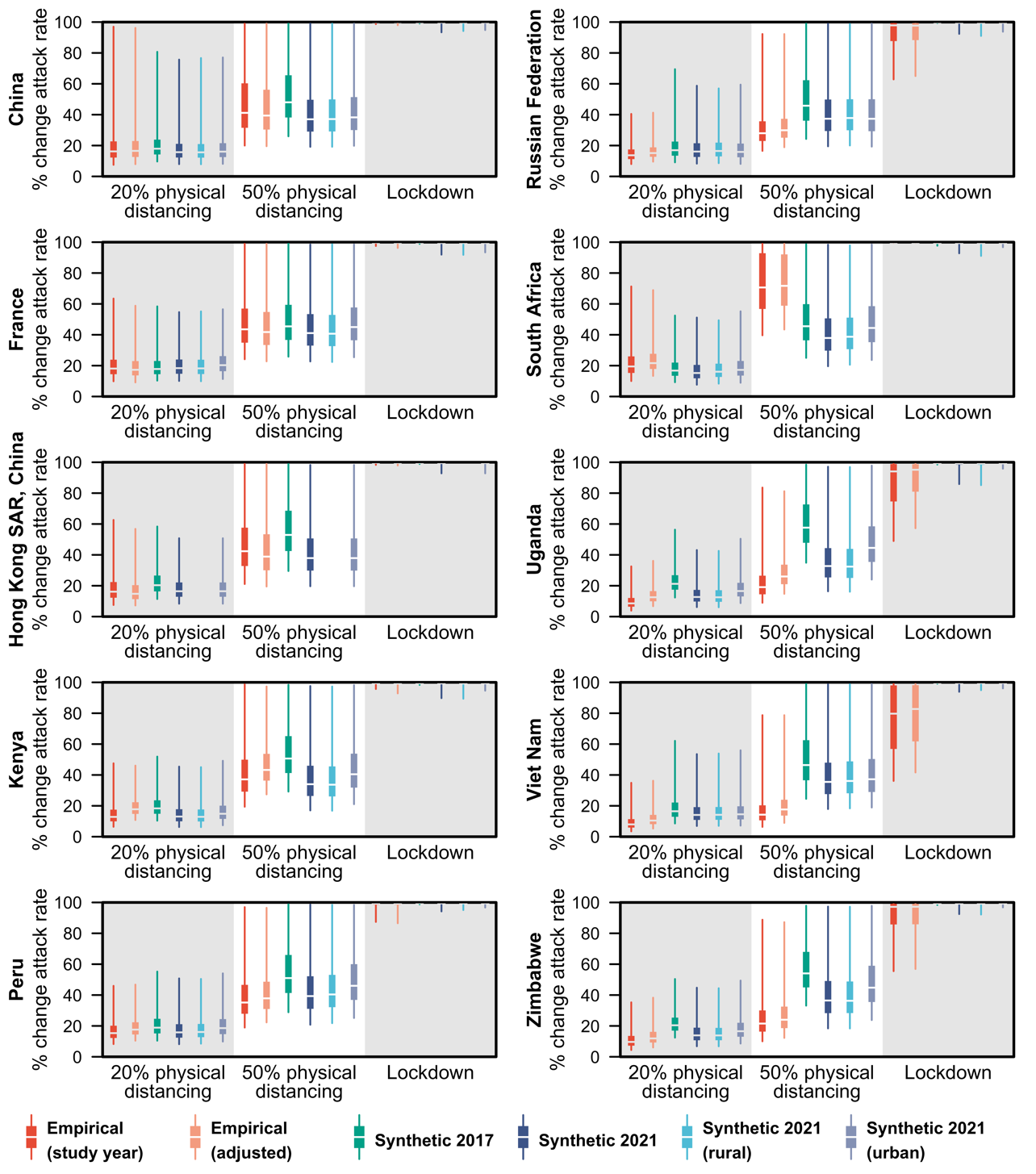 |
| --- |
| **Figure E.** Percentage change in case attack rate of COVID-19. |

| **Table D.** Possible reason for discrepancies in age-specific infection attack rates. |
| --- |

| **Discrepancies in age-specific infection attack rates** | **Survey-specific details** | **Possible reason for discrepancies** |
| --- | --- | --- |
| Underestimation of infection attack rate in the oldest age group (i.e., 75+ year-olds) in China, and Hong Kong SAR, China | Synthetic matrices report a lower proportion of contacts in other locations for individuals 75+ year-olds compared to empirical matrices in Shanghai and Hong Kong SAR | Individuals 75+ year old in two highly densely populated cities in China appear to report more contacts outside home and work than their counterparts in the POLYMOD surveyed countries in Europe. In the HK SAR survey, this may be due to a much larger proportion of older individuals choosing to use paper rather than online questionnaires; the survey found that individuals using paper surveys reported much higher numbers of contacts than those using online questionnaires. |
| Overestimation of infection attack rate in the 10–20-year-olds in China | Synthetic matrices report a higher proportion of contacts in other locations for individuals 10–20-year-olds compared to empirical matrices in Shanghai, China | Individuals 10–20-year-olds in Shanghai, China appear to report fewer contacts at other locations than their counterparts in the POLYMOD surveyed countries in Europe. |
| Differences in infection attack rate in individuals  50+ year-olds in Kenya | In the study in Kilifi, Kenya, individuals aged 21–50 were aggregated and the oldest age group in the survey is 51+ year-olds. | These differences are observed in age groups where the empirical matrices have aggregated age groups, which may obscure large differences within ages in that aggregated age group. |
| Differences in infection attack rate in individuals aged 70–74-year-olds in Peru | The study in San Marcos, Cajamarca-Peru did not survey any individuals aged 70–74-year-olds. | These differences are observed in age groups where the empirical matrices have missing age groups. Contacts in 70–74-year-olds in the empirical matrices are based on assuming reciprocity of contacts for younger people reporting contacts with this age group, which we know from other surveys may lead to incorrect estimations. |
| Differences in infection attack rate in individuals aged 20–24-year-olds in Russia | The study in Tomsk, Russia did not survey any individuals aged 20–24-year-olds. | These differences are observed in age groups where the empirical matrices have missing age groups. Contacts in 20–24-year-olds in the empirical matrices are based on assuming reciprocity of contacts for other people reporting contacts with this age group, which we know from other surveys may lead to incorrect estimations. |
| Differences in infection attack rate in individuals 50+ year-olds in South Africa | The oldest age group in the survey conducted in a South African township is 45+ year-olds. After adjusting for the South African 2020 population age composition, the differences narrow. | These differences are observed in age groups where the empirical matrices have aggregated age groups, which may obscure large differences within ages in that aggregated age group. |

| **Discrepancies in age-specific infection attack rates** | **Survey-specific details** | **Possible reason for discrepancies** |
| --- | --- | --- |
| Differences in infection attack rate in individuals 65+ year-olds in Uganda | The oldest age group in the survey conducted in a Sheema North Sub-District, Uganda is 65+ year-olds. These differences narrow after adjusting for Uganda’s 2020 population age composition. | These differences are observed in age groups where the empirical matrices have aggregated age groups, which may obscure large differences within ages in that aggregated age group. |
| Differences in infection attack rate in individuals 65+ year-olds in Vietnam | In the study in Red River Delta, North Vietnam, individuals aged 50–64 were aggregated and the oldest age group in the survey is 65+ year-olds. After adjusting for the Vietnamese 2020 population age composition, the differences narrow. | These differences are observed in age groups where the empirical matrices have aggregated age groups, which may obscure large differences within ages in that aggregated age group. |
| Differences in infection attack rate in individuals 65+ year-olds in Zimbabwe | Compared to the empirical matrices in Manicaland, Zimbabwe, the synthetic matrices underestimate the number of contacts for individuals 65+ year-olds at other locations. The differences narrow after adjusting for the 2020 population age composition in Zimbabwe. The survey was administered during the school holiday in one of the two sites; they found a lower number of contact was reported in schools. As school-going children are not in school, they could be spending more time at home and possibly with their grandparents; and this could be one possible reason for the high contacts in older individuals with the younger population. | Synthetic matrices report fewer contacts in other locations for individuals aged 65+ years compared to the empirical matrices in Manicaland, Zimbabwe. The differences narrow after adjusting for the 2020 population age composition in Zimbabwe. The survey was administered during the school holiday in one of the two sites; they found a lower number of contacts was reported in schools. As school-going children are not in school, they could be spending more time at home and possibly with their grandparents; and this could be one possible reason for the high contacts in older individuals with the younger population. |

## Degree of symmetry for empirical and synthetic matrices.

Given a matrix $M$, decompose $M$ into $M_{S}$ and $M_{A}$, the symmetric and skew-symmetric matrices corresponding to $M$ respectively, by defining $M_{S}=\frac{1}{2}(M-M^{T})$ and $M_{A}=\frac{1}{2}(M+M^{T})$, where $M^{T}$ is the transpose of $M$ (see e.g. Horn and Johnson, , Cambridge University Press 2012). Intuitively, the larger $M_{S}$ is compared to $M_{A}$, the more symmetric the matrix is.

Formally, we use the Frobenius norm of a matrix,

$$||M||=\sqrt{\sum|m_{i,j}|^{2}}$$

as a measure of its size, and define its degree of symmetry, $S(M)$ as

$$S(M)=(||M_{S}||-||M_{A}||)/(||M_{S}||+||M_{A}||).$$

**Table E** shows the degree of symmetry for empirical and synthetic matrices for locations where we have both. We find similarities apart from a few countries: our synthetic matrices are more symmetrical than the empirical ones for Shanghai, Hong Kong SAR, and Zimbabwe, but less for Kenya.

**Table E.** Degree of symmetry for empirical and synthetic matrices.

| **Region, Country** | **Degree of symmetry of empirical matrix** | **Degree of symmetry of synthetic matrix** |
| --- | --- | --- |
| Shanghai, China | 0.59 | 0.73 |
| France | 0.72 | 0.79 |
| Hong Kong SAR, China | 0.53 | 0.75 |
| Kenya | 0.64 | 0.59 |
| Peru | 0.72 | 0.76 |
| Russian Federation | Too many missing data | 0.75 |
| South Africa | 0.77 | 0.78 |
| Uganda | 0.70 | 0.76 |
| Vietnam | 0.61 | 0.71 |
| Zimbabwe | 0.45 | 0.68 |

#

1. Correspondence to [mark.jit@lshtm.ac.uk](mailto:mark.jit@lshtm.ac.uk). [↑](#footnote-ref-1)
