# Supplementary material for: Projecting contact matrices in 177 geographical regions: An update and comparison with empirical data for the COVID-19 era
Source: PLoS Comput Biol. 2021 Jul 26;17(7):e1009098. doi: 10.1371/journal.pcbi.1009098 (PMC8354454; doi:10.1371/journal.pcbi.1009098)
Supplement: Attachment — Submitted filename: responsetoreviewers2.pdf [file pcbi.1009098.s007.pdf]

Mark Jit BSc PhD MPH
Professor of Vaccine Epidemiology
Faculty of Epidemiology and Population Health
Department of Infectious Disease Epidemiology
London School of Hygiene & Tropical Medicine
Keppel Street
London, WC1E 7HT
United Kingdom

Phone : +44 (0) 207 327 7803
Email : mark.jit@lshtm.ac.uk

LONDON SCHOOL *of* HYGIENE &TROPICAL MEDICINE

6 May 2021

PLOS Computational Biology
Carlyle House
Carlyle Road
Cambridge CB4 3DN
United Kingdom

Dear Dr. Ferrari and Dr. Pitzer,

Second resubmission of *Projecting contact matrices in 177 geographical regions: an update and comparison with empirical data for the COVID-19 era*

Thank you for the positive feedback and for your remaining comments on the revised manuscript. We have now modified the main text to highlight the comparisons made in the Supplementary Material between empirical and synthetic matrices (see next page for details).

We therefore hope that our amendments address all remaining concerns from the Editors and the Reviewers.

Yours sincerely

Mark Jit

Response to comments from Editors and Reviewers

I thank the authors for their careful consideration of the reviewers' comments. I am satisfied with the work the authors have done to address these concerns, but I am concerned that a stronger case must be made in the main text about the qualitative and quantitative assessment of fit between the empirical and modeled matrices (presented in Supplementary Tables 4 and 5). These tables are quite helpful, but also put the magnitude of the fit into context (sometimes the fit is poor e.g. Zimbabwe in Table 5). At present there are only quite general references to the supplement, rather than explicit statements directing the reader to these specific results. I would encourage the authors to add specific text in the results to direct the reader to both Tables 4 and 5 (rather than just the corresponding sections) including a summary statement of what is to be found in the tables; e.g. a qualitative assessment of the fit and characteristics of each study (as in L348-354) and a quantitative comparison of the symmetry as a summary measure of fit.

Thank you for the suggestion. We have now added additional text to two sections of the manuscript in order to give specific details about the qualitative and quantitative assessment of fit, and to direct readers to specific Tables or Figures in the Supplementary Materials for more details.

To the Results, paragraph 4:

"The pronounced diagonals observed in all contact matrices are matched in the synthetic matrices (Fig 2A and 2B), as are the secondary diagonals indicating the occurrence of intergenerational mixing. The updated synthetic contact matrices show close similarities to empirical matrices (median correlation between normalised synthetic and empirical matrices 0.82, interquartile range 0.66–0.84). In most geographical regions, both matrices are similar in terms of symmetry. However, there are a few places such as Zimbabwe and China (Shanghai) where the synthetic matrix is more symmetrical than the empirical matrix, as the latter shows more weight above the diagonal (young people report more contacts with old people than vice versa). The degree of symmetry of both synthetic and empirical matrices in each region is compared in **Supplementary Materials Table 5**."

To the Results, paragraph 7:

"The choice of using synthetic or empirical matrices did not make a large difference to the infection attack rate for an unmitigated epidemic (**Supplementary Materials Figure 4**), or to the overall number of severe COVID-19 cases predicted in a mathematical model of SARS-CoV-2 transmission and disease across the three physical distancing interventions (Fig 4 and **Supplementary Materials Figure 5**). Where there were discrepancies, the relative magnitude of this discrepancy differed between countries. Differences were more marked in specific age groups (e.g. older people in Hong Kong SAR, Kenya, Peru, Uganda, Vietnam and Zimbabwe; 10-20 year olds in China; 20-24 year olds in Russia). The largest age-related differences could potentially be attributed to particular features of empirical survey design such as missing (Peru, Russia) or aggregated (Kenya, South Africa, Uganda, Vietnam) age groups, mode of questionnaire chosen by participants (Hong Kong SAR) and survey administration during school holidays (Zimbabwe) (See **Supplementary Materials Table 4** for details)."
