# Supplementary material for: Projecting contact matrices in 177 geographical regions: An update and comparison with empirical data for the COVID-19 era
Source: PLoS Comput Biol. 2021 Jul 26;17(7):e1009098. doi: 10.1371/journal.pcbi.1009098 (PMC8354454; doi:10.1371/journal.pcbi.1009098)
Supplement: Attachment — Submitted filename: responsetoreviewers.docx [file pcbi.1009098.s006.docx]

Mark Jit BSc PhD MPH

Professor of Vaccine Epidemiology

Faculty of Epidemiology and Population Health

Department of Infectious Disease Epidemiology

London School of Hygiene & Tropical Medicine

Keppel Street

London, WC1E 7HT

United Kingdom

Phone	**:** +44 (0) 207 327 7803

Email	: mark.jit@lshtm.ac.uk

12 April 2021

PLOS Computational Biology

Carlyle House

Carlyle Road

Cambridge CB4 3DN

United Kingdom

Dear Dr. Ferrari and Dr. Pitzer,

Resubmission of *Projecting contact matrices in 177 geographical regions: an update and comparison with empirical data for the COVID-19 era*

We would like to thank you and the reviewers for taking the time to consider our manuscript and for the comments provided in this revision. These have been very helpful, and we have adopted the editors’ and reviewers’ suggestions in our revised manuscript. Below you will find a detailed response to the comments.

We have in particular incorporated the following main changes in our manuscript:

1. Revised the presentation of Figures 2A and 2B, partly to enable better assessment of goodness-of-fit
2. Provided uncertainty estimates for the synthetic contact matrices
3. Added a new scenario for COVID-19-related interventions: a national lockdown based on the pooled setting-specific reduction observed during national lockdowns in different countries
4. Clarified the details of the hierarchical prior and added a description of the background contacts at workplaces and schools

We therefore hope the manuscript is now suitable for publication in *PLOS Computational Biology*.

Yours sincerely

Mark Jit

# Response to Reviewers

## Editorial comments

**With apologies to the authors for the long delay in this review. We have received three reviews that are complimentary of the work and I believe the concerns can be addressed with minor revisions. Specifically, both R3 and R4 make several minor comments that should be addressed in a revision.**

**R2 has suggested that some additional detail be provided on the out-of-sample fits. This could be done both quantitatively as the reviewer suggests, or qualitatively - e.g. describing the fit in more detail. For example, Figure 2A is carrying a lot of the weight for the claim the out-of-sample comparison is good. It seems that there is some additional nuance that could be discussed here and additional information that could be conveyed in the right-hand column of 2A. For example -- consider using different plotting characters (solid circles, open triangles, open squares) to represent matrix cells that are on, above, and below the diagonal, respectively. This would help to visualize whether or not the correlation is dominated by fits to different components of the contact matrix (for example the Shanghai matrix looks like it is dominated by good fit along the diagonal for young ages, but the off diagonals are mismatched - empirical is stronger above the diagonal, synthetic stronger below -- and poorly correlated for older ages; while other matrices appear to be very well correlated). This is just an example of a change to the plot, but the reviewers have requested some more substantive discussion of the out-of-sample fit, which I think would strengthen the manuscript.**

Response and action: We thank the editor for suggesting ways to improve the work. We have adopted the editor’s suggestion of using different plotting characters for the right-hand column of Figures 2A and 2B. Figures 2A and 2B are now shown as:

**Fig 2A. Comparison of the normalised empirical and synthetic age-specific contact matrices in five geographical regions**. The empirical matrices collected from contact surveys, modelled synthetic contact matrices, and the scatter plots of the entries in the observed (x-axis) and modelled (y-axis) contact matrices are presented. The correlation between the empirical and synthetic matrices are shown. The matrices are normalised such that its dominant eigenvalue is 1. To match the population surveyed in the empirical studies, the contact matrices from rural settings of Kenya and Peru are presented; and the contact matrix from urban settings of China is presented. No data are available in the grey regions.

**Fig 2B. Comparison of the normalised empirical and synthetic age-specific contact matrices in five geographical regions.** The empirical matrices collected from contact surveys, modelled synthetic contact matrices, and the scatter plots of the entries in the observed (x-axis) and modelled (y-axis) contact matrices are presented. The correlation between the empirical and synthetic matrices are shown. The matrices are normalised such that its dominant eigenvalue is 1. To match the population surveyed in the empirical studies, the contact matrices from rural settings of South Africa, Uganda, Vietnam, and Zimbabwe are presented; and the contact matrices from urban settings of the Russian Federation are presented. No data are available in the grey regions.

We think that the points raised may have been partially addressed by our previous comparison of the age-specific infection attack rate for the unmitigated epidemic under six different contact matrices in the Supplementary materials section 2.5. In Supplementary Table 4 of the section 2.5, we also describe possible reasons for discrepancies in outputs derived from models parameterised with the empirical and synthetic matrices. Supplementary Table 4 is as follows:

**Supplementary Table 4: Possible reason for discrepancies in age-specific infection attack rates**

| **Discrepancies in age-specific infection attack rates** | **Survey-specific details** | **Possible reason for discrepancies** |
| --- | --- | --- |
| Underestimation of infection attack rate in the oldest age group (i.e., 75+ year-olds) in China, and Hong Kong SAR, China | Synthetic matrices report a lower proportion of contacts in other locations for individuals 75+ year-olds compared to empirical matrices in Shanghai and Hong Kong SAR | Individuals 75+ year old in two highly densely populated cities in China appear to report more contacts outside home and work than their counterparts in the POLYMOD surveyed countries in Europe. In the HK SAR survey, this may be due to a much larger proportion of older individuals choosing to use paper rather than online questionnaires; the survey found that individuals using paper surveys reported much higher numbers of contacts than those using online questionnaires. |
| Overestimation of infection attack rate in the 10–20-year-olds in China | Synthetic matrices report a higher proportion of contacts in other locations for individuals 10–20-year-olds compared to empirical matrices in Shanghai, China | Individuals 10–20-year-olds in Shanghai, China appear to report fewer contacts at other locations than their counterparts in the POLYMOD surveyed countries in Europe. |
| Differences in infection attack rate in individuals 50+ year-olds in Kenya | In the study in Kilifi, Kenya, individuals aged 21–50 were aggregated and the oldest age group in the survey is 51+ year-olds. | These differences are observed in age groups where the empirical matrices have aggregated age groups, which may obscure large differences within ages in that aggregated age group. |
| Differences in infection attack rate in individuals aged 70–74-year-olds in Peru | The study in San Marcos, Cajamarca-Peru did not survey any individuals aged 70–74-year-old. | These differences are observed in age groups where the empirical matrices have missing age groups. Contacts in 70-74 year olds in the empirical matrices are based on assuming reciprocity of contacts for younger people reporting contacts with this age group, which we know from other surveys may lead to incorrect estimations. |
| Differences in infection attack rate in individuals aged 20–24-year-olds in Russia | The study in Tomsk, Russia did not survey any individuals aged 20–24-year-old. | These differences are observed in age groups where the empirical matrices have missing age groups. Contacts in 20-24 year olds in the empirical matrices are based on assuming reciprocity of contacts for other people reporting contacts with this age group, which we know from other surveys may lead to incorrect estimations. |
| Differences in infection attack rate in individuals 50+ year-olds in South Africa | The oldest age group in the survey conducted in a South African township is 45+ year-olds. After adjusting for the South African 2020 population age composition, the differences narrow. | These differences are observed in age groups where the empirical matrices have aggregated age groups, which may obscure large differences within ages in that aggregated age group. |
| Differences in infection attack rate in individuals 65+ year-olds in Uganda | The oldest age group in the survey conducted in a Sheema North Sub-District, Uganda is 65+ year-olds. These differences narrow after adjusting for Uganda’s 2020 population age composition. | These differences are observed in age groups where the empirical matrices have aggregated age groups, which may obscure large differences within ages in that aggregated age group. |
| Differences in infection attack rate in individuals 65+ year-olds in Vietnam | In the study in Red River Delta, North Vietnam, individuals aged 50–64 were aggregated and the oldest age group in the survey is 65+ year-olds. After adjusting for the Vietnamese 2020 population age composition, the differences narrow. | These differences are observed in age groups where the empirical matrices have aggregated age groups, which may obscure large differences within ages in that aggregated age group. |
| Differences in infection attack rate in individuals 65+ year-olds in Zimbabwe | Compared to the empirical matrices in Manicaland, Zimbabwe, the synthetic matrices underestimate the number of contacts for individuals 65+ year-olds at other locations. The differences narrow after adjusting for the 2020 population age composition in Zimbabwe. The survey was administered during the school holiday in one of the two sites; they found a lower number of contact was reported in schools. As school-going children are not in school, they could be spending more time at home and possibly with their grandparents; and this could be one possible reason for the high contacts in older individuals with the younger population. | Synthetic matrices report fewer contacts in other locations for individuals aged 65+ years compared to the empirical matrices in Manicaland, Zimbabwe. The differences narrow after adjusting for the 2020 population age composition in Zimbabwe. The survey was administered during the school holiday in one of the two sites; they found a lower number of contact was reported in schools. As school-going children are not in school, they could be spending more time at home and possibly with their grandparents; and this could be one possible reason for the high contacts in older individuals with the younger population. |

To address the point about the balance of diagonal, above diagonal and below diagonal elements, we have also calculated a measure of the symmetry^[1]^ for both the synthetic and empirical matrices, and added this to **Section 2.5** in the **Supplementary Materials**.

Given a matrix<https://www.codecogs.com/eqnedit.php?latex=M#0>, decompose<https://www.codecogs.com/eqnedit.php?latex=M#0> into<https://www.codecogs.com/eqnedit.php?latex=M_S#0> and<https://www.codecogs.com/eqnedit.php?latex=M_A#0>, the symmetric and skew-symmetric matrices corresponding to<https://www.codecogs.com/eqnedit.php?latex=M#0> respectively, by defining<https://www.codecogs.com/eqnedit.php?latex=M_S%20%3D%20%20%5Cfrac%7B1%7D%7B2%7D(M%20-%20M%5ET)#0> and<https://www.codecogs.com/eqnedit.php?latex=M_A%20%3D%20%20%5Cfrac%7B1%7D%7B2%7D(M%20%2B%20M%5ET)#0>, where<https://www.codecogs.com/eqnedit.php?latex=M%5ET#0> is the transpose of<https://www.codecogs.com/eqnedit.php?latex=M#0> (see e.g. Horn and Johnson, \textit{Matrix Analysis}, Cambridge University Press 2012). Intuitively, the larger<https://www.codecogs.com/eqnedit.php?latex=M_S#0> is compared to<https://latex-staging.easygenerator.com/eqneditor/editor.php?latex=M_A%24%2C%20the%20more%20symmetric%20the%20matrix%20is.#0>

Formally, we use the Frobenius norm of a matrix,

<https://www.codecogs.com/eqnedit.php?latex=%7C%7CM%7C%7C%20%3D%20%5Csqrt%7B%5Csum%7B%7Cm_%7Bi%2Cj%7D%7C%5E2%7D%7D#0>

as a measure of its size, and define its degree of symmetry,<https://www.codecogs.com/eqnedit.php?latex=S(M)#0> as

<https://www.codecogs.com/eqnedit.php?latex=S(M)%20%3D%20(%7C%7CM_S%7C%7C%20-%20%7C%7CM_A%7C%7C)%20%2F%20(%7C%7CM_S%7C%7C%20%2B%20%7C%7CM_A%7C%7C).#0>

Table 5 shows the degree of symmetry for empirical and synthetic matrices for locations where we have both. We find similarities apart from a few countries: our synthetic matrices are more symmetrical than the empirical ones for Shanghai and Zimbabwe, but less for Kenya.

**Supplementary Table 5. Degree of symmetry for empirical and synthetic matrices.**

| **Region, Country** | **Degree of symmetry of empirical matrix** | **Degree of symmetry of synthetic matrix** |
| --- | --- | --- |
| Shanghai, China | 0.59 | 0.70 |
| France | 0.72 | 0.73 |
| Hong Kong SAR, China | 0.53 | 0.68 |
| Kenya | 0.64 | 0.50 |
| Peru | 0.71 | 0.77 |
| Russian Federation | too much missing data | 0.66 |
| South Africa | 0.77 | 0.78 |
| Uganda | 0.70 | 0.71 |
| Viet Nam | 0.61 | 0.70 |
| Zimbabwe | 0.45 | 0.78 |

## Reviewer 1

**Reproducibility report has been uploaded as an attachment.**

Response and action: We thank the reviewer for assessing the reproducibility of the codes, and we are glad the reviewer was successful in reproducing the figures presented in the manuscript.

## Reviewer 2

1. **This study presents an update to the study by Prem et al., which is a useful tool for projecting contact matrices to countries that don’t have detailed data on contact structures. The original study was published in PLOS Comp Biol. This study makes some incremental but useful advances over the original, including providing breakdowns by urban/rural populations. Overall, this provides a great resource for the modeling community and is something that will continue to get a lot of use. The demonstration that the synthetic matrices yield similar predictions to the empirical matrices in most situations is also a useful validation of the approach, as is the discussion and table exploring possible reasons for discrepancies.**

Response and action: We thank the reviewer for the encouraging comments with regards to the potential contribution of our work to the modelling community. We are also appreciative that the reviewer took the time to raise the valuable comments that we address below.

2. **There are a number of ways in which data could be stratified sub-nationally. Urban/rural makes some sense and lines up with certain available data sources. But even within ‘urban’ areas there could be drastic differences in contact patterns, thinking of more and less affluent areas of major cities like London or New York. I'm not sure that much can be done about this, but the authors could discuss additional ways in which subnational projections could be generated.**

Response and action: The reviewer has raised a most valid point. We agree that there could be differences in contact patterns even within urban areas. In addition to age, location and rural/urban areas, person-to-person interactions depend on socioeconomic conditions (SES). However, neither review—Hoang et al.^[2]^ nor Liu et al.^[3]^—discuss SES as a variable collected in contact surveys: it was not routinely collected in POLYMOD nor most of the major contact surveys pre-COVID. While some contact surveys are now starting to incorporate SES markers (e.g. Jarvis et al.^[4]^, Kiti et al.), these are conducted during conditions of physical distancing/national lockdowns and so are not representative of baseline (pre-COVID) contacts that we are synthesising. Without empirical observations of how (baseline, and not in response to physical distancing measures) age- and location-specific contact patterns vary by SES and country, we are unable to make any inference and projections on them. We agree that this is a major area for future study, but we cannot assess this with available data at the current time. We have revised the **Discussion** to reflect the review’s point:

*Moreover, in addition to the rural-urban environment, age- and location-specific contact patterns could vary by socioeconomic conditions within countries.*

Could mention that neither review (Hoang or Liu) mentioned anything around SES as a variable in contact surveys, and it was not collected in POLYMOD. I think Comix UK collected this but it is not described in Jarvis et al. DHS may give household size by wealth quintile, but contact patterns are determined by more than household size so to do this you would need a breakdown by SES in the original contact surveys. I would take a quick look to see if any of the major surveys did include this variable.

3. **The github site contains the synthetic contact matrices for each country and for contact locations in each country. I didn’t see the associated uncertainty estimates, which might be useful to provide for use in certain modeling applications.**

Response and action: We thank the reviewer for this comment. The reviewer is right: providing uncertainty estimates for the synthetic contact matrices (age- and location-specific for each country) would be helpful to modellers. In this revision, we have included the 10th, 25th, 75th and 90th percentiles of the posterior distribution of the parameters and accompanying codes to generate the synthetic contact matrices (age- and location-specific for each country). They are now available on the GitHub site.

## Reviewer 3

1. **This manuscript presents updated results from a 2017 work from the same first author. The main changes with respect to the 2017 work include updating the datasets used to 2020 data, the inclusion of additional countries that account for an additional 1.3% of the world population and some methodological improvements in the estimation of the location specific contact matrices.**

**The methodology is sound and an adequate level of details is provided in order to assess the results, without the need to resort to the original 2017 paper.**

Response and action: We are grateful for the reviewer’s positive comments. We thank the reviewer for taking the time to raise the valuable suggestions that we address below. We have separated out the comments into distinct items for ease of response.

2. **I have one major point that I think should be explored and discussed, more specifically:**

**- When simulating the impact of physical distancing on COVID-19 spread, the authors use percentages of physical distancing are assumed uniform across all locations. However this is usually not the case when intervention strategies are put into place, for example when school closure, teleworking and the closure of leisure activities are implemented. Since contacts at home, school, work and other location for the synthetic matrices are generated according to different methods and assumptions, it would be interesting to understand how reduction in contacts that are location specific affect the results of the simulations. This would help in assess the possible bias that the synthetic matrices induce, especially when are used to assess the impact of intervention strategies.**

Response: To make our physical distancing scenarios more representative of what happened in many parts of the world, we have replaced our scenario comparing the impact of shielding with a scenario comparing the impact of a national lockdown, where we applied the pooled mean reduction in setting-specific contacts (i.e., at home, school, work, and other places) as observed in lockdowns implemented in several countries during the COVID-19 pandemic. We chose to show the combined rather than the individual effect on each setting to minimise the number of scenarios shown in the paper, keeping the reader’s attention to the comparison of different contact matrices rather than the potential impact of different interventions – the latter is not the focus of this analysis and would require country-specific data rather than global averages.

Action: We have since revised the subsection in the **Methods** section, it now reads:

## *Impact on modelling of interventions*

*We compare the difference in relative reduction of COVID-19 cases between using the empirical and synthetic matrices in models of COVID-19 epidemics in ten geographical regions—China, France, Hong Kong SAR, Kenya, Peru, the Russian Federation, South Africa, Uganda, Vietnam and Zimbabwe—using an age-stratified compartmental model (13,25). We model an unmitigated epidemic and three intervention scenarios: 20% physical distancing, 50% physical distancing, and national lockdown. In all intervention scenarios, we assume a 50% reduction in transmission from individuals with clinical symptoms through self-isolation. In addition, we assume the following: (i) 20% physical distancing: 20% reduction in transmission outside of the household, (ii) 50% physical distancing: 50% reduction in transmission outside of the household, (iii) national lockdown: where we applied the pooled mean reduction in setting-specific contacts (i.e. at home, school, work, and other places) as observed in lockdowns implemented in several countries during the COVID-19 pandemic (52–56). We considered six contact matrices when modelling the interventions to the COVID-19 pandemic: the empirically-constructed contact matrices at the study-year and adjusted for the 2020 population, the 2017 synthetic matrices, and the updated synthetic matrices at the national, rural, or urban settings. More details of the model can be found in sections 1.7 and 2.5 of the* ***Supplementary materials****.*

Figure 4 is now shown as:

**Fig 4. Reduction in cases due to interventions in models of COVID-19 epidemics under three intervention scenarios in ten geographical regions using the empirical and synthetic matrices.** The reduction in cases in each of the three intervention scenario—20% physical distancing, 50% physical distancing, and lockdown—against the unmitigated epidemic under different contact matrices is shown in the boxplots with boxes bounded by the interquartile range (25th and 75th percentiles), median in white and, whiskers spanning the 2.5–97.5th percentiles. Six contact matrices were considered in the COVID-19 modelling: the empirically-constructed contact matrices at the study-year and adjusted for the 2020 population, the 2017 synthetic matrices, and the updated synthetic matrices at the national, rural, or urban settings.

3. **I also have some minor comments that could be addressed by the authors to improve the manuscript readability that I list below.**

**“Assessing such policies requires differences between contact patterns in rural and urban environments to be quantified, which has previously been done only for a few countries (28,29)”. Also Melegaro et al. (already cited as citation 50 in the manuscript) records different contact patterns in rural and urban setting, and could be added to the sentence.**

Response and action: We thank the reviewer for pointing this out, we have now cited Melegaro et al. (reference number 30) at that sentence, as follows:

*Assessing such policies requires differences between contact patterns in rural and urban environments to be quantified, which has previously been done only for a few countries (28–30).*

4. **“In these studies, individuals in rural settings documented more contacts at home than their urban counterparts”. Why is the number of contacts at home discussed and not the results in terms of total number of contacts? In case there is no specific reason (e.g. relation to “stay at home” interventions), the overall result should be stressed here too.**

Response and action: The reviewer has raised a valid point. In the revised manuscript, we now discuss the difference in the total number of contacts across rural and urban settings. We have since revised the sentences in the **Introduction** section, it now reads:

*In these studies, individuals in rural settings documented more contacts at home than their urban counterparts (28,29). However, individuals in rural settings in Zimbabwe (30) reported a lower total number of contacts than those in peri-urban settings. The study in Southern China observed no qualitative difference in overall contact patterns between rural and urban populations (28).*

5. **The authors link the contacts at work with the matrix of the working population. However, when a participant makes a contact “at work” it could be a contact with a “customer” , who would not report that contact as happening at work. My point is that imposing the structure of the working population both at the participant level and at the contact level may induce a bias in the contact matrix. Could the authors discuss this issue more in depth?**

Response and action: We thank the reviewer for the opportunity to clarify. If a participant makes a contact at the workplace with a customer, it would be captured in the background contact parameters. Specifically, contacts made with visitors at home, workplace or school is allowed via background contact parameters—<https://www.codecogs.com/eqnedit.php?latex=%5Cdelta_H#0>,<https://www.codecogs.com/eqnedit.php?latex=%5Cdelta_W#0> and<https://www.codecogs.com/eqnedit.php?latex=%5Cdelta_S#0>—in the Bayesian hierarchical model.

We have included the following discussion in the **Supplementary Materials** section 1.8.

*The Poisson mean parameter has the general form*<https://www.codecogs.com/eqnedit.php?latex=%5Cmu_%7Bi%2C%5Calpha%7D%5EL%20%3D%20%5Csigma_i%20%5Clambda_%7Ba_i%2C%5Calpha%7D%5EL#0> *and varies across locations. In this model,*<https://www.codecogs.com/eqnedit.php?latex=%5Csigma_i#0> *is a random effect belonging to individual*<https://www.codecogs.com/eqnedit.php?latex=i#0> *which characterises differences in social activity levels across locations and allows for greater than Poisson variability in the number of contacts.*

*The mean was*<https://www.codecogs.com/eqnedit.php?latex=%5Cmu_%7Bi%2C%5Calpha%7D%5EH%20%3D%20%5Csigma_i%20%5Clambda_%7Ba_i%2C%5Calpha%7D%5EH%20(%5Cnu_%7Bi%2C%5Calpha%7D%20%2B%20%5Cdelta_H)#0> *for home contacts,*<https://www.codecogs.com/eqnedit.php?latex=%5Cmu_%7Bi%2C%5Calpha%7D%5EW%20%3D%20%5Csigma_i%20%5Clambda_%7Ba_i%2C%5Calpha%7D%5EW%20(w_i%20%2B%20%5Cdelta_W)#0> *for work contacts,*<https://www.codecogs.com/eqnedit.php?latex=%5Cmu_%7Bi%2C%5Calpha%7D%5ES%20%3D%20%5Csigma_i%20%5Clambda_%7Ba_i%2C%5Calpha%7D%5ES%20(s_i%20%2B%20%5Cdelta_S)#0> *for school contacts and*<https://www.codecogs.com/eqnedit.php?latex=%5Cmu_%7Bi%2C%5Calpha%7D%5EO%20%3D%20%5Csigma_i%20%5Clambda_%7Ba_i%2C%5Calpha%7D%5EO#0> *for contacts at all other locations. The number of cohabitants of*<https://www.codecogs.com/eqnedit.php?latex=i#0> *of age*<https://www.codecogs.com/eqnedit.php?latex=%5Calpha#0>*,*<https://www.codecogs.com/eqnedit.php?latex=%5Cnu_%7Bi%2C%5Calpha%7D#0>*, represents household age structure, while*<https://www.codecogs.com/eqnedit.php?latex=w_i#0> *and*<https://www.codecogs.com/eqnedit.php?latex=s_i#0> *indicate if*<https://www.codecogs.com/eqnedit.php?latex=i#0> *went to work or school on the day of the survey. Contact with visitors at home, workplace or school is allowed via background contact parameters*<https://www.codecogs.com/eqnedit.php?latex=%5Cdelta_H#0>*,*<https://www.codecogs.com/eqnedit.php?latex=%5Cdelta_W#0> *and*<https://www.codecogs.com/eqnedit.php?latex=%5Cdelta_S#0>*.*

6. **“We compare the difference in relative reduction of COVID-19 cases between using the empirical and synthetic matrices in models of COVID-19 epidemics […] using an age-stratified compartmental model (13,25)”. The authors should specify here that the model is presented in more depth in the additional material.**

Response and action: We thank the reviewer for this comment. We have added references to sections 1.7 and 2.5 of the Supplementary materials where there are more details.

7. **“We model an unmitigated epidemic and three intervention scenarios: 20% physical distancing, 50% physical distancing, and shielding.” Are these assumed reduction in physical distancing in line with the reduction measured from empirical contact data measured during and after lockdown? There are several examples in literature that could be used as a reference (Zhang et al. 2020, Jarvis et al. 2020, Coletti et al. 2020, Latsuzbaia et al. 2020).**

Response: We thank the reviewer for this comment.

Based on the reviewer’s suggestion, we have now included an intervention scenario based on empirical contact data. However, the degree to which physical distancing is practised differs by country and changes over time (pre-print review by Liu et al.^[5]^). The reduction in contacts as observed in the longitudinal CoMix survey has been estimated to be as much as 70% during the first national lockdown (March 2020) but has substantially changed over time^[6]^. In this paper, we show the impact of some interventions to illustrate the effect of the assumed mixing matrix on model results, and to cross-validate the modelling results of these synthetic matrices with empirical estimates, without the additional complexity needed to model the interventions as they have been implemented in each country. However, we now replaced the shielding scenario (also suggested by Reviewer 4 in comment 8) with a scenario where we modelled a national lockdown based on the pooled mean setting-specific reduction observed during national lockdowns in different countries.

Action: We have since revised the subsection in the **Methods** section, it now reads:

## *Impact on modelling of interventions*

*We compare the difference in relative reduction of COVID-19 cases between using the empirical and synthetic matrices in models of COVID-19 epidemics in ten geographical regions—China, France, Hong Kong SAR, Kenya, Peru, the Russian Federation, South Africa, Uganda, Vietnam and Zimbabwe—using an age-stratified compartmental model (13,25). We model an unmitigated epidemic and three intervention scenarios: 20% physical distancing, 50% physical distancing, and national lockdown. In all intervention scenarios, we assume a 50% reduction in transmission from individuals with clinical symptoms through self-isolation. In addition, we assume the following: (i) 20% physical distancing: 20% reduction in transmission outside of the household, (ii) 50% physical distancing: 50% reduction in transmission outside of the household, (iii) national lockdown: where we applied the pooled mean reduction in setting-specific contacts (i.e. at home, school, work, and other places) as observed in lockdowns implemented in several countries during the COVID-19 pandemic (52–56). We considered six contact matrices when modelling the interventions to the COVID-19 pandemic: the empirically-constructed contact matrices at the study-year and adjusted for the 2020 population, the 2017 synthetic matrices, and the updated synthetic matrices at the national, rural, or urban settings. More details of the model can be found in sections 1.7 and 2.5 of the* ***Supplementary materials****.*

Figure 4 is now shown as:

**Fig 4. Reduction in cases due to interventions in models of COVID-19 epidemics under three intervention scenarios in ten geographical regions using the empirical and synthetic matrices.** The reduction in cases in each of the three intervention scenario—20% physical distancing, 50% physical distancing, and lockdown—against the unmitigated epidemic under different contact matrices is shown in the boxplots with boxes bounded by the interquartile range (25th and 75th percentiles), median in white and, whiskers spanning the 2.5–97.5th percentiles. Six contact matrices were considered in the COVID-19 modelling: the empirically-constructed contact matrices at the study-year and adjusted for the 2020 population, the 2017 synthetic matrices, and the updated synthetic matrices at the national, rural, or urban settings.

8. **“Prospective surveys have been shown to be less prone to recall bias compared to their retrospective counterpart (43), but it is often more challenging to find willing participants for prospective surveys.” However, a study by Beutels et al. 2006 found no appreciable effect of retrospective vs prospective surveying, so this issue may be different, depending on the survey setting.**

Response and action: We thank the reviewer for sharing this reference. We have since included the reference and edit the text in the **Discussion** section, as follow:

*In Hong Kong, prospective surveys have been shown to be less prone to recall bias compared to their retrospective counterpart (44), but it is often more challenging to find willing participants for prospective surveys. However, a study in Belgium (58) found no appreciable effect between retrospective and prospective surveying.*

**References**

**Beutels P.et al. Social mixing patterns for transmission models of close contact infections: exploring self-evaluation and diary-based data collection through a web-based interface. Epidemiology & Infection.**

**Zhang, J. et al. Changes in contact patterns shape the dynamics of the covid-19 outbreak in china. Science**

**Jarvis, C. I. et al. Quantifying the impact of physical distance measures on the transmission of COVID-19 in the UK. BMC Medicine**

**Coletti P. et al. CoMix: comparing mixing patterns in the Belgian population during and after lockdown. Scientific Reports.**

**Latsuzbaia, A., Herold, M., Bertemes, J.-P. & Mossong, J. Evolving social contact patterns during the covid-19 crisis in Luxembourg. PLOS ONE 15,**

## Reviewer 4

1. **This is an impressive update to the earlier version of the matrices.**

Response and action: We thank the reviewer for the positive comments and for taking the time to raise the valuable comments that we address below.

2. **Abstract: “We found that the synthetic contact matrices reproduce the main traits of the contact patterns in the empirically-constructed contact matrices.” Needs to be more specific - what is the measure of similarity and how good is it.**

Response and action: The reviewer has a valid point, and we improved the clarity of this sentence in the **Abstract**; it now reads:

*We found that the synthetic contact matrices show qualitative similarities to the contact patterns in the empirically-constructed contact matrices.*

3. **No page numbers or line numbers! Using page numbers from the pdf.**

Response and action: We apologise for this oversight, and we thank the reviewer for their patience. We have included page and line numbers in the revised manuscript.

4. **Page 9, Line +3; These contact matrices have been useful and have been used to explore policy space. However, it is not clear that their shape is preserved in the presence of a severe pathogen. The CoMix study from the UK gets to this to some degree. The work presented here is still incredibly useful, but this section does need some caveats so prevent modelling studies making overly strong assumptions.**

Response: We are glad the reviewer believes our work is useful and policy-relevant. We thank the reviewer for this comment and for giving us the opportunity to clarify. Unlike the matrices from the CoMix studies, these synthetic matrices are baseline matrices and are not meant to be used in the same way as the CoMix matrices which give real-time estimates during interventions. In our response to comments 2 and 8 by Reviewer 3, we mentioned that we explored the impact of some non-pharmaceutical interventions to illustrate the effect of the assumed mixing matrix on model results. Understanding the impact of different interventions would require country-specific data rather than global averages, and it is outside the scope of this paper. However, we have now included a scenario populated with empirically collected data during the COVID-19 pandemic.

Action: We have since revised the subsection in the **Methods** section, it now reads:

## *Impact on modelling of interventions*

*We compare the difference in relative reduction of COVID-19 cases between using the empirical and synthetic matrices in models of COVID-19 epidemics in ten geographical regions—China, France, Hong Kong SAR, Kenya, Peru, the Russian Federation, South Africa, Uganda, Vietnam and Zimbabwe—using an age-stratified compartmental model (13,25). We model an unmitigated epidemic and three intervention scenarios: 20% physical distancing, 50% physical distancing, and national lockdown. In all intervention scenarios, we assume a 50% reduction in transmission from individuals with clinical symptoms through self-isolation. In addition, we assume the following: (i) 20% physical distancing: 20% reduction in transmission outside of the household, (ii) 50% physical distancing: 50% reduction in transmission outside of the household, (iii) national lockdown: where we applied the pooled mean reduction in setting-specific contacts (i.e. at home, school, work, and other places) as observed in lockdowns implemented in several countries during the COVID-19 pandemic (52–56). We considered six contact matrices when modelling the interventions to the COVID-19 pandemic: the empirically-constructed contact matrices at the study-year and adjusted for the 2020 population, the 2017 synthetic matrices, and the updated synthetic matrices at the national, rural, or urban settings. More details of the model can be found in sections 1.7 and 2.5 of the* ***Supplementary materials****.*

Figure 4 is now shown as:

**Fig 4. Reduction in cases due to interventions in models of COVID-19 epidemics under three intervention scenarios in ten geographical regions using the empirical and synthetic matrices.** The reduction in cases in each of the three intervention scenario—20% physical distancing, 50% physical distancing, and lockdown—against the unmitigated epidemic under different contact matrices is shown in the boxplots with boxes bounded by the interquartile range (25th and 75th percentiles), median in white and, whiskers spanning the 2.5–97.5th percentiles. Six contact matrices were considered in the COVID-19 modelling: the empirically-constructed contact matrices at the study-year and adjusted for the 2020 population, the 2017 synthetic matrices, and the updated synthetic matrices at the national, rural, or urban settings.

5. **10,+10; use of “proclivity” not clear here**

Response and action: The proclivity of contact between individuals refers to the typical contact rate per day. On reflection of this comment, we can see how the word “proclivity” may be unclear, and we have replaced it with “frequency”. We have clarified that sentence in the revision as follows:

*The*<https://www.codecogs.com/eqnedit.php?latex=%5Clambda_(a_i%2C%5Calpha)%5EL#0> *parameter denotes the frequency of contact between individuals (or contact rates per day) from two age groups,*<https://www.codecogs.com/eqnedit.php?latex=a#0> *and*<https://latex-staging.easygenerator.com/eqneditor/editor.php?latex=%5Calpha#0>*, at location*<https://www.codecogs.com/eqnedit.php?latex=L#0> *and it is the key estimand.*

6. **10,+13; How is the “spatial” smoothness. That doesn’t quite make sense to me.**

Response: We thank the reviewer for the opportunity to clarify. On reflection on this comment, we can see how the word “spatial” imparts ambiguity, and we have revised it. We smooth the estimates for nearby age groups using a hierarchical prior.

Action: We have revised the sentence in the **Methods** section, it now reads:

*Because the number of contacts should be comparable for individuals of similar ages, we imposed spatial smoothness between successive age groups for the*<https://www.codecogs.com/eqnedit.php?latex=%5Clambda_%7Ba_i%2C%5Calpha%7D%5EL#0> *parameter as described in section 1.8 in the* ***Supplementary Materials****.*

We have also included the following discussion in the **Supplementary Materials** section 1.8:

*The*<https://www.codecogs.com/eqnedit.php?latex=%5Clambda_%7Ba_i%2C%5Calpha%7D%5EL#0> *parameter was given a hierarchical prior to impose smoothness between successive age groups, i.e.:*

<https://www.codecogs.com/eqnedit.php?latex=%5Clog%5Clambda_%7Ba_i%2C%5Calpha%7D%5EL%3D%5Csum_%7B%5Cmathcal%7BA%7D%2CA%3A%5Cmathcal%7BA%7D%2CA%5Cin%5Cmathcal%7BN%7D_%7Ba_i%2C%5Calpha%7D%7D%20%20%5Cfrac%7B%5Cepsilon_%7B%5Cmathcal%7BA%7D%2CA%7D%5EL%7D%7B%7C%5Cmathcal%7BN%7D_%7Ba_i%2C%5Calpha%7D%7C%7D#0>

*where*<https://www.codecogs.com/eqnedit.php?latex=%5Cepsilon_%7B%5Cmathcal%7BA%7D%2CA%7D%5EL#0> *is a hyperparameter of*<https://www.codecogs.com/eqnedit.php?latex=%5Clambda_%7Ba_i%2C%5Calpha%7D%5EL#0> *and*<https://www.codecogs.com/eqnedit.php?latex=%5Cmathcal%7BN%7D_%7Ba_i%2C%5Calpha%7D#0> *is the set of (up to) four adjacent age groups together with*<https://www.codecogs.com/eqnedit.php?latex=(a_i%2C%5Calpha)#0> *itself (presented in* ***Figure 2****). The adjacent age groups (light blue regions) together with*<https://www.codecogs.com/eqnedit.php?latex=(a_i%2C%5Calpha)#0> *(dark blue regions i.e., A, B, and C) are elements of*<https://www.codecogs.com/eqnedit.php?latex=%5Cmathcal%7BN%7D_%7Ba_i%2C%5Calpha%7D#0>*.*

***Figure 2. Set of up to four adjacent age groups together with***<https://www.codecogs.com/eqnedit.php?latex=(a_i%2C%5Calpha)#0>***.***

*Contact surveys conducted in several countries^[7]^ indicate strong assortativity of social contacts with age. Hence, to allow the number of contacts to be comparable for individuals of similar ages, the hierarchical prior was designed to impose smoothness between successive age groups (right panel of* ***Figure 3****). This smoothness on the prior distribution of the parameter*<https://www.codecogs.com/eqnedit.php?latex=%5Clambda_%7Ba_i%2C%5Calpha%7D%5EL#0> *to allow the number of contacts to be comparable for individuals of similar ages (****Figure 3****). The hyperparameter*<https://www.codecogs.com/eqnedit.php?latex=%5Cepsilon_%7B%5Cmathcal%7BA%7D%2CA%7D%5EL#0> *has a non-informative prior distribution of*<https://www.codecogs.com/eqnedit.php?latex=%5Ctextrm%7BNormal%7D%20%5Csim%20(0%2C100%5E2)#0> *as represented on the left of* ***Figure 3****.*

***Figure 3. Smoothness between successive age groups on the prior distribution of the parameter.***

Consider this example: because we split the population age structure into five-year bands, an individual aged 5-year-old (cell C in **Figure 2**) and an individual aged 6-year-old (to the right of cell C in **Figure 2**) would be binned into different age categories. However, there may be some similarities in their mixing patterns, which we try to account for via the prior distribution of parameter<https://www.codecogs.com/eqnedit.php?latex=%5Clambda_%7Ba_i%2C%5Calpha%7D%5EL#0>.

7. **13,+14; Suggest a zenodo archive of the github with a doi as a permanent record of the code that goes with the paper**

Response and action: We thank the reviewer for this suggestion. We agree that Zenodo would be a better repository and will do so once we have a final version of the model after the peer-review.

8. **13,-6; shielding: this needs a little more discussion. There isn’t really evidence for this as an effective policy. It represents a desirable outcome, but wasn’t really achieved by a deliberate policy anywhere. School closure as a reduction in children’s contacts would be a better generic example.**

Response: We agree with the reviewer that shielding has not been implemented on a large scale as a response to COVID-19 in most settings, and have now replaced this scenario with a scenario implementing a lockdown with pooled mean reductions in setting-specific contacts (at home, school, work, and other places) as observed in multiple lockdowns implemented during the COVID-19 pandemic as a more generic example. We did not include a school-closure-only intervention because of the controversy over school closures during the COVID-19 pandemic, and strong views from many stakeholders that it should be a last resort when workplace closures are insufficient, rather than as a stand-alone intervention (e.g. report from the European Centre for Disease Prevention and Control^[8]^).

Action: We have since revised the subsection in the **Methods** section, it now reads:

## *Impact on modelling of interventions*

*We compare the difference in relative reduction of COVID-19 cases between using the empirical and synthetic matrices in models of COVID-19 epidemics in ten geographical regions—China, France, Hong Kong SAR, Kenya, Peru, the Russian Federation, South Africa, Uganda, Vietnam and Zimbabwe—using an age-stratified compartmental model (13,25). We model an unmitigated epidemic and three intervention scenarios: 20% physical distancing, 50% physical distancing, and national lockdown. In all intervention scenarios, we assume a 50% reduction in transmission from individuals with clinical symptoms through self-isolation. In addition, we assume the following: (i) 20% physical distancing: 20% reduction in transmission outside of the household, (ii) 50% physical distancing: 50% reduction in transmission outside of the household, (iii) national lockdown: where we applied the pooled mean reduction in setting-specific contacts (i.e. at home, school, work, and other places) as observed in lockdowns implemented in several countries during the COVID-19 pandemic (52–56). We considered six contact matrices when modelling the interventions to the COVID-19 pandemic: the empirically-constructed contact matrices at the study-year and adjusted for the 2020 population, the 2017 synthetic matrices, and the updated synthetic matrices at the national, rural, or urban settings. More details of the model can be found in sections 1.7 and 2.5 of the* ***Supplementary materials****.*

Figure 4 is now shown as:

**Fig 4. Reduction in cases due to interventions in models of COVID-19 epidemics under three intervention scenarios in ten geographical regions using the empirical and synthetic matrices.** The reduction in cases in each of the three intervention scenario—20% physical distancing, 50% physical distancing, and lockdown—against the unmitigated epidemic under different contact matrices is shown in the boxplots with boxes bounded by the interquartile range (25th and 75th percentiles), median in white and, whiskers spanning the 2.5–97.5th percentiles. Six contact matrices were considered in the COVID-19 modelling: the empirically-constructed contact matrices at the study-year and adjusted for the 2020 population, the 2017 synthetic matrices, and the updated synthetic matrices at the national, rural, or urban settings.

9. **15,+10; is HAM defined? What was the range of correlations? This is the key bit of the paper. I suggest a supp in figure of actual versus predicted heat-map-grid charts of predicted versus observed for the polymod data**

Response and action: The reviewer is right, and we are grateful that the missing definition has been identified! The household age matrix (HAM) of country<https://www.codecogs.com/eqnedit.php?latex=c#0>,<https://www.codecogs.com/eqnedit.php?latex=%5Cmathbf%7Bh%7D_%7Ba%2C%5Calpha%7D%5Ec#0>, represents the mean number of household members of age<https://www.codecogs.com/eqnedit.php?latex=%5Calpha#0> of an individual aged<https://www.codecogs.com/eqnedit.php?latex=a#0>. The median correlation between the empirical (or *actual*) and modelled HAM 0.92, with an interquartile range 0.85–0.95 and a range 0.54–0.97. We described the methods to reconstruct the empirical HAM for the POLYMOD and DHS countries in Section 1.2.2 of the Supplementary materials. The empirical household age structures (i.e., from the data) and the modelled household age structures (i.e., the leave-one-out validation, assuming no available household data) for each POLYMOD and DHS countries are presented in the first and second panels respectively of the figures in Section 2.1 of the Supplementary materials (an example is presented in **Figure 3**). In the third panel of **Figure 3 (below)**, a scatter plot of the entries in the observed (x-axis) and modelled (y-axis) HAM.

**Figure 3. An example of the empirical and modelled HAM comparison.**

We have included definitions of HAM in revised the sentence in the **Methods** section and provided links to sections 1.2.2 and 2.1 of the Supplementary materials at the end of the paragraph, as follows:

*We reconstructed the empirical household age structures for the POLYMOD and DHS countries with high fidelity (median correlation between the observed and modelled household age matrix (HAM) 0.92, with an interquartile range 0.85–0.95) (See* ***Supplementary Materials*** *Sections 1.2.2 and 2.1 for details). The differences in the population and households age composition by rural and urban settings are presented in section 2.2 in the* ***Supplementary Materials****.*

10. **15,-11; please state the magnitude of the difference, perhaps in % peak of epidemics or % change in time to peak, or in % change in attack rate. As with the original paper, this is an important resource, but its also a resource we will want to improve on over time. It’s therefore important to identify numerical targets for those improvements.**

Response and action: We thank the reviewer for this suggestion. We have included a figure in the Supplementary material (see **Supplementary Figure 5** below) tabulating the percentage change in attack rate of the interventions under the six different assumed mixing matrices.

**Supplementary Figure 5. Percentage change in case attack rate of COVID-19.**

*The percentage change in case attack rate in each of the three intervention scenario—20% physical distancing, 50% physical distancing, and lockdown—against the unmitigated epidemic under different contact matrices is shown in the boxplots with boxes bounded by the interquartile range (25th and 75th percentiles), median in white and, whiskers spanning the 2.5–97.5th percentiles (Figure 5). Six contact matrices were considered in the COVID-19 modelling: the empirically-constructed contact matrices at the study-year and adjusted for the 2020 population, the 2017 synthetic matrices, and the updated synthetic matrices at the national, rural, or urban settings.*

11. **17,-2; The discussion paragraph on time is important and should also reflect transient changes during, especially during the key initial phase of a pandemic.**

Response: We agree with the reviewer, which is why we have also updated the synthetic contact matrices with the most recent data during these revisions. We updated the data sources of the determinants of contacts in different countries like household size, labour participation, etc. We have acknowledged that using contact data from the POLYMOD survey published in 2008 is a limitation of our study. The POLYMOD survey has the advantage of contact matrices from eight countries using a standardised method. Unfortunately, no published, more recent large-scale multi-country study collects baseline contact pattern data using a standardised method. While these individual studies may be essential to explain the mixing patterns in their setting, it is challenging to harmonise a variety of surveys from different sources (with different survey tools and sampling methods) to inform our Bayesian hierarchical model. We have observed how the COVID-19 pandemic has engendered substantial changes to human behaviours, including contact patterns. During the pandemic, contact patterns are very different from the baseline whether due to non-pharmaceutical interventions (NPI) or reactive behaviours. Understanding the impact of the COVID-19 pandemic on contact patterns requires a detailed analysis of contact surveys conducted during the pandemic, taking into account both baseline contacts and NPI intensity. These data are starting to be collated (e.g. the pre-print review by Liu et al.^[9]^). Hence, synthetic matrices that project social contacts under different levels of NPIs (using proxies like mobility and NPI intensity) are needed; however, this is outside of the scope of this analysis. The COVID-19 pandemic has affected contact patterns, whether through NPI or reactive behavioural changes. Baseline, expected contact rates, as those inferred here, are critical for determining the amount of change in contact rates in response to the pandemic.

Action: We have since supplemented that paragraph in our **Discussion**, as follows:

*Another consideration affecting both synthetic and empirical matrices is that they change over time. Estimating synthetic matrices relies on the POLYMOD contact survey administered more than a decade ago. Another larger contact survey, BBC Pandemic (59,64) conducted in the UK used mobile phone-based GPS tracking instead of diary-based surveys, reported a decrease in contacts among adolescents compared to POLYMOD, which may reflect substitution of face-to-face contacts with electronic communication in this age group. Moreover, in addition to the rural-urban environment, age- and location-specific contact patterns could vary by socioeconomic conditions within countries. More differences are expected as countries implement physical distancing measures to mitigate the COVID-19 pandemic. The COVID-19 pandemic has affected contact patterns, whether through non-pharmaceutical interventions or reactive behavioural changes, in particular, how we come into contact with one another. Baseline, expected contact rates, as those inferred here, are critical for determining the amount of change in contact rates in response to the pandemic. Understanding the impact of the COVID-19 pandemic on contact patterns requires a detailed analysis of contact surveys conducted during the pandemic, taking into account baseline contacts and non-pharmaceutical interventions intensity. Future studies are also needed to quantify the possible long-term behavioural changes.*

We would like to thank the editors and reviewers for taking the time to suggest ways to improve the work and for the comments provided in this revision.

1. We thank the participants of <https://math.stackexchange.com/questions/2048817/metric-for-how-symmetric-a-matrix-is> for inspiring this definition.
2. Hoang T, Coletti P, Melegaro A, *et al.* A Systematic Review of Social Contact Surveys to Inform Transmission Models of Close-contact Infections. *Epidemiology* **30** (2019) doi: 10.1097/EDE.0000000000001047
3. Liu CY, Berlin J, Kiti MC, *et al.* Rapid review of social contact patterns during the COVID-19 pandemic *medRxiv* 2021.03.12.21253410; <https://doi.org/10.1101/2021.03.12.21253410>
4. Jarvis CI, Gimma A, van Zandvoort K *et al.* The impact of local and national restrictions in response to COVID-19 on social contacts in England: a longitudinal natural experiment. *BMC Med* **19,** 52 (2021). <https://doi.org/10.1186/s12916-021-01924-7>
5. Liu CY, Berlin J, Kiti MC, *et al.* Rapid review of social contact patterns during the COVID-19 pandemic *medRxiv* 2021.03.12.21253410; <https://doi.org/10.1101/2021.03.12.21253410>
6. Jarvis CI, Gimma A, van Zandvoort K *et al.* The impact of local and national restrictions in response to COVID-19 on social contacts in England: a longitudinal natural experiment. *BMC Med* **19,** 52 (2021). <https://doi.org/10.1186/s12916-021-01924-7>
7. Some examples of contact studies showing age assortative contact patterns: Mossong et al., 2008; Horby et al., 2011; Johnstone-Robertson et al., 2011; Kiti et al., 2014; Grijalva et al., 2015; Ajelli and Litvinova, 2017
8. European Centre for Disease Prevention and Control. Technical report: COVID-19 in children and the role of school settings in transmission -first update (2020) <https://www.ecdc.europa.eu/sites/default/files/documents/COVID-19-in-children-and-the-role-of-school-settings-in-transmission-first-update_1.pdf>
9. Liu CY, Berlin J, Kiti MC, *et al.* Rapid review of social contact patterns during the COVID-19 pandemic *medRxiv* 2021.03.12.21253410; <https://doi.org/10.1101/2021.03.12.21253410>
