# Supplementary material for: Correction: Projecting contact matrices in 177 geographical regions: An update and comparison with empirical data for the COVID-19 era
Source: PLoS Comput Biol. 2024 Sep 18;20(9):e1012454. doi: 10.1371/journal.pcbi.1012454 (PMC11410204; doi:10.1371/journal.pcbi.1012454)
Supplement: S3 File — (DOCX) [file pcbi.1012454.s003.docx]

Projecting contact matrices in 177 geographical regions: an update and comparison with empirical data for the COVID-19 era

Supplementary Material: Errata

Kiesha Prem, Kevin van Zandvoort, Petra Klepac, Rosalind M Eggo, Nicholas G Davies,

Centre for the Mathematical Modelling of Infectious Diseases COVID-19 Working Group,

Alex R Cook, Mark Jit^[[1]](#footnote-1)^

Table F highlights the changes and differences between urban and rural populations for each location, and Figures F and G illustrate this for two countries; Bolivia and France.

Table F: Summary of changes to the results following the errata.

| **Summary of changes** | **Effect of changes: urban vs rural** |
| --- | --- |
| **Age-specific contacts at home**   - When constructing HAM for countries without household age structured data, we had previously divided the HAM of a POLYMOD or DHS country by $P_{\alpha}^{c}$ instead of $P_{a}^{c}$. We have corrected the typographical mistake in the Supplementary Material (S1 Text section A.2.2.) and have updated the codes calculating HAM for countries without household age structure data - We defined $\lambda_{a_{i},\alpha}^{H}$ to allow for visitors to contribute to the total number of contacts through the parameter $\delta^{H}$. However, during the extrapolation of contacts at home, we had previously only accounted for household members, hence leaving out $\delta^{H}$ which represented visitors to the home from outside the household. We have now included $\delta^{H}$ in the calculations and text in S1 Text, previously missed in the extrapolation step (section A.5.). | The change in the contacts at home in urban and rural settings is similar (Supplementary Figures E and F top row). |
| **Age-specific contacts in the workplace**   - Extrapolation step to derive the frequency-dependent contact matrices at the workplace: we had previously written in the extrapolation step (in S1 Text section A8), contact matrices at the workplace were adjusted by age-specific population ratios in addition to the age-specific working and schooling populations. Because we cannot distinguish between visitors and work colleagues, as a result, we had to drop the $\delta^{W}$ parameter from the estimation of contact patterns at the workplace. - However, when deriving the age-specific contact matrices at the workplace and school using a frequency-dependent approach, we realised that this step is unnecessary. We have since removed this term in the text in the supplement. However, as not all working individuals went to work on the day of the survey, we adjusted the contact matrices at the workplace by the proportion of individuals who went to work on the day of the survey (changes to the equations in section A.5. of S1 Text). | The change in the contacts at the workplace in urban and rural settings is slightly different (Supplementary Figures E and F second row):   1. Urban: increase in contacts with younger age individuals at the workplace 2. Rural: minimal increase in contacts with younger age individuals at the workplace |
| **Age-specific contact in school**   - Extrapolation step to derive the frequency-dependent contact matrices at the school: we had previously written in the extrapolation step (in S1 Text section A.4.), contact matrices at the school were adjusted by age-specific population ratios in addition to the age-specific schooling populations. We revised how schooling populations are being constructed (changes to the equations in section A.4.3 of S1 Text). - Because we cannot distinguish between visitors and schoolmates, as a result, we had to drop the $\delta^{S}$ parameter from the estimation of contact patterns at school. When deriving the age-specific contact matrices at the school using a frequency-dependent approach, we realised that this step is unnecessary. We have since removed this term in the text in the supplement (in S1 Text sections A8 and A.5.). | The change in the contacts at schools in urban and rural settings are different (Supplementary Figures E and F third row):   1. Urban: increase in overall and age-assortative contacts 2. Rural: decrease in overall and age-assortative contacts   The changes in contacts at school are the most influential to the changes in overall contacts between urban and rural populations. |
| **Age-specific contact at other locations**   - We have corrected the calculations in the code (to reflect the equations in the methods) when we adjust the matrices by the age-specific population ratios in the extrapolation step for contacts at other locations. Previously we multiplied by the vector containing the age-specific population ratio to the rows of $\lambda_{a,\alpha}^{O}$matrix instead of the columns; this is now corrected | The change in the contacts at other locations in urban and rural settings is similar (Supplementary Figures E and F fourth row). |

| 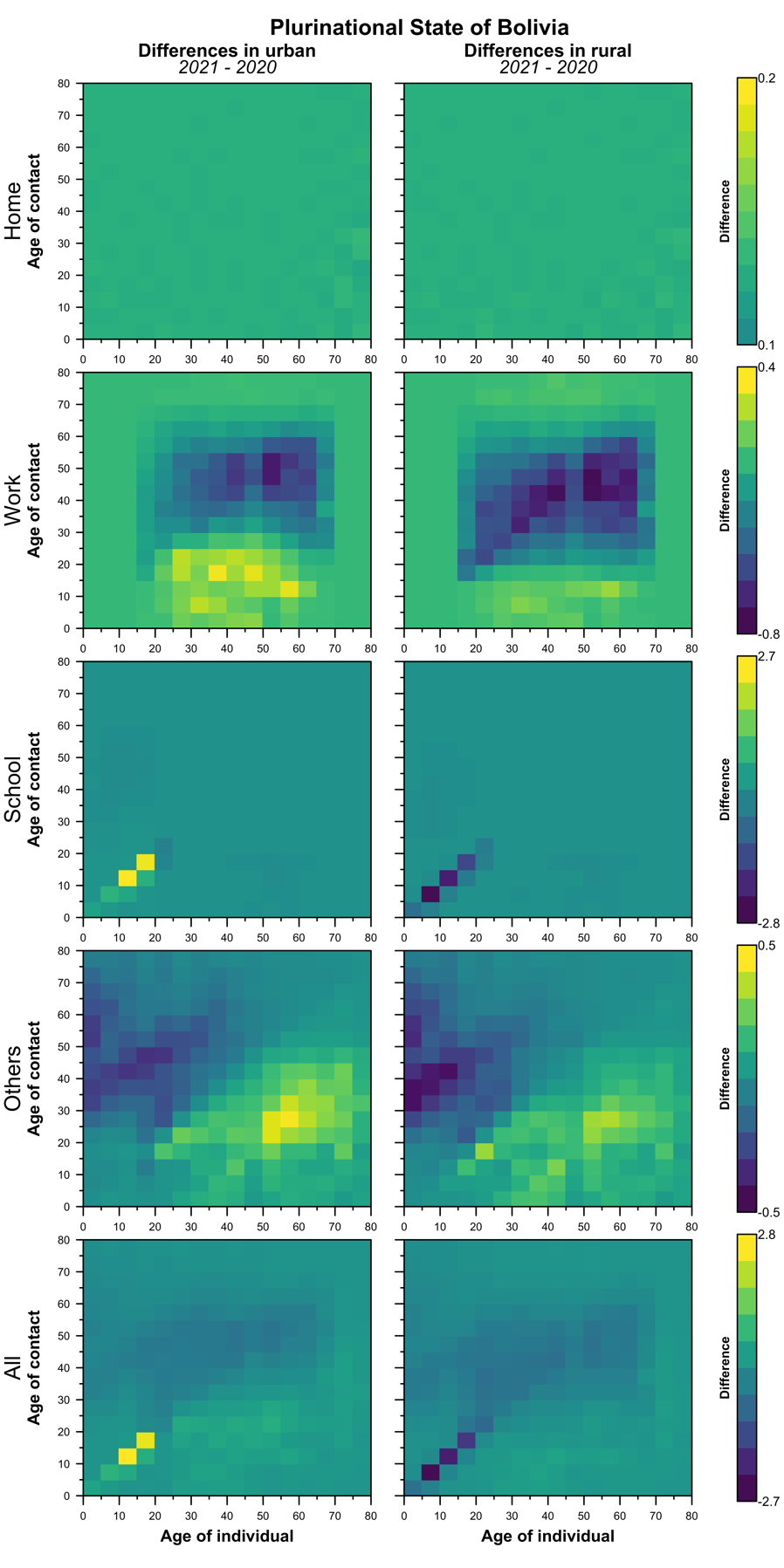 |
| --- |
| **Figure F.** Effect of changes on the contact matrices at rural and urban setting following the errata in the Plurinational State of Bolivia. |

| 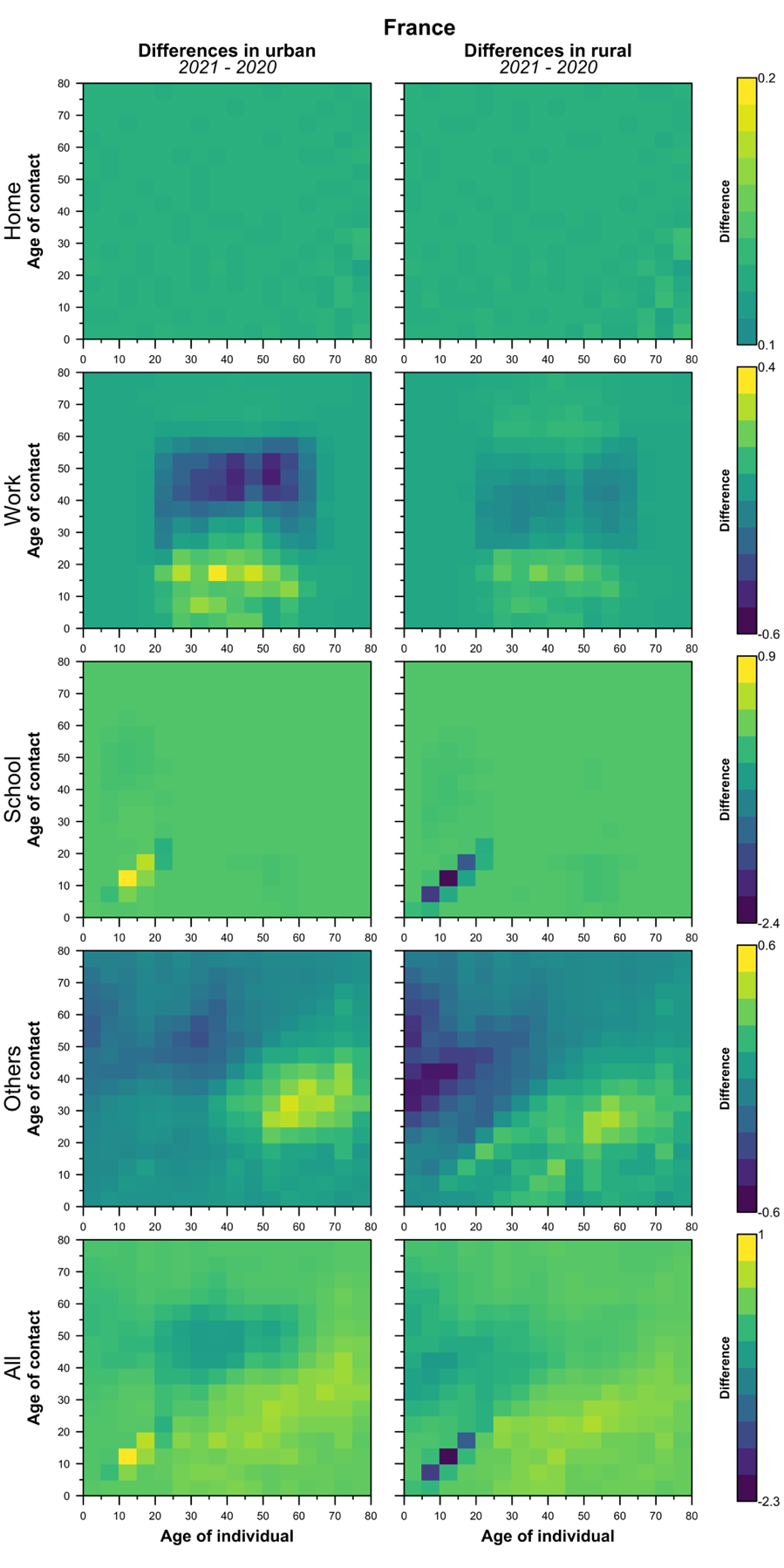 |
| --- |
| **Figure G.** Effect of changes on the contact matrices at rural and urban setting following the errata in France. |

1. Correspondence to [mark.jit@lshtm.ac.uk](mailto:mark.jit@lshtm.ac.uk). [↑](#footnote-ref-1)
